# Effectiveness of Terbutaline Pump for the Prevention of Preterm Birth. *A Systematic Review and Meta-Analysis*

**DOI:** 10.1371/journal.pone.0031679

**Published:** 2012-02-21

**Authors:** Laura M. Gaudet, Kavita Singh, Laura Weeks, Becky Skidmore, Alexander Tsertsvadze, Mohammed T. Ansari

**Affiliations:** 1 Evidence-Based Practice Center, Ottawa Hospital Research Institute, Ottawa, Ontario, Canada; 2 Horizon Health Network, Department of Obstetrics and Gynecology, The Moncton Hospital, Moncton, New Brunswick, Canada; 3 University of Ottawa, Ottawa, Ontario, Canada; Aga Khan University, Pakistan

## Abstract

**Background:**

Subcutaneous terbutaline (SQ terbutaline) infusion by pump is used in pregnant women as a prolonged (beyond 48–72 h) maintenance tocolytic following acute treatment of preterm contractions. The effectiveness and safety of this maintenance tocolysis have not been clearly established. We aimed to systematically evaluate the effectiveness and safety of subcutaneous (SQ) terbutaline infusion by pump for maintenance tocolysis.

**Methodology/Principal Findings:**

MEDLINE, EMBASE, CINAHL, the Cochrane Library, the Centre for Reviews and Dissemination databases, post-marketing surveillance data and grey literature were searched up to April 2011 for relevant experimental and observational studies.

Two randomized trials, one nonrandomized trial, and 11 observational studies met inclusion criteria. Non-comparative studies were considered only for pump-related harms. We excluded case-reports but sought FDA summaries of post-marketing surveillance data. Non-English records without an English abstract were excluded. Evidence of low strength from observational studies with risk of bias favored SQ terbutaline pump for the outcomes of delivery at <32 and <37 weeks, mean days of pregnancy prolongation, and neonatal death. Observational studies of medium to high risk of bias also demonstrated benefit for other surrogate outcomes, such as birthweight and neonatal intensive care unit (NICU) admission. Several cases of maternal deaths and maternal cardiovascular events have been reported in patients receiving terbutaline tocolysis.

**Conclusions/Significance:**

Although evidence suggests that pump therapy *may* be beneficial as maintenance tocolysis, our confidence in its validity and reproducibility is low, suggesting that its use should be limited to the research setting. Concerns regarding safety of therapy persist.

## Introduction

Preterm birth is defined as delivery before the completion of the 37th week of gestation and affects 13 percent (542 893 births in 2006) of live births in the United States (http://www.cdc.gov/nchs/fastats/birthwt.htm) [1]. Approximately 40 percent of preterm births occur after the spontaneous onset of preterm labor [2]. Long-term neonatal sequelae of prematurity such as bronchopulmonary dysplasia, grade III/IV intraventricular hemorrhage and retinopathy of prematurity determine the overall quality of life for the child and the family.

Terbutaline sulfate has been used off-label in selected patients as a maintenance therapy to inhibit uterine contractions for extended periods of time following primary tocolysis with first-line agents. Terbutaline, a β-sympathomimetic drug, acts to relax smooth muscle in the bronchial tree, blood vessels and myometrium [3]. Maternal side effects are common, and can include serious adverse reactions such as pulmonary edema, myocardial ischemia, cardiac arrhythmias, hypotension, and metabolic alterations [3]. Despite previous reviews which questioned the effectiveness and safety of subcutaneous terbutaline infusion, the use of such therapy is not uncommon in the United States [3], [4]. The exact frequency of use of subcutaneous terbutaline infusion for the prevention of preterm birth is not known.

This review, commissioned by the Agency for Healthcare Research and Quality through its established stakeholder topic nomination process, aims to systematically review and meta-analyze the evidence examining the efficacy, effectiveness, and harms of SQ terbutaline pump for preventing preterm labor, compared with placebo, conservative treatment, or any other active intervention. We investigated the clinical effectiveness and harms of pump therapy by systematically retrieving, appraising and synthesizing evidence on neonatal health outcomes and outcomes of maternal and neonatal harm. Surrogate outcomes, such as birthweight and prolongation of pregnancy were also examined. The potential confounding effects of maternal activity and maternal care on the above endpoints were explored, as was the incidence of pump-related outcomes. Particularly in light of a recent “black box” warning issued by the FDA, this review provides a contemporary and definitive summary of the available literature on the benefits and harms of terbutaline maintenance tocolysis.

## Methods

We followed a pre-specified and peer-reviewed study protocol (Information S1). The full evidence report (Information S2), including search strategies and a detailed list of *a priori* outcomes, risk of bias assessment and detailed evidence tables are available at www.effectivehealthcare.ahrq.gov/reports/final.cfm.

### Ethics

Ethics approval was not required for this review, as there is no potential for individual patient identification.

### Searching

We searched Ovid MEDLINE® In-Process & Other Non-Indexed Citations and Ovid MEDLINE® (1950 to April 1 2011); OVID EMBASE (1980 to April 1 2011); CINAHL via EBSCOhost (1985 to December 7, 2009), the Cochrane Library via the Wiley interface (April 1, 2011) (including CENTRAL, Cochrane Database of Systematic Reviews, DARE, HTA, and NHS EED), and the Centre for Reviews and Dissemination (CRD) databases (January 2, 2010). We hand-searched the bibliographies and text of review articles, letters to editors, and commentaries and the reference lists of included studies for additional references. We also reviewed grey literature sources and information received from pharmaceutical companies. Finally, we reviewed the FDA summary of post-marketing data to assess risk of maternal harm.

### Selection

Two reviewers screened abstracts and full-text reports with conflicts resolved by consensus or third party adjudication. Studies were included if they met the following criteria: evaluated pregnant women between 24–36 weeks gestation having had acute preterm labor arrested with primary tocolytic therapy; included at least one group that was administered SQ terbutaline pump; and assessed one of the specified outcomes (primary neonatal outcomes, surrogate outcomes, maternal harms, neonatal harms, pump-related outcomes and long-term childhood outcomes).

### Validity Assessment

For each study outcome, we assessed confounding and risk of selection, performance, detection and attrition bias. Selected items from the McMaster Quality Assessment Scale of Harms (http://hiru.mcmaster.ca/epc/mcharm.pdf) were also included. The overall risk of bias ratings were designated as high, medium, or low. Outcomes were rated as high risk of bias if there was a major flaw in the study. Separately, we evaluated the potential for financial conflict of interest.

Following published guidance for the Effective Health Care Program two reviewers graded the strength of evidence for incidence of delivery at various gestational ages, mean prolongation of pregnancy, bronchopulmonary dysplasia, significant intraventricular hemorrhage (grade III/IV), neonatal death, death within initial hospitalization, and maternal withdrawal due to adverse effects [5]. The guidance stipulates that the strength of evidence be rated as insufficient, low, moderate or high to reflect our confidence on the validity and reproducibility of evidence synthesis. According to the guidance, evidence was to be considered insufficient (i.e. inability to conclude) when there were no studies, studies showing opposite direction of effect, or when confidence intervals were wide enough to incorporate the possibilities of benefit, no difference or harms. Also, the strength of evidence based on observational studies conventionally starts off with a grade of low which is upgraded only when there is demonstration of a dose-response relationship, large effect size, or an effect despite confounding towards null. Generalisability or applicability of evidence was also rated as per previous guidance [6].

### Data Abstraction

One reviewer extracted data into a standardized electronic form and assessed study risk of bias and applicability. Extraction items included general study characteristics (e.g. year of publication, study design), population characteristics (e.g. inclusion/exclusion criteria, age, race, level of activity), intervention characteristics (e.g. dose, duration, details about comparators, level of care), and outcomes with their estimates. A second reviewer verified outcomes data and study risk of bias assessments. Ratings for level of care, level of activity, and assessments of applicability were verified by a clinical expert.

### Study Characteristics

Aside from case reports, all types of study designs were considered because evidence from experimental studies is often limited for reviews of comparative effectiveness [7]–[9]. Non-comparative studies (i.e. case series) were assessed only for pump-related harms, such as incidence of pump failure, missed doses, or overdose and maternal harms. Non-English language records without an English abstract were excluded. We also excluded case reports, but in a *post hoc* decision sought FDA summaries of post-marketing data highlighting serious harms.

Pump efficacy was examined for pre-specified subpopulations of women but harms were investigated across subgroups. The subgroups included women delivering extremely preterm (<28+0 weeks), very preterm (28+0 to 31+6 weeks), preterm (32+0 to 33+6 weeks), and later preterm (34+0 and 36+6 weeks); with multiple gestation; of different racial subgroups; with previous preterm birth; with history of preeclampsia and; with and without recurrent preterm labor.

### Quantitative Data Synthesis

We performed a meta-analysis of the RCTs with a random effects model when they were clinically and methodologically similar [10]. To assess statistical heterogeneity and its magnitude, we used Cochran's Q (α = 0.10) and the I^2^ statistic respectively. Odds ratios (ORs) were calculated for dichotomous outcomes and mean differences for continuous outcomes. Analyses were performed using Comprehensive Meta Analysis version 2.2.046 or version 2.2.055 (NJ, USA). We did not perform meta-analysis of the observational studies because of potential differences in confounders, nor did we combine studies of singleton and multiple pregnancies. Small number of included studies precluded meta-regression and exploration of heterogeneity in effect estimates.

## Results

### Flow of Included Studies

**Figure 1:**
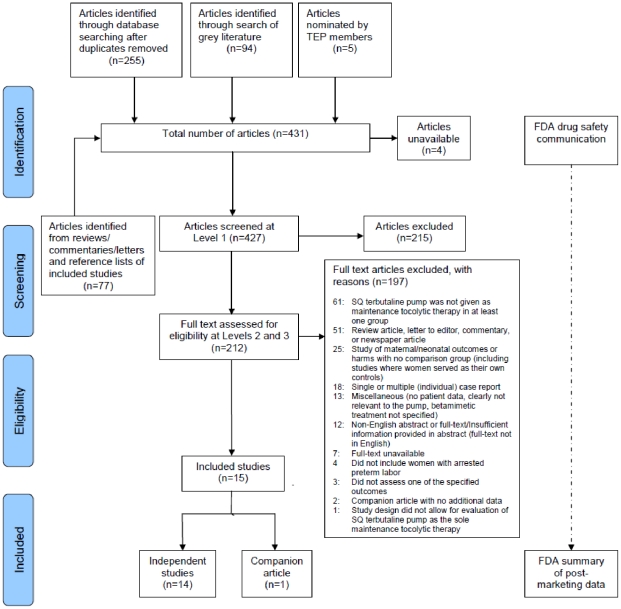
Flow of retrieved records through screening and inclusion.

The flow of retrieved records through the phases of literature screening is detailed in Figure 1. Fourteen independent studies and 1 companion article were included in the review [11], [12].

### Study Characteristics

Table 1 presents general summary characteristics of the included studies. Most studies were observational, and included cohorts and case series. Two studies were RCTs and one was a nonrandomized trial. Sample sizes ranged from 9 to 1 366, but over 70 percent of studies included at least 200 subjects (average 291±395). Despite meta-analysis, evidence from the two small RCTs remained underpowered for differences in the outcomes of benefit and harms (total N = 94).

**Table 1:** Summary characteristics of the included studies.

CHARACTERISTIC		NUMBER OF STUDIES	REFERENCES
Study Design	RCT	2	[21], [24]
	Nonrandomized trial	1	[22]
	Prospective Cohort	2	[13], [14]
	Retrospective Cohort	7	[11], [15]–[19], [25]
	Case Series	2	[20], [23]
Participant Recruitment	Single Center Sites	9	[11], [13], [14], [20]–[25]
	Matria Database	5	[15]–[19]
Funding	Industry	2	[21], [23]
	NonIndustry	3	[11], [14], [25]
	Not Reported	9	[13], [15]–[20], [22], [24]
Comparator*	Oral Nifedipine	3	[15]–[17]
	Oral Terbutaline	4	[11], [22], [24], [25]
	Oral Tocolytics	3	[14], [18], [19]
	Placebo (saline pump)	2	[21], [24]
	No treatment	1	[13]
	No comparison group	2	[20], [23]
Primary Tocolytic Treatment	IV Magnesium Sulfate only	1	[20]
	IV Magnesium Sulfate and/or other agents	5	[13], [21]–[24]
	Not Reported	8	[11], [14]–[19], [25]
Gestation	Singletons only	6	[13], [15], [17], [18], [21], [22]
	Twins only	2	[16], [19]
	Singletons and Twins	2	[23], [24]
	Not Reported	4	[11], [14], [20], [25]
Definition of Labor	Not reported	5	[15], [17]–[19], [25]
Risk of Bias*	Low	1	[21]
	Medium	7	[11], [16], [17], [19], [20], [22], [23]
	High	7	[13]–[15], [18], [22], [24], [25]
Outcomes	Improvement of Neonatal Outcomes	6	[13], [17]–[19], [21], [24]
	Improvement of Surrogate Outcomes	12	[11], [13]–[19], [21], [22], [24], [25]
	Maternal Harm Outcomes	6	[13], [18], [19], [21], [22], [25]
	Neonatal Harm Outcomes	1	[24]
	Outcomes of pump failure	3	[20], [23], [24]

Abbreviations: RCT = randomized controlled trial; IV = intravenous.

*One study contained two comparison groups [24]. Risk of bias of one study differed by outcome [22].

All studies were from the United States and patients were recruited either from single center study sites or from a national proprietary database run by Matria Healthcare. This database provides an outpatient perinatal program consisting of 24-hour nursing and pharmacy support, home uterine activity monitoring, individualized education, and provision of tocolytic therapy to women with preterm labor. Because five studies originated in the Matria database, and not all reported geographic region and/or years over which participants were recruited, the question of overlap in patients across these studies was an important concern. Comparator groups included placebo, no treatment, oral terbutaline, oral nifedipine, and mixed oral tocolytics.

The definition of labor was unclear in 36 percent of the included studies. The remaining studies included women with persistent contractions and cervical change. Parenteral magnesium sulfate was often the primary tocolytic to arrest acute preterm labor. In several studies, only women with two or more episodes of preterm labor (i.e. recurrent preterm labor) were eligible for inclusion [11], [13]–[20]. Some studies were conducted exclusively in women with singleton gestation [13], [15], [17], [18], [21], [22], while a few studies evaluated women with twins only [16], [19]. Some studies may have included women less than 24 weeks gestational age, but data for such participants could not be separated [11], [15]–[19], .

Maternal characteristics of participants in the included studies are summarized in Table 2. No studies presented data on concomitant medications, body mass index (BMI), history of preeclampsia, cervical position, cervical consistency, cervical station, Bishop's Score, or fetal fibronectin.

**Table 2:** Maternal characteristics.

Characteristic	Number of Studies that Reported Characteristic	Value
Mean maternal age	12 [11], [13]–[16], [18], [19], [21]–[25]	21.6–32.4 years
Mean GA at preterm labor	6 [13], [15]–[19]	29.5–31.6 weeks
Mean GA at start of therapy	6 [11], [20]–[24]	29.1–32.2 weeks
Race	5 [11], [13], [19], [21], [25] *	European(white); Hispanic; African; Asian; Other (“nonwhite”)
Comorbidities	2 [13], [23]	bacterial vaginosis; asthma; urinary tract infection; fibroids; chronic hypertension/pregnancy induced hypertension; HELLP syndrome
History of preterm birth	7 [13], [16]–[19], [21], [23]	10.8–75%
Cerclage	6 [15]–[19], [23]	2.8–13.1%
Gravidity	2 [18], [24]	2.6 (mean) [18]; 2.25, 2.5, 2.6 (medians) [24]
Parity	3 [11], [14], [24]	1.2 (mean) [11], 1.4 (mean) [14]; 0.5, 0.5, 0.7 (medians) [24]
Membrane status	8 [11], [13]–[16], [20], [21], [23]	Intact
Mean cervical length	1 [20]	0.2 cm
Mean cervical dilation	5 [11], [13], [20], [21], [24]	1.7–2.9 cm
Cervical effacement	1 [21]	50% (median)

Abbreviations: GA = gestational age; HELLP syndrome = hemolysis, elevated liver function values, low platelet count.

*None of these studies reported separate effect estimates for different races.

### Risk of Bias Assessment

Studies with important group imbalances in baseline characteristics or prognostic factors were rated as high risk of bias (Table 1). Those with no identifiable flaws but with incomplete reporting of information for risk assessment were judged to be of medium risk of bias. Although the randomization procedures in the two RCTs were appropriate, we rated one RCT as high risk of bias because more than 90 percent of eligible participants declined to participate, the study was underpowered, and blinding was ineffective. The two case series were judged to be of medium risk of bias because neither study provided clear definitions for the pump-related harm outcomes and several criteria such as compliance, adequacy of sample size, and selective outcome reporting, were unclear.

### Quantitative Data Synthesis

#### Neonatal Health Outcomes

One retrospective cohort with medium risk of bias, presented evidence of low strength suggesting SQ terbutaline pump may decrease the risk of neonatal death compared with oral tocolytics in women with recurrent preterm labor and twin gestation (OR = 0.09, 95 percent CI: 0.01, 0.70) (Table 3) [19].

**Table 3:** Strength of evidence for the outcomes of interest.

Outcome	Population; Comparator	N_Studies_	N_Subjects_	N_Events_	Effect Estimate	SoE Domains*	SoE
Neonatal death†	Twins+RPTL; Oral tocolytics	1 [19]	706	12	OR = 0.09 (0.01, 0.70)	Medium; N/A; Direct; Precise	Low
	Singleton+RPTL; Oral nifedipine	1 [17]	284	0	OR = 1.00 (0.02, 50.75)	Medium; N/A; Direct; Imprecise	Insufficient
Significant IVH (Grade III/IV)†	Singleton+RPTL; No treatment	1 [13]	60	4	OR = 0.30 (0.02, 5.85)	High; N/A; Direct; Imprecise	Insufficient
Incidence of delivery <32 weeks	Twins+RPTL; Oral nifedipine	1 [16]	656	192	OR = 0.47 (0.33, 0.68)	Medium; N/A; Indirect; Precise	Low
	Twins+RPTL; Oral tocolytics	1 [19]	706	124	OR = 0.52 (0.35, 0.76)	Medium; N/A; Indirect; Precise	Low
	Singleton + RPTL; Oral nifedipine	2 [15], [17]	1650	106	OR = 0.20–0.29 (0.07–0.16, 0.52–0.61)‡	High/Medium; Consistent; Indirect; Precise	Low
	Singleton+RPTL; Oral tocolytics	1 [18]	558	37	OR = 0.21 (0.09, 0.50)	High; N/A; Indirect; Precise	Low
	Singleton+RPTL; No treatment	1 [13]	60	21	OR = 0.04 (0.00, 0.65)	High; N/A; Indirect; Precise	Low
Incidence of delivery <37 weeks†	Singleton+RPTL; Oral nifedipine	2 [15], [17]	1650	925	OR = 0.72–0.75 (0.47–0.58, 0.90–1.20)‡	High/Medium; Consistent; Indirect; Imprecise	Insufficient
	Singleton+RPTL; Oral tocolytics	1 [18]	558	318	OR = 0.70 (0.50, 0.98)	High; N/A; Indirect; Precise	Low
	Singleton+RPTL; No treatment	1 [13]	60	50	OR = 0.04 (0.01, 0.23)	High; N/A; Indirect; Precise	Low
	Singleton/Multiple+RPTL; Oral terbutaline	1 [11]	64	38	OR = 0.10 (0.03, 0.32)	Medium; N/A; Indirect; Precise	Low
Pregnancy prolongation (days)†	Twins+RPTL; Oral nifedipine	1 [16]	656	N/A	MD = 7.20 (4.10, 10.30)	Medium; N/A; Indirect; Precise	Low
	Singleton+RPTL; Oral nifedipine	2 [15], [17]	1650	N/A	MD = 6.20–7.50 (0.79–4.94, 10.06–11.61)‡	High/Medium; Consistent; Indirect; Imprecise	Insufficient
	Singleton+RPTL; Oral tocolytics	1 [18]	558	N/A	MD = 5.50 (2.28, 8.72)	High; N/A; Indirect; Precise	Low
	Singleton+RPTL; No treatment	1 [13]	60	N/A	MD = 25.30 (16.77, 33.83)	High; N/A; Indirect; Precise	Low

Abbreviations: IVH = intraventricular hemorrhage; MD = mean difference; N = number; N/A = not applicable; OR = odds ratio; RPTL = recurrent preterm labor; SoE = strength of evidence.

*SoE domains are presented in the following order: Risk of Bias; Consistency; Directness; Precision.

† RCT evidence was available for neonatal death [24], significant IVH [21], delivery <37 weeks [21], and pregnancy prolongation [21], [24]. There were no events of neonatal death or significant IVH. Nonsignificant differences were reported for incidence of delivery <37 weeks and pregnancy prolongation. The RCT evidence was not graded because it did not apply to any of the subgroups of interest.

‡ These studies were not pooled. There was risk of double counting of participants across these studies.

Three retrospective cohort studies reported non-significant differences in rate of stillbirth in women with recurrent preterm labor and single or twin gestation [17]–[19]. However, these studies were likely underpowered, given the small number of events (<1%). Sparse evidence from underpowered studies addressed clinically important neonatal outcomes including necrotizing enterocolitis, retinopathy of prematurity, and sepsis. Results were, therefore, inconclusive [13], [24]. No data were available for bronchopulmonary dysplasia, death within initial hospitalization, periventricular leukomalacia, and seizures.

#### Mean Gestational Age at Delivery

**Figure 2:**
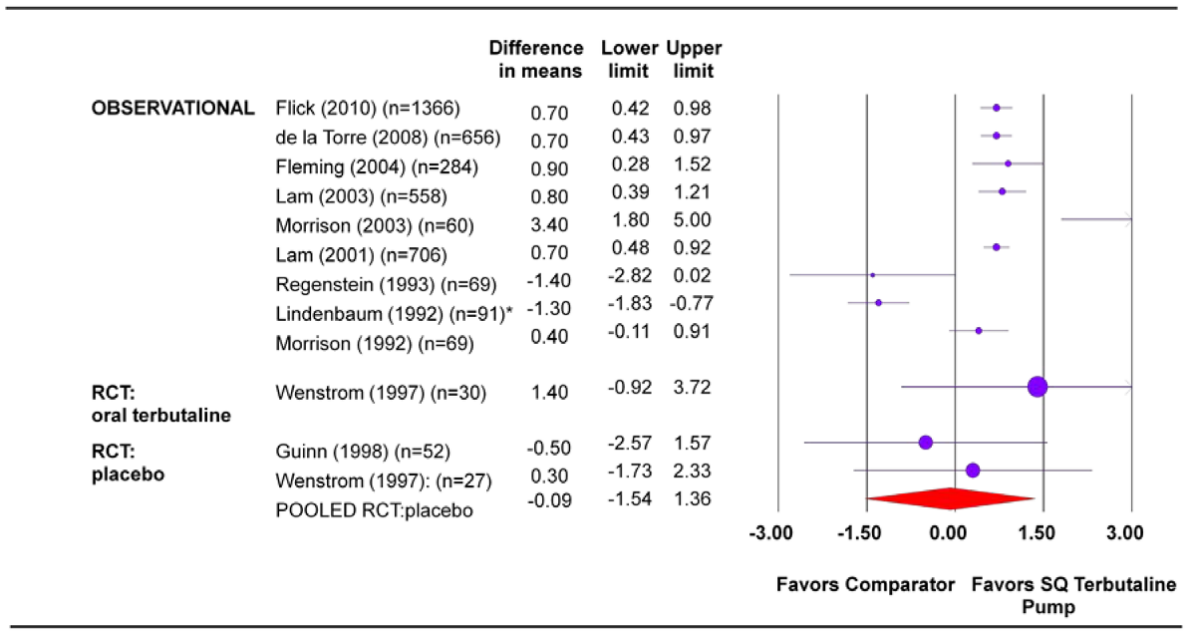
Mean gestational age at delivery (weeks). Statistical heterogeneity for the RCT pooled estimate: I^2^ = 0.0 percent, p-value>0.05. * There were discrepancies in the information presented in the text and table of this paper [22]. Mean gestational age at delivery for SQ terbutaline pump was reported as 36.6 weeks in table (as reported above) and 37.2 weeks in text. The value 36.6 weeks was used to calculate difference in means. SQ = subcutaneous.

Larger cohort studies in women with recurrent preterm labor and single or twin gestation demonstrated consistent benefit of SQ terbutaline pump compared with oral tocolytics or no treatment (although there is a high risk of bias in the available data) (Figure 2). For women with recurrent preterm labor and singleton pregnancies, the difference in gestational age ranged from 0.70 to 3.40 weeks (95% CI, lower bound range 0.28 to 1.80; upper bound range 0.98 to 5.00) favoring SQ terbutaline pump. In pregnancies complicated by recurrent preterm labor in twins, the difference in gestational age from two cohorts was 0.70 weeks (95 percent CI, lower bound range 0.43 to 0.48; upper bound range 0.92 to 0.97) (Table 4) [13], [15]–[19].

**Table 4:** Summary table for mean gestational age at delivery.

Study Design (number of studies)	Population	Comparator(s)	Risk of Bias
RCT (2)	Women with singleton gestation from Birmingham Hospital (n = 52) [21]	Placebo	Low
	Women with singleton or twin gestation from the University of Iowa Hospital (n = 42) [24]	Placebo and oral terbutaline	High
Nonrandomized Trial (1)	Women with singleton gestation from the Hospital of the University of Pennsylvania (n = 91) [22]	Oral terbutaline	High
Prospective Cohort (2)	Women with singleton gestation and RPTL (n = 60) [13]	No treatment	High
	Likely included a mixture of women with single and multiple gestation (n = 69) [14]	Oral tocolytics	High
Retrospective Cohort (6)	Women with singleton gestation and RPTL from the Matria database (n = 1366) [15]	Oral nifedipine	High
	Women with singleton gestation and RPTL from the Matria database (n = 284) [17]	Oral nifedipine	Medium
	Women with singleton gestation and RPTL from the Matria database (n = 558) [18]	Oral tocolytics (95.3% received oral terbutaline)	High
	Women with twin gestation and RPTL from the Matria database (n = 656) [16]	Oral nifedipine	Medium
	Women with twin gestation and RPTL from the Matria database (n = 706) [19]	Oral tocolytics (92.3% received oral terbutaline)	Medium
	Likely included a mixture of women with single and multiple gestation (n = 69) [25]	Oral terbutaline	High

Abbreviations: RCT = randomized controlled trial; RPTL = recurrent preterm labor.

#### Incidence of Delivery at Various Gestational Ages

As with other outcomes, the strength of evidence surrounding gestational age at delivery was low. The SQ terbutaline pump consistently appeared to reduce the odds of delivering <32 weeks in women with recurrent preterm labor with twin pregnancies in the six Matria-based cohort studies (Table 3). The odds ratios ranged from 0.04 to 0.52 (95% CI, lower bound range 0.00 to 0.35; upper bound range 0.50 to 0.76) [13], [15]–[19].

The risk of any preterm delivery (<37 weeks) was also assessed. Low strength of evidence favored SQ terbutaline pump compared with oral tocolytics or no treatment in women with recurrent preterm labor (Table 3). Four of five cohort studies with important risk of bias reported statistically significant reduction in the odds of delivery <37 weeks (OR 0.04 to 0.75; 95 percent CI, lower bound range 0.01 to 0.58; upper bound range 0.23 to 1.20) [11], [13], [15], [17], [18].

#### Prolongation of Pregnancy

As with other outcomes, the strength of evidence for prolongation of pregnancy was insufficient or low (Table 3). Evidence favored SQ terbutaline pump in women with recurrent preterm labor or twin gestation in cohort studies. The range for mean number of days of pregnancy prolongation was 5.50 to 25.30 (95 percent CI, lower bound range 0.79 to 16.77; upper bound range 8.72 to 33.83) (Table 3) [13], [15]–[18]. This evidence came from five cohort studies of medium to high risk of bias. In one Matria-based cohort study, more women in the SQ terbutaline pump group had pregnancy prolonged >7 days compared with women who received oral nifedipine (OR = 7.84, 95 percent CI, 3.59, 17.12) [15]. Other Matria-based studies reported statistically significant benefits in favor of the pump compared with oral tocolytics for prolongation >14 days (OR range = 1.93 to 3.47, 95 percent CI, lower bound range 0.87 to 2.34; upper bound range 2.65 to 5.15) [15]–[19].

#### Birthweight

**Figure 3:**
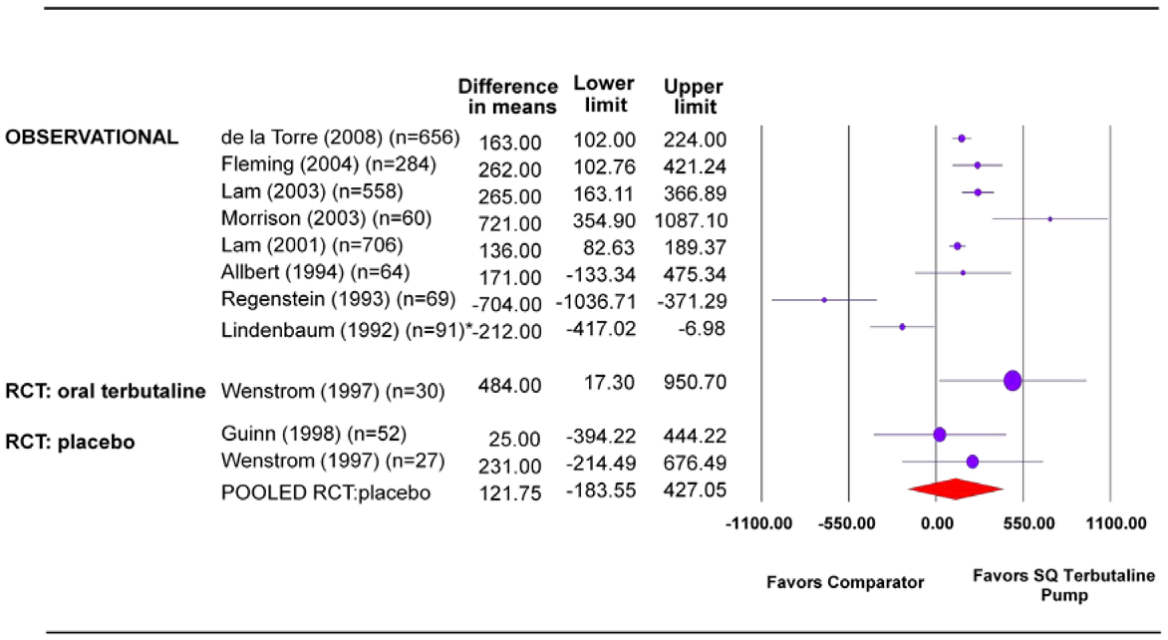
Mean birthweight (grams). Statistical heterogeneity for the RCT pooled estimate: I^2^ = 0.0 percent, p-value>0.05. * There were discrepancies in the information presented in the text and table of this paper [22]. The numbers reported in the table were used to calculate the difference in means. However, the text reported group data with numbers switched for the groups (i.e. SQ terbutaline pump: 3229±584 and oral terbutaline: 3017±303). SQ = subcutaneous.

Cohort studies of women with recurrent preterm labor and single or twin gestation demonstrated statistically significant differences in mean birthweight in favor of SQ terbutaline pump compared with oral tocolytics or no treatment (range of mean difference in grams = 136 to 721, 95 percent CI, lower bound range 83 to 355; upper bound range 189 to 1087) (Figure 3 and Table 5) [13], [16]–[19].

**Table 5:** Summary table for mean birthweight.

Study Design (number of studies)	Population	Comparator(s)	Risk of Bias
RCT (2)	Women with singleton gestation from Birmingham Hospital (n = 52) [21]	Placebo	Low
	Women with singleton or twin gestation from the University of Iowa Hospital (n = 42) [24]	Placebo and oral terbutaline	High
Nonrandomized Trial (1)	Women with singleton gestation from the Hospital of the University of Pennsylvania (n = 91) [22]	Oral terbutaline	High
Prospective Cohort (1)	Women with singleton gestation and RPTL (n = 60) [13]	No treatment	High
Retrospective Cohort (6)	Women with singleton gestation and RPTL from the Matria database (n = 284) [17]	Oral nifedipine	Medium
	Women with singleton gestation and RPTL from the Matria database (n = 558) [18]	Oral tocolytics (95.3% received oral terbutaline)	High
	Women with twin gestation and RPTL from the Matria database (n = 656) [16]	Oral nifedipine	Medium
	Women with twin gestation and RPTL from the Matria database (n = 706) [19]	Oral tocolytics (92.3% received oral terbutaline)	Medium
	Women with RPTL and likely included a mixture of women with single and multiple gestation (n = 64) [11]	Oral terbutaline	Medium
	Women with single or multiple gestation (n = 69) [25]	Oral terbutaline	High

Abbreviations: RCT = randomized controlled trial; RPTL = recurrent preterm labor.

Studies reporting the incidence of low birthweight (<2500 g) found statistically significant differences in favor of SQ terbutaline pump compared with no treatment or oral tocolytics (OR range = 0.24 to 0.64, 95 percent CI, lower bound range 0.06 to 0.51; upper bound range 0.62 to 0.96) [13], [15]–[19]. Most of the studies reporting the incidence of very low birthweight (<1500 g) also found statistically significant differences in favor of the pump (OR range = 0.22 to 0.46, 95 percent CI, lower bound range 0.07 to 0.29; upper bound range 0.60 to 1.06) [16]–[19]. The studies that reported birthweight were mostly of medium or high risk of bias.

#### NICU Admission

For incidence of NICU admission, statistically significant differences favoring SQ terbutaline pump were reported in studies of medium or high risk of bias (OR range 0.28 to 0.72, 95 percent CI, lower bound range 0.08 to 0.58; upper bound range 0.63 to 0.97) [13], [15]–[19]. Statistically significant differences in favor of SQ terbutaline pump were also reported for NICU length of stay in these cohort studies (range of mean difference in days: −3.50 to −17.90, 95 percent CI, lower bound range −5.26 to −32.88; upper bound range −1.74 to −3.54) [13], [15], [18], [19].

#### Other Surrogate Outcomes

One cohort study reported a non-significant difference between SQ terbutaline pump and oral tocolytics in requirement for ventilation among infants with NICU admission [18]. Pregnancy prolongation index was reported in two cohorts studies of medium and high risk of bias [11], [13]. Both found statistically significant differences in favor of SQ terbutaline pump compared with either no treatment or oral terbutaline (mean difference range = 0.14 to 0.41, 95 percent CI, lower bound range 0.26 to 0.56; upper bound range 0.02 to 0.26).

#### Maternal Harms

There were no reports of maternal death in the included studies. Underpowered studies demonstrated indeterminate results for pulmonary edema, therapy discontinuation and maternal hyperglycemia (i.e., type II error cannot be excluded) [13], [18], [19], [21], [22], [25]. One prospective cohort of women with singleton pregnancies and recurrent preterm labor suggested that pump use was associated with increased tachycardia/nervousness (OR = 25.48, 95 percent CI:1.23, 526.6) [13]. No data within the study settings were identified for withdrawal due to adverse events, heart failure, hypokalemia, myocardial infarction and refractory hypotension.

By 2009, 16 maternal deaths and 12 cases of maternal cardiovascular events (hypertension, myocardial infarction, tachycardia, arrhythmias and pulmonary edema) in patients receiving terbutaline tocolysis had been reported to the FDA. Three of the maternal deaths and three cardiovascular adverse events were reported in patients receiving SQ terbutaline pump therapy (http://www.fda.gov/Drugs/DrugSafety/ucm243539.htm).

#### Neonatal Harms

Data for neonatal harms were very sparse. Only one small RCT comparing SQ terbutaline pump with placebo and oral terbutaline demonstrated non-significant differences for neonatal hypoglycemia.

#### Incidence of Pump Failure

Two case series and one RCT reported outcomes related to the pump device [20], [23], [24]. In a case series of 51 women, one subject had dislodgment of catheter (2 percent, exact central CI: 0.5 percent, 10 percent) and there was one pump malfunction (2 percent, exact central CI: 0.5 percent, 10 percent) [23]. No infusion site infections or mechanical failures were observed in a case series of 9 women [20]. An underpowered RCT demonstrated indeterminate results for the outcomes of local pain and local skin irritation [24]. No data were available for missed doses or overdoses.

#### Assessment of Confounding by Level of Activity and Level of Care

Only a small number of studies could be rated for level of activity and level of care, precluding an exploration of the effect of these variables on maternal and neonatal outcomes. Qualitative assessments did not yield any further insights.

#### Applicability

Table 6 summarizes the applicability of the body of evidence. The majority of evidence pertained to women with recurrent preterm labor and singleton gestation. Very little is known about the study population's demographic and clinical characteristics, placing significant restrictions on the generalisability of results. Furthermore, the possibility that subjects represented a selective group of participants remains a concern.

**Table 6:** Applicability of the body of evidence.

**Population**	The majority of evidence pertained to women with recurrent preterm labor and singleton gestation in the United States. Very little information was reported about the study populations' demographic and clinical characteristics. Nine of 14 studies (64 percent) included women judged to be in labor on account of persistent contractions and cervical change. The definition of labor was unclear in other studies. Among the studies that suggested that the pump was efficacious, 50 percent reported cervical change and contractions as part of the definition of labor while 50 percent did not report how labor was defined.
**Intervention**	Although there were gaps in reporting, the intervention generally did not pose any serious limitations to applicability. Very few details were reported on cointerventions that could modify the effectiveness of therapy, such as administration of corticosteroids. In several studies, participants received specialized outpatient services from Matria Healthcare.
**Comparison**	Comparators included oral tocolytics, no treatment, and placebo.
**Outcomes**	Surrogate outcomes were the most commonly reported. Data on clinical outcomes, neonatal/maternal harms, and pump-related outcomes were sparse. Long-term outcomes have not been reported at all.
**Timing of Outcomes Measurement**	The absence of follow-up beyond delivery is a major limitation because important long-term outcomes have not been evaluated.
**Setting**	All studies were from the United States and participant data were acquired from a national database (Matria) or from single center sites. Women from the Matria database generally received a high level of care from an outpatient perinatal program. However, the distribution of regions from which patient data were included into the national database is unknown and information about the standards followed by the individual practice sites that provided obstetrical care was not reported. Similarly, for those studies that took place at single center sites, the standards of care followed at these sites are unclear.

## Discussion

Across several outcomes, the evidence favors subcutaneous terbutaline pump as maintenance tocolytic therapy for women with arrested preterm labor. However, our confidence in the validity and reproducibility of this evidence is low. Most of the evidence came from biased observational studies that reported surrogate outcomes only. Furthermore, the safety of the pump therapy remains unclear, largely because studies lacked power to detect differences in outcomes of harm.

A total of 14 unique studies comprised the body of evidence investigating efficacy and harms of SQ terbutaline pump therapy as maintenance tocolysis in women with arrested preterm labor. Evidence from the only two included RCTs was underpowered to detect differences in outcomes of efficacy and harms. Most data came from observational studies, several of which recruited subjects from a single Matria database. These studies were at significant risk of bias and exhibited considerable clinical and methodological diversity.

While a single study demonstrated improvement in neonatal death in women with recurrent preterm labor and twin gestation receiving SQ terbutaline maintenance tocolysis, several studies presented evidence favoring the pump therapy on surrogate outcomes of preterm birth. However, the evidence for important neonatal health outcomes, neonatal harms, and maternal harms was inconclusive because the studies lacked power to detect differences in clinical events. Furthermore, there was no data on the long-term effects of terbutaline infusion on offspring. Although many decisions regarding SQ terbutaline pump are currently made on the assumption that short-term outcomes will correlate well with improved long-term outcomes, rigorous scientific evaluation is needed to confirm whether such factors lead to better outcomes in this population.

These findings are consistent with those of two existing reviews of SQ terbutaline pump [3], [4]. As reported by Nanda et al., we found that the available RCT evidence demonstrated non-significant differences between the pump and placebo or oral terbutaline [3]. In agreement with another review, we found that the RCT and observational evidence is conflicting [4]. We noted that the RCT evidence did not demonstrate any benefit from the pump while cohort studies of limited methodological validity demonstrated statistically significant effects in favor of the pump for several outcomes.

Based on post-marketing surveillance data, the FDA has issued a new warning against the use of terbutaline in general, and particularly as an injection, as maintenance tocolysis (i.e. beyond 48–72 hours) in pregnant women. The warning is a response to several cases of poor maternal outcomes in pregnancies treated with subcutaneous terbutaline. These cases raised concern regarding a potential causative relationship. It is important to consider that the outcomes in question occur infrequently in the pregnant population, even in the absence of terbutaline use. Assessment of the magnitude of the association between terbutaline use and harm continues to be challenging, given the rarity of events and the lack of good quality, well powered studies. Until this relationship is further delineated, use of terbutaline for the prevention of preterm birth should be limited to carefully-controlled study settings.

### Limitations

The evidence base for this review contained several limitations. Most of the evidence originated from observational study designs with significant risk of bias. Important prognostic factors such as race, socioeconomic status, and fetal fibronectin level were not reported and co-interventions, such as administration of corticosteroids, were rarely described. Moreover, it is uncertain how free the available evidence is from confounding imposed by restriction of maternal activity and level of care. While our review of the literature was comprehensive in capturing comparative evidence estimating the magnitude of benefits and harms of pump therapy, one potential and practical limitation was restriction to evidence reported in the English language.

There are several factors of applicability that should be considered by maternity care providers and policymakers when translating the evidence from this review. The majority of available evidence included women with recurrent preterm labor (i.e. those with arrested preterm labor following first-line tocolytic therapy for 48 hours and then presenting with a second episode) and singleton gestation, with some evidence including women with twin gestation and recurrent preterm labor. Several studies included patients from a national proprietary database run by Matria Healthcare, which provides an outpatient perinatal program consisting of 24-hour nursing and pharmacy support, home uterine activity monitoring, individualized education, and provision of tocolytic therapy to women with preterm labor. These women received a high standard of care.

It is notoriously difficult to conduct trials to assess the efficacy of tocolytics. Studies regarding tocolytics have been plagued by the elusive diagnosis of preterm labor as up to 40 percent of women diagnosed with preterm labor may not actually be in labor [26]. As such, a significant proportion of women enrolled in clinical trials of tocolytic efficacy may not be destined to deliver preterm. A definitive trial in this domain must include a focus on accurate diagnosis of preterm labor, perhaps, combining stringent clinical criteria with factors such as positive fetal fibronectin and shortened trans-vaginal cervical length. Outcomes to be investigated should go beyond those of prolongation of pregnancy and birthweight to hard clinical endpoints of neonatal morbidity. Furthermore, the trial should include long-term follow-up to assess subsequent childhood outcomes. It is evidence of improvements in clinical effectiveness outcomes that can impact clinical decision making, societal healthcare costs and guideline recommendations.

In conclusion, our systematic review calls into question the evidence base supporting the current practice of using terbutaline pump as a maintenance tocolytic agent. We feel strongly that use of terbutaline infusion for maintenance tocolysis should be restricted to well-designed, carefully-controlled study settings until there is clear evidence supporting its use. Further, decision and policy makers should take into consideration the limitations of the available data, both in terms of benefit and harm, when formulating recommendations.

## Supporting Information

Information S1 **Study Protocol.**
(DOC)

Information S2 **Full Evidence Report.**
(PDF)
