# Supplementary material for: Effectiveness of Terbutaline Pump for the Prevention of Preterm Birth. A Systematic Review and Meta-Analysis
Source: PLoS One. 2012 Feb 21;7(2):e31679. doi: 10.1371/journal.pone.0031679 (PMC3283660; doi:10.1371/journal.pone.0031679)
Supplement: Information S1 — Study Protocol. (DOC) [file pone.0031679.s001.doc]

# Evidence-based Practice Center Systematic Review Protocol

# Project Title: Comparative Effectiveness of Terbutaline Pump for the Prevention of Preterm Birth

I. Background and Objectives for the Systematic Review

Preterm birth continues to be the largest contributor to neonatal morbidity and mortality and is associated with both short and long term disability. Preterm birth is defined as delivery before the completion of the 37th week of gestation and affects 12 percent of live births in the United States (more than a half a million births per year) .^1^ Rates of preterm birth continue to increase, resulting in a significant disease burden to the health care system.

Tocolytic therapy is aimed at arresting preterm labor with the goal of reducing neonatal morbidity and mortality by prolonging pregnancy without causing significant maternal or neonatal side effects. First-line tocolytic therapy is focused on short-term delay in delivery to allow for maternal administration of corticosteroids and transport to an appropriate facility for neonatal care. Maintenance tocolytic therapy is focused on preventing recurrent preterm labor once a primary episode of confirmed preterm labor has been arrested. Tocolytic therapy, regardless of agent or route of administration, has thus far demonstrated poor efficacy, likely because the parturitional process is already well established. The most appropriate measure to assess the efficacy of tocolytic agents should focus on improved health outcomes for infants; yet, most tocolytic trials to date have insufficient power to assess this endpoint. Surrogate endpoints (such as delay in delivery) are therefore common and meta-analyses are used to assess efficacy and safety.

Terbutaline sulfate is commonly used in selected patients as a maintenance therapy to inhibit uterine contractions for longer periods of time and thus prevent recurrent preterm labor. Therapy is initiated in women who have an arrest of preterm labor after initial treatment with first-line tocolytics. Terbutaline sulfate can be administered orally, intravenously, by subcutaneous shots, or via a subcutaneous pump. Administration by subcutaneous pump is usually dosed at a basal rate of 0.03-0.05 mg/hr with an intermittent bolus of 0.25 mg every 4 to 6 hours.^2^ This is an off-label use of the drug, which is approved by the United States Food and Drug Administration for use in the management of acute and chronic obstructive pulmonary disease only.

Terbutaline is a beta-sympathomimetic agent that relaxes smooth muscle in the bronchial tree, blood vessels, and myometrium by stimulating beta-receptors .^2^  Because terbutaline does not act on the myometrium alone, maternal side effects are common and can include pulmonary edema, myocardial ischemia, cardiac arrhythmias, hypotension, and metabolic alterations.^2^ Most effects are mild and self-limiting, however, such as shortness of breath, chest pain, anxiety, and fatigue^3^; but serious cardiopulmonary and metabolic complications have been reported[[1]](#footnote-2).

The Cochrane Collaboration regularly conducts meta-analyses of tocolytic drugs and has previously assessed terbutaline pump maintenance therapy.^4^ The authors of this review concluded that terbutaline delivered by a subcutaneous pump has not been shown to decrease the risk of preterm birth by prolonging pregnancy. Further, they indicated that the lack of information on the safety of the treatment as well as the cost associated with its use, argues against its use in clinical management of arrested preterm labor. Unfortunately, the two included trials were small (Guinn n=52 and Wenstrom n=42). This review was last updated in 2007.

In 2006, a Hayes Brief was published that included both trials in the Cochrane review and also four existing observational studies .^5^ Results were contradictory, with randomized trials failing to show efficacy and observational studies demonstrating positive results.

Given the prevalence of preterm birth and its associated morbidity, mortality and costs, a systematic review of this topic should prove useful for clarifying the equipoise regarding the efficacy, effectiveness, and safety of the use of the terbutaline pump for the prevention of preterm birth. A scoping literature search suggests that no randomized controlled trials have been published since the last Cochrane update. As such, we will include data from observational studies in our review. We expect this review should also elucidate the populations for which further randomized clinical research is most urgently needed.

A comprehensive evidence synthesis on the terbutaline pump for the prevention of pre-term birth has been commissioned by the Agency for Healthcare Research and Quality (AHRQ). As of 1990, terbutaline sulfate was one of the most widely prescribed tocolytics used to prevent preterm birth.^6^ It is, however, not currently approved by the United States Food and Drug Administration for use during pregnancy. Evidence regarding the efficacy and effectiveness of terbutaline pump is contradictory^4,5^ and evidence regarding the safety of terbutaline pump has not yet been synthesized. In general, it appears that there is a large amount of uncertainty surrounding the use of terbutaline and tocolytics. Overall, there is no well-established, clear, first-line therapy for tocolysis. Tocolytics have been shown to delay delivery 24 to 48 hours; however, significant maternal side effects and no effect on perinatal outcomes have been demonstrated. Further, in a recent cost analysis of four tocolytic agents, terbutaline had the highest cost due to the need for increased monitoring and adverse events associated with this therapy.[[2]](#footnote-3) Given the lack of efficacy, effectiveness, and safety data, as well as substantial cost, it is clear that variation in clinical practice and dilemmas in health policy decision making exist in regards to the use of terbutaline generally and terbutaline pump specifically for the prevention of preterm birth.

This evidence synthesis will focus on the efficacy, effectiveness, and safety of subcutaneous terbutaline delivered continuously by an infusion pump in terms of neonatal health outcomes, and maternal and neonatal harms. Further, as the level of care offered to patients and the level of activity of patients on continuous subcutaneous terbutaline might differ from that of patients on comparative treatments, we will explore these two variables as potential confounders in our analysis. Finally, we will also explore the practical utility of the pump by assessing incidence of pump failure, for example in terms of missed doses, dislodgment, and overdose.

II. The Key Questions

A. Introduction

The following key questions were posted for public comment on the AHRQ website from February 10, 2010 through March 10, 2010. No changes to the questions were made following that process, except to correct a typographical error in an outcome related to maternal harms. In the public posting of the key questions the outcome was listed as “hyperkalemia”, but should have been listed as “hypokalemia”. We have corrected this typographical error in the key questions listed below. The three comments received following the public posting process are summarized below along with a response from the EPC:

1. A formal comment was submitted on behalf of the American Congress of Obstetricians and Gynecologists (ACOG) and the American College of Obstetricians and Gynecologists (The College), which represent more than 53,000 physicians and partners dedicated to improving women's health.

*The comment indicated that both ACOG and the College agree the key questions appropriately represent their concerns regarding the research on the use of the terbutaline pump. No changes were made to the key questions as a result of this comment.*

1. A question raised by an individual concerned whether the review will examine outcomes specific to women with decreased cervical length and/or women with a positive fetal fibronectin test for Key Question 1.

*Data on both cervical length and fetal fibronectin will be extracted during the review and will be included in a meta-regression, if possible, to explore reasons for heterogeneity in effect sizes. No changes were made to the key questions as a result of this comment.*

1. An anonymous individual questioned whether we would explore an increase in the risk of autism spectrum disorder as a neonatal outcome related to Key Question 4.

*We have subsequently reviewed the literature related to autism spectrum disorder and determined that this outcome is more appropriately related to terbutaline as a drug, and not the terbutaline pump per se, which is the focus of our review. While we agree there might be benefits to investigating serious and rare adverse events in an hypothesis generating setting (based on non-comparative observational designs), the investigation does not particularly fit within the remit of a hypothesis testing comparative effectiveness review. Therefore, autism as a long-term outcome would be outside the scope of the current review which specifically examines the comparative effectiveness of the terbutaline pump. No changes were made to the key questions as a result of this comment.*

***Modification to the Key Questions subsequent to TEP #1 teleconference, held June 4, 2010:***

The following modifications have been made to the key questions:

1. Women with/without RPTL has been added as a subgroup (subgroup i) to key questions 1 and 2.
2. Ratio of birthweight/gestational age at delivery and PPI have been added as outcomes to key question 2.

*Point 1:*

During the TEP call it was suggested that women with threatened preterm labour and true preterm labour be differentiated in subgroup analyses. The presence of RPTL was mentioned as a possible indicator for women experiencing true preterm labour. Therefore, in addition to conducting heterogeneity analyses by other indicators of true preterm labour (i.e. cervical length and cervical dilation), we will also explore whether effect estimates vary among those studies that specify RPTL as an inclusion criterion (as a surrogate for study populations with true preterm labour) versus those studies that do not specify RPTL as an inclusion criterion (as a surrogate for study populations with threatened preterm labour).

*Point 2:*

These outcomes were suggested by the TEP as additional outcomes of interest.

B. Development of the Key Questions

The following considerations guided the development of the final Key Questions.

- Population(s):

Pregnant women diagnosed with arrest of preterm labor after primary tocolytic treatment (24-36 weeks gestation), including subgroups of <28 weeks gestation, <32 weeks, <34 weeks and <37 weeks, as well as multiple gestations, racial subgroups, and women with a history of preterm birth and preeclampsia.

- Interventions:

Subcutaneous terbutaline (Bricanyl, Brethine, generic terbutaline sulfate) delivered by infusion pump. Any dose and frequency of administration will be considered, as long as delivery is by infusion pump.

- Comparators:

Placebo, conservative treatment, or another intervention

- Outcomes for each question
  - Primary outcomes

Clinical neonatal outcomes (bronchopulmonary dysplasia, necrotizing enterocolitis, significant intraventricular hemorrhage, periventricular leukomalacia, seizures, retinopathy of prematurity, sepsis, stillbirth, death within initial hospitalization, neonatal death)

- - Secondary outcomes

Gestational age at delivery, incidence of delivery at various gestational ages (<28 weeks, <32 weeks, <34 weeks, <37 weeks), mean prolongation of pregnancy (days), birthweight, ratio of birthweight/gestational age at delivery, pregnancy prolongation index (PPI), need for assisted ventilation, need for oxygen per nasal cannula, NICU admission

- - Adverse events

Maternal side effects (pulmonary edema, heart failure, arrhythmia, myocardial infarction, hypokalemia, hyperglycemia, refractory hypotension), maternal withdrawal due to adverse effects (WDAE), maternal discontinuation of therapy, maternal death, pediatric side effects (hypoglycemia, hypocalcaemia, ileus).

- Timing

Duration of followup will depend on the data reported in the literature. All information available on neonatal outcomes will be assessed. It is anticipated that the majority of studies will limit followup to the immediate neonatal admission.

- Settings:

In general, this intervention is managed by tertiary care centers, while the patient is at home. However, if some studies are conducted in a primary care or community setting, these will also be included. Cointerventions will likely include restriction of maternal activities.

C. Key Questions

In women with arrested preterm labor, does treatment with a subcutaneous infusion of terbutaline delivered by a pump, in comparison with placebo, conservative treatment or other interventions:

KQ1: improve neonatal health outcomes, including bronchopulmonary dysplasia, necrotizing enterocolitis, significant intraventricular hemorrhage (grade III/IV), periventricular leukomalacia, seizures, retinopathy of prematurity, sepsis, stillbirth, death within initial hospitalization and neonatal death, for the following subgroups:

a. women <28 +0 weeks of gestation (extremely preterm)?

b. women between 28 +0 and 31 +6 weeks of gestation (very preterm)?

c. women between 32 +0 and 33 +6 weeks of gestation (preterm)?

d. women between 34 +0 and 36 +6 weeks of gestation (later preterm)?

e. multiple gestations?

f. racial subgroups?

g. women with previous preterm birth?

h. women with history of preeclampsia?

i. women with recurrent preterm labour (RPTL) and women without RPTL

KQ2: improve other outcomes, including gestational age at delivery, incidence of delivery at various gestational ages (<28 weeks, < 32 weeks, <34 weeks, <37 weeks), mean prolongation of pregnancy (days), birthweight, ratio of birthweight/gestational age at delivery, PPI,, need for assisted ventilation, need for oxygen per nasal cannula, NICU admission, for the following subgroups:

a. women <28 +0 weeks of gestation (extremely preterm)?

b. women between 28 +0 and 31 +6 weeks of gestation (very preterm)?

c. women between 32 +0 and 33 +6 weeks of gestation (preterm)?

d. women between 34 +0 and 36 +6 weeks of gestation (later preterm)?

e. multiple gestations?

f. racial subgroups?

g. women with previous preterm birth?

h. women with history of preeclampsia?

i. women with RPTL and women without RPTL

KQ3: increase the maternal harms of pulmonary edema, heart failure, arrhythmia, myocardial infarction, refractory hypotension, hypokalemia, hyperglycemia, and maternal deaths, or result in an increased rate of maternal withdrawal due to adverse effects (WDAE) and maternal discontinuation of therapy?

KQ4: increase the neonatal harms of hypoglycemia, hypocalcemia, and ileus?

KQ5: Can the differences in the outcomes above be partially explained by the differences in level of care (e.g. frequency of follow up, nurse visits, concomitant treatment, etc.) and level of activity (e.g. other children in the home, marital/support status, working status, bed rest, etc.) between the terbutaline pump group and the comparator group?

KQ6: What is the incidence of failure of the pump device used for terbutaline infusion, including missed doses, dislodgment, and overdose?

III. Analytic Framework

**Surrogate Outcomes:**

- Gestational age at delivery
- Incidence of delivery (<28, <32, <34, <37 weeks)
- Mean prolongation of pregnancy (days)
- Need for assisted ventilation
- Need for oxygen per nasal cannula
- NICU admission
- Birthweight
- Ratio of birthweight/gestational age at delivery
- PPI

**(KQ2)**

**(KQ1)**

**Pregnant women with arrest of preterm labor (24-36 weeks gestation)**

**Neonatal Outcomes***

- Bronchopulmonary dysplasia
- Necrotizing enterocolitis
- Grade III/IV Intraventricular hemorrhage
- Periventricular leukomalacia
- Seizures
- Sepsis
- Stillbirth
- Retinopathy of prematurity
- Death within initial hospitalization
- Neonatal death

Treatment with Terbutaline pump as compared to placebo, conservative treatment or other interventions

**(KQ3, KQ4)**

**Maternal Side Effects**

Pulmonary edema

Heart failure

Arrhythmia

Myocardial infarction

Hypotension

Hypokalemia

Hyperglycemia

WDAE

Maternal discontinuation of therapy

Maternal death

**Neonatal Side Effects**

Hypoglycemia

Hypocalcemia

Ileus

**(KQ6)**

Important Subgroups:

KQ1a, KQ2a: <28 weeks gestation

KQ2a, KQ2b: < 32 weeks gestation

KQ1c, KQ2c: <34 weeks gestation

KQ1d, KQ2d: <37 weeks gestation

KQ1e, KQ2e: multiple gestations

KQ1f, KQ2f: racial subgroups

KQ1g, KQ2g: history of preterm birth

KQ1h, KQ2h: history of preeclampsia

KQ1i, KQ2i: RPTL

**Pump Failure**

**Confounders (level of care, level of activity and co-interventions)**

**(KQ5)**

***Contributors to morbidity and mortality of infants born preterm**

IV. Methods

A. Criteria for Inclusion/Exclusion of Studies in the Review

Studies published in the English language conducted in any setting will be included. Studies will not be excluded based on publication status or publication date. Further inclusion criteria are outlined in Table 1.

Table 1: Screening Criteria

| Study Characteristic | Of Interest | Not of interest |
| --- | --- | --- |
| Patient population | - Pregnant women diagnosed with arrest of preterm labor after primary tocolytic treatment - Singleton or multiple gestations - 24-36 +6 weeks gestation - Intact membranes | - Premature rupture of membranes |
| Intervention  - Treatment | - Subcutaneous terbutaline delivered by infusion pump as maintenance therapy | - Oral terbutaline - Intravenous terbutaline - Subcutaneous terbutaline as first-line tocolytic - Subcutaneous terbutaline as part of a combined treatment protocol (i.e. combined with other tocolytics) |
| Intervention  - Comparison | - Placebo - Conservative treatment (i.e. no active treatment) - Other interventions |  |
| Outcomes – neonatal health outcomes | - Bronchopulmonary dysplasia - Necrotizing enterocolitis - Significant intraventricular hemorrhage (grade III/IV) - Periventricular leukomalacia - Seizures - Retinopathy of prematurity - Sepsis - Stillbirth - Death within initial hospitalization - Neonatal death |  |
| Outcomes – other health outcomes | - Gestational age at delivery - Incidence of delivery at <28 weeks, <32 weeks, <34 weeks and <37 weeks gestational age - Mean prolongation of pregnancy (days) - Birthweight - Ratio of birthweight/gestational age at delivery - PPI - Need for assisted ventilation - Need for oxygen per nasal cannula - NICU admission |  |
| Outcomes – maternal harms | - Pulmonary edema - Heart failure - Arrhythmia - Myocardial infarction - Refractory hypotension - Hypokalemia - Hyperglycemia - Maternal withdrawal due to adverse effects - Maternal discontinuation of therapy - Maternal death |  |
| Outcomes – neonatal harms | - Hypoglycemia - Hypocalcemia - Ileus |  |
| Outcomes – incidence of pump failure | - Missed doses - Dislodgment - Overdose |  |
| Study design | - Controlled trials (randomized and non-randomized) - Observational studies   - Prospective and retrospective cohort studies   - Case-control studies (prospective studies only for pump failure)   - Cross-sectional (all outcomes other than pump failure) - Non-comparative studies but not case reports (only pump failures) | - Case studies |

We have conducted a scoping literature search and reviewed relevant systematic reviews, which demonstrate that few randomized controlled trials (RCTs) are available on the topic. Further, the two RCTs included in a published Cochrane review had small sample sizes (52 and 42 participants) and short followup periods. Finally, published RCTs do not provide the necessary insight into questions regarding the safety of terbutaline. Therefore, in an effort to gain as much insight as possible into our key questions, observational studies will be included in the review. Observational studies identified through our scoping literature search consistently have larger sample sizes than published RCTs and in some cases include longer term outcomes. Further, observational studies have been identified that explore issues of terbutaline safety. We will conduct a thorough assessment of the risk of bias of all included studies so that we are well positioned to discuss the extent and limitations of all available evidence.

B. Searching for the Evidence: Literature Search Strategies for Identification of Relevant Studies to Answer the Key Questions.

Electronic search strategies will be developed and tested by an experienced medical information specialist in consultation with the EPC team. The search strategy will be peer reviewed according to the PRESS guideline.^7^ Published literature will be identified through searching MEDLINE, EMBASE, CINAHL, and the databases of The Cochrane Library. The strategy for MEDLINE is included as Appendix 1 and will be translated as appropriate for the other databases. The search strategies combine the use of controlled vocabulary and keywords. There are no language or date restrictions and no study design filters are used. A test of the strategy revealed that the use of study design filters did not substantially alter the number of hits and so, to be more comprehensive, study design filters will not be used. Animal studies will be excluded.

Grey (unpublished or difficult to locate) literature will be identified through searching the Web sites of relevant specialty societies and organizations, specialized public-access databases (e.g., TRIP), Health Technology Assessment agencies, guideline collections, trial registries, and conferences.

Additional references will be sought through scanning the reference list of systematic reviews on tocolytics in general or terbutaline in particular.

Two reviewers will independently screen all identified items at two levels. Level 1 screening will entail a broad screen based on item titles and/or abstracts, as available. The full-text of all items passing Level 1 screening will be retrieved for Level 2 screening: an ascertainment of final eligibility for the review. Discrepancies will be resolved by consensus or by involving a third team member. All screening will be conducted in DistillerSR using a priori developed inclusion criteria as described above.

C. Data Abstraction and Data Management

All data will be extracted by one reviewer and a second reviewer will subsequently verify all outcome data, quality assessments, applicability assessments, and ratings for maternal level of activity and level of care. Discrepancies will be resolved by consensus or by involving a third team member. Data extractors will not be blinded to any study information.

Before data extraction begins, a standardized data extraction form and data extraction guidelines will be iteratively developed and pilot tested until an appropriate level of consensus is reached. Further, all data extractors will meet in-person to discuss each extraction item as a means to ensure consistency and understanding of the process and definitions to be used for each item.

For all studies, the following data will be extracted:

- Study characteristics: first author, year of publication, country of origin, funding source, study design, study setting, duration of followup
- Population characteristics: inclusion/exclusion criteria, maternal sample characteristics (e.g., age, gender, race, gravidity, parity, co-morbidities, marital/support status, working status), singleton vs. multiple gestations, gestational age at preterm labor, definition of gestational age used/means of dating pregnancy, first-line tocolytic treatment used dosing schedule (e.g., indomethacin at a specific dose and number of doses), previous maintenance tocolytic therapy, presence of washout period after receiving beta-mimetic therapy (and before initiating pump), history of preterm birth, history of preeclampsia, cervical dilation, mean/median cervical length, cervical effacement, cervical position, cervical consistency, cervical station, Bishop’s Score, mean/median fetal fibronectin levels, level of activity (e.g., other children in the home, bed rest), , presence or absence of recurrent preterm labour prior to intervention, and body mass index (BMI).
- Intervention characteristics: dose (including concentration of terbutaline for basal infusion and boluses, rate of delivery, dose per injection, number of injections per day), duration of treatment, compliance, co-interventions (e.g., restriction of maternal activities, intensive guidance, home contraction monitoring), study protocol for subsequent episodes of preterm labor, concomitant medications (e.g., other β-adrenergic agents) timing of co-interventions, level of care (e.g., protocol for managing episodes of recurrent preterm labor, frequency of follow up, nurse visits, supervision of home treatment, daily phone calls from nursing staff, individualized dosage schedule, home uterine activity monitoring, regular changes in site of subcutaneous infusion), training to administer pump (e.g., number of hours, number of sessions, type of trainer), terbutaline manufacturer, infusion pump type and manufacturer
- Comparison intervention characteristics: intervention description, dose, duration of treatment
- Outcomes: In addition to quantitative data for each of the following outcomes, definitions used within each study to categorize each outcome will be extracted. Further, data will be extracted as number of patients with an event (as opposed to number of events) to ensure only one event is recorded per patient, in the case the same patient experiences multiple events:
  - KQ1: bronchopulmonary dysplasia, necrotizing enterocolitis, significant intraventricular hemorrhage (grade III/IV), periventricular leukomalacia, seizures, retinopathy of prematurity, sepsis, stillbirth, death within initial hospitalization and neonatal death
  - KQ2: gestational age at delivery, incidence of delivery at various gestational ages (<28 weeks, <32 weeks, <34 weeks, <37 weeks), mean prolongation of pregnancy, birthweight, ratio of birthweight/gestational age at delivery, PPI, need for assisted ventilation, need for oxygen per nasal cannula, NICU admission
  - KQ3: pulmonary edema, heart failure, arrhythmia, myocardial infarction, refractory hypotension, hypokalemia, and hyperglycemia, maternal withdrawal due to adverse effects (WDAE), maternal discontinuation of therapy (including reasons for discontinuation, if reported), maternal death
  - KQ4: hypoglycemia, hypocalcemia, ileus
  - KQ6: missed doses, dislodgment, overdose, infection, allergic reaction, thrombosis at site of infection
  - Other long-term childhood outcomes: childhood development, neurobehavioural testing, long-term lung function, long-term vision, other long-term outcomes reported in primary studies.

We will seek additional information from authors when necessary. Data will be extracted using an electronic and online screening and data abstraction software (DistillerSR).

D. Assessment of Methodological Quality of Individual Studies

The quality of included studies will be assessed using AHRQ’s recommended generic quality criteria for both controlled trials and observational studies.^8^ In addition, further criteria have been added specific to studies assessing harms of treatment by incorporating selected criteria of the McMaster Quality Assessment Scale of Harms(McHarm) into the quality assessment checklist.^9^ Criteria to assess study quality are summarized in Table 2.

Table 2: Criteria to assess risk of bias within included studies

| Criteria | Randomized Controlled trials | Observational studies (excluding case series)/  Nonrandomized trials | Case Series |
| --- | --- | --- | --- |
| Similarity of groups at baseline in terms of baseline characteristics and prognostic factors | x | x |  |
| Similarity of groups in terms of administration of primary tocolytic regimen to control acute episode of preterm labour | x | x |  |
| Extent to which valid primary outcomes are described | x | x | x |
| Blinding of subjects and providers to treatment allocation | x |  |  |
| Blinded assessment of outcomes | x |  |  |
| Blinding of providers to frequency and intensity of maternal contractions | x | x | x |
| Conduct of an intention-to-treat (ITT) analysis | x | x |  |
| Differential loss to followup between the compared groups or overall high loss to followup | x | x | x |
| Differential level of care between the compared groups^1^ | x | x |  |
| Conflict of interest | x | x | x |
| Selective outcome reporting^2^ | x | x | x |
| Data quality^3^ | x | x | x |
| Randomization sequence generation | x |  |  |
| Allocation concealment^4^ | x |  |  |
| Adequate sample size | x | x | x |
| Selection of participants |  | x |  |
| Compliance with treatment regimen | x | x | x |
| Methods to control for important confounders^5^ |  | x |  |
| Criteria for assessment of neonatal or maternal harms: definition of harms, mode of harms collection (i.e. active or passive), training/background of personnel collecting harms data (from the McHarm checklist) | x | x |  |

^1^ Will include an assessment of all level of care variables outlined in the Data Extraction section, including the study protocol for management of recurrent preterm labor.

^2^ We will assess selective outcome reporting through a comparison of the outcomes reported to be measured in the methods section of the study report and those reported in the results section.

^3^ Will include an assessment of the consistency of measurements across outcome assessors and, for retrospective studies, consistency in outcome definitions used across data sources.

^4^ Will include an assessment of both the knowledge of and the predictability of participant allocation to study interventions, and an assessment of the potential for selection bias based on the ability to predict the allocation sequence.

^5^ We will consider some important confounders to include: age, race, socioeconomic status, comorbidities, restriction of maternal activities, history of preterm birth, cervical length, cervical dilation, and fetal fibronectin.

Data on study quality will be extracted simultaneously with other study related data as outlined above. As with data extraction, study quality will be assessed by one reviewer and verified by a second reviewer, with discrepancies being resolved by consensus or involvement of a third team member. When data regarding study quality is extracted, reviewers will also be prompted to make comments regarding the potential implications for each identified source of potential bias on the study results. The methodology to assess ITT, loss to followup, and confounding by indication, is described in more detail below.

*Assessment of ITT:*

An assessment of ITT will be made for the following study designs:

- Randomized controlled trials
- Nonrandomized controlled/comparative trials
- Prospective cohort studies
- Retrospective cohort studies

In an ITT analysis, all subjects who have been assigned or included in an intervention group at the outset of a study are analyzed within that group even if subjects were lost to followup/discontinued treatment or switched over to an alternate group. Therefore, to determine if an ITT analysis has been conducted in these study designs, we will assess whether *both* loss to followup/discontinuation of treatment and unintended crossover to opposite intervention group(s) were accounted for in the results. Loss to followup will be assessed according to the process described below. Unintended crossover to opposite intervention group(s) will be assessed based on information reported in the paper; if no information has been reported, we will *not* assume that crossover has *not* occurred and, accordingly, we will indicate that it is unclear whether an ITT analysis was conducted.

Although the ITT principle may apply to case series designs, an assessment of ITT would only include loss to followup/discontinuation of treatment since crossover to an alternate intervention group is not possible. Therefore, we will only be assessing loss to followup/discontinuation of treatment for case series, as described below.

*Assessment of Loss to Followup:*

Since the reported sample size could represent either the total number of subjects recruited based on inclusion/exclusion criteria or the number of subjects *left* in the study after loss to followup has already occurred, the language used in the study must be assessed carefully. When not clearly reported in the paper, loss to followup will be assessed by comparing the number of subjects who entered the study with the number of subjects reported in outcome table(s) for the situations described below; if outcome table(s) report the same number of subjects in each intervention group as was present at the beginning of the study, then we will indicate no loss to followup.

- A retrospective cohort study that reports the number of subjects screened, the number of subjects excluded and the reasons for exclusion, and the number of subjects who met inclusion/exclusion criteria.
- A retrospective study that reports the number of subjects identified to have met inclusion/exclusion criteria within a defined time interval for data collection.
- A prospective cohort study, case series, or RCT that reports the number of subjects recruited based on inclusion/exclusion criteria within a specified time interval.

If a study simply reports that *x* number of subjects were analyzed or that *x* number participated in the study, then the outcome table cannot be relied upon to assess loss to followup and our assessment will be unclear.

A two-step process will be followed to make a complete assessment of study quality. First, the risk of bias (quality) of an *individual* study will be assessed as good, fair, or poor given the study design (e.g. RCT, cohort, etc)^8^; this assessment will be made separately for all relevant outcomes reported in the study. Second, studies will be aggregated by outcome and assigned a rating of low, medium, or high to describe the *overall* risk of bias; this overall rating will take into consideration study design and aggregate of quality ratings made in the previous step. Study quality will not be used as an exclusion criterion for this review; however, overall assessments will be used to guide interpretation of study results and to highlight important limitations in the body of evidence on this topic.

Table 3: Quality assessment categories for individual studies.^8^

**Good (low risk of bias).**  These studies have the least bias and results are considered valid. A study that adheres mostly to the commonly held concepts of high quality including the following: a formal randomized controlled study; clear description of the population, setting, interventions, and comparison groups; appropriate measurement of outcomes; appropriate statistical and analytic methods and reporting; no reporting errors; low dropout rate; and clear reporting of dropouts.

**Fair.** These studies are susceptible to some bias, but it is not sufficient to invalidate the results. They do not meet all the criteria required for a rating of good quality because they have some deficiencies, but no flaw is likely to cause major bias. The study may be missing information, making it difficult to assess limitations and potential problems.

**Poor (high risk of bias).**  These studies have significant flaws that imply biases of various types that may invalidate the results. They have serious errors in design, analysis, or reporting; large amounts of missing information; or discrepancies in reporting.

E. Applicability

Applicability assessments will be made following the PICOTs format during the data extraction process using relevant criteria as recommended by AHRQ.^8^

Applicability data will be extracted for each included study and individual assessments of applicability will be made at the study level. Summary applicability tables by study outcome across all included studies will be developed and reported alongside evidence tables.^8^ Clinical experts will verify all applicability assessments within and across studies. Important limitations to applicability will be highlighted during the interpretation of study results.

F. Data Synthesis

**Key questions 1-4**

Our ability to answer Key Questions 1 through 4 will be limited by the type and format of outcome data reported in primary studies. We have been comprehensive in our approach to developing a list of outcomes; however, it is possible and perhaps likely that evidence will not be available regarding each of the above outcomes from the small number of studies we expect to include. Further, the type and format of outcome data reported will most likely limit the potential for analyses by predefined subgroups as planned.

Quantitative synthesis

Data will only be pooled from studies without substantial clinical and methodological diversity, that are of similar quality, and that include similar comparison groups. Further, cointerventions must be deemed similar enough in all treatment and comparator groups to warrant combining outcome data. If pooling studies is deemed inappropriate, we will qualitatively summarize the available evidence.

If appropriate, a random effects model, following a DerSimonian and Laird approach, will be used to combine data, as clinical and methodological diversity are expected among included studies. Our scoping search of the literature suggests variations in study protocols, including study methods, cointerventions and level of care, and as such a random effects model that assumes treatment effects will follow some distribution across studies is most appropriate.

As appropriate for each outcome outlined in KQ1 through KQ4, we will calculate the following summary measures with 95 percent confidence intervals:

- For continuous outcomes (e.g., gestational age at delivery, birthweight) we will calculate the weighted mean difference.
- For dichotomous outcomes (e.g., neonatal brochopulmonary dysplasia, retinopathy of prematurity, maternal pulmonary edema) we will calculate the pooled odds ratio. For rare dichotomous outcomes (total event rate <1%), results will be combined using the Peto odds ratio method. For rare outcomes, only studies with events recorded will be included in combined estimates. Studies that are not quantitatively synthesized will be qualitatively summarized.
- For the time to event outcome (i.e. time to delivery) we will calculate the pooled hazard ratio.

Heterogeneity in outcomes will be assessed using Cochran’s Q, recognizing this test has low power to detect heterogeneity when the number of studies is relatively small as is expected for this review. For this statistical test a p-value of 0.1 will be used to determine statistical significance and thus the presence of heterogeneity in outcome data. In addition, the I^2^ statistic will be calculated to help quantify the magnitude of heterogeneity, although I^2^ values will not guide the decision to pool outcome data. Reasons for heterogeneity will be explored using subgroup analyses and meta-regression, as described below.

The potential for publication bias will be assessed through the development of a funnel plot, plotting treatment effect against the standard error of the treatment effect.^10^ An asymmetrical funnel plot will suggest a potential for publication bias, which would be considered when providing an interpretation of results.

Exploring heterogeneity

The primary analyses for KQ1 and KQ2 are inherently subgroup analyses to assess observed improvement in neonatal and other outcomes within certain gestational age groups, multiple versus single gestations, women with history of preterm birth, women with history of preeclampsia, and women with RPTL. Within each of these subgroups, we will explore heterogeneity in outcomes in a meta-regression based on the study level covariates of primary tocolytic treatment, terbutaline dose, fetal fibronectin levels, cervical dilation, cervical length, cervical effacement, cervical position, cervical consistency, cervical station, Bishop’s Score, BMI, whether healthcare providers are blinded to the frequency and intensity of maternal contractions at the time of initial presentation with preterm labor, and study risk of bias. A minimum of six studies will be required to conduct a meta-regression.

For KQ3 and KQ4, heterogeneity in maternal and neonatal harms related outcomes will be explored in a meta-regression based on the study level covariates of gestational age, racial subgroups, terbutaline dose, comorbidities and study quality. As for KQ1 and KQ2, a minimum of six studies will be required to proceed with a meta-regression.

The subgroups outlined above have been pre-specified as we expect they will contribute to clinical diversity among the included patient and study populations. Other pre-specified subgroup analyses, specifically related to level of care and level of maternal activity, are discussed below under KQ5. Additional subgroup analyses may be considered posthoc as deemed appropriate.

*Single versus multiple gestations*

In RCTs that include women with multiple gestations, the subject of randomization is pregnant women and not their neonates. Neonatal outcomes will, therefore, be confounded by a clustering effect akin to that in cluster randomized trials since neonates will not have been randomized. For the subgroup of women with multiple gestations, we are aware of this clustering effect on the outcomes of neonates born to the same mother; however, it is likely that clustering effects have not been properly accounted for within included studies.^11^ While analyses that do not account for clustering effects do not lead to biased effect estimates, the standard error of the effect estimate that results from these analyses is too small.^12^ Therefore, if uncorrected, such studies will receive too much weight in combined estimates of treatment effects.

To avoid this, studies that are exclusively of multiple gestations will not be pooled with studies of singletons or studies that include a mixed population of singletons and multiple gestations. However, we will consider pooling studies exclusively of singletons and studies with mixed populations. In studies with mixed populations, the number of women with multiple gestations will be small relative to women with singletons and, therefore, any overweighting that arises by including these studies in pooled estimates is expected to be minimal.

Sensitivity analyses

Sensitivity analyses are planned to explore the robustness of combined estimates. Specifically, we will conduct sensitivity analyses to explore the effect of including or excluding studies at high risk of bias; limiting meta-analyses to randomized trials only; and including studies in meta-analyses from singleton births only versus both singleton births and multiple gestations.

Sensitivity analyses will take the form of subgroup analyses as described above.

**Key question 5**

This question aims to further explore reasons for heterogeneity in outcomes by examining differences in level of care and level of maternal activity between different treatment groups. We will first assign a value of low, moderate or high level of care to each variable outlined in Table 4 for each treatment group within each included study. Similarly, we will assign a value of low, normal or high activity to each treatment group within each included study based on the classification scheme outlined in Table 5 and any other relevant data that is reported. If the level of care, and similarly the level of activity, is deemed similar across treatment groups, an overall assessment will be made for each included study. Alternatively, if the level of care or the level of activity is deemed different across treatment groups an overall assessment will not be possible due to potential confounding within a study. In such cases we will specify both a reason (i.e. based on which variable(s)) and a direction for the confounding.

As the data will allow, for each outcome in KQ1-KQ4 we will conduct a meta-regression based on each level of care and level of activity variable, including an overall global assessment. A minimum of six included studies will be required to proceed with any meta-regression, and studies that do not report sufficient data to make an assessment for any of the variables will be excluded.

Sensitivity analyses

In addition to the sensitivity analyses outlined above, we will conduct sensitivity analyses to explore the effect of including or excluding studies with and without confounding by level of care and level of activity, on combined effect estimates.

Table 4: Criteria to assign value for level of care

|  | **Level of Care** | | |
| --- | --- | --- | --- |
|  | **Low** | **Moderate** | **High** |
| **Nursing assessments** | No nursing assessment made | Patient questioned regarding any barriers to successful compliance of prescribed therapy | An in-person assessment at the patient’s home to identify barriers to successful compliance of prescribed therapy |
| **Home uterine activity monitoring** | No home uterine activity monitoring recommended | Home uterine activity monitoring recommended, with or without a monitor | Home uterine activity monitored and data sent via telephone or computer to a central care centre to be assessed by a trained health professional |
| **Home visits** | No home visits provided | At least one home visit provided | Regular (e.g., weekly) home visits provided |
| **Education about preterm labor** | - No education was provided - Written or oral education on the signs and symptoms of preterm labor, possible adverse reactions to treatment, etc. | - Written and oral education provided on signs and symptoms of preterm labor, possible adverse reactions to treatment, etc. | - Written and oral education provided on signs and symptoms of preterm labor, possible adverse reactions to treatment, etc and education was individualized. |
| **Telephone support** | No telephone support available | Telephone support available during select hours of the day only | Telephone support available 24 hours a day, 7 days a week by trained health professionals |
| **Restriction of maternal activities** | No suggestions made by a health professional regarding restriction of maternal activities | Global recommendations for restriction of maternal activities made for all women | Individualized suggestions for restriction of maternal activities made based on each patient’s condition |

Table 5: Criteria to assign value for level of maternal activity

|  | **Level of Activity** | | |
| --- | --- | --- | --- |
|  | **Low** | **Normal** | **High** |
| **Marital status** | Married or living common-law with partner not working outside the home | Married or living common-law with partner working outside the home | - Single, divorced, widowed or separated |
| **Working status** | Not working | Occasional or part-time work | Full-time work |
| **Caring for other children in the home^1^** | No other children in the home | One other child in the home | More than one other child in the home |
| **Available social support** | Women report substantial support from friends and family | Women report limited support to be available from friends and family | No support available to women from friends and family |
| **Bed rest** | Complete bed rest with bathroom privileges only | Bed rest suggested when an increase in uterine contractions only | Bed rest not recommended |
| **Restriction of maternal activities** | Maternal activities, such as household chores and intercourse, recommended to be completely restricted | Restriction of activities suggested when an increase in uterine contractions only | No restriction of maternal activities recommended |

^1^ If no data is provided regarding children in the home, data on parity (if reported) will be considered a reasonable proxy

**Key question 6**

For each outcome (e.g., missed doses, dislodgement and overdoses) we will pool reported incidence data using a random effects model to calculate the rate of pump failure per unit of time (e.g., weeks of pump use).

G. Grading the Evidence for Each Key Question

The body of evidence for the following important outcomes will be graded, based on the standard EPC approach and as outlined by AHRQ.^13^: incidence of delivery at various gestational ages (<28 weeks, <32 weeks, <34 weeks, <37 weeks); mean prolongation of pregnancy; bronchopulmonary dysplasia; significant intraventricular hemorrhage (grade III/IV); neonatal death and/or death within initial hospitalization; and, maternal withdrawal due to adverse effects (WDAE). For each important outcome, a global assessment of the strength of the evidence (i.e. high, moderate, low, insufficient) will be determined by consensus between two reviewers, based on the following criteria: risk of bias, consistency, directness, and precision.^13^ If no studies are available on an outcome of interest, or the individual study estimates are so inconsistent so as to preclude meaningful conclusions, the evidence will be graded as insufficient.

V. References

# Behrman RE, Stith BA eds. Preterm birth: causes, consequences and prevention. Washington, DC: National Academics Press, 2007.

# Iams JD, Romero R, Creasy RK. Preterm Labour and Birth. In Maternal-Fetal Medicine, Sixth Edition. Saunders Elsevier, Philadelphia PA, 2009. Pages 566-567.

# Guinn DA, Goepfert AR, Owen J, Wenstrom K, Hauth J. Terbutaline pump maintenance therapy for prevention of preterm delivery: a double-blind trial. American Journal of Obstetrics and Gynaecology 1998, Number 4. Pages 874-878.

# Nanda K, Cook LA, Gallo MF, Grimes DA. Terbutaline pump maintenance therapy after threatened preterm labor for preventing preterm birth. Cochrane Database of Systematic Reviews 2002, Issue 4. Art.No.:CD003933. DOI: 10.1002/14651858.CD003933.

# Hayes, Inc. Technology Brief: Continuous subcutaneous terbutaline infusion for the treatment of preterm labor. Published 2006 [updated 2008]. Accessed on Oct 7, 2008.

# Lam F, Elliott J, Jones JS, Katz M, Knuppel RA, Morrison J, Newman R, Phelan J, Willcourt R. Clinical issues surrounding the use of terbutaline sulfate for preterm labor. Obstet Gynecol Surv. 1998 Nov;53(11 Suppl):S85-95.

# Sampson M, McGowan J, Cogo E, Grimshaw J, Moher D, Lefebvre C. An evidence-based practice guideline for the peer review of electronic search strategies. J Clin Epidemiol 2009.

(8) AHRQ Methods Guide, in progress. Chapter 6: Assessing the Quality and Applicability of Included Studies.

# (9) Santaguida P, Raina P, Ismaila A. McMaster Quality Assessment Scale of Harms (McHarm) for primary studies: Manual for use of the McHarm.

# (10) Sterne J, Egger M. Funnel plots for detecting bias in meta-analysis: guidelines on choice of axis. J Clinical Epidemiol. 2004 Oct;54(10):1046-1055.

# (11) Eldridge SM, Ashby D, Feder GS, Rudnicka AR, Ukoumunne OC. Lessons for cluster randomized trials in the twenty-first century: a systematic review of trials in primary care. Clin Trials. 2004 Feb;1(1):80-90.

# (12) Higgins JPT, Deeks JJ, Altman DG (editors). Chapter 16: Special topics in statistics. In: Higgins JPT, Green S (editors), Cochrane Handbook for Systematic Reviews of Interventions Version 5.0.1 (updated September 2008). The Cochrane Collaboration, 2008.

# Available from [**www.cochrane-handbook.org**](http://www.cochrane-handbook.org/).

(13) Owens DK, Lohr KN, Atkins D, Treadwell JR, Reston JT, Bass EB, Chang S, Helfand M. AHRQ Series Paper 5: Grading the strength of a body of evidence when comparing medical interventions – Agency for Healthcare Research and Quality and the Effective Health-Care Program. J Clin Epidemiol 2010;63:513-23.

VI. Definition of Terms

Not applicable

VII. Summary of Protocol Amendments

On October 5, 2010, the following amendments were made to the protocol.

(1) Blinding of subjects, providers, and outcome assessors was removed from the quality assessment for observational studies.

Justification: Observational studies capture routine practice and so blinding generally cannot be implemented in such studies. *Blinding of outcome assessors* (if different from treatment providers) may be possible for occasional outcomes, but in the case of this review, that is unlikely to be true. This, we think, is a question that is unnecessary and not applicable for our primary studies. The increased potential of bias of evidence from observational studies has been acknowledged while rating the strength of evidence (i.e. these studies were started off with high risk of bias).

*(*2) Potential for confounding by indication was removed from the quality assessment for all studies.

Justification: This is essentially the same question as another one we ask while assessing risk of bias, and that question is about “*Similarity of groups at baseline in terms of baseline characteristics and prognostic factors*”. How else would there be confounding by indication were it not because of differences in prognostic factors. Variables that were considered when assessing baseline characteristics and prognostic factors included age, race, socioeconomic status, marital status, smoking status, cervical dilation, cervical effacement, parity, gravidity, cerclage, fetal fibronectin, cervical length, and history of preterm labour (the latter three were considered as important prognostic factors).

| NOTE: The following protocol elements are standard procedures for all protocols.  VIII. Review of Key Questions  For Comparative Effectiveness reviews (CERs) the key questions were posted for public comment and finalized after review of the comments. For other systematic reviews,  key questions submitted by partners are reviewed and refined as needed by the EPC and the Technical Expert Panel (TEP) to assure that the questions are specific and explicit about what information is being reviewed.  IX. Technical Expert Panel (TEP)  A TEP panel is selected to provide broad expertise and perspectives specific to the topic under development. Divergent and conflicted opinions are common and perceived as health scientific discourse that results in a thoughtful, relevant systematic review. Therefore study questions, design and/or methodological approaches do not necessarily represent the views of individual technical and content experts. The TEP provides information to the EPC to identify literature search strategies, review the draft report and recommend approaches to specific issues as requested by the EPC. The TEP does not do analysis of any kind nor contribute to the writing of the report.  X. Peer Review  Approximately five experts in the field will be asked to peer review the draft report and provide comments. The peer reviewer may represent stakeholder groups such as professional or advocacy organizations with knowledge of the topic. On some specific reports such as reports requested by the Office of Medical Applications of Research, National Institutes of Health there may be other rules that apply regarding participation in the peer review process. Peer review comments on the preliminary draft of the report are considered by the EPC in preparation of the final draft of the report. The synthesis of the scientific literature presented in the final report does not necessarily represent the views of individual reviewers. The dispositions of the peer review comments are documented and will, for CERs and Technical briefs, be published three months after the publication of the Evidence report.  It is our policy not to release the names of the Peer reviewers or TEP panel members until the report is published so that they can maintain their objectivity during the review process. |
| --- |

Appendix 1: MEDLINE search strategy

| 1. | exp Obstetric Labor, Premature/ |
| --- | --- |
| 2. | (PTL or PTB or RPTL).ti,ab. |
| 3. | ((premature* or pre-mature* or preterm or pre-term or early) adj5 (labor* or labour* or birth* or deliver*)).ti,ab. |
| 4. | ((premature* or pre-mature* or preterm or pre-term or early) adj5 ((uterine or uterus) adj2 contract*)).ti,ab. |
| 5. | Tocolysis/ or Tocolytic Agents/ |
| 6. | (tocolysis or tocolytic*).ti,ab. |
| 7. | 1 or 2 or 3 or 4 or 5 or 6 |
| 8. | exp Terbutaline/ |
| 9. | (Terbutalin* or Brethaire or Brethine or Bricanyl or "BRN 2370513" or "EINECS 245-385-8" or "UNII-N8ONU3L3PG").ti,ab. |
| 10. | (23031 25 6 terbutaline or 23031 32 5 terbutaline sulfate).rn. |
| 11. | 8 or 9 or 10 |
| 12. | exp Injections, Subcutaneous/ |
| 13. | exp Infusion Pumps/ |
| 14. | exp Home Infusion Therapy/ |
| 15. | exp Infusions, Parenteral/ |
| 16. | (subcutaneous* or SubQ or sub-cutaneous* or pump or pumps or infuse or infused or infuses or infusing or infusion* or infuser*).ti,ab. |
| 17. | ((home adj3 therapy) or (home adj3 therapies) or (home adj3 tocoyl*) or (home-based adj3 therapy) or (home-based adj3 therapies) or (home-based adj3 tocoyl*)).ti,ab. |
| 18. | ((maintenance adj3 therapy) or (maintenance adj3 therapies) or (maintenance adj3 therapeutic) or (maintenance adj3 treatment*) or (maintenance adj3 tocoly*) or (supportive adj3 therapy) or (supportive adj3 therapies) or (supportive adj3 treatment*) or (supportive adj3 tocoyls*) or (outpatient adj3 therapy) or (outpatient adj3 therapies) or (outpatient* adj3 treatment*) or (outpatient* adj3 tocoly*)).ti,ab. |
| 19. | ((long-term adj therapy) or (long-term adj therapies) or (long-term adj therapeutic) or (long-term adj treatment*) or (long-term adj management) or (long-term adj tocoly*) or (longterm adj therapy) or (longterm adj therapies) or (longterm adj therapeutic) or (longterm adj treatment*) or (longterm adj management) or (longterm adj tocoly*)).ti,ab. |
| 20. | 12 or 13 or 14 or 15 or 16 or 17 or 18 or 19 |
| 21. | 11 and 20 |
| 22. | 7 and 21 |

1. Also see: Witter FR, Zimmerman AW, Reichmann JP, Connors SL. In utero beta 2 adrenergic agonist exposure and adverse neurophysiologic and behavioral outcomes. Am J Obstet Gynecol 2009 Dec;553-9. [↑](#footnote-ref-2)
2. Hayes E, Moroz L, Pizzi L, Baxter J. A cost decision analysis of 4 tocolytic drugs. Am J Obstet Gynecol. Oct 2007;197(4):383;e1-6. [↑](#footnote-ref-3)
