# Supplementary material for: Effectiveness of Terbutaline Pump for the Prevention of Preterm Birth. A Systematic Review and Meta-Analysis
Source: PLoS One. 2012 Feb 21;7(2):e31679. doi: 10.1371/journal.pone.0031679 (PMC3283660; doi:10.1371/journal.pone.0031679)
Supplement: Information S2 — Full Evidence Report. (PDF) [file pone.0031679.s002.pdf]

Effective Health Care Program

Comparative Effectiveness Review
Number 35

Terbutaline Pump for the Prevention of Preterm Birth

DEPARTMENT OF HEALTH & HUMAN SERVICES • USA

AHRQ
Agency for Healthcare Research and Quality
Advancing Excellence in Health Care • www.ahrq.gov

Comparative Effectiveness Review
Number 35

Terbutaline Pump for the Prevention of Preterm Birth

Prepared for:
Agency for Healthcare Research and Quality
U.S. Department of Health and Human Services
540 Gaither Road
Rockville, MD 20850
www.ahrq.gov

Contract No. HHSA 290 2007 10059 I

Prepared by:
University of Ottawa Evidence-based Practice Center
Ottawa, Ontario, Canada

EPC Director:
David Moher, Ph.D.

Investigators:
Laura Gaudet, M.D.
Kavita Singh, M.P.H.
Laura Weeks, Ph.D.
Becky Skidmore, M.L.S.
Sophia Tsouros, B.H. Kin.
Alexander Tsertsvadze, M.D., M.Sc.
Raymond Daniel, B.A.
Steve Doucette, M.Sc.
Mark Walker, M.D.
Mohammed Ansari, M.B.B.S., M.Med.Sc., M.Phil.

AHRQ Publication No. 11-EHC068-EF
September 2011

This report is based on research conducted by the University of Ottawa Evidence-based Practice Center under contract to the Agency for Healthcare Research and Quality (AHRQ), Rockville, MD (Contract No. 290-2007-10059-I). The findings and conclusions in this document are those of the author(s), who are responsible for its content, and do not necessarily represent the views of AHRQ. No statement in this report should be construed as an official position of AHRQ or of the U.S. Department of Health and Human Services.

The information in this report is intended to help clinicians, employers, policymakers, and others make informed decisions about the provision of health care services. This report is intended as a reference and not as a substitute for clinical judgment.

This report may be used, in whole or in part, as the basis for the development of clinical practice guidelines and other quality enhancement tools, or as a basis for reimbursement and coverage policies. AHRQ or U.S. Department of Health and Human Services endorsement of such derivative products or actions may not be stated or implied.

This document is in the public domain and may be used and reprinted without permission except those copyrighted materials noted for which further reproduction is prohibited without the specific permission of copyright holders.

None of the investigators have any affiliations or financial involvement that conflicts with the material presented in this report.

Suggested citation: Gaudet L, Singh K, Weeks L, Skidmore B, Tsouros S, Tsertsvadze A, Daniel R, Doucette S, Walker M, Ansari MT. Terbutaline Pump for the Prevention of Preterm Birth. Comparative Effectiveness Review No. 35. (Prepared by the University of Ottawa Evidence-based Practice Center under Contract No. HHSA290-2007-10059-I.) AHRQ Publication No. 11-EHC068-EF. Rockville, MD: Agency for Healthcare Research and Quality. September 2011. Available at: www.effectivehealthcare.ahrq.gov/reports/final.cfm.

Preface

The Agency for Healthcare Research and Quality (AHRQ) conducts the Effective Health Care Program as part of its mission to organize knowledge and make it available to inform decisions about health care. As part of the Medicare Prescription Drug, Improvement, and Modernization Act of 2003, Congress directed AHRQ to conduct and support research on the comparative outcomes, clinical effectiveness, and appropriateness of pharmaceuticals, devices, and health care services to meet the needs of Medicare, Medicaid, and the Children's Health Insurance Program (CHIP).

AHRQ has an established network of Evidence-based Practice Centers (EPCs) that produce Evidence Reports/Technology Assessments to assist public- and private-sector organizations in their efforts to improve the quality of health care. The EPCs now lend their expertise to the Effective Health Care Program by conducting comparative effectiveness reviews (CERs) of medications, devices, and other relevant interventions, including strategies for how these items and services can best be organized, managed, and delivered.

Systematic reviews are the building blocks underlying evidence-based practice; they focus attention on the strength and limits of evidence from research studies about the effectiveness and safety of a clinical intervention. In the context of developing recommendations for practice, systematic reviews are useful because they define the strengths and limits of the evidence, clarifying whether assertions about the value of the intervention are based on strong evidence from clinical studies. For more information about systematic reviews, see http://www.effectivehealthcare.ahrq.gov/reference/purpose.cfm.

AHRQ expects that CERs will be helpful to health plans, providers, purchasers, government programs, and the health care system as a whole. In addition, AHRQ is committed to presenting information in different formats so that consumers who make decisions about their own and their family's health can benefit from the evidence.

Transparency and stakeholder input from are essential to the Effective Health Care Program. Please visit the Web site (http://www.effectivehealthcare.ahrq.gov) to see draft research questions and reports or to join an e-mail list to learn about new program products and opportunities for input. Comparative Effectiveness Reviews will be updated regularly.

We welcome comments on this CER. They may be sent by mail to the Task Order Officer named below at: Agency for Healthcare Research and Quality, 540 Gaither Road, Rockville, MD 20850, or by e-mail to epc@ahrq.hhs.gov.

Carolyn M. Clancy, M.D.
Director
Agency for Healthcare Research and Quality

Jean Slutsky, P.A., M.S.P.H.
Director, Center for Outcomes and Evidence
Agency for Healthcare Research and Quality

Stephanie Chang, M.D., M.P.H.
Task Order Officer, Director
Evidence-based Practice Program
Center for Outcomes and Evidence
Agency for Healthcare Research and Quality

Acknowledgments

The authors gratefully acknowledge the following individuals for their contributions to this project: Jessica Dy, M.D. and Elizabeth Darling R.M., M.Sc. for their intellectual contributions to the topic refinement document of the review.

Key Informants

Alan Fleischman, M.D.
March of Dimes Foundation
White Plains, NY

Elizabeth Hill-Karbowski, C.N.M., M.S.N.
St. Joseph's Hospital
Milwaukee, WI

Paul Keenan, M.D.
Oklahoma Health Care Authority
Oklahoma City, OK

Sylvia Lopez, M.D.
Oklahoma Health Care Authority
Oklahoma City, OK

Nancy Jo Reedy, R.N., C.N.M., M.P.H.
Andrews Midwife Center
Fort Worth, TX

Technical Expert Panel

Jeff Andrews, M.D., FRCSC, FACOG
Associate Professor
Vanderbilt University Medical Center
Nashville, TN

Jeanne-Marie Guise, M.D., M.P.H.
Department of Obstetrics and Gynecology
Oregon Health and Science University
Portland, OR

Sylvia Lopez, M.D.
Oklahoma Health Care Authority
Oklahoma City, OK

James Reichmann, M.B.A.
American Homepatient Inc.
Brentwood, TN

Fung Lam, M.D.
Comprehensive Center for Pelvic Medicine
California-Pacific Medical Center
San Francisco, California

Peer Reviewers

Debra Guinn, M.D.
Department of Obstetrics and Gynecology
Northwest Perinatal Centre
Portland, OR

John Morrison, M.D.
Department of Obstetrics and Gynecology
University of Mississippi Medical Centre
Jackson, MS

Hyagriv Simhan, M.D., M.S.C.R.
Department of Obstetrics, Gynecology & Reproductive Sciences
University of Pittsburgh
Pittsburgh, PA

Charles Lockwood, M.D., M.H.C.M.
Department of Obstetrics, Gynecology & Reproductive Sciences
Yale University
New Haven, CT

Katherine Wenstrom, M.D.
Department of Obstetrics and Gynecology
Brown University
Providence, RI

Brian Mercer, M.D.
Department of Reproductive Biology
Case Western Reserve University
Cleveland, OH

Terbutaline Pump for the Prevention of Preterm Birth

Structured Abstract

Background. Tocolytic agents inhibit contractions during the labor process. Subcutaneous terbutaline (SQ terbutaline) infusion by pump is used as a prolonged (beyond 48–72 hours) maintenance tocolytic following acute treatment of preterm contractions. The effectiveness and safety of this maintenance tocolysis have not been clearly established.

Objectives. To compare the benefits and harms of the SQ terbutaline pump with other tocolytics, conservative management, or placebo in specific populations of women with arrested preterm labor, and to explore confounding by level of maternal activity and care.

Data Sources. MEDLINE (1950 to April 1, 2011); Embase (1980 to April 1, 2011); CINAHL (1985 to December 7, 2009), the Cochrane Library (April 1, 2011), and the Centre for Reviews and Dissemination databases (January 2, 2010). We also reviewed grey literature.

Review Methods. We followed a prior systematic review protocol. Two reviewers independently included reports that investigated SQ terbutaline pump therapy in women between 24–36 weeks' gestation following arrest of preterm labor. We included noncomparative studies only for pump-related harms. Non-English records without an English abstract were excluded. We also excluded case reports but sought Food and Drug Administration (FDA) summaries of postmarketing surveillance data. One reviewer extracted data into a standardized electronic form and assessed study risk of bias and applicability. A second reviewer verified data.

Results. Two randomized trials, one nonrandomized trial, and 11 observational studies met inclusion criteria. In women with recurrent preterm labor (RPTL) and singleton gestation, the strength of evidence favoring the SQ terbutaline pump over oral tocolytics or no treatment is low for the outcomes of incidence of delivery at <32 weeks and <37 weeks, and mean days of pregnancy prolongation. In women with RPTL and twin gestation, the strength of evidence favoring the pump over oral tocolytics is low for neonatal death, incidence of delivery at <32 weeks, and mean days of pregnancy prolongation. Strength of evidence is insufficient for bronchopulmonary dysplasia, incidence of delivery <28 weeks and <34 weeks, and withdrawals due to adverse events. Observational studies of medium to high risk of bias showed the benefit of the SQ terbutaline pump for other surrogate outcomes, such as birth weight and neonatal intensive care unit (NICU) admission. Absent or inconclusive evidence addressed all other neonatal health outcomes, neonatal harms, maternal harms, and pump-related outcomes. An assessment of confounding by maternal activity and maternal care was not possible due to sparse data. Until 2009, several cases of maternal deaths and maternal cardiovascular events in association with terbutaline tocolysis have been reported to the FDA.

Conclusions. The evidence base consists of a small number of biased studies. A substantial body of evidence originated from one proprietary database. Although evidence suggests that pump therapy is beneficial as maintenance tocolysis, our confidence in its validity and reproducibility is low. While postmarketing surveillance has detected cases of serious harms, safety of the therapy remains unclear.

Contents

Executive Summary ES-1
Introduction 1
Conceptual Framework and Key Questions 3
Methods 5
Topic Development and Refinement 5
Search Strategy 7
Study Selection 7
Data Extraction 9
Risk of Bias Assessment 9
Grading the Body of Evidence 11
Applicability 11
Data Synthesis and Analysis 11
Results 13
Literature Search 13
General Study Characteristics 14
Population 15
Intervention 16
Double-Counting of Outcomes Data 16
Risk of Bias Assessment 17
Cohort Studies (n=9) 17
Case Series (n=2) 18
Nonrandomized Comparative Trials (n=1) 19
RCTs (n=3, Randomized Arms) 21
Sources of Funding 22
Key Question 1. Neonatal Health Outcomes 22
Key Points 23
Detailed Analysis 23
Key Question 2. Other Surrogate Outcomes 29
Key Points 29
Detailed Analysis 32
Key Question 3. Maternal Harms 58
Key Points 58
Detailed Analysis 59
Key Question 4. Neonatal Harms 61
Key Points 61
Detailed Analysis 61
Key Question 5. Level of Activity and Level of Care 62
Key Points 62
Detailed Analysis 62
Evidence Synthesis 63
Key Question 6. Incidence of Pump Failure 63
Key Points 63
Detailed Analysis 64

Overall Applicability for Body of Evidence 65
Discussion 68
Limitations of Included Studies 68
Summary of Key Findings 69
Key Question 1. Neonatal Health Outcomes 69
Key Question 2. Other Surrogate Outcomes 69
Key Question 3. Maternal Harms 70
Key Question 4. Neonatal Harms 71
Key Question 5. Level of Activity and Level of Care 71
Key Question 6. Incidence of Pump Failure 71
Conclusions 71
Comparison of Results With Other Systematic Reviews 72
Applicability 73
Future Research 73
Randomized Trials 74
Observational Studies 74
Implications 75
References 76
Acronyms and Abbreviations 79
Glossary 80

Tables

Table A. Summary Characteristics of the Included Studies. ES-8
Table B. SQ Terbutaline Pump Versus Comparator: Strength of Evidence for Populations of Interest. ES-10
Table C. Overall Applicability of the Body of Evidence ES-14
Table 1. Overall Risk of Bias Ratings 10
Table 2. Maternal Characteristics. 15
Table 3. Intervention Characteristics. 16
Table 4. Sources of Funding. 22
Table 5. Summary Table for Neonatal Death. 24
Table 6. Neonatal Death – Strength of Evidence for Populations of Interest. 25
Table 7. Neonatal Death – Strength of Evidence for Nonspecific Preterm Labor Populations 25
Table 8. Summary Table for Significant Intraventricular Hemorrhage (Grade III/IV). 26
Table 9. Significant Intraventricular Hemorrhage (Grade III/IV) - Strength of Evidence for Populations of Interest. 27
Table 10. Significant Intraventricular Hemorrhage (Grade III/IV) - Strength of Evidence for Nonspecific Preterm Labor Populations. 27
Table 11. Summary Table for Stillbirth. 28
Table 12. Summary Table for Mean Gestational Age at Delivery (Subgroup: Multiple Gestation). 33
Table 13. Summary Table for Mean Gestational Age at Delivery (Subgroup: RPTL). 34
Table 14. Summary Table for Mean Gestational Age at Delivery (Overall Evidence). 35
Table 15. Summary Table for Incidence of Delivery < 32 Weeks' Gestation (Subgroup: Multiple Gestation). 37

Table 16. Summary Table for Incidence of Delivery < 32 Weeks' Gestation (Subgroup: RPTL). 38
Table 17. Incidence of Delivery < 32 Weeks' Gestation – Strength of Evidence for Populations of Interest. 39
Table 18. Incidence of Delivery < 34 Weeks' Gestation – Strength of Evidence for Nonspecific Preterm Labor Populations. 39
Table 19. Summary Table for Incidence of Delivery < 37 Weeks' Gestation (Subgroup: RPTL). 40
Table 20. Incidence of Delivery < 37 Weeks' Gestation – Strength of Evidence for Populations of Interest. 42
Table 21. Incidence of Delivery < 37 Weeks' Gestation – Strength of Evidence for Nonspecific Preterm Labor Populations. 42
Table 22: Summary Table for Mean Prolongation of Pregnancy (Subgroup: RPTL). 44
Table 23. Summary Table for Mean Prolongation of Pregnancy (Overall Evidence). 44
Table 24. Mean Prolongation of Pregnancy – Strength of Evidence for Populations of Interest. 46
Table 25. Mean Prolongation of Pregnancy – Strength of Evidence for Nonspecific Preterm Labor Populations. 46
Table 26. Summary Table for Pregnancy Prolongation > 7 Days (Overall Evidence). 47
Table 27. Summary Table for Pregnancy Prolongation > 14 Days (Subgroup: Multiple Gestation). 48
Table 28. Summary Table for Pregnancy Prolongation > 14 Days (Subgroup: RPTL). 48
Table 29. Summary Table for Mean Birthweight (Subgroup: Multiple Gestation). 49
Table 30. Summary Table for Mean Birthweight (Subgroup: RPTL). 50
Table 31. Summary Table for Mean Birthweight (Overall Evidence). 51
Table 32. Summary Table for Incidence of Low Birthweight (Subgroup: Multiple Gestation). 52
Table 33. Summary Table for Incidence of Low Birthweight (Subgroup: RPTL). 53
Table 34. Summary Table for Incidence of Very Low Birthweight (Subgroup: Multiple Gestation). 54
Table 35. Summary Table for Incidence of Very Low Birthweight (Subgroup: RPTL). 54
Table 36. Summary Table for Mean Pregnancy Prolongation Index (Subgroup: RPTL). 55
Table 37. Summary Table for Incidence of NICU Admission (Subgroup: Multiple Gestation). 56
Table 38. Summary Table for Incidence of NICU Admission (Subgroup: RPTL). 57
Table 39. Summary Table for NICU Length of Stay (Subgroup: RPTL). 58
Table 40. Summary Table for Maternal Hyperglycemia. 60
Table 41. Summary Table for Mortality. 60
Table 42. Summary Table for Pulmonary Edema. 60
Table 43. Summary Table for Maternal Discontinuation of Therapy. 61
Table 44. Studies That Could be Rated for Level of Maternal Care. 63
Table 45. Applicability Assessment by the PICOTS Domains. 65

Figures

Figure A. Analytical Framework of Terbutaline Pump for Maintenance Tocolysis ES-4
Figure B. PRISMA Diagram. ES-7

Figure 1. Analytical Framework of Terbutaline Pump for Maintenance Tocolysis 6
Figure 2. PRISMA Diagram 14
Figure 3. Risk of Bias Assessment for Cohort Studies 18
Figure 4. Risk of Bias Assessment for Case Series (n=2) 19
Figure 5. Risk of Bias Assessment for Nonrandomized Trials (n=1) 20
Figure 6. Risk of Bias Assessment for RCTs (n=3, Randomized Arms) 21
Figure 7. Mean Gestational Age (in Weeks) at Delivery (RCT Pooled Estimate: I^2 = 0.0 Percent, p-Value > 0.05) 36
Figure 8. Incidence of Delivery < 32 Weeks' Gestation 38
Figure 9. Incidence of Delivery < 37 Weeks' Gestation 41
Figure 10. Mean Prolongation of Pregnancy (RCT Pooled Estimate: I^2 = 0.0 Percent, p-Value > 0.05) 45
Figure 11. Pregnancy Prolongation > 14 Days 49
Figure 12. Mean Birthweight (RCT Pooled Estimate: I^2 = 0.0 Percent, p-Value > 0.05) 52
Figure 13. Incidence of Low Birth Weight 53
Figure 14. Incidence of Very Low Birth Weight 55
Figure 15. NICU Admission Incidence 57
Figure 16. NICU Length of Stay 58

Appendixes

Appendix A. Search Strategies
Appendix B. Grey Literature Search
Appendix C. Scientific Information Packet Request
Appendix D. Screening, Data Extraction, Risk of Bias, and Applicability Forms
Appendix E. Excluded Studies
Appendix F. Evidence Tables
Appendix G. Modifications to Key Questions

Executive Summary

Background

Preterm birth is defined as delivery before the completion of the 37th week of gestation, and it affects 13 percent of live births in the United States.[1] According to the 2010 National Vital Statistics report, there were 542,893 preterm births in the United States in 2006.[2] Rates of preterm birth result in a significant disease burden to the health care system. Although overall rates of neonatal mortality continue to decline, infants born too early are at risk for long-term morbidity.[3]

Tocolytics are drugs used to delay or inhibit contractions during the labor process. Several tocolytics are available to prevent preterm birth. These agents may be administered as primary therapy to control acute episodes of preterm labor or as maintenance therapy to prevent subsequent episodes. Maintenance tocolysis is usually provided for prolonged periods beyond 48 to 72 hours after arrest of acute preterm labor to inhibit the process of parturition until full term. While several studies have examined these agents for the control of acute episodes of preterm labor, the evidence to support their safety and efficacy as maintenance therapy is limited.

The β-agonist agent, terbutaline sulfate, has been used orally and subcutaneously as maintenance tocolytic therapy in women following acute treatment and arrest of confirmed preterm labor. As with all other contemporary tocolytics, the use of terbutaline for maintenance tocolysis is off-label. The Food and Drug Administration (FDA) has approved terbutaline for the management of acute and chronic obstructive pulmonary disease only. When administered through the subcutaneous (SQ) route, terbutaline may be administered by a pump that provides a steady continuous infusion with allowance for boluses. Compared with the oral route of administration, the SQ terbutaline pump uses lower doses (usual basal rate is 0.03–0.05 mg/hr with an intermittent bolus of 0.25 mg every 4 to 6 hours) and has less potential for tachyphylaxis.[4]

The effectiveness and safety of the SQ terbutaline pump for maintenance tocolytic therapy was examined in two systematic reviews. One review, which was based on two small randomized controlled trials (RCTs), concluded that the SQ terbutaline pump offers no advantages compared with the saline pump or oral terbutaline.[4] The second review found contradictory results among RCTs and observational studies; the RCTs found no difference between the SQ terbutaline pump and comparators, although the observational studies demonstrated positive effect estimates in favor of the pump.[5]

Despite previous systematic reviews, uncertainty surrounding the use of terbutaline and other tocolytics as maintenance therapy to prevent recurrent episodes of preterm labor still exists. No clear first-line maintenance tocolytic therapy has yet emerged. The possibility of maternal side effects and unclear evidence on perinatal outcomes contribute to the ambiguity of terbutaline's role in obstetrical practice. Moreover, in a recent cost analysis of four tocolytic agents, subcutaneous terbutaline had the highest cost.[6] The expense is due not only to the device, but also to the need for increased monitoring and management of adverse events associated with this therapy.[6]

Given the importance and associated uncertainty about the appropriateness of ongoing use of the terbutaline pump for maintenance tocolysis for clinicians, patients, and policymakers, a review about the effectiveness and safety of SQ terbutaline pump was commissioned by the Agency for Healthcare Research and Quality (AHRQ) to address six Key Questions. This

evidence report will add to previous systematic reviews by performing an up-to-date search of the literature, synthesizing evidence in the context of specific populations of women, addressing confounding by level of maternal activity and level of care, and grading the strength of evidence for important outcomes to help decisionmakers develop evidence-based recommendations and policies.

Objectives

The objectives of this review were to examine the efficacy, effectiveness, and safety of the SQ terbutaline pump as prolonged maintenance tocolysis for inhibiting progression of parturition in women with arrested acute preterm labor. The SQ terbutaline pump was compared with placebo, conservative treatment, or any other active intervention in the following specific populations: women delivering at various gestational ages, classified as extremely preterm (<28 weeks of gestation), very preterm (28 weeks to 31 weeks of gestation), preterm (32 weeks to 33 weeks of gestation), and later preterm (34 weeks to 36 weeks of gestation); women with multiple gestation; women of different racial or ethnic backgrounds; women with previous preterm birth; women with history of preeclampsia; and women with recurrent preterm labor (RPTL) during the same pregnancy. Clinical endpoints, which included neonatal health outcomes and maternal/neonatal harms, were assessed in addition to several surrogate outcomes, such as birth weight and prolongation of pregnancy. The potential confounding effects of maternal activity and maternal care on the above endpoints were explored. Lastly, the pump device was evaluated by examining the incidence of pump-related outcomes, such as missed doses, dislodgment, and overdose.

These objectives were framed in the following Key Questions:

In women with arrested preterm labor, does treatment with an SQ infusion of terbutaline delivered by a pump, in comparison with placebo, conservative treatment, or other interventions:

Key Question 1: improve neonatal health outcomes, including bronchopulmonary dysplasia, neonatal death, death within initial hospitalization, significant intraventricular hemorrhage (grade III/IV), necrotizing enterocolitis, periventricular leukomalacia, retinopathy of prematurity, seizures, sepsis, and stillbirth for the following subgroups:

a. Women <28 weeks of gestation (extremely preterm)?
b. Women between 28 weeks and 31 weeks of gestation (very preterm)?
c. Women between 32 weeks and 33 weeks of gestation (preterm)?
d. Women between 34 weeks and 36 weeks of gestation (later preterm)?
e. Multiple gestation?
f. Racial or ethnic subgroups?
g. Women with previous preterm birth?
h. Women with history of preeclampsia?
i. Women with RPTL and women without RPTL?

Key Question 2: improve other surrogate outcomes, including gestational age at delivery, incidence of delivery at various gestational ages (<28 weeks, < 32 weeks, <34 weeks, <37 weeks), mean prolongation of pregnancy (days), birth weight, ratio of birth weight/gestational age at delivery, pregnancy prolongation index, need for assisted ventilation, need for oxygen per nasal cannula, and neonatal intensive care unit (NICU) admission for the following subgroups:

a. Women <28 weeks of gestation (extremely preterm)?
b. Women between 28 weeks and 31 weeks of gestation (very preterm)?
c. Women between 32 weeks and 33 weeks of gestation (preterm)?
d. Women between 34 weeks and 36 weeks of gestation (later preterm)?
e. Multiple gestation?
f. Racial or ethnic subgroups?
g. Women with previous preterm birth?
h. Women with history of preeclampsia?
i. Women with RPTL and women without RPTL?

Key Question 3: increase the maternal harms of arrhythmia, heart failure, hyperglycemia, hypokalemia, maternal mortality, myocardial infarction, pulmonary edema, or refractory hypotension, or result in an increased rate of maternal discontinuation of therapy or maternal withdrawal due to adverse effects (Withdrawal-AE)?

Key Question 4: increase the neonatal terbutaline-related harms of hypoglycemia, hypocalcemia, and ileus?

Key Question 5: Can the differences in the outcomes above be partially explained by the differences in level of care (e.g., frequency of followup, nurse visits, concomitant treatment, etc.) and level of activity (e.g., other children in the home, marital/support status, working status, bedrest, etc.) between the terbutaline pump group and the comparator group?

Key Question 6: What is the incidence of failure of the pump device used for terbutaline infusion, including missed doses, dislodgment, and overdose?

Analytic Framework

We developed an analytic framework depicting links between the intervention and related clinical and intermediate efficacy and harms outcomes and other unintended adverse effects (Figure A). In the framework below, the key questions of interest can be seen to encompass a holistic inquiry of the topic.

Figure A. Analytical framework of terbutaline pump for maintenance tocolysis

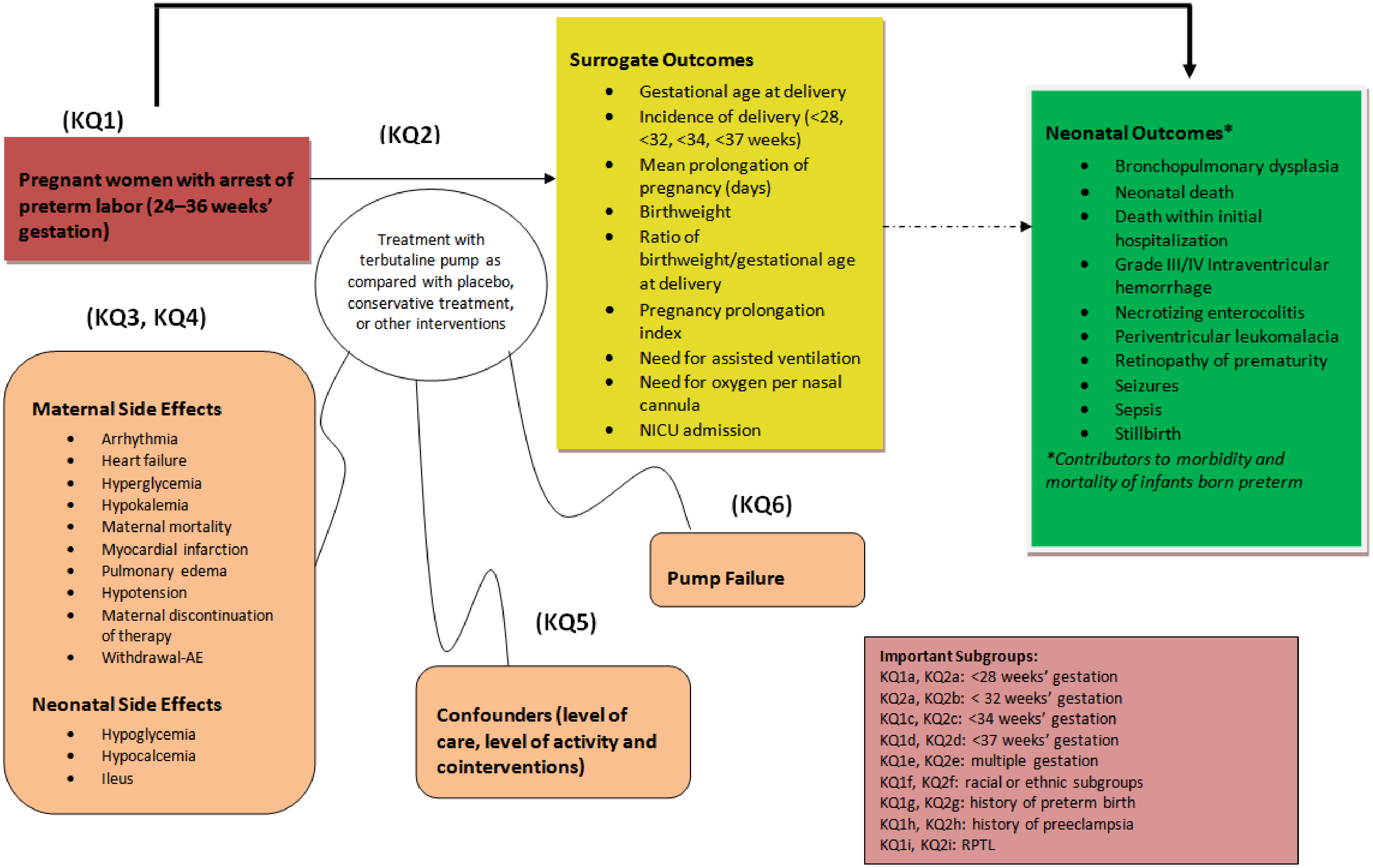

Methods

Input From Stakeholders

We formulated the population, intervention, comparator, outcome, timing, setting (PICOTS) conceptual framework and Key Questions in consultation with key informants during a topic refinement stage. The public was invited to provide comments on the Key Questions. During the review process, we followed a research protocol we developed with the clinical and methodological input of a technical expert panel. The protocol followed the Effective Health Care Program's Methods Guide for Effectiveness and Comparative Effectiveness Reviews.[7]

Data Sources and Searches

We developed a peer-reviewed search strategy and searched the following databases: MEDLINE In-Process & Other Non-Indexed Citations and MEDLINE (1950 to April 1, 2011); Embase (1980 to April 1, 2011); Cumulative Index to Nursing and Allied Health Literature (CINAHL) via EBSCOhost (1985 to December 7, 2009), the Cochrane Library via the Wiley interface (April 1, 2011) (including CENTRAL, Cochrane Database of Systematic Reviews, Database of Abstracts of Reviews of Effects – DARE, Health Technology Assessment – HTA, and the National Health Service Economic Evaluation Database – NHS EED), and the Centre for Reviews and Dissemination (CRD) databases (January 2, 2010). Appendix A provides details of the search strategies. We hand-searched the bibliographies and text of review articles, letters to editors, and commentaries and the reference lists of included studies for additional references. We also reviewed grey literature sources and information received from pharmaceutical companies (see Appendixes B and C), and sought unpublished information from Matria (now called Alere) Healthcare about their perinatal program and associated database.

In February 2011, the FDA issued new warnings against the use of terbutaline to treat preterm labor, so we also accessed a summary of the FDA postmarketing surveillance results. This decision was made post hoc.

Study Selection

Two reviewers screened abstracts and full-text reports with conflicts resolved by consensus or third-party adjudication. Studies were included if they met the following criteria: evaluated pregnant women between 24 and 36 weeks' gestation having had acute preterm labor arrested with primary tocolytic therapy; contained at least one group that was administered the SQ terbutaline pump; and assessed one of the specified outcomes listed in the key questions or described a long-term childhood outcome. Noncomparative studies (i.e., case series) were assessed only for pump-related harms outcomes, such as incidence of pump failure, missed doses, or overdose. Non-English records without an English abstract were excluded. We also excluded case reports, but in a post hoc decision sought FDA summaries of postmarketing data highlighting serious harms.

Data Extraction and Risk of Bias Assessment

One reviewer extracted data into a standardized electronic form and assessed study risk of bias and applicability. Extraction items included general study characteristics (e.g., year of publication, study design), population characteristics (e.g., inclusion/exclusion criteria, age, race,

level of activity), intervention characteristics (e.g., dose, duration, details about comparators, level of care), and outcomes with their estimates. A second reviewer verified outcomes data and study risk of bias assessments. Ratings for level of activity, level of care, and assessments of applicability were verified by a clinical expert. Level of activity and level of care were rated based on composite assessments across preidentified variables.

We assessed study risk of bias given the study design, by outcome, using generic items to assess confounding and various types of bias (e.g., selection, performance, detection bias, attrition bias). Selected items from the McMaster Quality Assessment Scale of Harms were also incorporated into the risk of bias assessment for harm-related outcomes.[8] Certain criteria were specific to particular study designs (e.g., allocation generation and concealment applied only to RCTs). We rated each relevant outcome in a study with an overall risk of bias rating designated as high, medium, or low. Outcomes were rated as high risk of bias if there was an apparent and major flaw in the study that would invalidate results.

Appendix D provides the data extraction, risk of bias, and applicability forms.

Data Synthesis and Analysis

We meta-analyzed the RCTs with a random effects model, following a DerSimonian and Laird approach, when they were clinically and methodologically similar. To assess statistical heterogeneity and the magnitude of heterogeneity, we used Cochran's Q (α=0.10) and the I^2 statistic respectively. Odds ratios (ORs) were calculated for dichotomous outcomes and mean differences for continuous outcomes. All analyses were performed using Comprehensive Meta Analysis version 2.2.046 or version 2.2.055 (New Jersey, USA). We did not meta-analyze observational studies because of potential differences in confounders, nor did we combine studies of singleton and multiple pregnancies. Synthesis of evidence from observational studies was, therefore, undertaken qualitatively. Due to the small number of studies, we could not perform any meta-regression to explore statistical heterogeneity in effect estimates.

Strength of Evidence and Applicability

Based on published guidance for the Effective Health Care Program,[9] two reviewers graded the strength of evidence using the four primary domains (i.e., risk of bias, consistency, directness, and precision) for the following outcomes: incidence of delivery at various gestational ages (<28 weeks, <32 weeks, <34 weeks, <37 weeks), mean prolongation of pregnancy, bronchopulmonary dysplasia, significant intraventricular hemorrhage (grade III/IV), neonatal death, death within initial hospitalization, and maternal withdrawal due to adverse effects (Withdrawal-AE). We described population, intervention, comparison, outcome, timing, and setting characteristics to summarize the applicability of the body of evidence.

Results

Study Selection

We screened 427 citations and included 14 unique records in the review. The PRISMA (Preferred Reporting Items for Systematic Reviews and Meta-Analyses) diagram below depicts the flow of records from identification to inclusion (Figure B). Most records were excluded at full-text screening (n=197) based on the reasons listed in the diagram. Appendix E provides a list of excluded studies, and Appendix F provides individual-level study data.

Figure B. PRISMA diagram

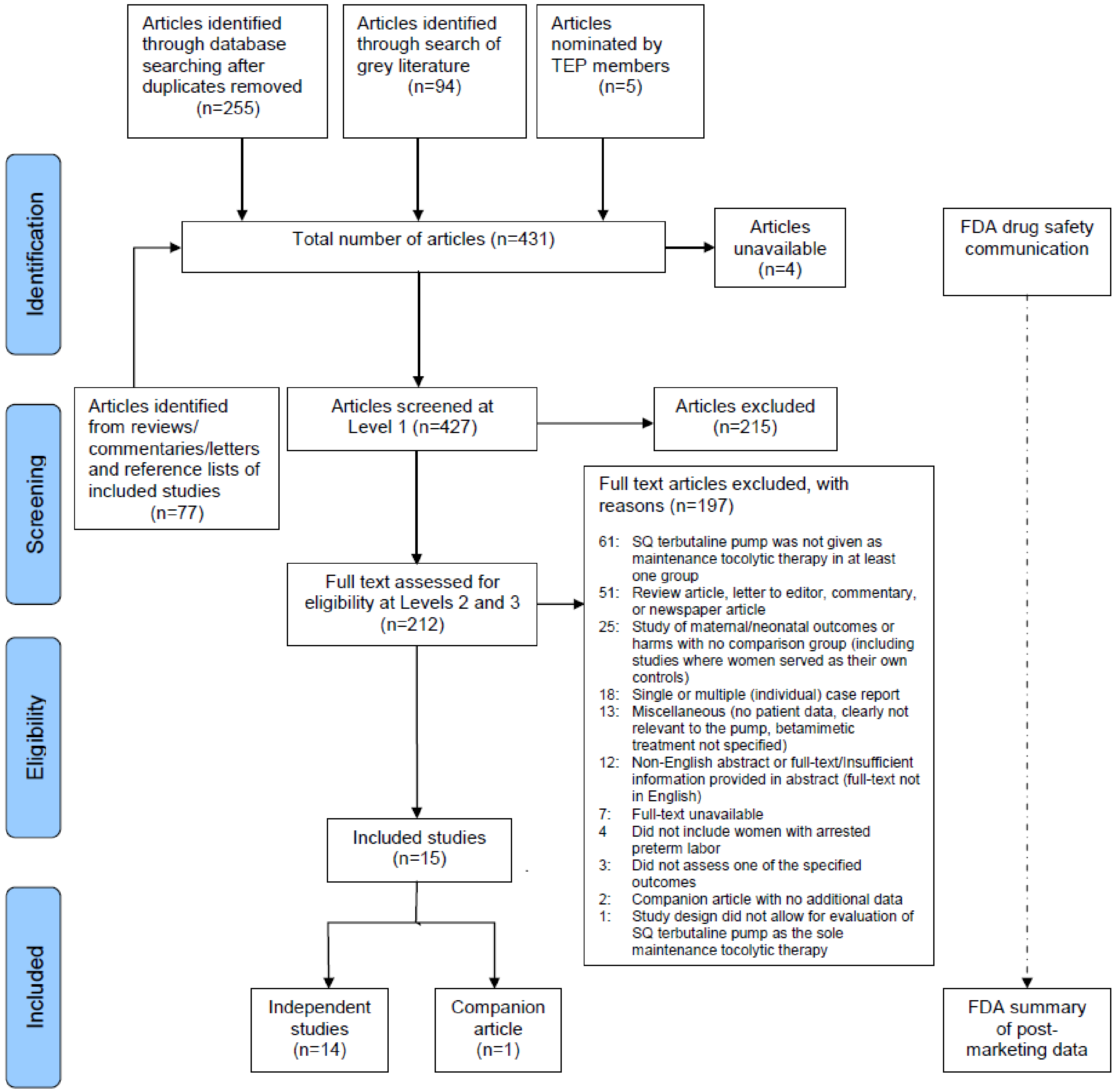

FDA = U.S. Food and Drug Administration; SQ = subcutaneous; TEP = Technical Expert Panel

Study Characteristics

Table A presents general summary characteristics of the included studies. Most studies were observational and included cohorts and case series. Two studies were RCTs, and one was a nonrandomized trial. Sample sizes ranged from 9 to 1,366, but greater than 70 percent of studies included at least 200 participants (average 291 ± 395). All studies were from the United States, and participants were recruited either from single-center study sites or from a national proprietary database run by Matria Healthcare. The Matria database provides an outpatient

perinatal program consisting of 24-hour nursing and pharmacy support, home uterine activity monitoring, individualized education, and provision of tocolytic therapy to women with preterm labor. Because five studies originated in the Matria database, and not all reported geographic region and/or years over which participants were recruited, the question of overlap in participants across these studies was an important concern of reviewers. Through the Scientific Resource Center (SRC), we requested this missing information from Matria (now called Alere) Healthcare but did not receive a response. Therefore, where appropriate, we report this risk of double-counting of participants.

Several studies included women with RPTL and singleton gestation. Comparator groups included placebo, no treatment, oral terbutaline, oral nifedipine, and mixed oral tocolytics. The definition of labor was unclear in 36 percent of the included studies. The remaining studies included women with persistent contractions and cervical change.

Table A. Summary characteristics of the included studies

Characteristic		Number of Studies	References
Study design	RCT	2	10,11
	Nonrandomized trial	1	12
	Prospective cohort	2	13,14
	Retrospective cohort	7	15-21
	Case series	2	22,23
Participant recruitment	Single center sites	9	10-14,20-23
	Matria database	5	15-19
Funding	Industry	2	10,22
	Nonindustry	3	14,20,21
	Not reported	9	11-13,15-19,23
Comparator*	Oral nifedipine	3	15-17
	Oral terbutaline	4	11,12,20,21
	Oral tocolytics	3	14,18,19
	Placebo (saline pump)	2	10,11
	No treatment	1	13
	No comparison group	2	22,23
Primary tocolytic treatments	IV magnesium sulfate only	1	23
	IV magnesium sulfate and/or other agents	5	10-13,22
	Not reported	8	14-21
Gestation	Singletons only	6	10,12,13,15,17,18
	Twins only	2	16,19
	Singletons and twins	2	11,22
	Not reported	4	14,20,21,23
Definition of labor	Not reported	5	15,17-19,21
Risk of bias**	Low	1	10
	Medium	7	12,16,17,19,20,22,23
	High	7	11-15,18,21
By Key Question	Key Question 1	6	10,11,13,17-19
	Key Question 2	12	10-21
	Key Question 3	6	10,12,13,18,19,21
	Key Question 4	1	11
	Key Question 6	3	11,22,23

RCT = randomized controlled trial; IV = intravenous
* One study contained two comparison groups. [11]
** Risk of bias of one study differed by outcome.[12]

Risk of Bias Assessment

We rated studies as low, medium, or high risk of bias for the relevant reported outcomes. Although the randomization procedures in the two RCTs were appropriate, we rated one RCT as low risk of bias[10] and the second RCT as high risk of bias because more than 90 percent of eligible participants declined to participate, the study was underpowered, and blinding was ineffective.[11]

The single nonrandomized trial was high risk of bias for the outcomes of birth weight and gestational age at delivery due to potential prognostic imbalances in groups. However, we did not anticipate that such imbalances would impact the outcome of maternal hyperglycemia, which we rated as medium risk of bias, due to insufficient information to assess several other criteria.[12]

We rated most of the cohort studies as high risk of bias because there were important group imbalances in baseline characteristics or prognostic factors.[13-15,18,21] The other cohort studies we rated as medium risk of bias; although these studies had no identifiable flaws, several criteria could not be assessed due to incomplete reporting.[16,17,19,20]

Lastly, we rated the two case series as medium risk of bias because neither study provided clear definitions for the pump-related harm outcomes, and several criteria, such as compliance, adequacy of sample size, and selective outcome reporting, were unclear.[22,23]

Neonatal Health Outcomes (KQ1)

Strength of evidence is insufficient for bronchopulmonary dysplasia, death within initial hospitalization, and significant intraventricular hemorrhage (grade III/IV). Based on one retrospective cohort of medium risk of bias, the strength of evidence favoring the SQ terbutaline pump compared with oral tocolytics for neonatal death in women with twin gestation and RPTL is low (Table B). This study investigated women from the Matria database and reported a statistically significant difference in neonatal death in favor of SQ terbutaline pump (OR = 0.09, 95% CI: 0.01, 0.70).[19] Sparse evidence from underpowered studies addressed necrotizing enterocolitis, retinopathy of prematurity, and sepsis with inconclusive results.[11,13] No data were available for periventricular leukomalacia and seizures.

Three retrospective cohort studies from the Matria database reported stillbirths in women with RPTL and single or twin gestation.[17-19] All three studies found nonsignificant differences between the SQ terbutaline pump and oral tocolytics. However, these studies were likely underpowered to detect a difference in still birth, given the small number of events (<1%).

Table B. SQ terbutaline pump versus comparator: Strength of evidence for populations of interest

Outcome	Number of Studies	Number of Participants	Total Number of Events	Population of Interest	Comparator	Effect Estimate	Strength of Evidence
**Neonatal Health Outcomes (KQ1):** BPD	0	0	N/A	N/A	N/A	N/A	Insufficient
**Neonatal Health Outcomes (KQ1):** Neonatal death*	1[19]	706	12	Twin gestation + RPTL	Oral tocolytics	OR = 0.09 (0.01, 0.70)	Low
	1[17]	284	0	Singleton gestation + RPTL	Oral nifedipine	OR = 1.00 (0.02, 50.75)	Insufficient
**Neonatal Health Outcomes (KQ1):** Death within initial hospitalization	0	0	N/A	N/A	N/A	N/A	Insufficient
**Neonatal Health Outcomes (KQ1):** Significant IVH (Grade III/IV)†	1[13]	60	4	Singleton gestation + RPTL	No treatment	OR = 0.30 (0.02, 5.85)	Insufficient
**Other Surrogate Outcomes (KQ2):** Incidence of delivery < 28 weeks	0	0	N/A	N/A	N/A	N/A	Insufficient
**Other Surrogate Outcomes (KQ2):** Incidence of delivery < 32 weeks	1[16]	656	192	Twin gestation + RPTL	Oral nifedipine	OR = 0.47 (0.33, 0.68)	Low
	1[19]	706	124	Twin gestation + RPTL	Oral tocolytics	OR = 0.52 (0.35, 0.76)	Low
	2[15,17]	1650	106	Singleton gestation + RPTL	Oral nifedipine	OR = 0.20-0.29 (lower CI range 0.07-0.16, upper CI range 0.52-0.61)††	Low
	1[18]	558	37	Singleton gestation + RPTL	Oral tocolytics	OR = 0.21 (0.09, 0.50)	Low
	1[13]	60	21	Singleton gestation + RPTL	No treatment	OR = 0.04 (0.00, 0.65)	Low
**Other Surrogate Outcomes (KQ2):** Incidence of delivery < 34 weeks‡	0	0	N/A	N/A	N/A	N/A	Insufficient

Table B. SQ terbutaline pump versus comparator: Strength of evidence for populations of interest (continued)

Outcome	Number of Studies	Number of Participants	Total Number of Events	Population of Interest	Comparator	Effect Estimate	Strength of Evidence
**Other Surrogate Outcomes (KQ2):** Incidence of delivery < 37 weeks[§]	2[15,17]	1650	925	Singleton gestation + RPTL	Oral nifedipine	OR = 0.72-0.75 (lower CI range 0.47-0.58, upper CI range 0.90-1.20)[††]	Insufficient
	1[18]	558	318	Singleton gestation + RPTL	Oral tocolytics	OR = 0.70 (0.50, 0.98)	Low
	1[13]	60	50	Singleton gestation + RPTL	No treatment	OR = 0.04 (0.01, 0.23)	Low
	1[20]	64	38	Singleton/ Multiple gestation + RPTL	Oral terbutaline	OR = 0.10 (0.03, 0.32)	Low
**Other Surrogate Outcomes (KQ2):** Mean prolongation of pregnancy (days)[**]	1[16]	656	N/A	Twin gestation + RPTL	Oral nifedipine	MD = 7.20 (4.10, 10.30)	Low
	2[15,17]	1650	N/A	Singleton gestation + RPTL	Oral nifedipine	MD = 6.20-7.50 (lower CI range 0.79-4.94, upper CI range 10.06-11.61)[††]	Insufficient
	1[18]	558	N/A	Singleton gestation + RPTL	Oral tocolytics	MD = 5.50 (2.28, 8.72)	Low
	1[13]	60	N/A	Singleton gestation + RPTL	No treatment	MD = 25.30 (16.77, 33.83)	Low
**Maternal Harms (KQ3):** Withdrawal-AE	0	0	N/A	N/A	N/A	N/A	Insufficient

BPD = bronchopulmonary dysplasia; CI = confidence interval; IVH = intraventricular hemorrhage; KQ = Key Question; MD = mean difference; N/A = not applicable; OR = odds ratio; RPTL = recurrent preterm labor; withdrawal-AE = withdrawal due to adverse effects

* One RCT also reported neonatal death.[11] No events occurred in the SQ terbutaline pump group or in the two comparator groups. We did not grade this evidence here because it did not pertain to any of the subgroups of interest.

† One RCT reported significant intraventricular hemorrhage.[10] No events were observed in pump or comparator groups. We did not grade this evidence here because it did not pertain to any of the subgroups of interest.

‡ Incidence of delivery < 34 weeks was reported in one RCT, which showed a nonsignificant difference between SQ terbutaline pump and placebo (OR = 0.95, 95% CI: 0.32, 2.87). We did not grade this evidence here because it did not pertain to any of the subgroups if interest.

§ Incidence of delivery < 37 weeks was also reported in one RCT, which showed a nonsignificant difference between SQ terbutaline pump and placebo (OR = 1.57, 95% CI: 0.49, 5.02). We did not grade this evidence here because it did not pertain to any of the subgroups of interest.

** Mean prolongation of pregnancy was also reported in two RCTs, with nonsignificant effect estimates. We did not grade this evidence here because it did not apply to any of the subgroups of interest.

[††] Studies were not pooled. Also, there was risk of double-counting of participants across these studies.

Other Surrogate Outcomes (KQ2)

Studies reported surrogate outcomes of preterm labor much more frequently than neonatal or maternal clinical endpoints. However, none of the included studies examined incidence of delivery < 28 weeks (strength of evidence is insufficient, Table B), need for oxygen per nasal cannula, or ratio of birth weight/gestational age at delivery.

Incidence of Delivery at Various Gestational Ages

Incidence of delivery < 32 weeks: The strength of evidence favoring SQ terbutaline pump compared with either oral tocolytics or no treatment is low for women with RPTL and those additionally with twin gestation (OR range = 0.04–0.52, 95% CI range: 0.00–0.35, 0.50–0.76) (Table B). The evidence originated in six, mostly Matria-based, cohort studies of medium to high risk of bias.[13,15-19]

Incidence of delivery < 34 weeks: The strength of evidence for this outcome is insufficient (Table B). One small RCT (n=52) that did not address any of the populations of interest, showed a nonsignificant difference between SQ terbutaline pump and placebo in women with singleton gestation.[10]

Incidence of delivery < 37 weeks: The strength of evidence favoring SQ terbutaline pump compared with oral tocolytics or no treatment is insufficient or low for women with RPTL (Table B). Four of five cohort studies of medium to high risk of bias, mostly from the Matria database, reported statistically significant differences in favor of SQ terbutaline pump (OR range = 0.04–0.75, 95% CI range: 0.01–0.58, 0.23–1.20).[13,15,17,18,20]

Mean Gestational age at Delivery

Larger cohort studies of medium to high risk of bias in women with RPTL and single or twin gestation demonstrated consistent benefit of SQ terbutaline pump compared with oral tocolytics or no treatment (RPTL and singleton gestation: difference in means range = 0.70–3.40 weeks, 95% CI range: 0.28–1.80 weeks, 0.98–5.00 weeks; RPTL and twin gestation: difference in means = 0.70 weeks, 95% CI range: 0.43–0.48 weeks, 0.92–0.97 weeks).[13,15-19] Most participants in the cohort studies came from the Matria database. RCT evidence not directly addressing the populations of interest yielded a nonsignificant effect estimate between the pump and placebo (n=52 and n=42).[10,11]

Prolongation of Pregnancy

The strength of evidence favoring SQ terbutaline pump compared with oral tocolytics or no treatment is insufficient or low for women with twin gestation and/or RPTL (difference in means range 5.50–25.30, 95% CI range: 0.79–16.77, 8.72–33.83) (Table B).[13,15-18] This evidence came from five cohort studies of medium to high risk of bias, mostly from the Matria database. Two small RCTs (n=52 and n=42), which did not pertain to any of the populations of interest, showed nonsignificant differences between SQ terbutaline pump and placebo.[10,11]

In one Matria-based cohort study, more women in the SQ terbutaline pump group had pregnancy prolonged > 7 days compared with women who received oral nifedipine (OR = 7.84, 95% CI: 3.59, 17.12).[15] Other Matria-based studies reported statistically significant benefits in favor of the pump compared with oral tocolytics for prolongation > 14 days (OR range = 1.93–3.47, 95% CI range: 0.87–2.34, 2.65–5.15).[15-19]

Birth Weight

Cohort studies of women with RPTL and single or twin gestation demonstrated statistically significant differences in mean birth weight in favor of SQ terbutaline pump compared with oral tocolytics or no treatment (range of mean difference in grams = 136–721, 95% CI range: 83–355, 189–1087).[13,16-19] Aside from one study, all were from the Matria database.[16-19] Two small RCTs (n=52 and n=42), which did not pertain to any of the populations of interest, reported nonsignificant differences between SQ terbutaline pump and placebo.[10,11]

Incidence of low birth weight (< 2500 g) and very low birth weight (< 1500 g) were reported in cohort studies. Most of these studies originated from the Matria database. All studies that reported low birth weight found statistically significant differences in favor of SQ terbutaline pump compared with no treatment or oral tocolytics (OR range = 0.24–0.64, 95% CI range: 0.06–0.51, 0.62–0.96).[13,15-19] Most studies also found statistically significant differences in favor of the pump for incidence of very low birth weight (OR range = 0.22-0.46, 95% CI range: 0.07–0.29, 0.60–1.06).[16-19]

Pregnancy Prolongation Index

Pregnancy prolongation index was reported in two cohort studies.[13,20] Both found statistically significant differences in favor of the SQ terbutaline pump compared with either no treatment or oral terbutaline (mean difference = 0.41, 95% CI: 0.26, 0.56; and 0.14, 95% CI: 0.02–0.26).

Need for Assisted Ventilation

One cohort study from the Matria database reported a nonsignificant difference between the SQ terbutaline pump and oral tocolytics in requirement for ventilator among infants with NICU admission.[18]

NICU Admission

Incidence of NICU Admission: Statistically significant differences in favor of the SQ terbutaline pump compared with oral tocolytics or no treatment were reported in cohort studies of women with RPTL and single or twin gestation (OR range 0.28–0.72, 95% CI range: 0.08–0.58, 0.63–0.97).[13,15-19] Again, most of these studies were Matria-based.[15-19] One small RCT (n=52), which did not pertain to any of the populations of interest, reported a nonsignificant difference between the SQ terbutaline pump and placebo.[10]

NICU length of stay: Statistically significant differences in favor of the SQ terbutaline pump compared with oral tocolytics or no treatment were also reported for NICU length of stay in mostly Matria-based cohort studies of women with RPTL and single or twin gestation (range of mean difference in days: -3.50 to -17.90, 95% CI range: -5.26 to -32.88, -1.74 to -3.54).[13,15,18,19] Another small RCT (n=42), which did not address any of the subgroups of interest, reported a nonsignificant difference between the SQ terbutaline pump and placebo or oral terbutaline.[11]

Maternal Harms (KQ3)

The strength of evidence is insufficient for Withdrawal-AE (Table B). One prospective cohort in women with singleton gestation and RPTL demonstrated highly unreliable odds favoring no treatment compared with the pump for tachycardia/nervousness (OR=25.48, 95% CI:1.23, 526.6).[13] Underpowered studies demonstrated indeterminate results for the outcomes of mortality, pulmonary edema, and therapy discontinuation (i.e., type II error cannot be excluded).[10,18,19] Two studies, a retrospective cohort and a nonrandomized trial, demonstrated

nonsignificant differences between the SQ terbutaline pump and oral terbutaline in the incidence of gestational diabetes, though type II error cannot be excluded. No data were available on heart failure, myocardial infarction, refractory hypotension, and hypokalemia.

Until 2009, 16 maternal deaths and 12 cases of maternal cardiovascular events (hypertension, myocardial infarction tachycardia, arrhythmias, and pulmonary edema) in association with terbutaline tocolysis were reported to the FDA. Of these, at least three maternal deaths and three cardiovascular adverse events were clearly reported to be in association with the use of the SQ terbutaline pump.[24]

Neonatal Harms (KQ4)

Neonatal harms data were very sparse. Neonatal hypoglycemia was reported in only one RCT that compared the SQ terbutaline pump with placebo and oral terbutaline.[11] Differences between the SQ terbutaline pump and placebo or oral terbutaline were nonsignificant. However, given the small number of events and limited sample size (n=42), the RCT was underpowered and the results are inconclusive. No studies reported neonatal hypocalcemia or ileus.

Assessment of Confounding by Level of Activity and Level of Care (KQ5)

Only a small number of studies could be rated for level of activity and level of care. Therefore, we could not carry out meta-regressions to explore the effect of these variables on maternal and neonatal outcomes. Furthermore, we could not even explore the impact of level of activity on effect estimates in a qualitative manner because all studies that could be rated were designated as having “low” level of activity. No apparent trends in effect estimates according to level of care based on qualitative assessments were observed.

Incidence of Pump Failure (KQ6)

Two case series and one RCT reported outcomes related to the pump device.[11,22,23] In a case series of 51 women, one participant had dislodgment of catheter (2 percent, exact central CI: 0.5%, 10%) and there was one pump that malfunctioned (2 percent, exact central CI: 0.5%, 10%).[22] No infusion site infections or mechanical failures were observed in a case series of nine women.[23] An underpowered RCT demonstrated indeterminate results for the outcomes of local pain and local skin irritation.[11] No data were available for missed doses or overdoses.

Applicability

In Table C below, we summarize the overall applicability of the evidence base, according to the domains of population, intervention, comparison, outcomes, timing, and setting.

Table C. Overall applicability of the body of evidence

**Population**	The majority of evidence pertained to women with recurrent preterm labor and singleton gestation in the United States. Very little information was reported about the study populations' demographic and clinical characteristics. Nine of 14 studies (64 percent) included women judged to be in labor on account of persistent contractions and cervical change. The definition of labor was unclear in other studies. Among the studies that suggested that the pump was efficacious, 50 percent reported cervical change and contractions as part of the definition of labor while 50 percent did not report how labor was defined.
**Intervention**	Although there were gaps in reporting, the intervention generally did not pose any serious limitations to applicability. Very few details were reported on cointerventions that could modify the effectiveness of therapy, such as administration of corticosteroids. In several studies, participants received specialized outpatient services from Matria Healthcare.
**Comparison**	Comparators included oral tocolytics, no treatment, and placebo.
**Outcomes**	Surrogate outcomes were the most commonly reported. Data on clinical outcomes, neonatal/maternal harms, and pump-related outcomes were sparse. Long-term outcomes have not been reported at all.
**Timing of Outcomes Measurement**	The absence of followup beyond delivery is a major limitation because important long-term outcomes have not been evaluated.
**Setting**	All studies were from the United States and participant data were acquired from a national database (Matria) or from single center sites. Women from the Matria database generally received a high level of care from an outpatient perinatal program. However, the distribution of regions from which patient data were included into the national database is unknown and information about the standards followed by the individual practice sites that provided obstetrical care was not reported. Similarly, for those studies that took place at single center sites, the standards of care followed at these sites are unclear.

Discussion

In this small review of 14 studies, most data came from observational designs, and several studies analyzed data from the Matria database. Aside from two RCTs, the studies exhibited considerable clinical and methodological heterogeneity. For the gradable outcomes, the available evidence addressed only two specific populations of interest—women with RPTL or those additionally with twin gestation. The strength of evidence favoring the SQ terbutaline pump compared with oral tocolytics for neonatal death in women with twin gestation and RPTL is low (OR = 0.09, 95% CI: 0.01, 0.70). While this result is striking in the presence of insufficient findings on other neonatal health outcomes summarized below, it is apparent that it stems from the largest of studies contributing data on neonatal health outcomes with more than 700 patients. As such, it is the only outcome that appears to be adequately powered to reach statistical significance. Strength of evidence favoring terbutaline pump compared to oral tocolytics or no treatment is also low for women with twin gestation and/or RPTL for the surrogate outcomes of pregnancy prolongation. For bronchopulmonary dysplasia, significant intraventricular hemorrhage, death within initial hospitalization, and Withdrawal-AE, strength of evidence is insufficient. The evidence was inconclusive for all other neonatal health outcomes, neonatal harms, maternal harms, and pump-related outcomes.

Based on postmarketing surveillance data, the FDA has issued a new warning against the use of terbutaline in general, and as an injection in particular, as maintenance tocolysis (i.e., beyond 48–72 hours) in pregnant women.[24] Although meriting transparent disclosure in the form of a warning, evidence emerging from case reports is usually regarded as noncomparative and hypothesis generating signal rather than a hypothesis testing confirmation.[25] Furthermore, case reports are useful in identifying rare and unexpected adverse events—the rarer the adverse event, the stronger is the effect size, and the magnitude of effect size is an important criterion that increases our confidence in an estimate.[9] However, adverse events such as death, hypertension,

myocardial infarction, tachycardia, arrhythmias, and pulmonary edema that were reported with the use of terbutaline are not so unexpected in any adult population—pregnant women may experience these adverse events in the absence of terbutaline therapy due to other reasons.

Observational studies of medium to high risk of bias, primarily from the Matria database, showed benefit of SQ terbutaline pump compared with oral tocolytics or no treatment for other surrogate outcomes, such as birth weight and NICU admission, for women with twin gestation and/or RPTL. In contrast, two small RCTs that did not address any of the populations of interest, reported nonsignificant differences for several surrogate outcomes.

The evidence base for this review contained several limitations. Most evidence came from observational designs of medium to high risk of bias. Several outcomes revealed nonsignificant results that could be attributed to type II error. Type II error is a statistical term that implies inability of studies to find a difference when it might truly exist because of their small sample size (false negative). Many important variables, such as race, socioeconomic status, and fetal fibronectin level were not reported. Furthermore, cointerventions, such as administration of corticosteroids, were rarely described. None of the included studies assessed long-term childhood outcomes, such as childhood development, neurobehavioral testing, long-term lung function, and long-term vision. Our review comprehensively reviewed the literature and selected reports based on well-defined inclusion and exclusion criteria. However, one potential limitation of our review process is that we excluded potentially relevant non-English publications. Also, we could not investigate the impact of publication bias. However, in completing this review, we undertook an extensive grey literature search. Further, we requested relevant scientific information from the industry and had many experts in the field participate in the review process. Despite this thorough process, the number of identified studies was very small—we had too few studies per outcome to perform statistical assessment of publication bias. We believe that all relevant data regarding the use of subcutaneous terbutaline for the prevention of preterm labor is captured in this review. Any exaggerated positive findings are more likely due to the medium to high risk of bias detected in observational studies than publication bias.

In conclusion, the available evidence suggests that pump therapy is beneficial as maintenance tocolysis. However, our confidence in the validity and reproducibility of this evidence is low. While postmarketing surveillance has detected cases of serious harms, safety of the therapy remains unclear.

Future Research

Although cohort studies have provided a glimpse of the potential for the SQ terbutaline pump to improve short-term neonatal outcomes for fetuses at risk for preterm birth, the answers to several important questions remain unanswered. Most importantly, it remains to be seen whether SQ terbutaline pump therapy alters long-term development or systemic impairment of offspring, and neonatal/maternal morbidity and mortality. The limitations of the available data must also be recognized. Most of the cohort studies were medium to high risk of bias. In addition, several of the cohort studies investigated participants from a single proprietary database (Matria), which raises concerns regarding double-counting of patients and common biases. Therefore, results showing effectiveness should be interpreted with caution, especially in light of the most recent FDA warning recommending against the use of terbutaline for maintenance tocolysis.

Information is lacking on the effectiveness and safety of SQ terbutaline pump as a maintenance tocolytic treatment in specific populations, including women who deliver at specific gestational ages, women of different racial or ethnic backgrounds, and women with previous

preterm birth or preeclampsia. Future studies, whether observational or experimental in design, should focus on garnering evidence for these specific populations.

Below we provide some specific recommendations for the conduct of RCTs and observational studies to further elucidate the potential benefits and harms of SQ terbutaline pump for maintenance tocolysis.

Randomized Trials

We recommend that an adequately powered randomized controlled and pragmatic clinical trial that assesses the SQ terbutaline pump as a maintenance tocolytic be conducted. A pragmatic RCT is designed to have broad applicability so that the results can guide decisions about practice.[26] Such a trial should be placebo controlled and include blinding of study participants, care providers, and study personnel. Consideration should be given to employing multiple treatment arms in order to evaluate the pump against other tocolytic agents and conservative management. Furthermore, the level of care provided to participants (i.e., nursing assessments, home uterine monitoring, education, telephone support, and restriction of activities) should be practical, feasible, and likely to be adopted in routine practice. Important cointerventions, such as administration of corticosteroids, should be reported. A full accounting of the number of women approached but not enrolled should be included to allow users to assess the impact of respondent bias. The analysis should be “intent to treat,” where all participants assigned by randomization to each group are included in the primary comparisons, regardless of whether the assigned medication was received. Outcomes to be examined should go beyond those of prolongation of pregnancy and birth weight to hard clinical endpoints of neonatal morbidity, such as bronchopulmonary dysplasia, necrotizing enterocolitis, significant intraventricular hemorrhage (grade III/IV), retinopathy of prematurity, sepsis, stillbirth, and neonatal death. Lastly, there should be long-term followup to assess subsequent childhood outcomes. Pharmacodynamic and pharmacokinetic outcome measures can additionally be studied to understand inter-individual differences in effectiveness and toxicity and avoidance of β-agonist related tachyphylaxis.

Conducting RCTs to assess the efficacy of tocolytics in general is notoriously difficult. A definitive trial in this domain must include a focus on accurate diagnosis of preterm labor (perhaps combining stringent clinical criteria with factors such as positive fetal fibronectin and shortened transvaginal cervical length). Emphasis must also be placed on securing funding and maintaining followup for an appropriate duration of time to allow assessment of long-term childhood outcomes, including neurobehavioral testing and developmental assessment.

Observational Studies

Although the RCT is the ideal study design for evaluating the efficacy of interventions, it may not be feasible for a number of reasons, such as a prohibitive sample size requirements and ethical considerations. We realize that collecting RCT evidence on clinically important outcomes may not be possible because a large number of patients will need to be recruited to detect rare events, such as maternal deaths. Therefore, we additionally propose:

- Well-designed, well powered cohort studies examining clinical outcomes. These studies should include a representative and inception cohort of all patients with arrested preterm labor. Since observational studies are susceptible to the effects of confounding, future observational studies should measure, report, and adjust for potential confounders such as fetal fibronectin, cervical length/dilation, cerclage, maternal characteristics (e.g., age, race), level of care and activity, and concomitant medications. Propensity scores based on

these variables may be considered. Other considerations about power, multiple comparison groups, level of care, reporting of cointerventions, and long-term followup are the same as for RCTs.

- Record linkage studies in which mothers' prenatal and infants' NICU and childhood developmental electronic health records are linked may be a more practical research proposition for the near future with improvements in quality and accessibility of electronic patient records. NICU registries in which prenatal data of mothers are available can be a very valuable source. However, such linkage based studies may also be impacted by biases not uncommon to cohort study designs, especially confounding because of unmeasured or unrecorded variables with important prognostic implications.

Introduction

Preterm birth continues to be one of the largest contributors to neonatal morbidity and mortality worldwide and is associated with both short- and long-term disability. Preterm birth is defined as delivery before the completion of the 37th week of gestation and affects 12.3 percent of live births in the United States.[1] According to the 2010 National Vital Statistics report, there were 542,893 preterm births in the United States in 2006.[2] Approximately 40 percent of preterm births occur after the spontaneous onset of preterm labor.[27] Rates of preterm birth result in a significant disease burden to the health care system. Interestingly, the most recent data suggest a modest decrease in the preterm birth rate.[28] When medically indicated preterm births are excluded, the rates of spontaneous labor appear to have fallen by 20 to 30 percent.[29] The reasons behind this encouraging improvement deserve further elucidation.

Although overall rates of neonatal mortality are declining, infants born too early continue to be at risk.[3] Early preterm deliveries (less than 32 weeks' gestation) comprise 2 percent of all births in the United States, yet 54 percent of all infant mortalities occur in this group.[3] Neonatal survival increases steadily with increasing gestational age, particularly prior to 32 weeks' gestation, largely due to advances in neonatal care. Although this improvement is promising, the resulting increase in short- and long-term morbidity and the effect on quality of life for survivors are of concern.

The diagnosis of preterm labor is elusive, largely because the exact sequence and timing of events are poorly described and incompletely understood. This is partly due to the multifactorial nature of preterm labor, which arises from several different pathways but culminates in the same outcome of preterm birth. Further, the symptoms of preterm labor (i.e., pelvic pressure, increased vaginal discharge, backache, and menstrual-like cramping) are often vague. In the past, the presence of regular uterine contractions was sufficient to diagnose preterm labor and much of the literature uses this criterion. Due to the lack of precision involved in diagnosis, up to 40 percent of women with a preterm labor diagnosis are not actually in labor.[30] Consequently, a significant proportion of women enrolled in clinical trials of tocolytic efficacy were not destined to deliver preterm.

Currently, most clinicians require appropriate evidence of progressive change in cervical dilation and/or effacement before a diagnosis of preterm labor is made. A diagnosis of preterm labor made based on contraction frequency of ≥ six per hour and cervical dilation ≥ 3 cm and/or effacement ≥ 80 percent, or if membranes rupture or bleeding occurs, is reasonably accurate.[31,32] Most clinicians, however, view any documented cervical change accompanied by regular contractions to indicate preterm labor, and intervene before the aforementioned criteria are met. Although doing so results in treatment of more women who are not destined to deliver preterm, early intervention is believed to benefit the infants of women who are experiencing true preterm labor. Documentation of cervical change is thought to increase the sensitivity and specificity of the diagnosis; this may allow for more rigorous evaluation of the effectiveness of treatments for preterm labor and reduce the number of patients treated unnecessarily.

Perinatal morbidity among premature infants can be loosely divided into short-term and long-term sequelae. Short-term outcomes include respiratory distress syndrome, intraventricular hemorrhage, necrotizing enterocolitis, sepsis, hypoglycemia, thermal instability, and jaundice.[33]Although these immediate concerns are important and often require treatment in the neonatal intensive care unit (NICU), long-term outcomes determine the overall quality of life for

children and their families. These outcomes include bronchopulmonary dysplasia, significant intraventricular hemorrhage (grade III/IV), and retinopathy of prematurity.

Preterm labor is notoriously difficult to treat, owing in large part to the multifactorial nature of the condition. Risk factors for preterm labor include history of preterm birth, multiple gestation, maternal nonwhite race, low socioeconomic status, maternal underweight status, and maternal stress.[34-36] Spontaneous preterm labor occurs in the absence of maternal or fetal illness. The ultimate goal of treating preterm labor is to reduce long-term mortality and morbidity of the offspring. Unfortunately, most studies to date have focused on the prevention of preterm birth using surrogate endpoints such as gestational age at delivery, birthweight, and NICU admission.

A tocolytic is a drug used to inhibit contractions and delay the parturitional process. Tocolytic therapy has thus far demonstrated poor efficacy, likely because the parturitional process is already well established. The goals of tocolysis are to reduce neonatal morbidity and mortality without causing significant maternal or neonatal side effects. Tocolytic therapy to date has primarily focused on short-term delay in delivery to allow for maternal administration of corticosteroids and transport to an appropriate facility for neonatal care. The most appropriate measures to assess the efficacy of tocolytic agents should focus on improved health outcomes for infants. To date, most tocolytic trials of maintenance therapy have insufficient power to assess such endpoints.

The majority of tocolytic agents used to inhibit uterine contractions are efficacious for a period of approximately 48 hours. In contrast, terbutaline sulfate has been used in selected patients as maintenance tocolytic therapy to inhibit uterine contractions for longer periods of time after an episode of preterm labor has been arrested acutely with first-line tocolytic agents (including but not limited to indomethacin, magnesium sulfate, nifedipine, and nitroglycerin). Terbutaline is a β-sympathomimetic drug that relaxes smooth muscle in the bronchial tree, blood vessels, and myometrium.[4] For maintenance tocolysis, terbutaline is delivered by a subcutaneous (SQ) pump, usually at a basal rate of 0.03–0.05 mg/hr with intermittent boluses of 0.25 mg every 4 to 6 hours.[37] As with all other contemporary tocolytics, the use of terbutaline for maintenance tocolysis is off-label. The Food and Drug Administration has approved terbutaline for the management of acute and chronic obstructive pulmonary disease.

Maternal side effects are common because terbutaline does not act on the myometrium alone. Although most side effects are mild and self-limiting, such as shortness of breath, chest pain, anxiety and fatigue,[10] serious adverse reactions such as pulmonary edema, myocardial ischemia, cardiac arrhythmias, hypotension, and metabolic alterations may also occur.[4]

Maintenance tocolytic therapy with a terbutaline pump has been evaluated in two systematic reviews. A Cochrane review concluded that the pump does not decrease the risk of preterm birth by prolonging pregnancy based on two small randomized trials (Guinn n=52 and Wenstrom n=42).[4] Further, lack of information on safety and costs to implement therapy do not support use of the pump for the clinical management of arrested preterm labor. Another review included both randomized trials from the Cochrane review and also four additional observational studies.[5] Results were contradictory, with randomized trials failing to show efficacy and observational studies demonstrating positive results.

An Agency for Healthcare Research and Quality (AHRQ) review examined the use of β-mimetic agents for maintenance tocolysis.[38] No benefits were observed for gestational age at birth, prolongation of pregnancy, or birthweight. In addition, β-mimetics were classified as conferring a high probability of maternal risk, including cardiovascular harms. However, the

investigators did not distinguish between first-line and maintenance therapies when assessing harms and the SQ terbutaline pump was not examined specifically.

Despite conflicting evidence from previous systematic reviews, proponents of the terbutaline pump believe it still has a role as a maintenance tocolytic agent in women with arrested preterm labor. Given the discrepancy between available data and clinical practice, AHRQ commissioned a new systematic review of the literature to solidify the benefits and harms of the SQ terbutaline pump for maintenance tocolysis.

In this report we have systematically reviewed and summarized the available literature on the use of terbutaline pump for maintenance tocolytic therapy in women with arrested preterm labor. This evidence report will add to previous systematic reviews by performing an up to date search of the literature, synthesizing evidence in the context of specific populations of women, addressing confounding by level of maternal activity and level of maternal care, and grading the strength of evidence for important outcomes to help decision-makers develop evidence-based recommendations and policies.

A systematic review of terbutaline pump for maintenance tocolysis will inform clinicians, patients, and policymakers about the appropriateness of ongoing use and provide support for clinical guidelines. We hope that this review will result in more safe and effective treatment of women with preterm labor and, ultimately, in a reduction in mortality and long-term morbidity for their offspring.

Based on comments received during the peer-review process, we made modifications to the format of the Key Questions (For details, see Appendix G).

Conceptual Framework and Key Questions

As shown in the analytic framework (Figure A, Executive Summary), we focused our evidence review on the following six Key Questions.

In women with arrested preterm labor, does treatment with a SQ infusion of terbutaline delivered by a pump, in comparison with placebo, conservative treatment or other interventions:

Key Question 1: improve neonatal health outcomes, including bronchopulmonary dysplasia, neonatal death, death within initial hospitalization, significant intraventricular hemorrhage (grade III/IV), necrotizing enterocolitis, periventricular leukomalacia, retinopathy of prematurity, seizures, sepsis, and stillbirth for the following subgroups:

a. Women <28 weeks, 0 days of gestation (extremely preterm)?
b. Women between 28 weeks, 0 days and 31 weeks, 6 days of gestation (very preterm)?
c. Women between 32 weeks, 0 days and 33 weeks, 6 days of gestation (preterm)?
d. Women between 34 weeks, 0 days and 36 weeks, 6 days of gestation (later preterm)?
e. Multiple gestation?
f. Racial or ethnic subgroups?
g. Women with previous preterm birth?
h. Women with history of preeclampsia?
i. Women with recurrent preterm labor (RPTL) and women without RPTL

Key Question 2: improve other surrogate outcomes, including gestational age at delivery, incidence of delivery at various gestational ages (<28 weeks, < 32 weeks, <34 weeks, <37 weeks), mean prolongation of pregnancy (days), birth weight, ratio of birth weight/gestational age at delivery, pregnancy prolongation index, need for assisted ventilation, need for oxygen per nasal cannula, and NICU admission for the following subgroups:

a. Women <28 weeks, 0 days of gestation (extremely preterm)?
b. Women between 28 weeks, 0 days and 31 weeks, 6 days of gestation (very preterm)?
c. Women between 32 weeks, 0 days and 33 weeks, 6 days of gestation (preterm)?
d. Women between 34 weeks, 0 days and 36 weeks, 6 days of gestation (later preterm)?
e. Multiple gestation?
f. Racial or ethnic subgroups?
g. Women with previous preterm birth?
h. Women with history of preeclampsia?
i. Women with RPTL and women without RPTL

Key Question 3: increase the maternal harms of arrhythmia, heart failure, hyperglycemia, hypokalemia, maternal mortality, myocardial infarction, pulmonary edema, or refractory hypotension, or result in an increased rate of maternal discontinuation of therapy and maternal withdrawal due to adverse effects (withdrawal-AE)?

Key Question 4: increase the neonatal terbutaline-related harms of hypoglycemia, hypocalcemia, and ileus?

Key Question 5: Can the differences in the outcomes above be partially explained by the differences in level of care (e.g., frequency of followup, nurse visits, concomitant treatment, etc.) and level of activity (e.g., other children in the home, marital/support status, working status, bed rest, etc.) between the terbutaline pump group and the comparator group?

Key Question 6: What is the incidence of failure of the pump device used for terbutaline infusion, including missed doses, dislodgment, and overdose?

The report is organized into the following chapters. The Methods chapter describes the review methodology and provides details about topic development, the search strategy, study selection, data extraction, and statistical analyses. The Results chapter provides the results, organized by the six key questions, and a description of the applicability of the body of evidence according to the PICOTS framework. In the Discussion chapter, we provide a discussion to summarize the key findings, enumerate final conclusions, and offer recommendations for future research. Appendixes A and B provide details about the search strategies and grey literature sources respectively. In Appendix C, we list the companies that were sent requests for Scientific Information Packet. Screening, data extraction, risk of bias, and applicability forms are provided in Appendix D. Appendix E provides the citations of excluded studies. Evidence tables with study-level data are presented in Appendix F. Finally, Appendix G describes modifications to the format of the Key Questions subsequent to the peer review process.

Methods

Topic Development and Refinement

With input from key informants, we developed the PICOTS (population, intervention, comparator, outcome, timing, setting), conceptual framework, and key questions during the topic refinement stage. The Key Questions were posted to the Effective Health Care Web site. The public was invited to comment on the Key Questions. After reviewing the public commentary, we drafted the final Key Questions and submitted them to the Agency for Healthcare Research and Quality for approval. The Technical Expert Panel (TEP) reviewed the protocol and provided additional clinical and methodological input. The analytic framework (Figure 1), which was developed by the review team in consultation with the TEP, outlines the main elements of each Key Question.

Figure 1. Analytical framework of terbutaline pump for maintenance tocolysis

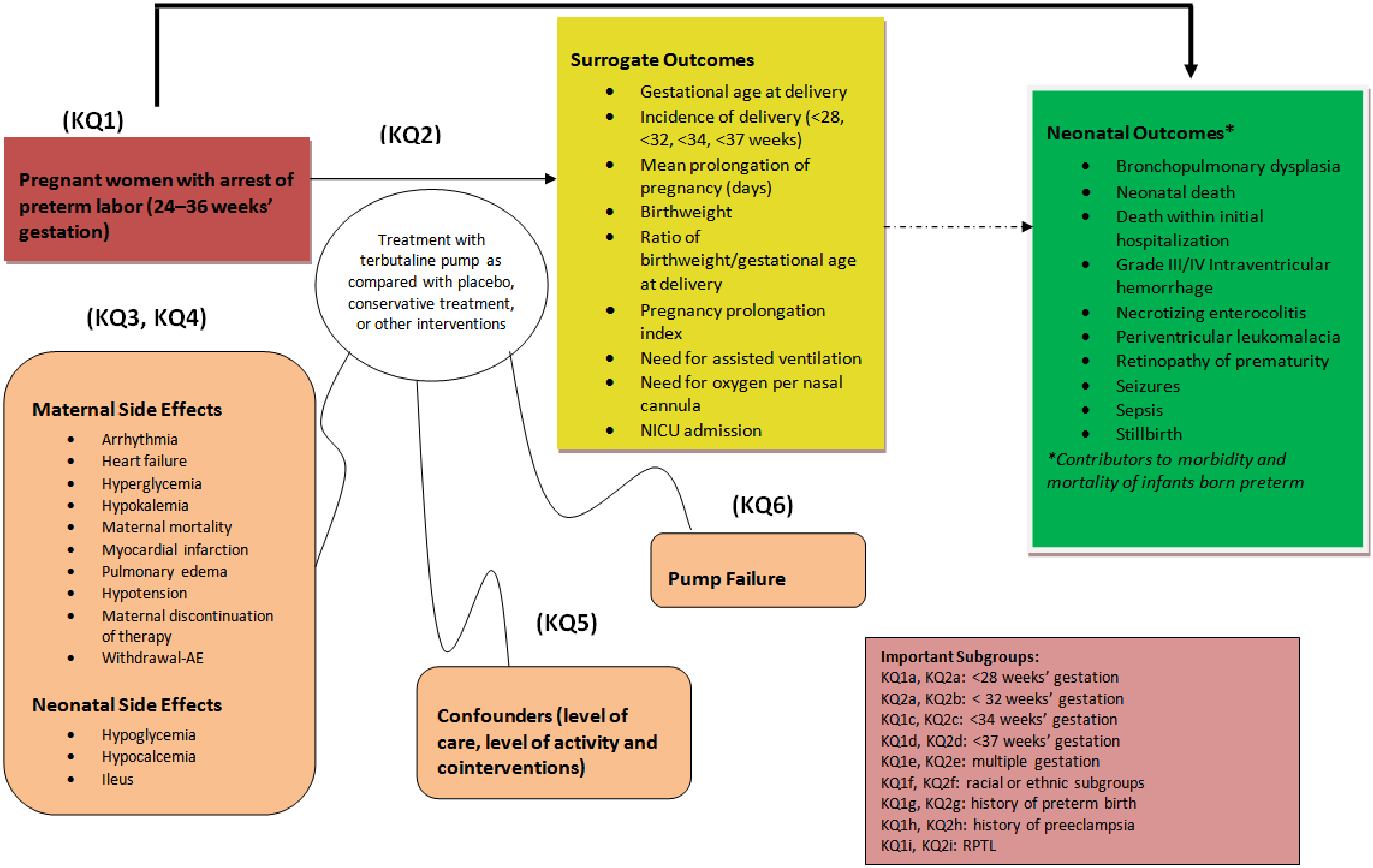

Search Strategy

In consultation with the rest of the team, our medical information specialist developed and tested electronic search strategies through an iterative process. Following published recommendations, MEDLINE and Embase strategies were peer reviewed by another information specialist using the PRESS Checklist and any amendments were subsequently applied to all databases.[39] A combination of controlled vocabulary and keywords were used in the search strategies and no restrictions were placed by date or language. The following databases were searched: Ovid MEDLINE In-Process & Other Non-Indexed Citations and Ovid MEDLINE (1950 to April 1, 2011); Ovid Embase (1980 to April 1, 2011); Cumulative Index to Nursing and Allied Health Literature (CINAHL) via EBSCOhost (1985 to December 7, 2009), the Cochrane Library (April 1, 2011) via Wiley interface (including CENTRAL, Cochrane Database of Systematic Reviews, Database of Abstracts of Reviews of Effects – DARE, Health Technology Assessment – HTA, and the National Health Service Economic Evaluation Database – NHS EED), and the Centre for Reviews and Dissemination (CRD) databases.(January 2,2010) Details of the search strategies are outlined in Appendix A.

We obtained additional references by hand-searching the bibliographies and text of review articles, letters to the editor, and commentaries identified during the screening of titles, abstracts, and full texts and with input from members of the TEP. We also hand-searched the reference lists of included studies for relevant citations.

We conducted a grey (unpublished) literature search by scanning the Web sites of relevant specialty societies and organizations, health technology assessment agencies, guideline collections, regulatory agencies, and trial registries (see Appendix B). The Scientific Resource Center (SRC) also conducted a grey literature search of regulatory information, clinical trial registries, abstracts and conference papers, grants and federally funded research, and N.Y, Academy of Medicine's Grey Literature Index (see Appendix B). Materials obtained from the grey literature searches were evaluated by one reviewer for additional relevant references. In February 2011, the US Food and Drug Administration (FDA) issued new warnings against the use of terbutaline to treat preterm labor, so we also accessed a summary of the FDA postmarketing surveillance results. This decision was made post hoc.

The SRC requested information about published and unpublished randomized controlled trials (RCTs) and observational studies from pharmaceutical companies (see Appendix C). We screened the Scientific Information Packages that were submitted by industries, and sought unpublished information from Matria (now called Alere) Healthcare about their perinatal program and associated database.

The searches yielded a total of 431 citations after removal of duplicates. All citations were imported into an electronic database for screening and data extraction (Distiller Systematic

Review Software; an Internet-based software program intended to facilitate collaboration among reviewers during the screening of abstracts and full texts, data extraction, exclusion reports, and table construction).

Study Selection

We developed inclusion and exclusion criteria based on the patient population, intervention, outcome measures, and study designs specified for the Key Questions. We screened titles and abstracts at Level 1 and full texts at Levels 2 and 3. The full-text articles of relevant abstracts, as assessed at Level 1 screening, were retrieved and assessed for relevancy by reapplying the

inclusion criteria at Level 2 and Level 3 screening. The purpose of Level 3 screening was to further classify studies based on outcome and study design. Articles that passed through Level 1 to Level 3 screening were included in the review (see Appendix D for Level 1, 2, and 3 screening forms). Non-English language records without an English abstract were excluded. Results published only in abstract form were considered for inclusion only if sufficient information was presented to assess eligibility and validity. Two reviewers independently screened abstracts and full-text articles. Conflicts were resolved by consensus or by third-party adjudication.

Studies with the following population, intervention, comparators, and outcomes were included:

- Population: Pregnant women 24–36 weeks' gestation and with preterm labor that had been arrested with primary tocolytic therapy
- Intervention: Subcutaneous terbutaline (SQ terbutaline) delivered by infusion pump
- Comparators: Either placebo, conservative treatment, or any other intervention
- Outcomes:
    1. Primary (neonatal) outcomes included bronchopulmonary dysplasia, necrotizing enterocolitis, significant intraventricular hemorrhage (grade III/IV), periventricular leukomalacia, seizures, sepsis, stillbirth, retinopathy of prematurity, death within initial hospitalization, and neonatal death.
    2. Secondary (surrogate) outcomes included gestational age at delivery (continuous variable), incidence of delivery at various gestational ages (<28, <32, <34, <37 weeks), mean prolongation of pregnancy (days), need for assisted ventilation, need for oxygen per nasal cannula, neonatal intensive care unit admission, birth weight, ratio of birth weight/gestational age at delivery, and mean pregnancy prolongation index. Although not specified in the protocol, prolongation of pregnancy was also extracted as a dichotomous variable (i.e., prolongation > 7 days and prolongation > 14 days).
    3. Maternal side effects included pulmonary edema, heart failure, arrhythmia, myocardial infarction, refractory hypotension, hypokalemia, hyperglycemia, maternal withdrawal due to adverse effects (withdrawal-AE), maternal discontinuation of therapy, and death. Neonatal side effects included hypoglycemia, hypocalcemia, and ileus.
    4. Outcomes of pump failure included missed doses, dislodgment, and overdose.
    5. Long-term childhood outcomes included childhood development, neurobehavioral testing, long-term lung function, and long-term vision.

We also included observational studies because very few RCTs were available on this topic. We considered prospective and retrospective cohort studies, case-control studies, cross-sectional studies, and case series (exclusively for outcomes related to pump failure) as eligible study designs. As a post hoc decision, we sought FDA summaries of postmarketing data highlighting serious harms.

We did not undertake indirect comparisons of RCTs of other tocolytics because, based on a scoping literature search, sparse evidence was anticipated for maintenance tocolytic therapies—mostly single RCTs of various tocolytics, such as atosiban, nifedipinie, and ritodrine. [40-42] Comparisons from such scant indirect evidence would likely have been inconclusive. Furthermore, indirect comparisons are premature at this point because the efficacy of maintenance tocolysis versus no maintenance tocolysis or placebo remains to be clearly

established. Indirect comparisons are helpful when direct comparisons of otherwise efficacious treatments are not available.

Data Extraction

We extracted the following items using the online program Distiller Systematic Review Software: general study characteristics (e.g., year of publication, country of origin, study design, setting, number screened, number included), population characteristics (e.g., inclusion/exclusion criteria, age, race, ratings for maternal level of activity), intervention characteristics (e.g., dose, duration, ratings for maternal level of care, details about comparators), outcomes (definitions and results), risk of bias, and applicability. One reviewer provided ratings for maternal level of activity, maternal level of care, and summarized applicability characteristics. Level of activity was rated as low, normal, or high based on a composite assessment of the following variables: marital status, working status, caring for other children in the home, available social support, bed rest, and restriction of maternal activities. Level of care was rated as low, moderate, or high based on the following variables: nursing assessments, home uterine activity monitoring, home visits, education about preterm labor, telephone support, restriction of maternal activities, and other cointerventions. Each variable was provided a rating based on predefined criteria (Tables F12 and F14 in Appendix F). We categorized responses into three tier levels and compared each level with another to decide the ratings of low, moderate/normal, and high. These assessments were verified by a clinical expert, with consensus reached by discussion. All other data were extracted by one reviewer, and outcome data was verified by a second reviewer.

When there were multiple reports of the same study, we referenced the most relevant record as the primary identifying study and extracted additional data as available from the companion report(s).

Risk of Bias Assessment

We evaluated risk of bias for each relevant outcome in individual studies using generic criteria for controlled trials and observational studies. Selected items from the McMaster Quality Assessment Scale of Harms were also incorporated into the assessment for those studies that evaluated treatment harms.[8] Two reviewers assessed risk of bias and consensus was reached by discussion or involvement of a third team member. Appendix D presents the risk of bias form used to evaluate studies.

The following risk of bias criteria were evaluated for all included study designs (RCTs, nonrandomized trials, and observational studies including case series):

- Extent to which valid primary outcomes were described. We considered both an explicit and implicit description as adequate, and assessed this only for the stated primary outcomes.
- Differential loss to followup between the compared groups or overall high loss to followup.
- Selective outcome reporting.
- Data quality (i.e., consistency of measurements across outcome assessors and consistency in outcome definitions across data sources – the latter point pertained only to retrospective cohorts).
- Adequacy of sample size.
- Compliance with treatment regimen.

- Selected criteria from the McHarm checklist for studies assessing treatment harms (definition of harms, mode of harms collection, and training/background of personnel collecting harms data).

The following criteria were assessed for all included study designs aside from case series:

- Similarity of groups in terms of baseline characteristics and prognostic factors
- Similarity of groups in terms of administration of primary tocolytic regimen to control acute episodes of preterm labor
- Intention-to-treat analysis
- Differential level of care between the compared groups

Blinding of patients, health care providers, and outcome assessors to treatment allocation and maternal contractions was assessed only for experimental designs (i.e., RCTs and nonrandomized trials). Based on the outcomes of interest, the outcome assessor was assumed to be the same as the health care provider. Two criteria, which pertained exclusively to RCTs, were generation and concealment of the allocation sequence. Two additional criteria were applied only to observational studies (excluding case series) and nonrandomized controlled trials. These included an assessment of whether the same population was used to sample intervention and comparison groups and methods used to control for confounders.

We evaluated intention-to-treat by examining both loss to followup/discontinuation of treatment and unintended crossover to opposite intervention group(s). Loss to followup was assessed either by what was reported in the study or, if not clearly reported, by comparing the number of participants who entered the study with the number of participants reported in outcome table(s). Unlike randomized controlled trials, for which numbers randomized are reported, the reported sample size of nonrandomized studies could be a posthoc determination depending upon the number of participants left for analysis. Therefore, comparing the number of study participants with the number of participants analyzed as reported in tables may not truly reflect those who were lost to followup or dropped out for nonrandomized studies. For such study designs, assessment of intention-to-treat analysis required that the study reports the number of participants who met inclusion/exclusion criteria.

For each relevant outcome in a study, we provided an overall risk of bias rating, designated as high, medium, or low (Table 1). We made these summary ratings within a study design. In order to be classified as high risk of bias, a study must have demonstrated some apparent and major flaw (within that study design category) that would invalidate results.

Table 1. Overall risk of bias ratings

**Low risk of bias.** These studies have the least bias, and results are considered valid. Studies that adhere mostly to the commonly held concepts of high quality including the following: a formal randomized controlled design; clear description of the population, setting, interventions, and comparison groups; appropriate measurement of outcomes; appropriate statistical and analytic methods and reporting; no reporting errors; low dropout rate; and clear reporting of dropouts.
**Medium risk of bias.** These studies are susceptible to some bias, but it is not sufficient to invalidate the results. They do not meet all the criteria required for a rating of good quality because they have some deficiencies, but no flaw is likely to cause major bias. Studies may be missing information, making it difficult to assess limitations and potential problems.
**High risk of bias.** These studies have significant flaws that imply biases of various types that may invalidate the results. They have serious errors in design, analysis, or reporting; large amounts of missing information; or discrepancies in reporting.

Grading the Body of Evidence

The strength of a body of evidence was graded based on the following four domains as per previously published guidance: overall risk of bias by outcome, consistency, directness, and precision.[9] Optional domains such as dose-response association and existence of confounders were considered as not relevant to this comparative effectiveness review. Publication bias was also not considered as an important concern because we searched for grey literature, scientific information packets from industries, and had many experts in this field participate as Key Informants, Technical Expert panelists and peer reviewers. No concerns about additional unpublished studies were raised. Furthermore, as we had few studies per outcome, publication bias could not be statistically investigated.[43]

In consultation with the TEP, the review team chose the following outcomes for grading: incidence of delivery at various gestational ages (<28 weeks, <32 weeks, <34 weeks, <37 weeks); mean prolongation of pregnancy; bronchopulmonary dysplasia; significant intraventricular hemorrhage (grade III/IV); neonatal death and/or death within initial hospitalization; and, maternal Withdrawal-AE. These outcomes were chosen based on importance to patients and clinicians. Each domain was graded by two reviewers, and consensus was reached by discussion.

We used four domains to grade outcomes: overall risk of bias, consistency, directness, and precision. For the body of evidence from observational studies, an initial grade of "low" could be upgraded across the domains where possible. We took care not to double count the inherent limitations of observational studies, so we did not factor in study design when assessing risk of bias. The overall risk of bias of an observational study, therefore, could potentially be "low." We took into account the inherent limitations of observational study designs when we graded the strength of evidence.

Applicability

We considered several factors to assess the applicability of the body of evidence. Population factors included breadth of inclusion/exclusion criteria, exclusion rate, patient demographics, and attrition rate. Intervention factors included dosing and treatment schedules, cointerventions, level of care, pump training, and dose of comparative agent. Outcomes were judged based on clinical utility, definition of harms, and timing of measurement. Geographic and clinical settings were also assessed.

One reviewer summarized the applicability of the body of evidence using the determinants of PICOTS (population, intervention, comparison, outcome, timing, and setting) and a clinical expert provided verification (see Appendix D for applicability form).

Important determinants of applicability (population, intervention, and comparator) are presented by outcome for the available evidence. However, this information was not presented if the strength of evidence for an outcome was graded as insufficient (i.e., absent or inconclusive evidence).

Data Synthesis and Analysis

We used a random effects model, following a DerSimonian and Laird approach, to meta-analyze study estimates if they met the following criteria of clinical and methodological homogeneity: (1) same study design, (2) no important differences in the following factors: demographic and obstetrical characteristics; level of care; intervention; comparator type, dose,

and frequency of administration; definition of outcome; timing; and clinical setting, and (3) similar risk of bias ratings. We compared SQ terbutaline pump with no treatment, saline infusion, or another tocolytic. If observational studies presented adjusted odds ratios (ORs), then we extracted and used these values in analyses. Otherwise, ORs and 95 percent confidence intervals were calculated for relevant outcomes in each included study. If a study group had no events, we added 0.5 to both event and nonevent cells. An OR of less than one indicates a smaller event rate in the SQ terbutaline pump group. Exact central confidence intervals were calculated for incidence rates presented in case series. These estimates were not meta-analyzed because only single studies were available by outcome. Statistical heterogeneity was assessed using Cochran's Q (α=0.10) and I^2 statistic was calculated to quantify the magnitude of heterogeneity. All analyses were performed using Comprehensive Meta Analysis version 2.2.046 or version 2.2.055 (New Jersey, USA).

We considered observational studies for meta-analysis only if the reports made it clear that they were similar with respect to major confounding factors (e.g., age, race, comorbidities, history of preterm birth, cervical length, cervical dilation, and fetal fibronectin). Although some studies matched for either one or more variables (e.g., by gestational age)[18-20] in no case was it apparent that there was equivalency in all or even most of these confounders. Therefore, observational studies were not pooled for any of the key questions, even if they were similar with respect to the PICOTS domains.

Studies that were exclusively of women with singletons were not pooled with studies exclusively of women with multiple gestation to avoid the unit of analysis error due to the cluster effect. Clustering may arise in studies of women with multiple gestation because the unit of randomization or allocation is the mother rather than infant. Also, pooling was not carried when we could not rule out the probability that participants were double counted (i.e., use of the same participants and their outcomes data in different studies). A qualitative analysis was conducted on those studies that could not be synthesized quantitatively.

We needed a minimum of six studies to explore statistical heterogeneity in effect estimates through meta-regression. Since we could pool only a small number of studies, meta-regression was not possible.

Results

Literature Search

The PRISMA diagram in Figure 2 depicts the flow of retrieved records through the phases of screening and inclusion. The titles and/or abstracts of 427 citations were screened at Level 1. These citations were identified from database searching, reference lists, grey literature, and Technical Expert Panel nomination. We did not identify any relevant data in the Scientific Information Packets submitted by pharmaceutical industries.

At full-text screening, 212 records were reviewed. Most records (n=197) were excluded with reasons listed in the PRISMA diagram. Ultimately, 14 unique records comprised the evidence base to answer the key questions. One record was an informative companion article for the study by Allbert et al. (1994).[20,44] A list of excluded studies is provided in Appendix E.

Figure 2. PRISMA diagram

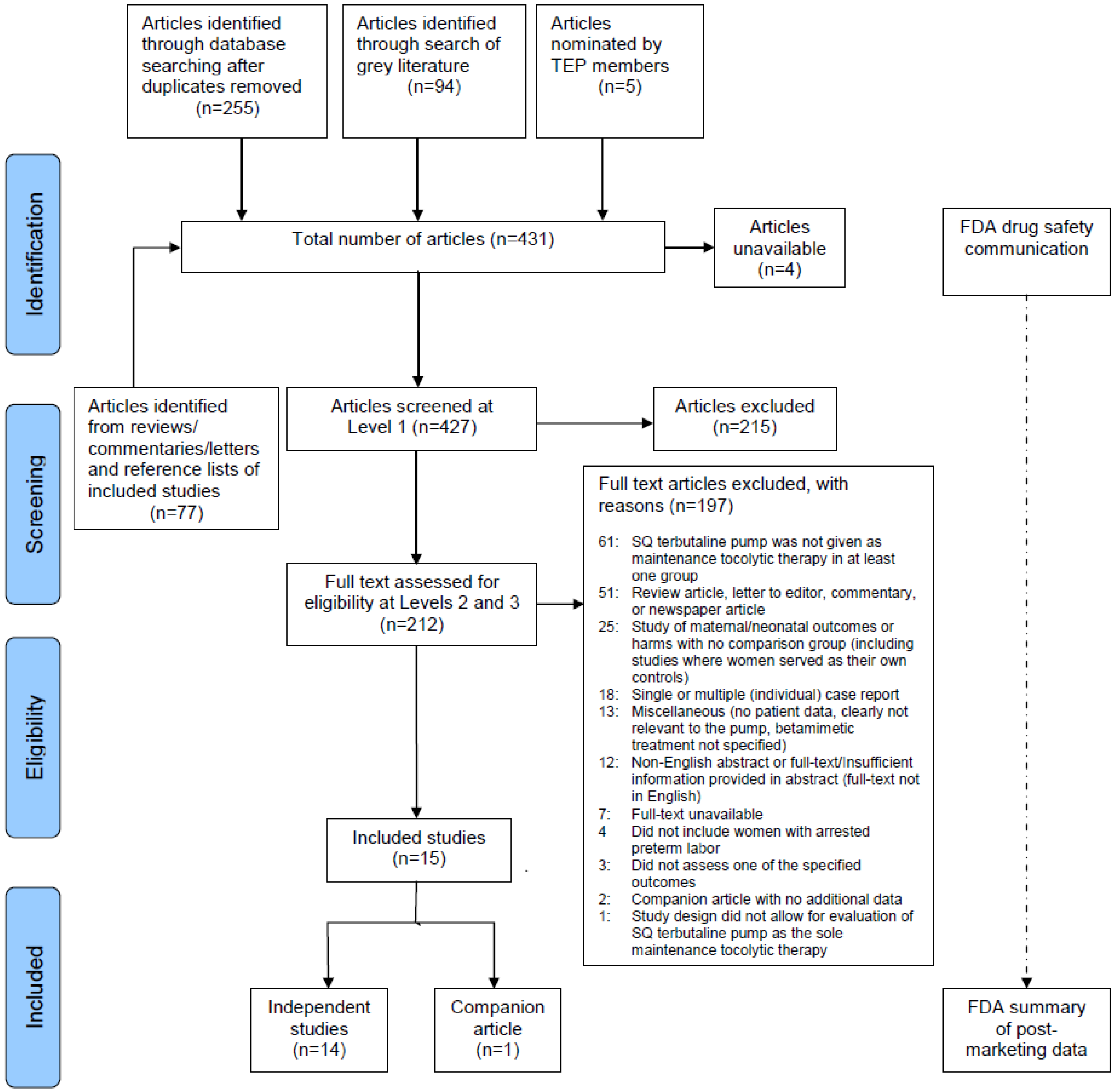

FDA = U.S. Food and Drug Administration; SQ = subcutaneous; TEP = Technical Expert Panel

General Study Characteristics

Most studies were observational in design and all were from the United States. Participants were recruited from single center study sites (n=9)[10-14,20-23] or from a national proprietary database run by Matria (now called Alere) Healthcare, which provides an outpatient perinatal program consisting of 24-hour nursing and pharmacy support, home uterine activity monitoring, individualized education, and provision of tocolytic therapy to women with preterm labor

(n=5).[15-19] The comparison groups were placebo (saline pump),[10,11] no treatment,[13] oral terbutaline,[11,12,20,21] oral nifedipine,[15-17] and oral tocolytics.[14,18,19] The definition of labor was unclear in 36 percent of the included studies. The remaining studies included women with persistent contractions and cervical change.

Population

All studies included women who had at least one episode of preterm labor that was arrested with primary tocolytic treatment (often with parenteral magnesium sulfate) with subsequent placement on a maintenance tocolytic regimen. Nine of 14 studies reported persistent contractions or contractions > 4 per hour accompanied by cervical changes as their definition of labor (Appendix F, Table F1). In several studies, only women with two or more episodes of preterm labor during the same pregnancy (i.e., recurrent preterm labor) were eligible for inclusion.[13-20,23] Several studies were conducted exclusively in women with singleton gestation,[10,12,13,15,17,18] although a few studies evaluated women with twins only.[16,19] Some studies may have included women less than 24 weeks' gestational age (an inclusion criterion for the review was 24–36 weeks plus 6 weeks), but data for these participants could not be separated.[10,15-20,22]

No studies presented data on concomitant medications, body mass index (BMI), history of preeclampsia, cervical position, cervical consistency, cervical station, Bishop's Score, or fetal fibronectin. Table 2 presents other maternal characteristics that were reported in the studies.

Table 2. Maternal characteristics

Characteristic	Number of Studies That Reported Characteristic	Value
Mean maternal age	12[10-16,18-22]	21.6–32.4 years
Mean GA at preterm labor	6[13,15-19]	29.5–31.6 weeks
Mean GA at start of therapy	6[10-12,20,22,23]	29.1–32.2 weeks
Race	5[10,13,19-21]*	European (white); Hispanic; African; Asian; Other ("nonwhite")
Comorbidities	2[13,22]	Bacterial vaginosis; asthma; urinary tract infection; fibroids; chronic hypertension/pregnancy induced hypertension; HELLP syndrome
History of preterm birth	7[10,13,16-19,22]	10.8–75%
Cerclage	6[15-19,22]	2.8–13.1%
Gravidity	2[11,18]	2.6 (mean)[18]<br>2.25, 2.5, 2.6 (medians)[11]
Parity	3[11,14,20]	1.2 (mean),[20] 1.4 (mean)[14]<br>0.5, 0.5, 0.7 (medians)[11]
Membrane status	8[10,13-16,20,22,23]	Intact
Mean cervical length	1[23]	0.2 cm
Mean cervical dilation	5[10,11,13,20,23]	1.7–2.9 cm
Cervical effacement	1[10]	50% (median)

GA = gestational age; HELLP syndrome = hemolysis, elevated liver function values, low platelet count
* None of these studies reported separate effect estimates for different races.

Intervention

At least one group was administered subcutaneous terbutaline (SQ terbutaline) infusion by pump for maintenance tocolysis in all studies. The basal infusion rate was 0.05 to 0.086 mg/h.[10,18-20,23] In several studies, the basal infusion was individualized based on uterine activity pattern, subjective symptoms, BMI, and/or other pharmacokinetic parameters.[12,13,15-17,19,22] The mean bolus dose was 0.24 to 0.30 mg.[10-13,15,18,19,22,23] Most studies provided some details about pump training, such as instruction on self-management, site selection and care, syringe change, administration of additional bolus doses, and/or heart rate monitoring.[10,13,15-20,22,23]

No study reported data on dose of primary tocolytic agents, compliance with pump protocol, or brand name of terbutaline. Other intervention characteristics are presented below in Table 3.

Table 3. Intervention characteristics

Characteristic	Number of Studies That Reported Characteristic	Value
Primary tocolytic agent	6[10-13,22,23]	Magnesium sulfate (IV); indomethacin (PO, PR); terbutaline (SQ injections); ritodrine (IV); nifedipine (PO)
Corticosteroid use during acute tocolysis	1[11]	IM betamethasone
Mean number of boluses per day	5[11,18-20,23]	5.4 - 8
Mean terbutaline dose per day	4[17-19,21]	2.5 - 3.9 mg
Pump type	6[10,12,18,19,22,23]	AS6-C U300; microinfusion pump; model 404-S; model 404-SP
Pump manufacturer	10[10-14,17-19,22,23]	Autosyringe, Travenol; Minimed Technologies; Disetronics

IM = intramuscular; IV = intravenous; PO = oral; PR = rectal; SQ = subcutaneous

Double-counting of Outcomes Data

Five studies originated in the Matria database, and not all reported geographic region and/or years over which participants were recruited. Therefore, the question of overlap in participants across these studies was an important concern of reviewers. Through the Scientific Resource Center, we requested this missing information from Matria (now called Alere) Healthcare but did not receive a response. Hence, where appropriate, we report this risk of double-counting of participants.

Participants may have been double-counted among the following sets of Matria-based studies:

- Flick et al. and Fleming et al. both selected women with singleton gestation who were prescribed oral nifedipine as a comparator and Lam et al. (2003) selected women with singleton gestation prescribed oral tocolytics as a comparator. Participants may have been double-counted in both SQ terbutaline pump and comparator groups in the studies by Flick et al. and Fleming et al.[16,17] Double-counting of participants in the comparator group between Flick et al. or Fleming et al. and the latter third study is expected to be minimal because 95.3 percent in the comparator group of Lam et al. were taking oral terbutaline.[18] However, there is a possibility that the SQ terbutaline pump sample of participants overlapped in all three studies.
- de la Torre et al. and Lam et al. (2001) selected women with twin gestation and used oral nifedipine or oral tocolytics as comparators respectively.[16,19] Double-counting of participants in the comparator groups will be minimal because 92.3 percent of

participants in the oral tocolytic group of the study by Lam et al. (2001) were administered oral terbutaline.[19] However, there is still a possibility that participants in the SQ terbutaline pump groups of these two studies overlapped.

Risk of Bias Assessment

Figures 5, 6, 7, and 8 present risk of bias charts for cohorts, case series, nonrandomized trials, and randomized controlled trials (RCTs) respectively. The bars in black represent studies with risk of bias, and the bars in grey represent studies without risk of bias for the corresponding criteria. Studies that were rated as unclear are represented by white bars. Table F2 in Appendix F presents the full text question that was posed for the criteria listed in the charts. The overall ratings for risk of bias of individual studies are presented in the evidence table (Table F1, Appendix F). Table F3 in Appendix F presents detailed risk of bias assessments for each study.

We rated studies as high risk of bias if we could identify at least one major flaw with the potential to significantly bias results. If we could not assess several factors due to incomplete information, but there was no major flaw, then we rated studies as medium risk of bias. We provided a rating of low risk of bias only if there were no identifiable flaws and there was sufficient information to evaluate most criteria.

Cohort Studies (n=9)

We rated five cohort studies as high risk of bias [13-15,18,21] and four as medium risk of bias (Figure 3).[16,17,19,20] The cohort studies with high risk of bias all had imbalances in baseline characteristics or prognostic factors. We rated the remaining cohort studies as medium risk of bias because we could not assess several criteria due to incomplete reporting.

Figure 3. Risk of bias assessment for cohort studies

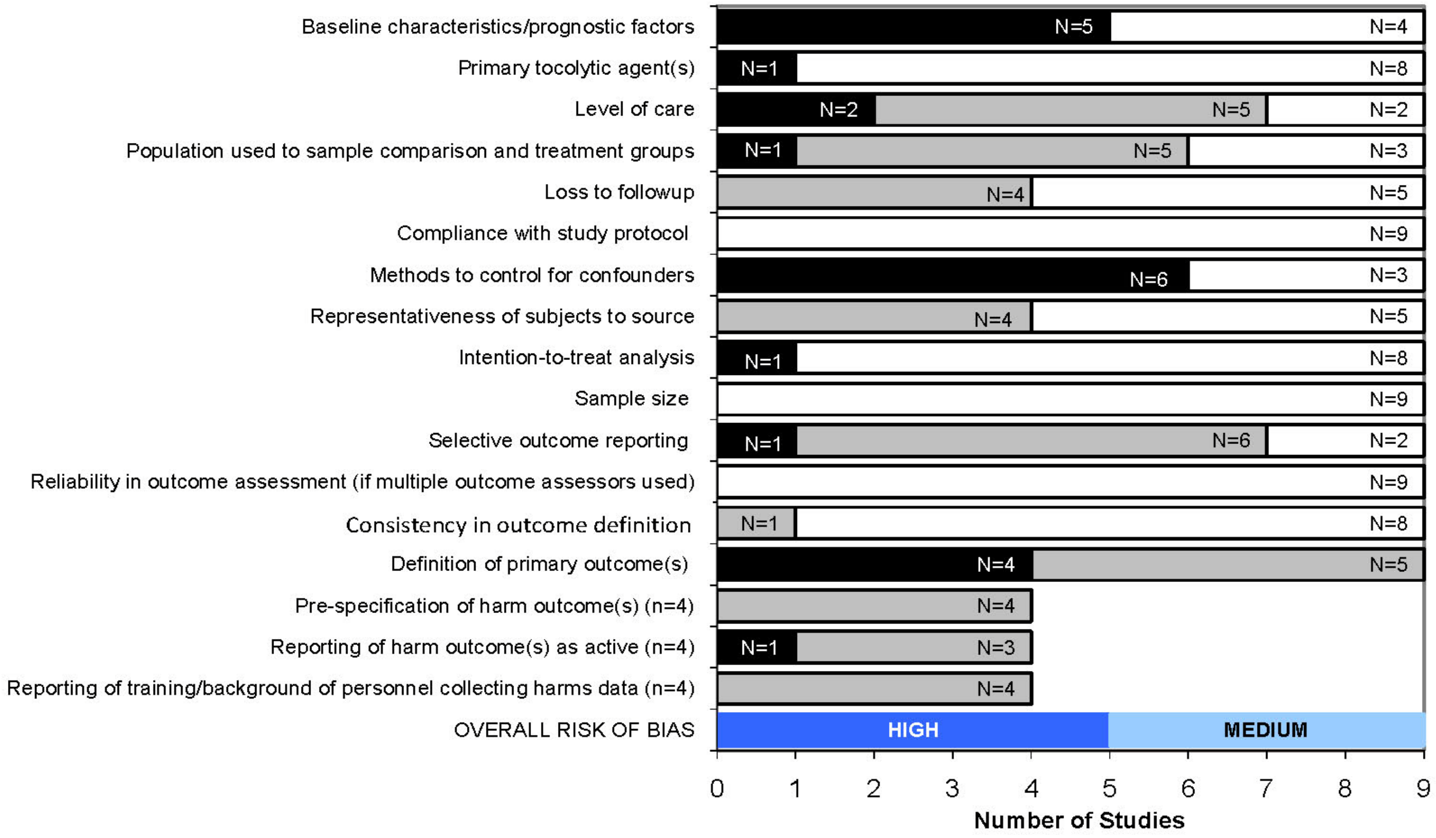

Case Series (n=2)

We rated both case series as medium risk of bias (Figure 4).[22,23] Although we could not identify any major methodological flaws, neither study provided clear definitions for the pump-related harm outcomes. Several criteria, such as compliance, adequacy of sample size, and selective outcome reporting, were unclear.

Figure 4. Risk of bias assessment for case series (n=2)

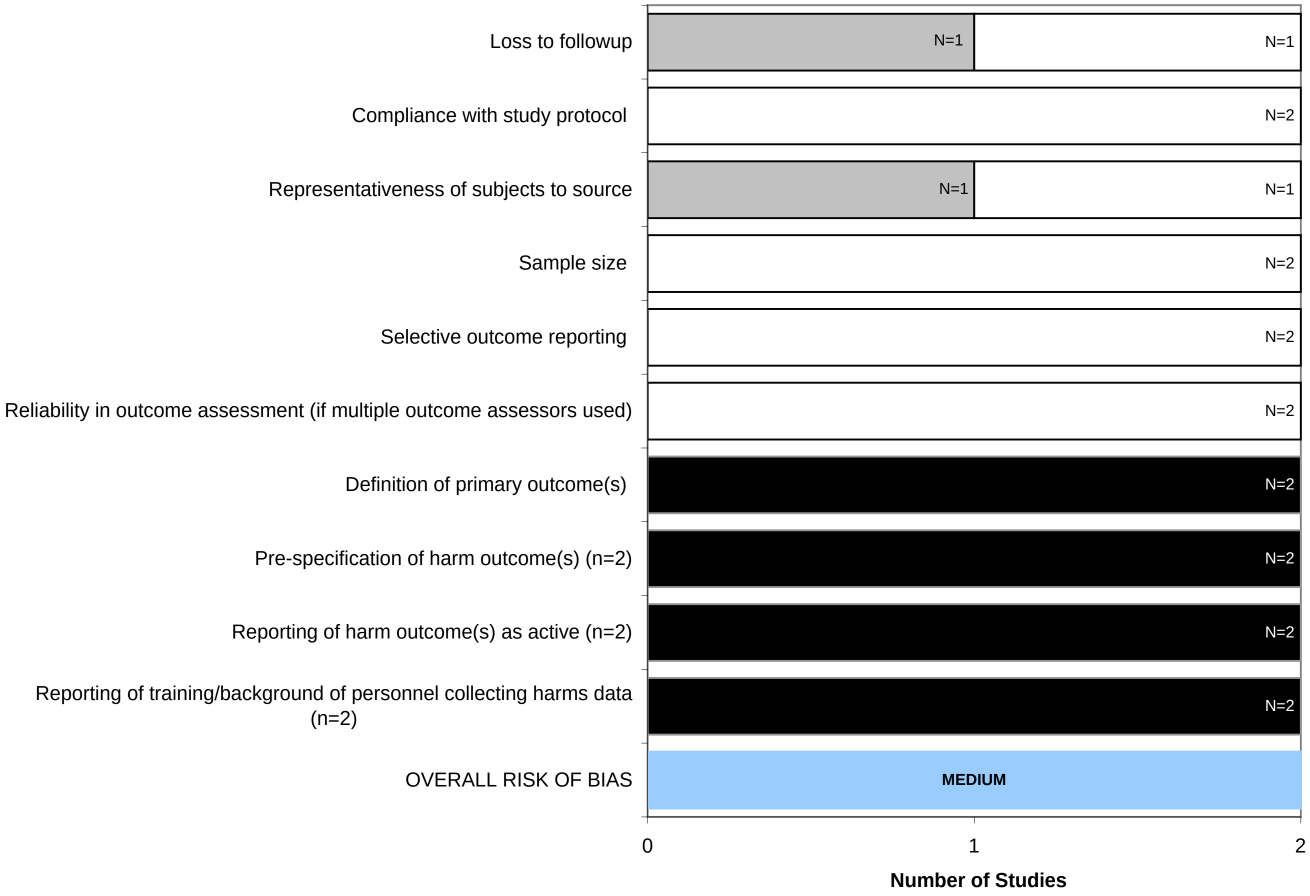

Nonrandomized Comparative Trials (n=1)

We rated the single nonrandomized trial as having high risk of bias for the outcomes birthweight and gestational age at delivery, and medium risk of bias for the outcome maternal hyperglycemia (Figure 5).[12] Allocation to intervention or comparator groups was based on primary tocolytic treatment. Participants who received < 24 hours of primary tocolysis were placed on oral terbutaline and participants who received > 24 hours of primary tocolysis, multiple courses of tocolysis, or multiple agents, were placed on terbutaline pump. This allocation scheme may have created prognostic differences among groups, which may have had an impact on the preterm birth outcomes (i.e., birth weight and gestational age at delivery). However, there is no clear indication that this would impact the outcome of maternal hyperglycemia.

Figure 5. Risk of bias assessment for nonrandomized trials (n=1)

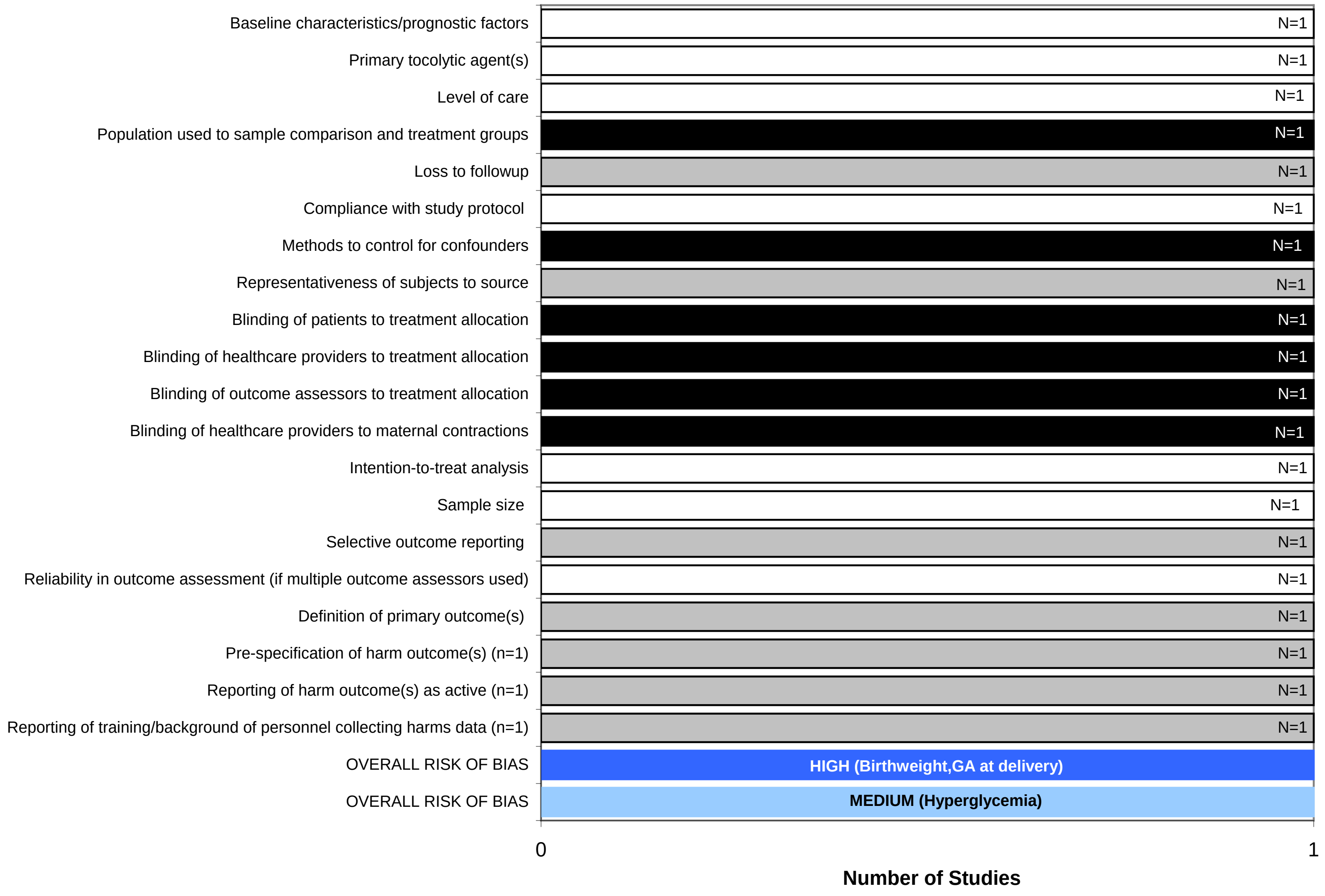

RCTs (n=3, Randomized Arms)

Figure 6 presents risk of bias assessments for RCTs.[10,11] One RCT had two comparators (placebo and oral terbutaline), and we assessed each randomized arm separately.[11] We rated both arms as high risk of bias because the study was likely underpowered, blinding was ineffective, and the participants who participated in the study represented a select group of patients because more than 90 percent of eligible participants declined to participate.[11] We rated the second RCT as low risk of bias because randomization was carried out properly and patients and health care providers were blinded.[10]

Figure 6. Risk of bias assessment for RCTs (n=3, randomized arms)

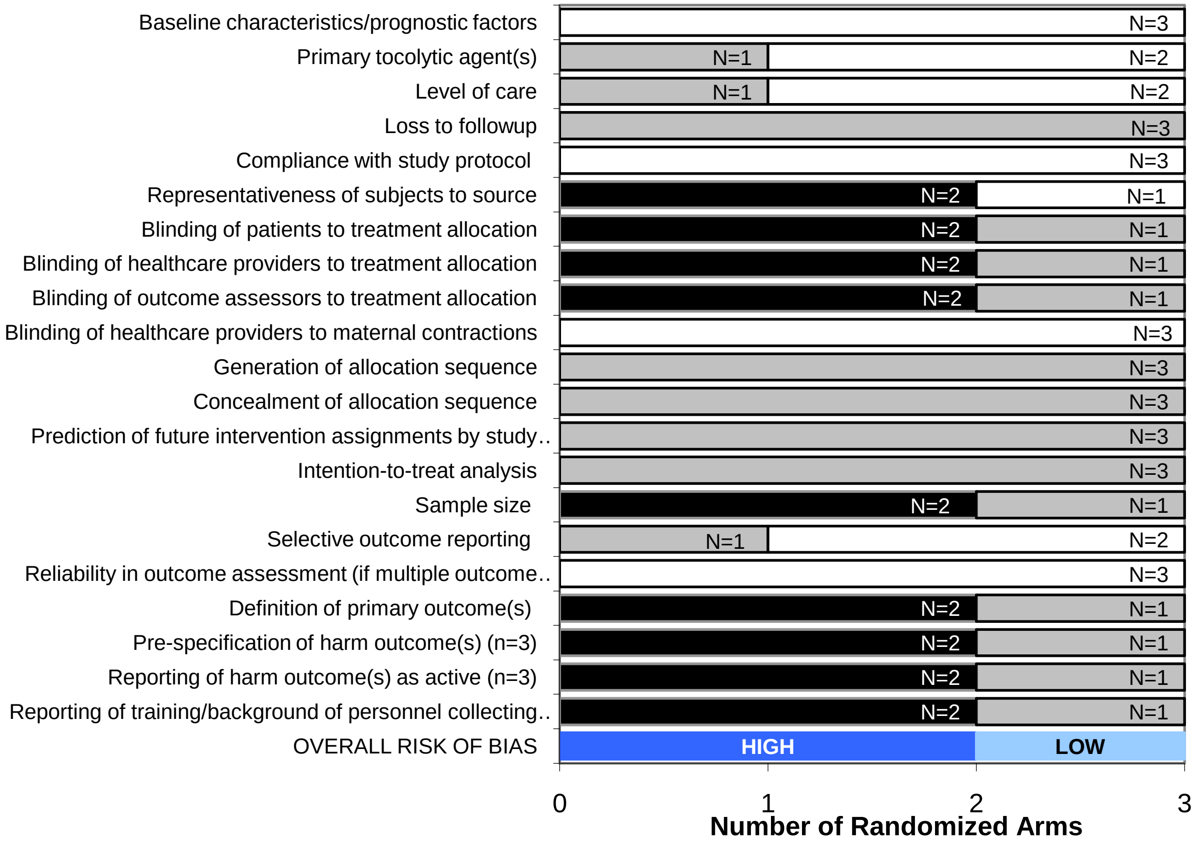

Sources of Funding

Most studies did not report sources of funding (Table 4). One RCT [10] and one case series [22] were industry funded.

Table 4. Sources of funding

Reference	Source
Flick (2010)[15]	Not reported
de la Torre (2008)[16]	Not reported
Fleming (2004)[17]	Not reported
Lam (2003)[18]	Not reported
Morrison (2003)[13]	Not reported
Lam (2001)[19]	Not reported
Guinn (1998)[10]	MiniMed Technologies (supported in part)
Wenstrom (1997)[11]	Not reported
Allbert (1994)[20]	Vicksburg Hospital Medical Foundation (supported in part)
Adkins (1993)[22]	PharmaThera Inc.
Regenstein (1993)[21]	National Institutes of Health Training
Lindenbaum (1992)[12]	Not reported
Morrison (1992)[14]	Vicksburg Hospital Medical Foundation (supported in part)
Lam (1988)[23]	Not reported

Key Question 1. Neonatal Health Outcomes

In women with arrested preterm labor, does treatment with a SQ infusion of terbutaline delivered by a pump, in comparison with placebo, conservative treatment or other interventions, improve neonatal health outcomes, including bronchopulmonary dysplasia, neonatal death, death within initial hospitalization, significant intraventricular hemorrhage (grade III/IV), necrotizing enterocolitis, periventricular leukomalacia, retinopathy of prematurity, seizures, sepsis, and stillbirth for the following subgroups:

a. Women <28 weeks, 0 days of gestation (extremely preterm)?
b. Women between 28 weeks, 0 days of gestation and 31 weeks, 6 days of gestation (very preterm)?
c. Women between 32 weeks, 0 days of gestation and 33 weeks, 6 days of gestation (preterm)?
d. Women between 34 weeks, 0 days of gestation and 36 weeks, 6 days of gestation (later preterm)?
e. Multiple gestation?
f. Racial or ethnic subgroups?
g. Women with previous preterm birth?
h. Women with history of preeclampsia?
i. Women with recurrent preterm labor (RPTL) and women without RPTL?

Key Points

- Strength of evidence was graded as insufficient for the outcomes of bronchopulmonary dysplasia, death within initial hospitalization, and significant intraventricular hemorrhage (grade III/IV) and for all populations of interest.
- For neonatal death, strength of evidence favoring SQ terbutaline pump over oral tocolytics is low for women with twin gestation and RPTL. For other populations, the evidence was graded as insufficient.
- Underpowered studies demonstrated indeterminate results for the outcomes of necrotizing enterocolitis, retinopathy of prematurity, sepsis, and stillbirth (i.e., type II error cannot be excluded).
- No data were available for periventricular leukomalacia and seizures.
- Data were unavailable for subgroups a–d and for subgroups f–h.

Detailed Analysis

Table F4 in Appendix F presents data for Key Question 1. None of the included studies reported data on bronchopulmonary dysplasia (strength of evidence is insufficient), death within initial hospitalization (strength of evidence is insufficient), periventricular leukomalacia, and seizures. Data could not be separated for women of specific gestational ages (subgroups a–d), racial or ethnic subgroups (subgroup f), women with previous preterm birth (subgroup g), and women with history of preeclampsia (subgroup h).

Below we report results by outcome, grade strength of evidence (for the following prespecified outcomes that were reported in studies: significant intraventricular hemorrhage and neonatal death), and summarize determinants of applicability where relevant. Results from Matria-based studies pertained to women who were inducted into a U.S. national database and who received specialized services from an outpatient perinatal program. We graded the strength of evidence for the specific populations of interest, as indicated in the key question. We also graded evidence from two RCTs that pertained to a nonspecific population of women with preterm labor; one RCT was in women with singleton gestation [10] and the other RCT was in women with either single or twin gestation (effect estimates were not presented separately by gestation).[11]

Summary tables are presented if more than one study was available for an outcome, otherwise all information has been summarized in the text.

Neonatal Death

Neonatal death was reported in three studies. Heterogeneity in study design, patient population, and comparator groups precluded evidence synthesis (Table 5). Either no or a sparse number of events were reported in all studies for followup to delivery.

In a retrospective cohort of women with singleton gestation and RPTL, no neonatal deaths were reported among the SQ terbutaline pump group or oral nifedipine comparator group.[17] Similarly, no neonatal deaths occurred in an RCT of women with singleton or twin gestation who received SQ terbutaline pump or placebo.[11] However, in a retrospective cohort of women with twin gestation and RPTL from the Matria database, Lam et al. demonstrated a statistically significant difference between SQ terbutaline pump and oral tocolytics, with fewer neonatal deaths occurring in the SQ terbutaline pump group (odds ratio [OR] = 0.09, 95% confidence interval [CI]: 0.01, 0.70).[19]

The overall strength of evidence in favor of SQ terbutaline pump for neonatal death was graded as low for twin gestation (subgroup e) and RPTL (subgroup i), based on the study by Lam et al. (Table 6).[19] Strength of evidence for all other populations was insufficient. Strength of evidence for neonatal death for a nonspecific preterm labor population described in an RCT is insufficient (Table 7).[11]

Table 5. Summary table for neonatal death

Study Design (Number of Studies)	Population	Comparator(s)	Risk of Bias	Overall Findings
Retrospective cohort (2)	Women with singleton gestation and RPTL from the Matria database (n=284)[17]	Oral nifedipine	Medium	No neonatal deaths were observed in two of the three studies.[11,17] One retrospective cohort of women with twin gestation and RPTL demonstrated a statistically significant difference in neonatal death, with fewer events in the SQ terbutaline pump group (OR=0.09, 95% CI: 0.01, 0.70).
	Women with twin gestation and RPTL from the Matria database (n=706)[19]	Oral tocolytics (92.3% received oral terbutaline)	Medium	
RCT (1)	Women with single or twin gestation from the University of Iowa Hospital (n=42)[11]	Placebo and oral terbutaline	High	

CI = confidence interval; OR = odds ratio; RCT = randomized controlled trial; RPTL = recurrent preterm labor; SQ = subcutaneous

Table 6. Neonatal death – Strength of evidence for populations of interest

Population	Number of Studies	Number of Participants	Number of Events	Strength of Evidence Domain: Risk of Bias	Strength of Evidence Domain: Consistency	Strength of Evidence Domain: Directness	Strength of Evidence Domain: Precision	OR (95% CI)	Strength of Evidence
Twin gestation + RPTL COHORT: SQ terbutaline pump vs. oral tocolytics[19]	1	706	12	Medium	N/A	Direct	Precise	0.09 (0.01, 0.70)	Low
Singleton gestation + RPTL COHORT: SQ terbutaline pump vs. oral nifedipine[17]	1	284	0	Medium	N/A	Direct	Imprecise	1.00 (0.02, 50.75)	Insufficient

CI = confidence interval; OR = odds ratio; RPTL = recurrent preterm labor; SQ = subcutaneous

Table 7. Neonatal death – Strength of evidence for nonspecific preterm labor populations

Population	Number of Studies	Number of Participants	Number of Events	Strength of Evidence Domain: Risk of Bias	Strength of Evidence Domain: Consistency	Strength of Evidence Domain: Directness	Strength of Evidence Domain: Precision	OR (95% CI)	Strength of Evidence
Singleton or twin gestation RCT: SQ terbutaline pump vs. placebo[11]	1	27	0	High	N/A	Direct	Imprecise	0.79 (0.01, 42.38)	Insufficient
Singleton or twin gestation RCT: SQ terbutaline pump vs. oral terbutaline[11]	1	30	0	High	N/A	Direct	Imprecise	0.85 (0.02, 45.00)	Insufficient

CI = confidence interval; OR = odds ratio; RCT = randomized controlled trial; SQ = subcutaneous

Significant Intraventricular Hemorrhage (Grade III/IV)

Two studies reported data on significant intraventricular hemorrhage (grade III/IV) (Table 8). These studies were not synthesized because of heterogeneous study designs, patient populations, and comparators. Both studies were underpowered to detect a difference due to sparse event rates and small sample sizes.

Morrison et al. compared SQ terbutaline pump with no treatment in a prospective cohort of women with singleton gestation and RPTL.[13] This study reported a nonsignificant difference (OR = 0.30, 95% CI: 0.02, 5.85).[13] In the second study, which did not pertain to any of the populations of interest, women with singleton gestation were randomly allocated to receive either SQ terbutaline pump or placebo; significant intraventricular hemorrhage was not observed in either group.[10]

We graded the overall strength of evidence as insufficient for women with RPTL (subgroup i) based on the prospective cohort study (Table 9). Strength of evidence for all other populations of interest was insufficient because no studies were available. Based on the RCT in a nonspecific preterm labor population, strength of evidence is insufficient (Table 10).[10]

Table 8. Summary table for significant intraventricular hemorrhage (grade III/IV)

Study Design (Number of Studies)	Population	Comparator(s)	Risk of Bias	Overall Findings
Prospective cohort (1)	Women with singleton gestation and RPTL (n=60)[13]	No treatment	High	No events were observed in the RCT. A statistically nonsignficant difference was observed in the prospective cohort (OR=0.30, 95% CI: 0.02, 5.85).
RCT (1)	Women with singleton gestation from Birmingham Hospital (n=52)[10]	Placebo	Low	

CI = confidence interval; OR = odds ratio; RCT = randomized controlled trial; RPTL = recurrent preterm labor

Table 9. Significant intraventricular hemorrhage (grade III/IV) – Strength of evidence for populations of interest

Population	Number of Studies	Number of Participants	Number of Events	Strength of Evidence Domain: Risk of Bias	Strength of Evidence Domain: Consistency	Strength of Evidence Domain: Directness	Strength of Evidence Domain: Precision	OR (95% CI)	Strength of Evidence
Singleton gestation + RPTL COHORT: SQ terbutaline pump vs. no treatment[13]	1	60	4	High	N/A	Direct	Imprecise	0.30 (0.02, 5.85)	Insufficient

CI = confidence interval; OR = odds ratio; RPTL = recurrent preterm labor; SQ = subcutaneous

Table 10. Significant intraventricular hemorrhage (grade III/IV) – Strength of evidence for nonspecific preterm labor populations

Population	Number of Studies	Number of Participants	Number of Events	Strength of Evidence Domain: Risk of Bias	Strength of Evidence Domain: Consistency	Strength of Evidence Domain: Directness	Strength of Evidence Domain: Precision	OR (95% CI)	Strength of Evidence
Singleton gestation RCT: SQ terbutaline pump vs. placebo[10]	1	52	0	Low	N/A	Direct	Imprecise	1.21 (0.02, 63.48)	Insufficient

CI = confidence interval; OR = odds ratio; RCT = randomized controlled trial; SQ = subcutaneous

Necrotizing Enterocolitis

Necrotizing enterocolitis was reported in a single prospective cohort that compared the SQ terbutaline pump with no treatment in women with singleton gestation and RPTL.[13] One case was reported in the no treatment comparator group, which resulted in a nonsignificant effect estimate (OR=0.96, 95% CI: 0.04, 24.74).[13] Given that there was only one event among a sample size of 60 participants, this study was clearly underpowered to detect a difference in this outcome. We rated this study as high risk of bias because intervention and comparator groups were imbalanced in risk factors for preterm birth, primary tocolytic therapy, and level of care.

Retinopathy of Prematurity and Sepsis

Retinopathy of prematurity and sepsis were reported in a single RCT of women with singleton or twin gestation who presented to a university hospital.[11] This study did not address any of the subgroups of interest. One infant in the SQ terbutaline pump group developed retinopathy of prematurity, and one infant in the oral terbutaline group developed sepsis.[11] Although both results were statistically nonsignificant, this study was underpowered to detect differences in either outcome. This study was rated as high risk of bias because of selection bias, limitations in study power, and absence of blinded outcome assessment.

Stillbirth

Three retrospective cohort studies reported data on stillbirth (Table 11).[17-19] These studies could not be meta-analyzed because sample populations and comparators were diverse and differences in confounders could not be excluded.

All three studies indicated statistically nonsignificant differences in the occurrence of stillbirth between the SQ terbutaline pump group and oral nifedipine or oral tocolytic comparator groups. Two of these studies were exclusively in women with singleton gestation [17,18] and one restricted to twins.[19] All studies were underpowered to detect a difference in this outcome because of low event rates (total number of events ranged from one to seven). Furthermore, there is potential for participant overlap among the SQ terbutaline pump groups of the two singleton studies.[17,18]

Table 11. Summary table for stillbirth

Study Design (Number of Studies)	Population	Comparator(s)	Risk of Bias	Overall Findings
Retrospective cohort (3)	Women with singleton gestation and RPTL from the Matria database (n=284)[17]	Oral nifedipine	Medium	All studies found statistically nonsignificant differences between SQ terbutaline pump and comparators. (OR=2.01, 95% CI: 0.18, 22.47; OR=3.01, 95% CI: 0.12, 74.23; OR=0.75, 95% CI: 0.17, 3.36). Participants in the SQ terbutaline pump groups of the singleton studies may have been double-counted.
	Women with singleton gestation and RPTL from the Matria database (n=558)[18]	Oral tocolytics (95.3% received oral terbutaline)	High	
	Women with twin gestation and RPTL from the Matria database (n=706)[19]	Oral tocolytics (92.3% received oral terbutaline)	Medium	

CI = confidence interval; OR = odds ratio; RPTL = recurrent preterm labor; SQ = subcutaneous

Key Question 2. Other Surrogate Outcomes

In women with arrested preterm labor, does treatment with a SQ infusion of terbutaline delivered by a pump, in comparison with placebo, conservative treatment or other interventions improve other surrogate outcomes, including mean gestational age at delivery, incidence of delivery at various gestational ages (<28 weeks, < 32 weeks, < 34 weeks, < 37 weeks), mean prolongation of pregnancy (days), birth weight, ratio of birth weight/gestational age at delivery, mean pregnancy prolongation index, need for assisted ventilation, need for oxygen per nasal cannula, and neonatal intensive care unit (NICU) admission, for the following subgroups:

a. Women <28 weeks, 0 days of gestation (extremely preterm)?
b. Women between 28 weeks, 0 days of gestation and 31 weeks, 6 days of gestation (very preterm)?
c. Women between 32 weeks, 0 days of gestation and 33 weeks, 6 days of gestation (preterm)?
d. Women between 34 weeks, 0 days of gestation and 36 weeks, 6 days of gestation (later preterm)?
e. Multiple gestation?
f. Racial or ethnic subgroups?
g. Women with previous preterm birth?
h. Women with history of preeclampsia?
i. Women with RPTL and women without RPTL?

Key Points

No data were available for the following outcomes: incidence of delivery < 28 weeks (strength of evidence is insufficient), need for oxygen per nasal cannula, or ratio of birth weight/gestational age at delivery.

Mean Gestational age at Delivery

Multiple Gestation

Two cohorts of medium risk of bias from the Matria database reported statistically significant differences in favor of the SQ terbutaline pump, compared with oral tocolytics. The risk of double-counting of participants could not be ruled out.

RPTL

Six cohorts of medium to high risk of bias, mostly from the Matria database, reported statistically significant differences in favor of SQ terbutaline pump compared with oral tocolytics or no treatment. Again, there is risk of double-counting of participants across these studies.

Overall Evidence

Two RCTs and two observational studies, which did not pertain to any population of interest, reported statistically nonsignificant differences between SQ terbutaline pump and placebo or oral tocolytics. This evidence contrasted with results from larger cohort studies, which demonstrated consistent benefit. The RCTs and other observational studies, which showed nonsignificant differences, may have been underpowered.

Incidence of Delivery at Various Gestational Ages

< 32 weeks

Strength of evidence favoring SQ terbutaline pump compared with oral tocolytics or no treatment for women with twin gestation or RPTL is low. This evidence came mostly from Matria-based studies.

< 34 weeks

Strength of evidence is insufficient for all populations of interest. One RCT of medium risk of bias, which did not pertain to any population of interest, reported a statistically nonsignificant difference compared with placebo, although type II error cannot be excluded.

< 37 weeks

Strength of evidence favoring SQ terbutaline pump compared with oral tocolytics or no treatment is insufficient or low for women with RPTL. This evidence came mostly from Matria-based studies.

Prolongation of Pregnancy

Strength of evidence favoring the SQ terbutaline pump compared with oral tocolytics or no treatment for women with twin gestation or RPTL was graded as insufficient or low for mean prolongation of pregnancy. This evidence came mostly from Matria-based studies.

Two retrospective cohorts of medium and high risk of bias, from the Matria database, reported pregnancy prolongation > 7 days in women with RPTL. One cohort reported a statistically significant difference in favor of the SQ terbutaline pump, compared with oral tocolytics. The other reported a nonsignificant difference, although type II error cannot be excluded.

Five retrospective cohorts of medium to high risk of bias, from the Matria database, reported pregnancy prolongation > 14 days in women with RPTL and single or twin gestation. All reported statistically significant differences in favor of SQ terbutaline pump, compared with oral tocolytics. Overlap in the study sample between these studies cannot be ruled out.

Mean Birth Weight

Multiple Gestation

Two cohorts of medium risk of bias from the Matria database reported statistically significant differences in favor of SQ terbutaline pump, compared with oral tocolytics. Overlap in the study sample between the two studies cannot be ruled out.

RPTL

Five of six cohorts, mostly from the Matria database and of medium to high risk of bias, reported statistically significant differences in favor of SQ terbutaline pump compared with oral tocolytics or no treatment. Overlap in the study sample between the Matria-based studies cannot be ruled out.

Overall evidence

Two RCTs, which did not pertain to any population of interest, reported statistically nonsignificant differences between SQ terbutaline pump and placebo. This result is indeterminate because of possible type II error. The RCT evidence contrasted with results from larger cohort studies, which demonstrated consistent benefit.

Incidence of low Birth Weight

Multiple Gestation

Two cohorts of medium risk of bias from the Matria database reported statistically significant differences in favor of SQ terbutaline pump, compared with oral tocolytics. Overlap in the study sample between the two studies cannot be ruled out.

RPTL

Six cohorts, mostly from the Matria database and of medium to high risk of bias, reported statistically significant differences in favor of SQ terbutaline pump compared with oral tocolytics or no treatment. Study sample may have overlapped among the Matria-based studies.

Incidence of Very low Birth Weight

Multiple Gestation

Two cohorts of medium risk of bias from the Matria database reported statistically significant differences in favor of SQ terbutaline pump, compared with oral tocolytics. Overlap in the study sample between the two studies cannot be ruled out.

RPTL

Three of four cohorts of medium to high risk of bias, from the Matria database, reported statistically significant differences in favor of SQ terbutaline pump compared with oral tocolytics. The study sample may have overlapped.

Pregnancy Prolongation Index

Two cohorts of medium and high risk of bias in women with RPTL reported statistically significant differences in favor of SQ terbutaline pump, compared with oral terbutaline or no treatment.

Need for Assisted Ventilation

One retrospective cohort of high risk of bias in women with singleton gestation and RPTL from the Matria database reported a nonsignificant difference in need for ventilator among infants with NICU admission.

Incidence of NICU Admission

Multiple Gestation

Two cohorts of medium risk of bias from the Matria database reported statistically significant differences in favor of SQ terbutaline pump, compared with oral tocolytics. Overlap in the study sample between the two studies cannot be ruled out.

RPTL

Six cohorts of medium to high risk of bias, mostly from the Matria database, reported statistically significant differences in favor of SQ terbutaline pump, compared with oral tocolytics or no treatment. Participant overlap among Matria-based studies cannot be ruled out.

NICU Mean Length of Stay

Multiple Gestation

One retrospective cohort of medium risk of bias, in women with twin gestation from the Matria database, reported a statistically significant difference in favor of SQ terbutaline pump compared with oral tocolytics.

RPTL

Four retrospective cohorts, mostly from the Matria database and primarily of high risk of bias, reported statistically significant differences in favor of SQ terbutaline pump, compared with oral tocolytics or no treatment. Participant overlap among Matria-based studies cannot be ruled out.

Detailed Analysis

Tables F5 to F9 in Appendix F present data extracted for Key Question 2. No data were available for incidence of delivery < 28 weeks (strength of evidence is insufficient), need for oxygen per nasal cannula, or ratio of birthweight/gestational age at delivery. Information was unavailable for women of specific gestational ages (subgroups a–d), racial or ethnic subgroups (subgroup f), women with previous preterm birth (subgroup g), or women with history of preeclampsia (subgroup h).

Results are presented below by outcome, along with strength of evidence grades for certain prespecified outcomes (i.e., incidence of delivery at various gestational ages and mean prolongation of pregnancy) and determinants of applicability. We graded the strength of evidence for the specific populations of interest, as indicated in the key question. We also graded evidence from two RCTs that pertained to a nonspecific population of women with preterm labor; one RCT was in women with singleton gestation[10] and the other RCT was in women with either single or twin gestation (effect estimates were not presented separately by gestation).[11]

If a single study was available on an outcome, then all information has been summarized in the text. Otherwise, we have summarized information in tables for each outcome according to the populations of interest. When we had information that did not pertain to any of the specific populations, we also summarized the population-specific and nonspecific data (which we have termed overall evidence) in tables and/or forest plots. We have presented forest plots to display the entire body of evidence for outcomes that had data from several studies.

The results from Matria-based studies pertained to women who were inducted into a U.S. national database and who received specialized services from an outpatient perinatal program. There is risk of double-counting of participants across some of the Matria-based studies.

Mean Gestational age at Delivery

Table F5 in Appendix F presents study-level data for mean gestational age at delivery. Eleven heterogeneous studies reported gestational age at delivery. Two were RCTs,[10,11] one was a nonrandomized trial,[12] two were prospective cohorts,[13,14] and the remaining were retrospective

cohorts.[15-19,21] Comparator groups included placebo,[10,11] no treatment,[13] and various oral tocolytic agents.[12,14-19,21] Data were available for women with twin gestation (subgroup e)[16,19] and women with RPTL (subgroup i)[13,15-19] The other studies did not explicitly address any of the populations of interest.[10-12,14,21]

Subgroup: Multiple Gestation

Two retrospective cohorts that used the Matria database were exclusively in women with twin gestation (Table 12).[16,19] Both studies demonstrated statistically significant differences in gestational age at delivery, with greater mean gestational age among women who received SQ terbutaline pump (difference in means = 0.70 weeks, 95% CI: 0.43 weeks, 0.97 weeks and 0.70 weeks, 95% CI: 0.48 weeks, 0.92 weeks).[16,19] Both studies were rated as medium risk of bias because several criteria, such as similarity in baseline characteristics and prognostic factors, could not be assessed due to incomplete reporting.

Table 12. Summary table for mean gestational age at delivery (subgroup: multiple gestation)

Study Design (Number of Studies)	Population	Comparator(s)	Risk of Bias	Overall Findings
Retrospective cohort (2)	Women with twin gestation and RPTL from the Matria database (n=656,)[16]	Oral nifedipine	Medium	Both studies demonstrated statistically significant differences in favor of SQ terbutaline pump.[16,19] Participants in the SQ terbutaline pump groups may have overlapped.
	Women with twin gestation and RPTL from the Matria database (n=706)[19]	Oral tocolytics (92.3% received oral terbutaline)	Medium	

RPTL = recurrent preterm labor ; SQ = subcutaneous

Subgroup: RPTL

One prospective cohort[13] and five retrospective cohorts specified RPTL as an inclusion criterion (Table 13).[15-19] Two of these studies were also in women with twin gestation, as described above.[16,19] The remaining four studies were in women with singleton gestation.

The prospective cohort reported a statistically significant difference in gestational age at delivery between the SQ terbutaline pump and no treatment, in favor of the pump (difference in means = 3.40 weeks, 95% CI: 1.80 weeks, 5.00 weeks).[13] However, this study was rated as high risk of bias because the groups were imbalanced in preterm birth risk factors, primary tocolytic therapy, and level of care. This study included women with singleton gestation only, the majority of whom were of African American origin.

All retrospective cohorts used the Matria database and reported statistically significant differences in favor of SQ terbutaline pump, compared with oral tocolytics (difference in means range = 0.70-0.90 weeks, 95% CI range: 0.28-0.48 weeks, 0.92-1.52 weeks).[15-19] Two studies were rated as high risk of bias because of group imbalances[15,18] and three were rated as medium risk of bias because the information presented in the reports was insufficient to assess several criteria, such as group comparability.[16,17,19]

Table 13. Summary table for mean gestational age at delivery (subgroup: RPTL)

Study Design (Number of Studies)	Population	Comparator(s)	Risk of Bias	Overall Findings
Prospective cohort (1)	Women with singleton gestation and RPTL (n=60)[13]	No treatment	High	All studies demonstrated statistically significant differences, in favor of SQ terbutaline pump. The possibility of participant overlap among the two Matria-based twin studies and among the three Matria-based singleton studies cannot be ruled out.
Retrospective cohort (5)	Women with singleton gestation and RPTL from the Matria database (n=1366)[15]	Oral nifedipine	High	
	Women with singleton gestation and RPTL from the Matria database (n=284) [17]	Oral nifedipine	Medium	
	Women with singleton gestation and RPTL from the Matria database (n=558)[18]	Oral tocolytics (95.3% received oral terbutaline)	High	
	Women with twin gestation and RPTL from the Matria database (n=656)[16]	Oral nifedipine	Medium	
	Women with twin gestation and RPTL from the Matria database (n=706)[19]	Oral tocolytics (92.3% received oral terbutaline)	Medium	

RPTL = recurrent preterm labor ; SQ = subcutaneous

Overall Evidence

Irrespective of patient populations and comparators, a total of 11 studies contributed evidence to the outcome of gestational age at delivery (Table 14). When compared with placebo, the RCT evidence for SQ terbutaline pump was indeterminate, given the small sample size (Figure 7).[10,11] Five larger observational cohort studies that used the Matria database and that were of medium to high risk of bias, showed consistent benefit with the pump in comparison with other tocolytics.[15-19]

Table 14. Summary table for mean gestational age at delivery (overall evidence)

Study Design (Number of Studies)	Population	Comparator(s)	Risk of Bias	Overall Findings
RCT (2)	Women with singleton gestation from Birmingham Hospital (n=52)[10]	Placebo	Low	A pooled estimate of the placebo arms of the two RCTs was nonsignificant. This result is indeterminate, given the small sample sizes of both studies. Five larger observational cohort studies of medium to high risk of bias showed a consistent benefit with the pump in comparison with other tocolytics.[15-19] However, there is a possibility that participants overlapped among some of these studies.<br><br>Other observational studies demonstrated nonsignificant differences.[14,21] The possibility of type II error cannot be excluded.
	Women with singleton or twin gestation from the University of Iowa Hospital (n=42)[11]	Placebo and oral terbutaline	High	
Nonrandomized trial (1)	Women with singleton gestation from the Hospital of the University of Pennsylvania (n=91)[12]	Oral terbutaline	High	
Prospective cohort (2)	Women with singleton gestation and RPTL (n=60)[13]	No treatment	High	
	Likely included a mixture of women with single and multiple gestation (n=69)[14]	Oral tocolytics	High	
Retrospective cohort (6)	Women with singleton gestation and RPTL from the Matria database (n=1366)[15]	Oral nifedipine	High	
	Women with singleton gestation and RPTL from the Matria database (n=284) [17]	Oral nifedipine	Medium	
	Women with singleton gestation and RPTL from the Matria database (n=558)[18]	Oral tocolytics (95.3% received oral terbutaline)	High	
	Women with twin gestation and RPTL from the Matria database (n=656)[16]	Oral nifedipine	Medium	
	Women with twin gestation and RPTL from the Matria database (n=706)[19]	Oral tocolytics (92.3% received oral terbutaline)	Medium	
	Likely included a mixture of women with single and multiple gestation (n=69)[21]	Oral terbutaline	High	

RCT = randomized controlled trial; RPTL = recurrent preterm labor

Figure 7. Mean gestational age (in weeks) at delivery (RCT pooled estimate: I^2 = 0.0 percent, p-value>0.05)

		Difference in means	Lower limit	Upper limit
OBSERVATIONAL	Flick (2010) (n=1366)	0.70	0.42	0.98
OBSERVATIONAL	de la Torre (2008) (n=656)	0.70	0.43	0.97
OBSERVATIONAL	Fleming (2004) (n=284)	0.90	0.28	1.52
OBSERVATIONAL	Lam (2003) (n=558)	0.80	0.39	1.21
OBSERVATIONAL	Morrison (2003) (n=60)	3.40	1.80	5.00
OBSERVATIONAL	Lam (2001) (n=706)	0.70	0.48	0.92
OBSERVATIONAL	Regenstein (1993) (n=69)	-1.40	-2.82	0.02
OBSERVATIONAL	Lindenbaum (1992) (n=91)*	-1.30	-1.83	-0.77
OBSERVATIONAL	Morrison (1992) (n=69)	0.40	-0.11	0.91
RCT:oral terbutaline	Wenstrom (1997) (n=30)	1.40	-0.92	3.72
RCT:placebo	Guinn (1998) (n=52)	-0.50	-2.57	1.57
RCT:placebo	Wenstrom (1997): saline (n=27)	0.30	-1.73	2.33
POOLED RCT:placebo		-0.09	-1.54	1.36

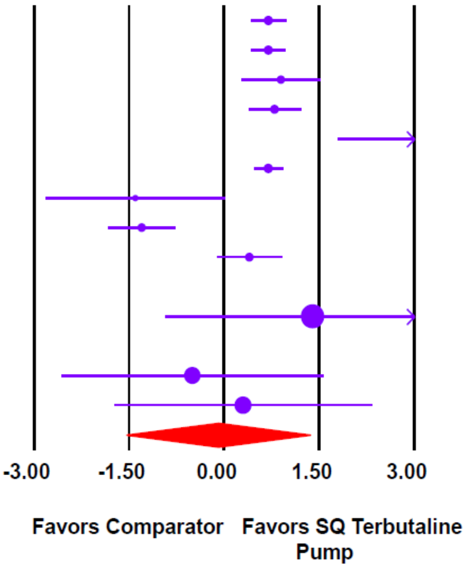

RCT = randomized controlled trial; SQ = subcutaneous
* Discrepancies were found in the information presented in the text and table of this paper. Mean gestational age at delivery for SQ terbutaline pump was reported as 36.6 weeks in table and 37.2 weeks in text. The value 36.6 weeks was used to calculate difference in means.

Incidence of Delivery at Various Gestational Ages

Study-level data for incidence of delivery at various gestational ages are presented in Table F6, Appendix F.

Incidence of Delivery < 32 Weeks' Gestation

Six cohort studies reported incidence of delivery at gestational age < 32 weeks' gestation. Data were available for women with multiple gestation[16,19] and women with RPTL.[13,15-19]

Subgroup: Multiple Gestation

Two retrospective cohorts included women with twin gestation only (Table 15).[16,19] Both studies reported a statistically significant difference in the incidence of delivery < 32 weeks, with fewer cases in the SQ terbutaline pump group compared with oral tocolytics (OR=0.47, 95% CI: 0.33, 0.68 and OR=0.52, 95% CI: 0.35, 0.76).[16,19] We rated both studies as medium risk of bias because incomplete reporting precluded assessment of several criteria, such as similarity in baseline characteristics and prognostic factors.

Based on these two studies, we graded the strength of evidence favoring the SQ terbutaline pump compared with oral tocolytics as low, for the population of women with twin gestation (Table 20). This evidence pertained to women with twin gestation and RPTL from the Matria database.

Table 15. Summary table for incidence of delivery < 32 weeks' gestation (subgroup: multiple gestation)

Study Design (Number of Studies)	Population	Comparator(s)	Risk of Bias	Overall Findings
Retrospective cohort (2)	Women with twin gestation and RPTL from the Matria database (n=656,)[16]	Oral nifedipine	Medium	Both studies demonstrated statistically significant differences in favor of SQ terbutaline pump.[16,19] Participants in the SQ terbutaline pump groups may have overlapped.
	Women with twin gestation and RPTL from the Matria database (n=706)[19]	Oral tocolytics (92.3% received oral terbutaline)	Medium	

RPTL = recurrent preterm labor; SQ = subcutaneous

Subgroup: RPTL

The entire body of evidence for this outcome pertained to women with RPTL (Table 16 and Figure 8). Four studies were in women with singleton gestation,[13,15,17,18] and two studies, which are described above, were in women with twin gestation.[16,19] These studies all found statistically significant differences in favor of the SQ terbutaline pump, compared with either no treatment or oral tocolytics.[13,15-19] Three studies were rated as high risk of bias due to group imbalances,[13,15,18] and three studies were rated as medium risk of bias due to incomplete reporting, which precluded an assessment of group comparability.[16,17,19]

Strength of evidence for different comparators and patient populations (i.e., singletons and twins) favoring the SQ terbutaline pump is low for women with RPTL (Table 17). Aside from the comparison against no treatment in women with singletons, the evidence for all other comparators and populations pertained to women from the Matria database. The study with the no treatment comparison group included women who were mostly of African American origin.[13]

Incidence of Delivery < 34 Weeks' Gestation

Strength of evidence was graded insufficient for all populations of interest. Only a single RCT, which compared the SQ terbutaline pump with placebo in women with singleton gestation, reported a nonsignificant difference in the incidence of delivery < 34 weeks (OR = 0.95, 95% CI: 0.32, 2.87).[10] Based on the small sample size, the possibility of type II error cannot be excluded. The strength of evidence for this nonspecific preterm labor population is insufficient (Table 18).

Table 16. Summary table for incidence of delivery < 32 weeks' gestation (subgroup: RPTL)

Study Design (Number of Studies)	Population	Comparator(s)	Risk of Bias	Overall Findings
Prospective cohort (1)	Women with singleton gestation and RPTL (n=60)[13]	No treatment	High	Multiple Gestation: Two retrospective studies in women with twins reported statistically significant differences, in favor of SQ terbutaline pump. Possibility of overlap in participants cannot be ruled out.<br><br>RPTL:<br>Entire body of evidence pertained to women with RPTL. All studies reported statistically significant differences, in favor of SQ terbutaline pump. Possibility of participant overlap among some studies cannot be ruled out.
Retrospective cohort (5)	Women with singleton gestation and RPTL from the Matria database (n=1366)[15]	Oral nifedipine	High	
	Women with singleton gestation and RPTL from the Matria database (n=284)[17]	Oral nifedipine	Medium	
	Women with singleton gestation and RPTL from the Matria database (n=558)[18]	Oral tocolytics (95.3% received oral terbutaline)	High	
	Women with twin gestation and RPTL from the Matria database (n=656)[16]	Oral nifedipine	Medium	
	Women with twin gestation and RPTL from the Matria database (n=706)[19]	Oral tocolytics (or 92.3% received oral terbutaline)	Medium	

RPTL = recurrent preterm labor; SQ = subcutaneous

Figure 8. Incidence of delivery < 32 weeks' gestation

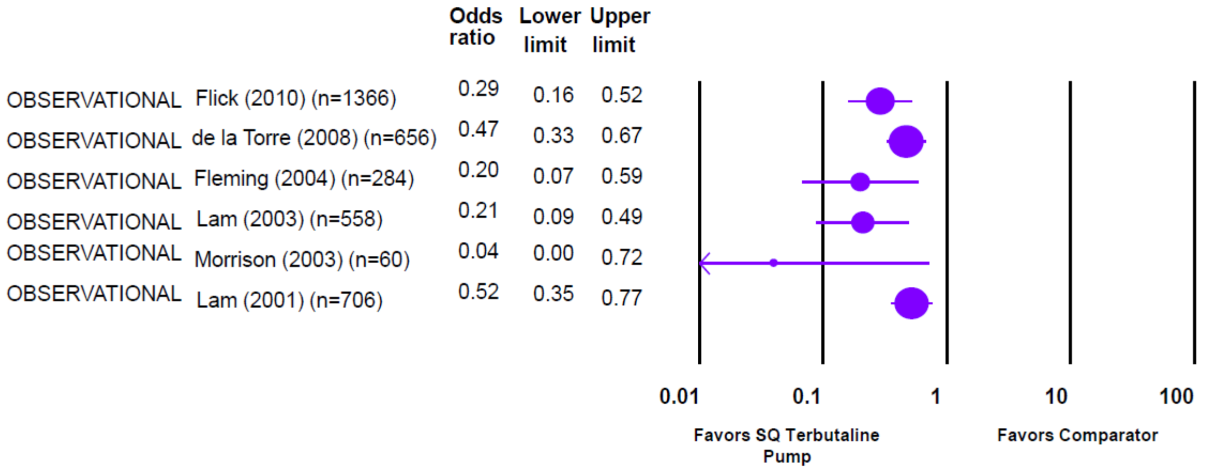

SQ = subcutaneous

Table 17. Incidence of delivery < 32 weeks' gestation – Strength of evidence for populations of interest

Population	N studies	N Participants	Number of Events	Strength of Evidence Domains: Risk of Bias	Consistency	Directness	Precision	OR (95% CI)	Strength of Evidence
Twin Gestation + RPTL									
COHORT: SQ terbutaline pump vs. oral nifedipine (twins)[16]	1	656	192	Medium	N/A	Indirect	Precise	0.47 (0.33, 0.68)	Low
COHORT: SQ terbutaline pump vs. oral tocolytics[19]	1	706	124	Medium	N/A	Indirect	Precise	0.52 (0.35, 0.76)	Low
Singleton Gestation + RPTL									
COHORT: SQ terbutaline pump vs. oral nifedipine[15,17]	2	1650	106	High/Medium	Consistent*	Indirect	Precise	0.20–0.29 (lower CI range 0.07-0.16, upper CI range 0.52-0.61)	Low
COHORT: SQ terbutaline pump vs. oral tocolytics [18]	1	558	37	High	N/A	Indirect	Precise	0.21 (0.09, 0.50)	Low
COHORT: SQ terbutaline pump vs. no treatment [13]	1	60	21	High	N/A	Indirect	Precise	0.04 (0.00, 0.65)	Low

CI = confidence interval; N/A = not applicable; OR = odds ratio; RPTL = recurrent preterm labor; SQ = subcutaneous

* Studies were not pooled. Also, there was risk of double-counting of participants across these studies.

Table 18. Incidence of delivery < 34 weeks' gestation – Strength of evidence for nonspecific preterm labor populations

Population	N studies	N Participants	Number of Events	Strength of Evidence Domains: Risk of Bias	Consistency	Directness	Precision	OR (95% CI)	Strength of Evidence
Singleton Gestation									
RCT: SQ terbutaline pump vs. placebo[10]	1	52	22	Low	N/A	Indirect	Imprecise	0.95 (0.32, 2.87)	Insufficient

CI = confidence interval; OR = odds ratio; RCT = randomized controlled trial; SQ = subcutaneous

Incidence of Delivery < 37 Weeks' Gestation

Six studies reported incidence of delivery < 37 weeks' gestation. Population-specific data were available only for women with RPTL.[13,15,17,18,20] One RCT, which did not pertain to any specific population of interest, randomized women with singleton gestation to SQ terbutaline pump or placebo and reported a nonsignificant difference (OR=1.57, 95% CI: 0.49, 5.02) (Figure 9).[10]

Subgroup: RPTL

Four retrospective cohorts and one prospective cohort reported incidence of delivery < 37 weeks in women with RPTL (Table 19).[13,15,17,18,20] Aside from one study, all included women with singleton gestation only.[13,15,17,18] One study likely consisted of women with single and multiple gestation.[20] Four of the five studies reported statistically significant differences in favor of SQ terbutaline pump, compared with oral tocolytics or no treatment (OR range = 0.04-0.72, 95% CI range: 0.01-0.58, 0.23-0.98).[13,15,18,20] These studies were of medium to high risk of bias.

Although Fleming et al. and Flick et al. both investigated women with singleton gestation from the Matria database and used the same comparator (i.e., oral nifedipine), Fleming et al. reported a nonsignificant difference (OR=0.75, 95% CI: 0.47, 1.20)[17] but Flick et al. reported a significant difference in favor of SQ terbutaline pump (OR=0.72, 95% CI: 0.58, 0.90).[15] The sample size in the Flick et al. study was much larger so, in the absence of any apparent clinical diversity, it appears that lower power in the Fleming et al. study may explain this difference.

Table 19. Summary table for incidence of delivery < 37 weeks' gestation (subgroup: RPTL)

Study Design (Number of Studies)	Population	Comparator(s)	Risk of Bias	Overall Findings
Prospective cohort (1)	Women with singleton gestation and RPTL (n=60)[13]	No treatment	High	Most studies reported statistically significant differences, in favor of SQ terbutaline pump. Participants may have overlapped among some of the Matria-based studies.
Retrospective cohort (4)	Women with singleton gestation and RPTL from the Matria database (n=1366)[15]	Oral nifedipine	High	
	Women with singleton gestation and RPTL from the Matria database (n=284)[17]	Oral nifedipine	Medium	
	Women with singleton gestation and RPTL from the Matria database (n=558)[18]	Oral tocolytics (95.3% received oral terbutaline)	High	
	Women with RPTL and likely included a mixture of women with single and multiple gestation (n=64)[20]	Oral terbutaline	Medium	

RCT = randomized controlled trial; RPTL = recurrent preterm labor; SQ = subcutaneous

For the population of women with RPTL, we graded the strength of evidence favoring SQ terbutaline pump compared with various comparators as insufficient or low (Table 20). The majority of this evidence was derived from the Matria database.[15,17,18] The study with the no treatment comparator group included women who were mostly of African American origin[13] and the study with oral terbutaline as a comparator group likely included women with single and multiple gestation, the majority of whom were classified as "nonwhite."[20] The strength of evidence for a nonspecific preterm labor population from the RCT is insufficient (Table 21).[10]

Figure 9. Incidence of delivery < 37 weeks' gestation

		Odds ratio	Lower limit	Upper limit
OBSERVATIONAL	Flick (2010) (n=1366)	0.72	0.58	0.90
OBSERVATIONAL	Fleming (2004) (n=284)	0.75	0.47	1.20
OBSERVATIONAL	Lam (2003) (n=558)	0.70	0.50	0.98
OBSERVATIONAL	Morrison (2003) (n=60)	0.04	0.01	0.24
OBSERVATIONAL	Allbert (1994) (n=64)	0.10	0.03	0.33
RCT	Guinn (1998) (n=52)	1.57	0.49	5.03

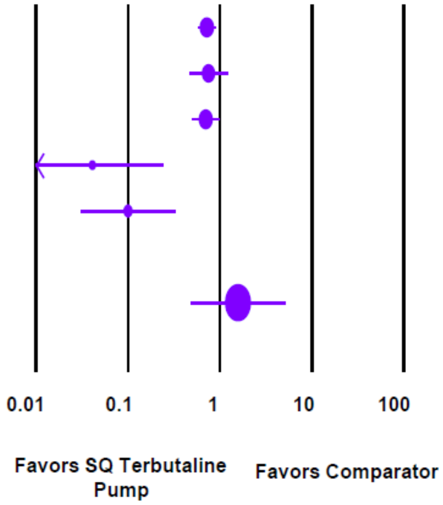

RCT = randomized controlled trial; SQ = subcutaneous

Table 20. Incidence of delivery < 37 weeks' gestation – Strength of evidence for populations of interest

Population	Number of Studies	Number of Participants	Number of Events	Strength of Evidence Domains: Risk of Bias	Strength of Evidence Domains: Consistency	Strength of Evidence Domains: Directness	Strength of Evidence Domains: Precision	OR (95% CI)	Strength of Evidence
Singleton Gestation + RPTL									
COHORT: SQ terbutaline pump vs. oral nifedipine[15,17]	2	1650	925	High/Medium	Consistent*	Indirect	Imprecise	0.72-0.75 (lower CI range 0.47-0.58, upper CI range 0.90-1.20)	Insufficient
COHORT: SQ terbutaline pump vs. oral tocolytics[18]	1	558	318	High	N/A	Indirect	Precise	0.70 (0.50, 0.98)	Low
COHORT: SQ terbutaline pump vs. no treatment[13]	1	60	50	High	N/A	Indirect	Precise	0.04 (0.01, 0.23)	Low
Singleton/Multiple Gestation + RPTL									
COHORT: SQ terbutaline pump vs. oral terbutaline[20]	1	64	38	Medium	N/A	Indirect	Precise	0.10 (0.03, 0.32)	Low

CI = confidence interval; OR = odds ratio; RPTL = recurrent preterm labor; SQ = subcutaneous

* Studies were not pooled. Also, there was risk of double-counting of participants across these studies.

Table 21. Incidence of delivery < 37 weeks' gestation – Strength of evidence for nonspecific preterm labor populations

Population	Number of Studies	Number of Participants	Number of Events	Strength of Evidence Domains: Risk of Bias	Strength of Evidence Domains: Consistency	Strength of Evidence Domains: Directness	Strength of Evidence Domains: Precision	OR (95% CI)	Strength of Evidence
Singleton Gestation									
RCT: SQ terbutaline pump vs. placebo[10]	1	52	34	Low	N/A	Indirect	Imprecise	1.57 (0.49, 5.02)	Insufficient

CI = confidence interval; OR = odds ratio; RCT = randomized controlled trial; SQ = subcutaneous

Prolongation of Pregnancy

Studies that reported prolongation of pregnancy are presented in Table F7, Appendix F. This outcome was reported either as a continuous variable (i.e., mean prolongation of pregnancy) or as a dichotomous variable (i.e., pregnancy prolongation > 7 days or > 14 days). In most studies, the prolongation of pregnancy interval was defined.[10,13,15-17,19] Population-specific data were available for women with multiple gestation and women with RPTL.

Mean Prolongation of Pregnancy

Seven studies reported mean prolongation of pregnancy. One observational study included women with twin gestation only[16] and five observational studies pertained to women with RPTL.[13,15-18] Two RCTs did not pertain to any populations of interest.[10,11]

Subgroup: Multiple Gestation

One retrospective cohort compared SQ terbutaline pump with oral nifedipine in women with twin gestation and RPTL from the Matria database.[16] Prolongation of pregnancy was measured from episode of RPTL to delivery. A statistically significant difference was observed in favor of the SQ terbutaline pump (difference in means in days: 7.20, 95% CI: 4.10, 10.30). We rated this study as medium risk of bias because there was insufficient information to assess several criteria, such as comparability of groups in baseline characteristics and prognostic factors.

The strength of evidence in favor of the SQ terbutaline pump compared with oral nifedipine for this specific population is low, based on this single study (Table 24).

Subgroup: RPTL

Five studies pertained to women with RPTL, including the one study in twins described above (Table 22 and Figure 10).[13,15-18] Statistically significant differences were reported by all studies, compared with either oral tocolytics or no treatment (difference in means in days ranged from 5.50-25.30, 95% CI range: 0.79-16.77, 8.72-33.83).[13,15-18] Three studies were rated as high risk of bias because groups were imbalanced in baseline characteristics and/or prognostic factors.[13,15,18] Two studies were rated as medium risk of bias because information presented in the report was insufficient to assess several criteria, such as group comparability.[16,17]

Strength of evidence favoring SQ terbutaline pump against various comparators for the populations of women with RPTL is insufficient or low (Table 24). The majority of evidence came from the Matria-based studies.[15-18] In the study with the no treatment comparison group, most women were of African American origin.[13] Strength of evidence for nonspecific preterm labor populations from RCTs is insufficient (Table 25).

Table 22. Summary table for mean prolongation of pregnancy (subgroup: RPTL)

Study Design (Number of Studies)	Population	Comparator(s)	Risk of Bias	Overall Findings
Prospective cohort (1)	Women with singleton gestation and RPTL (n=60)[13]	No treatment	High	All studies reported statistically significant differences, in favor of SQ terbutaline pump. Participants may have overlapped among some of these studies.
Retrospective cohort (4)	Women with singleton gestation and RPTL from the Matria database (n=1366)[15]	Oral nifedipine	High	
	Women with singleton gestation and RPTL from the Matria database (n=284)[17]	Oral nifedipine	Medium	
	Women with singleton gestation and RPTL from the Matria database (n=558)[18]	Oral tocolytics (95.3% received oral terbutaline)	High	
	Women with twin gestation and RPTL from the Matria database (n=656)[16]	Oral nifedipine	Medium	

RPTL = recurrent preterm labor; SQ = subcutaneous

Overall Evidence

Table 23 and Figure 10 present data for all studies that contributed to this outcome, regardless of study design and comparators. Evidence from RCTs showed indeterminate results (pooled mean difference=0.63, 95% CI: -9.6, 10.9) in contrast with evidence from observational studies of medium to high risk of bias, which showed consistent benefit. Plausible explanations include differences in study power (RCTs were underpowered compared with observational studies) and inherent risk of bias of observational study designs.

Table 23. Summary table for mean prolongation of pregnancy (overall evidence)

Study Design (Number of Studies)	Population	Comparator(s)	Risk of Bias	Overall Findings
RCT (2)	Women with singleton gestation from Birmingham Hospital (n=52)[10]	Placebo	Low	A pooled estimate of the placebo arms of the two RCTs was nonsignificant. This result contrasts with evidence from observational studies, which showed consistent benefit in favor of SQ terbutaline pump. However, the RCT evidence is indeterminate, given the small sample sizes of both studies.
	Women with singleton or twin gestation from the University of Iowa Hospital (n=42)[11]	Placebo and oral terbutaline	High	
Prospective cohort (1)	Women with singleton gestation and RPTL (n=60)[13]	No treatment	High	
Retrospective cohort (4)	Women with singleton gestation and RPTL from the Matria database (n=1366)[15]	Oral nifedipine	High	
	Women with singleton gestation and RPTL from the Matria database (n=284)[17]	Oral nifedipine	Medium	
	Women with singleton gestation and RPTL from the Matria database (n=558)[18]	Oral tocolytics (95.3% received oral terbutaline)	High	
	Women with twin gestation and RPTL from the Matria database (n=656)[16]	Oral nifedipine	Medium	

RCT = randomized controlled trial; RPTL = recurrent preterm labor; SQ = subcutaneous

Figure 10. Mean prolongation of pregnancy (RCT pooled estimate: I^2 = 0.0 percent, p-value > 0.05)

		Difference in means	Lower limit	Upper limit
OBSERVATIONAL	Flick (2010) (n=1366)	7.50	4.94	10.06
OBSERVATIONAL	de la Torre (2008) (n=656)	7.20	4.10	10.30
OBSERVATIONAL	Fleming (2004) (n=284)	6.20	0.79	11.61
OBSERVATIONAL	Lam (2003) (n=558)	5.50	2.28	8.72
OBSERVATIONAL	Morrison (2003) (n=60)	25.30	16.77	33.83
RCT:oral terbutaline	Wenstrom (1997) (n=30)	5.60	-14.45	25.65
RCT:placebo	Guinn (1998) (n=52)	0.90	-11.36	13.16
RCT:placebo	Wenstrom (1997) (n=27)	0.00	-18.53	18.53
POOLED RCT:placebo		0.63	-9.60	10.85

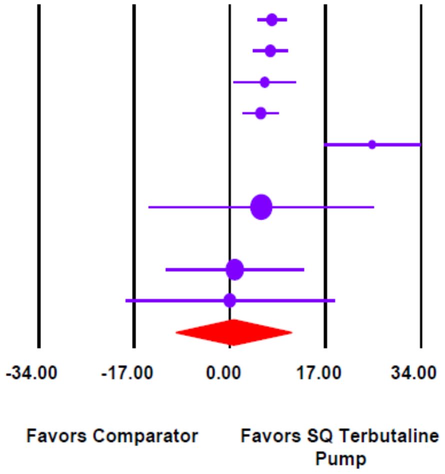

RCT = randomized controlled trial; SQ = subcutaneous

Table 24. Mean prolongation of pregnancy – Strength of evidence for populations of interest

Population	$N_{studies}$	$N_{Participants}$	Strength of Evidence Domains				MD (95% CI) (days)	Strength of Evidence
			Risk of Bias	Consistency	Directness	Precision		
Twin Gestation + RPTL								
COHORT: SQ terbutaline pump vs. oral nifedipine[16]	1	656	Medium	N/A	Indirect	Precise	7.20 (4.10, 10.30)	Low
Singleton Gestation + RPTL								
COHORT: SQ terbutaline pump vs. oral nifedipine[15,17]	2	1650	High/Medium	Consistent*	Indirect	Imprecise	6.20-7.50 (lower CI range 0.79-4.94, upper CI range 10.06-11.61	Insufficient
COHORT: SQ terbutaline pump vs. oral tocolytics[18]	1	558	High	N/A	Indirect	Precise	5.50 (2.28, 8.72	Low
COHORT: SQ terbutaline pump vs. no treatment[13]	1	60	High	N/A	Indirect	Precise	25.30 (16.77, 33.83)	Low

CI = confidence interval; MD = mean difference; RPTL = recurrent preterm labor; SQ = subcutaneous
* Studies were not pooled. Also, there was risk of double-counting of participants across these studies

Table 25. Mean prolongation of pregnancy – Strength of evidence for nonspecific preterm labor populations

Population	$N_{studies}$	$N_{Participants}$	Strength of Evidence Domains				MD (95% CI) (days)	Strength of Evidence
			Risk of Bias	Consistency	Directness	Precision		
Singelton or Twin Gestation								
RCT: SQ terbutaline pump vs. placebo[10,11]	2	79	Low/High	Consistent	Indirect	Imprecise	0.63 (-9.60,10.85)	Insufficient
RCT: SQ terbutaline pump vs. oral terbutaline[11]	1	30	High	N/A	Indirect	Imprecise	5.60 (-14.45, 25.65)	Insufficient

CI = confidence interval; MD = mean difference; RCT = randomized controlled trial; SQ = subcutaneous

Pregnancy Prolongation > 7 Days

Two observational studies reported pregnancy prolongation > 7 days (Table 26).[15,17] Both included women with singleton gestation and RPTL from the Matria database and had oral nifedipine as a comparator. Flick et al. found that significantly more participants in the SQ terbutaline pump group had pregnancy prolonged for more than 7 days compared with the group that received oral nifedipine (OR=7.84, 95% CI: 3.59, 17.12; total number of events/sample size=1281/1366). We rated this study as high risk of bias because of group imbalances. Fleming et al., however, reported a nonsignificant difference between SQ terbutaline pump and oral nifedipine (OR=2.53, 95% CI: 0.87, 7.38; total number of events/sample size = 267/284).[17] Given that the study by Flick et al. had a much larger sample size and reported a significant result, the nonsignificant difference reported by Fleming et al. may have been due to low power. We rated the study by Fleming et al. as medium risk of bias because there was insufficient information to assess several criteria, such as group comparability.

Table 26. Summary table for pregnancy prolongation > 7 days (overall evidence)

Study Design (Number of Studies)	Population	Comparator(s)	Risk of Bias	Overall Findings
Retrospective cohort (2)	Women with singleton gestation and RPTL from the Matria database (n=1366)[15]	Oral nifedipine	High	One study reported a statistically significant difference, in favor of SQ terbutaline pump[15] and the other study reported a nonsignificant difference.[17] The latter study may have been underpowered.
	Women with singleton gestation and RPTL from the Matria database (n=284)[17]	Oral nifedipine	Medium	

RPTL = recurrent preterm labor; SQ = subcutaneous

Pregnancy Prolongation > 14 Days

Five retrospective cohorts reported pregnancy prolongation > 14 days. All of these studies used the Matria database and were exclusively in women with RPTL.

Subgroup: Multiple Gestation

Two studies were in women with twin gestation and both reported statistically significant differences in favor of SQ terbutaline pump, compared with oral tocolytics (OR=2.48, 95% CI: 1.65, 3.73; total number of events/sample size = 488/656 and OR=1.93, 95% CI: 1.40, 2.65; total number of events/sample size=469/706) (Table 27).[16,19] We rated these studies as medium risk of bias because there was insufficient information to assess several criteria, such as group comparability.

Table 27. Summary table for pregnancy prolongation > 14 days (subgroup: multiple gestation)

Study Design (Number of Studies)	Population	Comparator(s)	Risk of Bias	Overall Findings
Retrospective cohort (2)	Women with twin gestation and RPTL from the Matria database (n=656,)[16]	Oral nifedipine	Medium	Both studies demonstrated statistically significant differences in favor of the SQ terbutaline pump.[16,19] The possibility that patients on the SQ terbutaline pump overlapped between the studies cannot be ruled out.
	Women with twin gestation and RPTL from the Matria database (n=706)[19]	Oral tocolytics (92.3% received oral terbutaline)	Medium	

RPTL = recurrent preterm labor; SQ = subcutaneous

Subgroup: RPTL

All studies were in women with RPTL, of either single or twin gestation (the studies in women with twin gestation have been described above) (Table 28 and Figure 11). Consistent, statistically significant differences in favor of SQ terbutaline pump compared with oral tocolytics were found across all studies (OR range=1.93-3.47, 95% CI range: 0.87-2.34, 2.65-5.15) (Figure 11).[15-18] Two studies were rated as high risk of bias because of differences in baseline characteristics/prognostic factors among groups [15,18] and three were rated as medium risk of bias because there was insufficient information to assess several criteria, such as group comparability.[16,17,19]

Table 28. Summary table for pregnancy prolongation > 14 days (subgroup: RPTL)

Study Design (Number of Studies)	Population	Comparator(s)	Risk of Bias	Overall Findings
Retrospective cohort (5)	Women with singleton gestation and RPTL from the Matria database (n=1366)[15]	Oral nifedipine	High	All studies pertained to women with RPTL. Two of these studies were in women with twin gestation and the rest were in singletons. All studies reported statistically significant differences, in favor of the SQ terbutaline pump. Participants may have overlapped among some studies.
	Women with singleton gestation and RPTL from the Matria database (n=284)[17]	Oral nifedipine	Medium	
	Women with singleton gestation and RPTL from the Matria database (n=558)[18]	Oral tocolytics (95.3% received oral terbutaline)[18]	High	
	Women with twin gestation and RPTL from the Matria database (n=656)[16]	Oral nifedipine	Medium	
	Women with twin gestation and RPTL from the Matria database (n=706)[19]	Oral tocolytics (92.3% received oral terbutaline)	Medium	

RPTL = recurrent preterm labor; SQ = subcutaneous

Figure 11. Pregnancy Prolongation > 14 Days

		Odds ratio	Lower limit	Upper limit
OBSERVATIONAL	Flick (2010) (n=1366)	3.47	2.34	5.15
OBSERVATIONAL	de la Torre (2008) (n=656)	2.48	1.65	3.73
OBSERVATIONAL	Fleming (2004) (n=284)	2.82	1.30	6.12
OBSERVATIONAL	Lam (2003) (n=558)	2.40	1.57	3.67
OBSERVATIONAL	Lam (2001) (n=706)	1.93	1.40	2.66

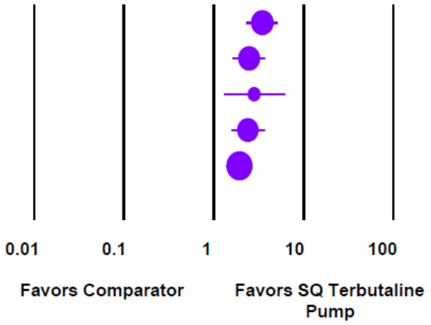

SQ = subcutaneous

Birth Weight

Birth weight was reported as either a continuous or dichotomous variable (i.e., incidence of low birth weight and very low birth weight) in seven observational studies, one nonrandomized trial, and two RCTs. Observational studies reported birthweight for women with twin gestation[16,19] and RPTL.[13,15-20] Four studies did not pertain to any specific population of interest.[10-12,21] Study-level data is presented in Table F8 in Appendix F.

Mean Birth Weight

Subgroup: Multiple Gestation

As shown in Table 29, two retrospective cohort studies that compared the SQ terbutaline pump with oral tocolytics in women with twin gestation and RPTL from the Matria database reported statistically higher birth weights among infants of the SQ terbutaline pump group (mean differences in grams = 163, 95% CI: 102, 224 and 136, 95% CI 83, 189).[16,19] Both studies were rated as medium risk of bias because several criteria, such as similarity in baseline characteristics and prognostic factors, could not be assessed due to incomplete reporting.

Table 29. Summary table for mean birth weight (subgroup: multiple gestation)

Study Design (Number of Studies)	Population	Comparator(s)	Risk of Bias	Overall Findings
Retrospective cohort (2)	Women with twin gestation and RPTL from the Matria database (n=656,)[16]	Oral nifedipine	Medium	Both studies reported statistically significant differences, in favor of SQ terbutaline pump. Study populations may have overlapped.
	Women with twin gestation and RPTL from the Matria database (n=706)[19]	Oral tocolytics (92.3% received oral terbutaline)	Medium	

RPTL = recurrent preterm labor; SQ = subcutaneous

Subgroup: RPTL

Table 30 presents information on studies that reported birth weight in women with RPTL. Two of these studies were in women with twin gestation, as described above.[16,19] Aside from one study that reported a nonsignificant result among a study population that likely consisted of women with single and multiple gestation,[20] all demonstrated statistically significant differences in favor of SQ terbutaline pump, compared with either oral tocolytics or no treatment (range of mean difference in grams was 136–721, 95% CI range: 83–355, 189–1087).[13,16-19] Two studies in this body of evidence were rated as high risk of bias because of apparent differences in groups[13,18] and the remaining four studies were rated as medium risk of bias because missing information prevented adequate assessment of potential limitations.[16,17,19,20] The majority of this evidence came from the Matria database.[16-19] The study with the no treatment comparator group included women who were mostly of African American origin[13] and the study with oral terbutaline as a comparator group likely included women with single and multiple gestation, the majority of whom were classified as "nonwhite."[20]

Table 30. Summary table for mean birth weight (subgroup: RPTL)

Study Design (Number of Studies)	Population	Comparator(s)	Risk of Bias	Overall Findings
Prospective cohort (1)	Women with singleton gestation and RPTL (n=60)[13]	No treatment	High	Aside from one study that included a mixture of women with single and multiple gestation,[20] all reported statistically significant differences, in favor of the SQ terbutaline pump. Participants may have overlapped among some of the Matria-based studies.
Retrospective cohort (5)	Women with singleton gestation and RPTL from the Matria database (n=284) [17]	Oral nifedipine	Medium	
	Women with singleton gestation and RPTL from the Matria database (n=558)[18]	Oral tocolytics (95.3% received oral terbutaline)	High	
	Women with twin gestation and RPTL from the Matria database (n=656)[16]	Oral nifedipine	Medium	
	Women with twin gestation and RPTL from the Matria database (n=706)[19]	Oral tocolytics (92.3% received oral terbutaline)	Medium	
	Women with RPTL and likely included a mixture of women with single and multiple gestation (n=64)[20]	Oral terbutaline	Medium	

RPTL = recurrent preterm labor; SQ = subcutaneous

Overall Evidence

Table 31 and Figure 12 present the entire body of evidence for mean difference in birthweight, regardless of subgroups. The study by Lindenbaum et al. reported discrepant results between table and text and therefore, the study estimate was deemed unreliable.[12] The pooled evidence from RCTs was inconclusive (difference in means = 121.75, 95% CI: -183.55, 427.05), but type II error cannot be ruled out (Figure 12). In contrast, evidence from larger observational studies of medium to high risk of bias showed statistically higher birth weights for women receiving the SQ terbutaline pump compared with oral tocolytics.

Table 31. Summary table for mean birth weight (overall evidence)

Study Design (Number of Studies)	Population	Comparator(s)	Risk of Bias	Overall Findings
RCT (2)	Women with singleton gestation from Birmingham Hospital (n=52)[10]	Placebo	Low	A pooled estimate of the placebo arms of the two RCTs was nonsignificant. This result is indeterminate, given the small sample sizes of both studies. Larger observational cohort studies of medium to high risk of bias showed a consistent benefit with the pump in comparison with other tocolytics.[16-19] However, participants may have overlapped among some of these studies. Other observational studies demonstrated a nonsignificant difference[20] or a statistically significant difference in favor of comparator.[21]
	Women with singleton or twin gestation from the University of Iowa Hospital (n=42)[11]	Placebo and oral terbutaline	High	
Nonrandomized trial (1)	Women with singleton gestation from the Hospital of the University of Pennsylvania (n=91)[12]	Oral terbutaline	High	
Prospective cohort (1)	Women with singleton gestation and RPTL (n=60)[13]	No treatment	High	
Retrospective cohort (6)	Women with singleton gestation and RPTL from the Matria database (n=284) [17]	Oral nifedipine	Medium	
	Women with singleton gestation and RPTL from the Matria database (n=558)[18]	Oral tocolytics (95.3% received oral terbutaline)	High	
	Women with twin gestation and RPTL from the Matria database (n=656)[16]	Oral nifedipine	Medium	
	Women with twin gestation and RPTL from the Matria database (n=706)[19]	Oral tocolytics (92.3% received oral terbutaline)	Medium	
	Women with RPTL and likely included a mixture of women with single and multiple gestation (n=64)[20]	Oral terbutaline	Medium	
	Women with single or multiple gestation (n=69)[21]	Oral terbutaline	High	

RCT = randomized controlled trial; RPTL = recurrent preterm labor

Figure 12. Mean birth weight (RCT Pooled Estimate: I^2 = 0.0 percent, p-value > 0.05)

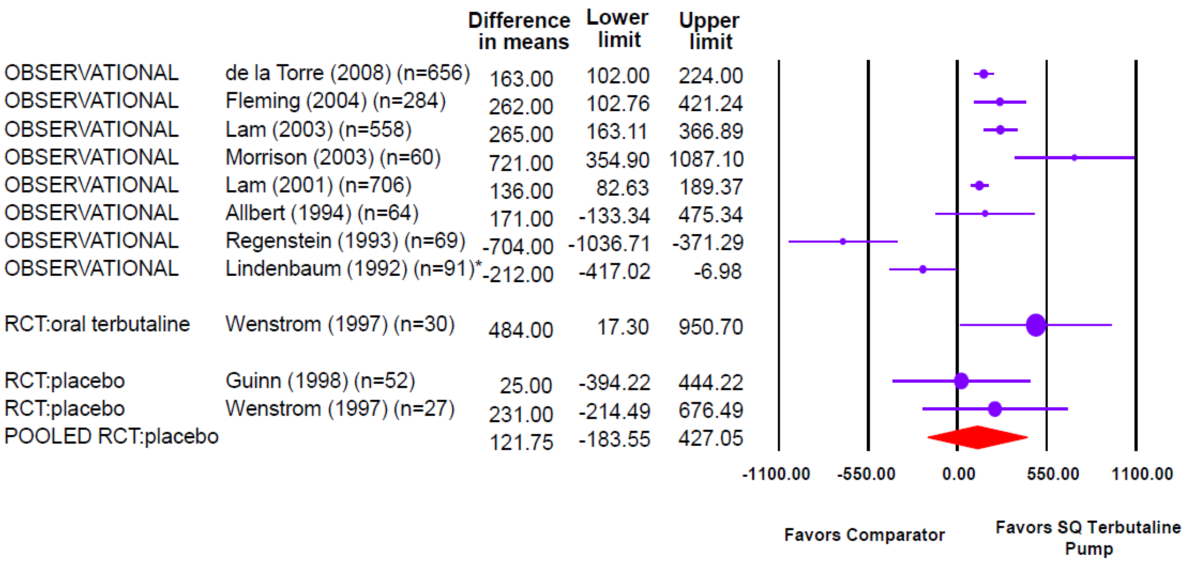

RCT = randomized controlled trial; SQ = subcutaneous
*Discrepancies were found in the information presented in the text and table of this paper. The numbers reported in the table were used to calculate difference in means. However, the text reported groups with the reverse numbers (i.e., SQ terbutaline pump: 3229 ± 584 and oral terbutaline: 3017 ± 303).

Incidence of low Birth Weight

Tables 32 and 33 and Figure 13 below present studies that reported low birth weight, which was defined as < 2,500 g. All studies were observational in design.

Subgroup: Multiple Gestation

Two studies were in women with twin gestation and RPTL from the Matria database (Table 32). Both reported statistically significant results in favor of SQ terbutaline pump compared with oral tocolytics (OR = 0.57, 95% CI: 0.44, 0.73; total number of events/number of infants = 975/1312 and OR = 0.64, 95% CI: 0.51, 0.80; total number of events/number of infants = 926/1393).[16,19] These studies were rated as medium risk of bias because there was insufficient information to assess potential limitations.

Table 32. Summary table for incidence of low birth weight (subgroup: multiple gestation)

Study Design (Number of Studies)	Population	Comparator(s)	Risk of Bias	Overall Findings
Retrospective cohort (2)	Women with twin gestation and RPTL from the Matria database (n=656,)[16]	Oral nifedipine	Medium	Both studies reported statistically significant differences, in favor of the SQ terbutaline pump. Study populations may have overlapped.
	Women with twin gestation and RPTL from the Matria database (n=706)[19]	Oral tocolytics (92.3% received oral terbutaline)	Medium	

RPTL = recurrent preterm labor; SQ = subcutaneous

Subgroup: RPTL

All studies pertained to women with RPTL (Table 33). Five of these studies were from the Matria database, so there may have been overlap in participant data.[15-19] Statistically significant differences were found across all studies of medium to high risk of bias in favor of the SQ terbutaline pump, compared with oral tocolytics or no treatment (OR range = 0.24-0.64, 95% CI range: 0.06-0.51, 0.62-0.96). The majority of women in the prospective cohort with the no treatment comparator group were of African American origin.[13]

Table 33. Summary table for incidence of low birth weight (subgroup: RPTL)

Study Design (Number of Studies)	Population	Comparator(s)	Risk of Bias	Overall Findings
Prospective cohort (1)	Women with singleton gestation and RPTL (n=60)[13]	No treatment	High	Statistically significant differences in favor of the pump were found in all studies. The five retrospective cohorts recruited women from the Matria database, so participants may have overlapped in some of these studies.
Retrospective cohort (5)	Women with singleton gestation and RPTL from the Matria database (n=1366)[15]	Oral nifedipine	High	
	Women with singleton gestation and RPTL from the Matria database (n=284)[17]	Oral nifedipine	Medium	
	Women with singleton gestation and RPTL from the Matria database (n=558)[18]	Oral tocolytics (95.3% received oral terbutaline)	High	
	Women with twin gestation and RPTL from the Matria database (n=656)[16]	Oral nifedipine	Medium	
	Women with twin gestation and RPTL from the Matria database (n=706)[19]	Oral tocolytics (92.3% received oral terbutaline)	Medium	

RPTL = recurrent preterm labor; SQ = subcutaneous

Figure 13. Incidence of low birth weight

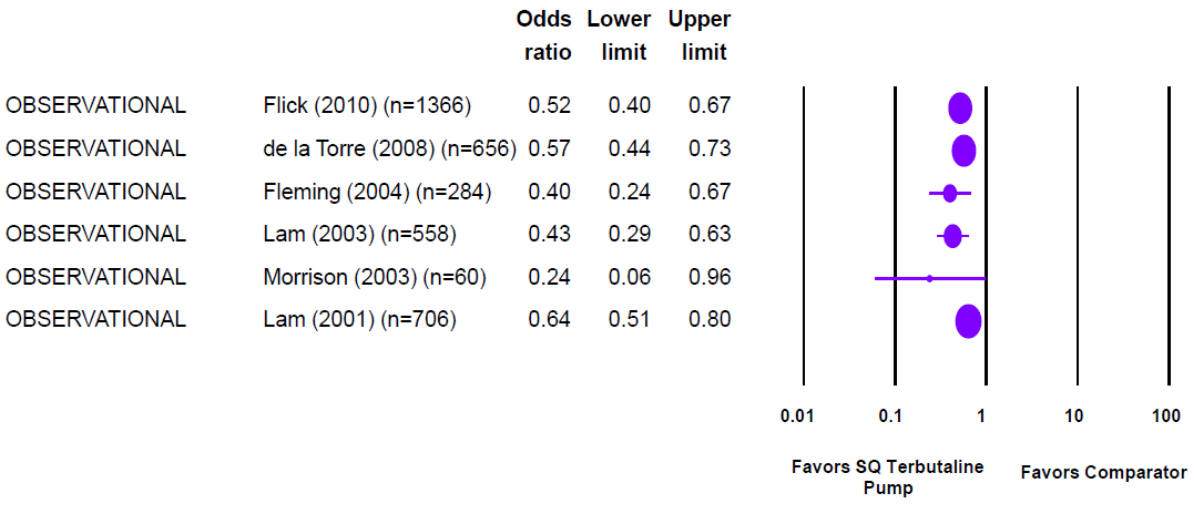

SQ = subcutaneous

Incidence of Very low Birth Weight

Tables 34 and 35 and Figure 14 below present studies that reported incidence of very low birthweight, which was defined as < 1,500 g.

Subgroup: Multiple Gestation

Two of these studies were in women with twin gestation and RPTL from the Matria database (Table 34). Both reported statistically significant results in favor of SQ terbutaline pump compared with oral tocolytics (OR = 0.40, 95% CI: 0.26, 0.60; total number of events/number of infants = 156/1312 and OR=0.46, 95% CI: 0.29, 0.73; total number of events/sample size = 88/1393) (Table 32).[16,19] These studies were rated as medium risk of bias because there was insufficient information to assess potential limitations.

Table 34. Summary table for incidence of very low birth weight (subgroup: multiple gestation)

Study Design (Number of Studies)	Population	Comparator(s)	Risk of Bias	Overall Findings
Retrospective cohort (2)	Women with twin gestation and RPTL from the Matria database (n=656,)[16]	Oral nifedipine	Medium	Both studies reported statistically significant differences, in favor of SQ terbutaline pump. Study populations may have overlapped.
	Women with twin gestation and RPTL from the Matria database (n=706)[19]	Oral tocolytics (92.3% received oral terbutaline)	Medium	

RPTL = recurrent preterm labor; SQ = subcutaneous

Subgroup: RPTL

All studies pertained to women with RPTL (Table 35).[16-19] Aside from the study by Fleming et al, which showed a nonsignificant result,[17] statistically significant differences were found across all studies of medium to high risk of bias in favor of SQ terbutaline pump, compared with oral tocolytics (OR range = 0.22–0.46, 95% CI range: 0.07–0.29, 0.60–0.73).[16,18,19] No apparent explanations for this inconsistency in effect estimates could be found. However, the Fleming et al. study had the smallest sample size and, therefore, it may have been underpowered to detect a difference in this outcome.

Table 35. Summary table for incidence of very low birth weight (subgroup: RPTL)

Study Design (Number of Studies)	Population	Comparator(s)	Risk of Bias	Overall Findings
Retrospective cohort (4)	Women with singleton gestation and RPTL from the Matria database (n=284) [17]	Oral nifedipine	Medium	All studies pertained to women with RPTL and most demonstrated statistically significant differences in favor of SQ terbutaline pump. One study, which showed a nonsignificant difference may have been underpowered.[17] Study populations may have overlapped.
	Women with singleton gestation and RPTL from the Matria database (n=558)[18]	Oral tocolytics (95.3% received oral terbutaline)	High	
	Women with twin gestation and RPTL from the Matria database (n=656)[16]	Oral nifedipine	Medium	
	Women with twin gestation and RPTL from the Matria database (n=706)[19]	Oral tocolytics (92.3% received oral terbutaline)	Medium	

RPTL = recurrent preterm labor; SQ = subcutaneous

Figure 14. Incidence of very low birth weight

		Odds ratio	Lower limit	Upper limit
OBSERVATIONAL	de la Torre (2008) (n=656)	0.40	0.26	0.61
OBSERVATIONAL	Fleming (2004) (n=284)	0.28	0.08	1.02
OBSERVATIONAL	Lam (2003) (n=558)	0.22	0.07	0.68
OBSERVATIONAL	Lam (2001) (n=46)	0.46	0.29	0.73

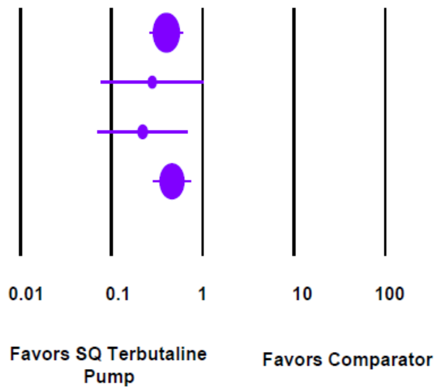

SQ = subcutaneous

All subsequent outcomes are presented at the study-level in Table F9 in Appendix F.

Mean Pregnancy Prolongation Index

Two observational studies defined pregnancy prolongation index as the ratio of the number of days from RPTL to delivery divided by the number of days to 37 weeks' gestation (i.e., the desired prolongation) (Table 36).[13,20] Both studies were in women with RPTL and showed statistically significant differences in favor of the SQ terbutaline pump, compared with oral terbutaline or no treatment (mean difference = 0.41, 95% CI: 0.26, 0.56; and 0.14, 95% CI: 0.02-0.26).[13,20] One study was rated as high risk of bias because groups were clearly imbalanced in risk factors for preterm birth, primary tocolytic therapy, and level of care.[13] The other study was rated as medium risk of bias because several criteria were rated as unclear due to incomplete reporting.[20] One study pertained to women with singleton gestation, the majority of whom were of African American origin[13] and the other study likely included women with single and multiple gestation, the majority of whom were classified as "nonwhite."[20]

Table 36. Summary table for mean pregnancy prolongation index (subgroup: RPTL)

Study Design (Number of Studies)	Population	Comparator(s)	Risk of Bias	Overall Findings
Prospective cohort (1)	Women with singleton gestation and RPTL (n=60)[13]	No treatment	High	Both studies reported statistically significant differences, in favor of SQ terbutaline pump.
Retrospective cohort (1)	Women with RPTL and likely included a mixture of women with single and multiple gestation (n=64)[20]	Oral terbutaline	Medium	

RPTL = recurrent preterm labor; SQ = subcutaneous

Need for Assisted Ventilation

One retrospective cohort that compared the SQ terbutaline pump with oral tocolytics in women with singleton gestation and RPTL from the Matria database reported requirement for ventilator among infants with NICU admission.[18] This study reported a nonsignificant difference (OR = 0.91, 95% CI: 0.62, 1.33; total number of events/sample size = 141/558). We rated this study as high risk of bias because there were apparent differences among groups in baseline characteristics and prognostic factors.

Incidence of NICU Admission

Seven studies reported incidence of NICU admission. Six observational studies, mostly from the Matria database, reported data for women with twin gestation and RPTL (Tables 37 and 38).[13,15-19] In addition, one underpowered RCT of low risk of bias showed no difference between SQ terbutaline pump and placebo in women with singletons (Figure 15) (total number of events/sample size = 23/51).[10]

Subgroup: Multiple Gestation

Two studies were in women with twin gestation and RPTL from the Matria database (Table 37). Both reported statistically significant results in favor of SQ terbutaline pump compared with oral tocolytics (OR = 0.72, 95% CI: 0.58, 0.91; total number of events/number of infants = 655/1312) and OR = 0.51, 95% CI: 0.41, 0.63; total number of events/sample size = 650/1393) (Table 38).[16,19] These studies were rated as medium risk of bias because there was insufficient information to assess potential limitations.

Table 37. Summary table for incidence of NICU admission (subgroup: multiple gestation)

Study Design (Number of Studies)	Population	Comparator(s)	Risk of Bias	Overall Findings
Retrospective cohort (2)	Women with twin gestation and RPTL from the Matria database (n=656,)[16]	Oral nifedipine	Medium	Both studies reported statistically significant differences, in favor of SQ terbutaline pump. Study populations may have overlapped.
	Women with twin gestation and RPTL from the Matria database (n=706)[19]	Oral tocolytics (92.3% received oral terbutaline)	Medium	

RPTL = recurrent preterm labor; SQ = subcutaneous

Subgroup: RPTL

All observational studies, including the two studies in women with twin gestation described above, pertained to women with RPTL (Table 38).[13,15-19] These studies were of medium to high risk of bias and five studies used the Matria database.[15-19] Overall, a consistent and significant benefit associated with pump was noted across the observational studies (OR range 0.28–0.72, 95% CI range: 0.08–0.58, 0.63–0.97).

Table 38. Summary table for incidence of NICU admission (subgroup: RPTL)

Study Design (Number of Studies)	Population	Comparator(s)	Risk of Bias	Overall Findings
Prospective cohort (1)	Women with singleton gestation and RPTL (n=60)[13]	No treatment	High	All observational studies pertained to women with RPTL and reported statistically significant differences in favor of the SQ terbutaline pump. Participant data may have overlapped in the Matria-based studies.
Retrospective cohort (5)	Women with singleton gestation and RPTL from the Matria database (n=1366)[15]	Oral nifedipine	High	
	Women with singleton gestation and RPTL from the Matria database (n=284) [17]	Oral nifedipine	Medium	
	Women with singleton gestation and RPTL from the Matria database (n=558)[18]	Oral tocolytics (95.3% received oral terbutaline)	High	
	Women with twin gestation and RPTL from the Matria database (n=656)[16]	Oral nifedipine	Medium	
	Women with twin gestation and RPTL from the Matria database (n=706)[19]	Oral tocolytics (92.3% received oral terbutaline)	Medium	

RCT = randomized controlled trial; RPTL = recurrent preterm labor; SQ = subcutaneous

Figure 15. NICU admission incidence

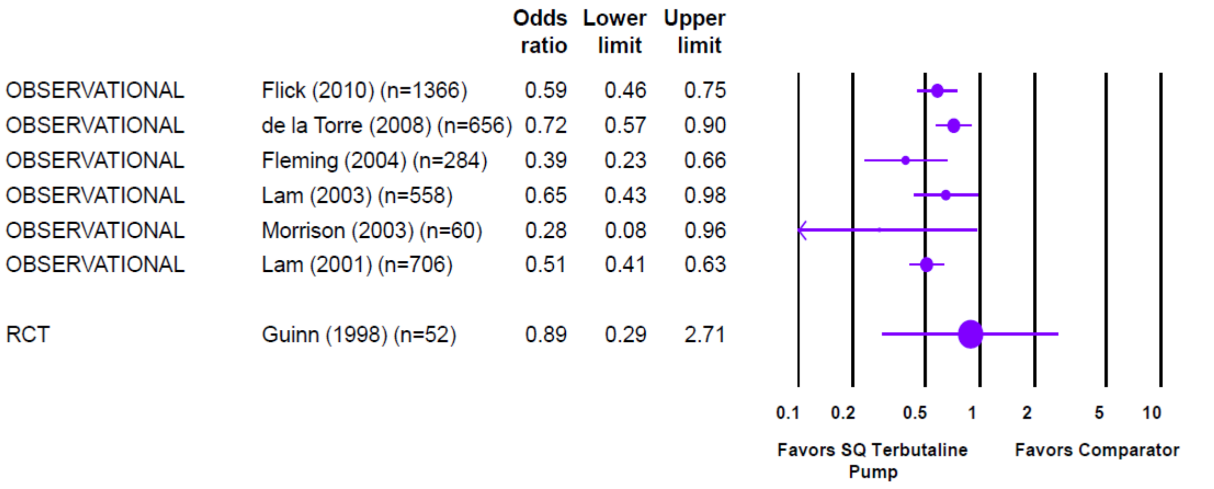

RCT = randomized controlled trial; SQ = subcutaneous

NICU Mean Length of Stay

Five studies reported data on NICU mean length of stay. Four observational studies, primarily from the Matria database, were available in women with RPTL[13,15,18,19]; one of these studies was additionally in women with twin gestation (Table 39 and Figure 16). These studies were mostly of high risk of bias. All reported statistically significant differences in favor of SQ terbutaline pump, compared with oral tocolytics or no treatment (range of mean difference in days: -3.50 to -17.90, 95% CI range: -5.26 to -32.88, -1.74 to -3.54). One RCT, which did not specifically pertain to any of the populations of interest, was conducted in women with single and twin gestation.[11] This study reported a statistically nonsignificant differences for the SQ terbutaline pump compared with placebo or oral terbutaline.[11] The plausible explanation for discrepant results between the RCT and observational evidence is inadequacy of study power.

Table 39. Summary table for NICU length of stay (subgroup: RPTL)

Study Design (Number of Studies)	Population	Comparator(s)	Risk of Bias	Overall Findings
Prospective cohort (1)	Women with singleton gestation and RPTL (n=60)[13]	No treatment	High	All studies reported statistically significant differences, in favor of the SQ terbutaline pump. Participant data in the SQ terbutaline pump groups of the Matria studies in singleton gestation may have overlapped.
Retrospective cohort (3)	Women with singleton gestation and RPTL from the Matria database (n=1366)[15]	Oral nifedipine	High	
	Women with singleton gestation and RPTL from the Matria database (n=558)[18]	Oral tocolytics (95.3% received oral terbutaline)	High	
	Women with twin gestation and RPTL from the Matria database (n=706)[19]	Oral tocolytics (92.3% received oral terbutaline)	Medium	

NICU = neonatal intensive care unit; RPTL = recurrent preterm labor; SQ = subcutaneous

Figure 16. NICU length of stay

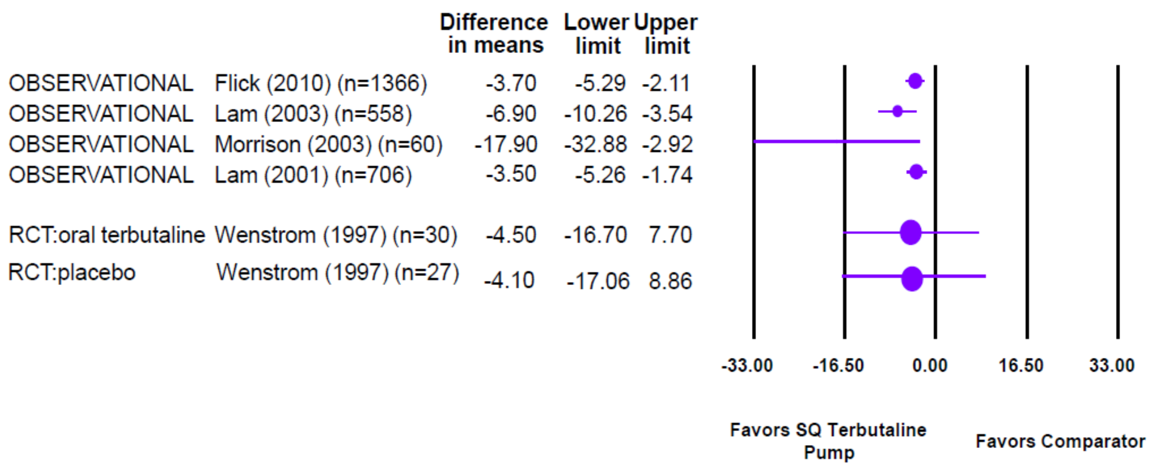

NICU = neonatal intensive care unit; RCT = randomized controlled trial; SQ = subcutaneous

Key Question 3. Maternal Harms

In women with arrested preterm labor, does treatment with a SQ infusion of terbutaline delivered by a pump, in comparison with placebo, conservative treatment, or other interventions increase the maternal harms of arrhythmia, heart failure, hyperglycemia, hypokalemia, maternal mortality, myocardial infarction, pulmonary edema, refractory hypotension, or result in an increased rate of maternal discontinuation of therapy or maternal withdrawal due to adverse effects (Withdrawal-AE)?

Key Points

- Strength of evidence is insufficient for Withdrawal-AE.
- Tachycardia/nervousness was significantly higher among women who received the SQ terbutaline pump in comparison with no treatment in a prospective cohort of women with singleton gestation and RPTL, although the point estimate was unreliable.

- Underpowered studies demonstrated indeterminate results for the outcomes of mortality, pulmonary edema, and therapy discontinuation (i.e., type II error cannot be excluded).
- Two studies demonstrated nonsignificant differences between the SQ terbutaline pump and oral terbutaline in the incidence of gestational diabetes, though type II error cannot be excluded.
- No data were available for the following outcomes: heart failure, hypokalemia, myocardial infarction, or refractory hypotension.
- FDA postmarketing surveillance has identified at least three maternal deaths and three cases of cardiovascular adverse events associated with the use of SQ terbutaline delivered by pump.

Detailed Analysis

Table F10 in Appendix F presents data for Key Question 3. None of the included studies reported data on heart failure, hypokalemia, myocardial infarction, refractory hypotension, or Withdrawal-AE (strength of evidence is insufficient). The evidence and determinants of applicability are presented below by outcome. Summary tables are presented if more than one study was available for an outcome; otherwise, all information has been summarized in the text.

Arrhythmia

In a prospective cohort study of women with singleton gestation and RPTL, Morrison et al. reported three cases of tachycardia/nervousness in women receiving the SQ terbutaline pump compared with no cases in the control group (OR=25.48, 95% CI: 1.23, 526.64).[13] We rated this study as high risk of bias because groups were imbalanced in risk factors for preterm birth, primary tocolytic therapy, and level of care. This evidence pertained to women with singleton gestation and RPTL, the majority of whom were of African American origin. However, given that the outcome was not restricted to arrhythmia specifically (i.e., nervousness was included), applicability is limited.

Hyperglycemia

Two studies reported data on gestational diabetes, diagnosed by 3-hour glucose tolerance test (GTT) (Table 40). Studies were not pooled because of heterogeneity in study designs and patient populations. Type II error cannot be excluded for the available evidence because these studies may be underpowered.

In a retrospective cohort, Regenstein et al. found a higher percentage of gestational diabetes among women in the SQ terbutaline pump group compared with the oral terbutaline group, but the difference was statistically nonsignificant (OR=1.94, 95% CI: 0.49, 7.65; total number of events/sample size=10/65).[21] This study included women with single or multiple gestations, the majority of whom were Caucasian.

Lindenbaum et al. conducted a nonrandomized trial in women with singleton gestation from a university hospital and found a lower percentage of gestational diabetes among women in the SQ terbutaline pump group compared with the oral terbutaline group. This result was also statistically nonsignificant (OR=0.46, 95% CI: 0.09, 2.40; total number of events/sample size=8/91).[12] The study by Lindenbaum et al. only included women who demonstrated normal 1-hour oral GTT between 24 to 28 weeks' gestation, so this study population may have been at lower risk for gestational diabetes than the population recruited by Regenstein et al.

Table 40. Summary table for maternal hyperglycemia

Study Design (Number of Studies)	Population	Comparator(s)	Risk of Bias	Overall Findings
Retrospective cohort (1)	Women with single or multiple gestation (n=69)[21]	Oral terbutaline	High	Both studies reported nonsignificant differences in the incidence of gestational diabetes. Possibility of type II error cannot be excluded.
Nonrandomized trial (1)	Women with singleton gestation from the Hospital of the University of Pennsylvania (n=91)[12]	Oral terbutaline	Medium	

Mortality

Two retrospective cohort studies investigated maternal mortality (Table 41). Lam et al. (2003) studied women with singleton gestation and RPTL[18] and Lam et al. (2001) studied women with twin gestation and RPTL.[19] Both studies used oral tocolytics as comparators. No maternal deaths were reported in either study.[18,19]

Table 41. Summary table for mortality

Study Design (Number of Studies)	Population	Comparator(s)	Risk of Bias	Overall Findings
Retrospective cohort (2)	Women with singleton gestation and RPTL from the Matria database (n=558)[18]	Oral tocolytics (95.3% received oral terbutaline)	High	No events reported.
	Women with twin gestation and RPTL from the Matria database (n=706)[19]	Oral tocolytics (92.3% received oral terbutaline)	Medium	

RPTL = recurrent preterm labor

Pulmonary Edema

Lam et al. (2003) and Lam et al. (2001) also reported data on pulmonary edema (Table 42).[18,19] Lam et al. (2003) observed one case of pulmonary edema in the oral tocolytic group (OR=0.33, 95% CI: 0.01, 8.19) and Lam et al. (2001) observed one case in the SQ terbutaline pump group (OR=3.01, 95% CI: 0.12, 74.11).[18,19] Both studies were likely underpowered to detect a difference due to low event rates.

Table 42. Summary table for pulmonary edema

Study Design (Number of Studies)	Population	Comparator(s)	Risk of Bias	Overall Findings
Retrospective cohort (2)	Women with singleton gestation and RPTL from the Matria database (n=558)[18]	Oral tocolytics (95.3% received oral terbutaline)	High	Both studies found statistically nonsignificant differences between SQ terbutaline pump and oral tocolytics. However, they were likely underpowered.
	Women with twin gestation and RPTL from the Matria database (n=706)[19]	Oral tocolytics (92.3% received oral terbutaline)	Medium	

RPTL = recurrent preterm labor; SQ = subcutaneous

Therapy Discontinuation

One prospective cohort and one RCT investigated maternal discontinuation of therapy (Table 43).[10,13] Morrison et al. reported no discontinuation of therapy in a prospective cohort of women with singleton gestation and RPTL.[13] Guinn et al. reported a higher percentage of treatment discontinuation among women with singleton gestation randomized to the SQ terbutaline pump group compared with placebo, but this difference was statistically nonsignificant (OR=1.79, 95%

CI: 0.58, 5.52; total number of events/sample size=20/52).[10] The RCT was likely underpowered to detect a difference for this outcome.

Table 43. Summary table for maternal discontinuation of therapy

Study Design (Number of Studies)	Population	Comparator(s)	Risk of Bias	Overall Findings
Prospective cohort (1)	Women with singleton gestation and RPTL (n=60)[13]	No treatment	High	No women discontinued treatment in the prospective cohort. In the RCT, discontinuation was higher in the SQ terbutaline pump group compared with placebo, but the difference was statistically nonsignificant (OR=1.79, 95% CI: 0.58, 5.52). Type II error cannot be excluded.
RCT (1)	Women with singleton gestation from Birmingham Hospital (n=52)[10]	Placebo	Low	

CI = confidence interval; OR = odds ratio; RCT = randomized controlled trial; RPTL = recurrent preterm labor; SQ = subcutaneous

Until 2009, 16 maternal deaths and 12 cases of maternal cardiovascular events (hypertension, myocardial infarction tachycardia, arrhythmias, and pulmonary edema) in association with terbutaline tocolysis were reported to the FDA. Of these, at least three maternal deaths and three cardiovascular adverse events were clearly reported to be in association with the use of the SQ terbutaline pump.[24]

Key Question 4. Neonatal Harms

In women with arrested preterm labor, does treatment with an SQ infusion of terbutaline delivered by a pump, in comparison with placebo, conservative treatment, or other interventions increase the neonatal harms of hypoglycemia, hypocalcemia, and ileus?

Key Points

- One case of hypoglycemia was reported in the placebo group of an underpowered RCT.[11]
- No data were available for the outcomes of hypocalcemia and ileus.

Detailed Analysis

Table F11 in Appendix F presents data for Key Question 4. No information was available for hypocalcemia or ileus. Hypoglycemia was reported by Wenstrom et al. in an RCT of women with single or twin gestation recruited from a university hospital in the United States.[11] This study compared the SQ terbutaline pump with placebo and oral terbutaline. One case of hypoglycemia was observed in the placebo group. No cases were reported among women who received the SQ terbutaline pump or oral terbutaline (OR=0.25, 95% CI: 0.01, 6.53 for SQ terbutaline pump versus placebo). The occurrence of a single hypoglycemic event among a sample size of 42 participants indicates that this study was underpowered to detect a difference in this outcome. Furthermore, we rated this study as high risk of bias because of selection bias, limitations in study power, and absence of blinding.

Key Question 5. Level of Activity and Level of Care

Can the differences in the outcomes above be partially explained by differences in level of care (e.g., frequency of followup, nurse visits, concomitant treatment, etc.) and level of activity (e.g., other children in the home, marital/support status, working status, bed rest, etc.) between the terbutaline pump group and the comparator group?

Key Points

- Few studies reported the level of maternal activity and the level of maternal care as study-level covariates, precluding meta-regression on outcomes.
- Qualitative analysis revealed no apparent trends between level of activity or level of care and the outcomes specified in Key Questions 1–4.

Detailed Analysis

Ratings for level of maternal activity and level of maternal care are provided in Appendix F, Tables F13 and F15. These tables provide overall ratings for each study, as well as ratings for the individual variables that comprise the level of activity and level of care variables.

Level of Maternal Activity

Level of maternal activity could not be rated for most studies due to insufficient information (i.e., incomplete reporting of marital status, working status, caring for other children, social support, bed rest, and restriction of maternal activities).[10,12,14-19,21] The participants in other studies were rated as having a low level of maternal activity, primarily because they were advised to remain at bed rest.[11,13,20,22,23] Level of activity in these studies did not vary by treatment groups, so these ratings represented study-level covariates.

Level of Maternal Care

Level of maternal care could not be rated for three studies due to insufficient information (i.e., incomplete reporting of nursing assessments, home uterine activity monitoring, home visits, education about preterm labor, telephone support, restriction of maternal activities, and other cointerventions).[11,12,21] Table 44 below summarizes ratings for all other studies. In two studies, level of care was found to vary among the SQ terbutaline pump and comparator groups and, therefore, in these cases it was not a study-level covariate.[13,20]

Table 44. Studies that could be rated for level of maternal care

First Author (Year)	Rating (Low, Moderate, High)
Flick (2010)[15]	High
de la Torre (2008)[16]	High
Fleming (2004)[17]	High
Lam (2003)[18]	High
Morrison (2003)[13]	SQ terbutaline pump group: High<br>Control group: Moderate
Lam (2001)[19]	High
Allbert (1994)[20]	SQ terbutaline pump group: High<br>Control group: Moderate
Guinn (1998)[10]	Moderate
Adkins (1993)[22]	Moderate
Morrison (1992)[14]	Moderate
Lam (1988)[23]	High

SQ = subcutaneous

Evidence Synthesis

A minimum of 12 studies were needed to explore the effect of level of activity or level of care on the outcomes specified in Key Questions 1–4 through meta-regression (number of studies needed is equal to 6×(n-1) for n levels for a categorical covariate). Studies were to be considered for meta-regression only if all of the following criteria were satisfied: there was no within-study confounding by level of activity or level of care, studies were not rated as unclear, and studies were similar enough with respect to patient population, intervention, and comparator.

A meta-regression could not be conducted for level of activity because sufficient information was available to rate only five studies. Furthermore, it was impossible to assess the impact of level of activity on effect estimates even in a qualitative manner because all five studies were rated as “low.”

Similarly, a meta-regression could not be conducted for level of care because only 11 studies were ratable and, of these, 2 were confounded by level of care. Of the remaining nine studies, three were rated as moderate [10,14,19,22] and six were rated as high.[15-18,23] These studies were qualitatively examined to explore trends in effect estimates by level of care. No trends were apparent between level of care and any of the outcomes in Key Questions 1–4.

Key Question 6. Incidence of Pump Failure

What is the incidence of failure of the pump device used for terbutaline infusion, including missed doses, dislodgment, and overdose?

Key Points

- Based on evidence from a case series, the incidence of dislodgement and pump malfunction were 2 percent (exact central CI, 0.5%, 10%).
- An underpowered RCT demonstrated indeterminate results for the outcomes of local pain and local skin irritation.
- No data were available for the outcomes of missed doses or overdose.

Detailed Analysis

Table F16 in Appendix F presents data for Key Question 6. None of the studies reported data on the incidence of missed doses or overdose. At least one study reported data on dislodgment and other pump-related outcomes, including infusion site infection, local pain, local skin irritation, and pump malfunction/mechanical failures and complications. The following pump manufacturers and models were reported in the studies: Adkins et al. used pump model 404-SP from MiniMed Technologies[22]; Lam et al. used pump model Autosyringe AS6-C U300 from Travenol, Deerfield, IL[23]; and Wenstrom et al. used pumps from Minimed Technologies (model not specified).[11] The sites of implantation were not reported in any study.

Dislodgment

Adkins et al. conducted a case series analysis of 51 women prescribed the SQ terbutaline pump and found one participant with catheter dislodgment (2 percent, exact central CI: 0.5%, 10%).[22]

Other Pump-Related Outcomes

Lam et al. found no infusion site infections in a case series analysis of nine women on the SQ terbutaline pump.[23]

Wenstrom et al. reported two cases of local pain among women randomized to the SQ terbutaline pump group and two cases in the placebo group.[11] This study also reported one case of local skin irritation in the SQ terbutaline pump group. Results for both local pain and local skin irritation were statistically nonsignificant when compared with either placebo or oral terbutaline (local pain: OR=0.77, 95% CI: 0.09, 6.45 for placebo and OR=5.74, 95% CI: 0.25, 130.38 for oral terbutaline; local skin irritation: OR=2.59, 95% CI: 0.10, 69.34 for placebo and OR=3.21, 95% CI: 0.12, 85.21 for oral terbutaline). However, given the sparse event rates, this RCT was likely underpowered to detect differences in either outcome.

The outcomes of pump malfunction/mechanical failures and complications were reported by two case series.[22,23] Lam et al. observed no events in a case series of nine women.[23] Adkins et al. reported an incidence of 2 percent (exact central CI, 0.5%, 10%) in a case series of 51 women.[22]

Overall Applicability for Body of Evidence

Table 45. Applicability assessment by the PICOTS domains

Population		Overall Conclusions
Inclusion/exclusion criteria	Most studies included women exclusively with RPTL.[13-20,23] In other studies, RPTL was not mentioned as an inclusion criterion, so it is unclear whether these populations consisted of women with single or multiple preterm labor episodes.[10-12,21,22]	The majority of evidence pertained to women with RPTL and singleton gestation. A couple of studies included women exclusively with RPTL and twin gestations; these participants represent a particularly high-risk, specialized group of patients.[16,19]<br><br>Very little is known about the study populations' demographic and clinical characteristics. Furthermore, the possibility that participants represent a select group of individuals cannot be entirely ruled out for a large proportion of the evidence base due to poor reporting of exclusion rates and sampling methodology.<br><br>Nine of 14 studies (64 percent) included women judged to be in labor on account of persistent contractions and cervical change. The definition of labor was unclear in other studies. Among the evidence that suggested that the pump was efficacious, 50 percent reported cervical change and contractions as part of the definition of labor while 50 percent did not report how labor was defined.
Demographic characteristics	Several studies (n=9) took place at single centers in the United States with limited demographic information.[10-14,20-23] Although age was reported in most of the single-center studies and race in some, there was little information on measures of socioeconomic status. Other studies included patients from a U.S.-based national database run by Matria Healthcare (now called Alere). These studies reported information on age and marital status but, as with the single-center studies, complete demographic information was lacking.	
Exclusion rate	The overall impact on applicability due to participant exclusion is unknown for much of the evidence because many studies did not report an exclusion rate. In one RCT, more than 90 percent of the eligible population declined to participate.[11]	
Run-in period (attrition before randomization)	In the two RCTs included in the review, no issues pertaining to run-in period were identified.	

Table 45. Applicability assessment by the PICOTS domains (continued)

Intervention		Overall Conclusions
Dose and duration	The dose and duration of the SQ terbutaline pump were generally typical of those used in clinical practice. However, some studies failed to provide adequate information regarding bolus and basal doses to allow assessment.	No major issues were identified with respect to the intervention, although there were gaps in reporting. Very few details were reported on cointerventions that could modify the effectiveness of therapy.
Level of care and training on pump administration	The level of care and training provided on pump administration were also deemed to be typical in most studies but, again, this information was not reported in some instances. In several studies, patients received specialized outpatient support, which may not be typical of practice.	
Cointerventions	Cointerventions with the potential to affect outcomes were considered to be bed rest, restriction of maternal activities, and administration of betamethasone. Corticosteroid use was reported in only one study, and details about bed rest and restriction of maternal activities were rarely reported.	
**Comparison**		**Overall Conclusions**
Dose/schedule and whether comparator is best available alternative	Several types of comparison groups were used in the studies. No issues were identified in the studies with an active treatment comparison group that would limit applicability.	No serious limitations to applicability due to comparators were identified.
**Outcomes**		**Overall Conclusions**
Clinical benefits (versus surrogate)	At least one clinical outcome was reported in most studies (i.e., neonatal outcomes of NEC, IVH, retinopathy of prematurity, sepsis, stillbirth, death; neonatal harm of hypoglycemia; and maternal harms of pulmonary edema, arrhythmia, hyperglycemia, death, and discontinuation of therapy). A few studies only reported surrogate outcomes (i.e., gestational age at delivery, birth weight, prolongation of pregnancy, or NICU admission). None of the studies reported any long-term outcomes such as childhood development, neurobehavioral testing, lung function, or vision.	Surrogate outcomes are the most commonly reported in this literature. Data on clinical outcomes and neonatal/maternal harms, including pump-related outcomes, is sparse. Several important clinical outcomes have not been reported. Assessment of long-term outcomes are also absent.
Individual harms and how defined	At least one neonatal or maternal harm outcome was reported in several studies. Very few studies reported outcomes related to the pump.	
**Timing of Followup**		**Overall Conclusions**
Timing of followup	In all studies, outcomes were assessed up to the point of delivery.	The absence of followup beyond delivery is a major limitation because important long-term outcomes were not evaluated.

Table 45. Applicability assessment by the PICOTS domains (continued)

Setting		Overall Conclusions
Geographic setting (standards of care)	All studies took place in the United States. Most studies were conducted at single study centers, and the remaining used a national database of women who were referred to an outpatient perinatal program. Most studies that took place at single study centers were at teaching hospitals, although one study took place at a private urban obstetrics and gynecology group practice.	All studies were from the United States, and participants were recruited either from a national database (Matria) or from single center sites. Women from the Matria database generally received a high level of care from an outpatient perinatal program. However, the distribution of regions from which patients were recruited into the national database is unknown and information about the standards followed by the individual practice sites that provided obstetrical care was not reported. Similarly, for those studies that took place at single center sites, the standards of care followed at these sites are unclear.
Clinical setting (level of care and population)	Women recruited into the national database received services from a specialized perinatal program that consisted of 24-hour nursing and pharmacy support, home uterine activity monitoring, individualized education, and provision of tocolytic therapy, including the SQ terbutaline pump. All women in these studies had RPTL and were either exclusively of singleton or twin gestation. Details of the clinical setting in the single-center studies were reported inconsistently. Some studies reported the provision of patient education, telephone support, home visits, and/or home uterine activity monitoring.	

IVH = intraventricular hemorrhage; NEC = necrotizing enterocolitis; NICU = neonatal intensive care unit; PICOTS = population, intervention, comparison, outcome, timing, setting; RCT = randomized controlled trial; RPTL = recurrent preterm labor; SQ = subcutaneous

Discussion

The rate of preterm birth in North America is considerably high at 12.3 percent.[1] These births will contribute to the health care burden, including increased short- and long-term neonatal morbidity. An effective and safe intervention to delay or prevent preterm birth would be a welcome addition to the maternity care provider's armamentarium.

To clarify the evidence on the efficacy and safety of subcutaneous terbutaline (SQ terbutaline) infusion by pump for the prevention of preterm birth, the Agency for Healthcare Research and Quality requested an evidence report answering six distinct questions. We applied rigorous selection criteria and assessed risk of bias of each study. This evidence report outlines a comprehensive review of all the available research.

In this final chapter, we first review the limitations of included studies, then the major findings pertaining to each key question and the strength of the evidence for the prespecified outcomes of incidence of delivery at various gestational ages; mean prolongation of pregnancy; bronchopulmonary dysplasia; significant intraventricular hemorrhage (grade III/IV); neonatal death and/or death within initial hospitalization; and maternal withdrawal due to adverse effects (Withdrawal-AE). We graded the strength of evidence based on the domains of overall risk of bias, consistency, directness, and precision. We then present our conclusions, make recommendations for future research, and offer clinical and public health perspectives.

Limitations of Included Studies

Studies contributing evidence were either absent or sparse for most outcomes. Although evidence from randomized controlled trials (RCTs) pertained to women with preterm labor, the specific populations of investigational interest were not distinguished. Furthermore, the two trials were clearly underpowered for outcomes of benefit and harms. Evidence pertaining to specific populations of women with preterm labor originated in observational studies of medium to high risk of bias. Across several studies, our concern that participants might have been double-counted because a common database was used could not be ruled out. Baseline clinical and socioeconomic characteristics with important prognostic implications were not reported across all studies. For example, no studies presented data on concomitant medications, body mass index, history of preeclampsia, cervical position, cervical consistency, cervical station, Bishop's Score, or fetal fibronectin. Cointerventions, such as administration of corticosteroids, were rarely described. None of the included studies assessed long-term childhood outcomes, such as childhood development, neurobehavioral testing, long-term lung function, and long-term vision.

In completing this review, we undertook an extensive grey literature search. Further, we requested relevant scientific information from the industry, Matria (now called Alere) Healthcare, and had many experts in the field participate in the review process. Despite this thorough process, the number of identified studies was very small—we had too few studies per outcome to perform statistical assessment of publication bias. We believe that all relevant data regarding the use of subcutaneous terbutaline for the prevention of preterm labor is captured in this review. Any exaggerated positive findings are more likely due to the medium to high risk of bias detected in observational studies than publication bias.

Summary of Key Findings

Key Question 1. Neonatal Health Outcomes

Information regarding neonatal health outcomes is derived from a few underpowered studies that examine the effect of SQ terbutaline infusion for the prevention of preterm birth on the key predictors of long-term health sequelae for offspring. The outcomes assessed in this review include those neonatal conditions that are generally accepted to be associated with mortality or impaired function later in life. Studies were either absent or underpowered for outcomes such as bronchopulmonary dysplasia, intraventricular hemorrhage, necrotizing enterocolitis, retinopathy of prematurity, sepsis, stillbirth, periventricular leukomalacia, and seizures, thereby limiting the utility of the data. Strength of evidence is insufficient for bronchopulmonary dysplasia and significant intraventricular hemorrhage.

Neonatal death was assessed using two different outcomes: classic neonatal death (i.e., death within the first 28 days of life) and death within initial hospitalization. Death within initial hospitalization is important given that the topic of interest is preterm birth—risk of morbidity and mortality in this population may extend beyond the first 28 days of life. However, no studies examined this variable and strength of evidence for this outcome, therefore, is insufficient. For neonatal death, strength of evidence favoring the SQ terbutaline pump over maintenance oral tocolytic therapy (92.3% received oral terbutaline) is low for women with recurrent preterm labor (RPTL) and twin gestation based on a single study from the Matria database (odds ratio [OR] = 0.09, 95% confidence interval [CI]: 0.01, 0.70). While this result is striking in the presence of insufficient findings on other neonatal health outcomes reported above, it is apparent that it stems from the largest of studies contributing data on neonatal health outcomes with over 700 patients. As such, it is the only outcome that appears to be adequately powered to reach statistical significance. For other populations of pregnant women with arrested preterm labor, the evidence was graded insufficient.

Key Question 2. Other Surrogate Outcomes

Surrogate outcomes are commonly used in maintenance tocolytic trials to assess efficacy. For many of these outcomes we could not assess data for important populations (as it was not reported), including delivery <28 weeks, specific gestational ages, racial or ethnic subgroups, women with previous preterm birth, or women with a history of preeclampsia.

A common outcome used in tocolytic trials of maintenance therapy is the incidence of delivery at various gestational ages. We chose to group gestational age at delivery according to commonly accepted categories of <28 weeks, <32 weeks, <34 weeks and <37 weeks, which correlate with improvements in clinical outcomes. Under this Key Question, we graded the strength of evidence for incidence of delivery at each gestational age cut-point and mean prolongation of pregnancy for prespecified populations. The strength of evidence for incidence of delivery at <28 and <34 weeks is insufficient. However, for the other outcomes strength of evidence favoring pump over oral tocolytics or no treatment is generally low for women with twin gestation and/or RPTL.

Mean birth weight significantly increased with the SQ terbutaline pump compared with oral tocolytics or no treatment in women with twin gestation and/or RPTL. This evidence largely originated from observational studies that used the same Matria database. Therefore, the studies were at risk of double-counting of participants across them. Two RCTs, which did not pertain to any specific population of interest, reported statistically nonsignificant differences between SQ

terbutaline pump and placebo. However, this result is inconclusive because of the possibility of type II error. The RCT evidence contrasted with results from larger cohort studies, which demonstrated consistent benefit.

The final group of surrogate outcomes that were assessed involved need for specialized neonatal care. For women with twin gestation and/or RPTL, evidence from observational studies, mostly from the Matria database, showed lower incidence of neonatal intensive care unit (NICU) admission and shorter duration of stay for infants whose mothers used SQ terbutaline pump (incidence of NICU admission: OR range 0.28–0.72, 95% CI range: 0.08–0.58, 0.63–0.97 and NICU mean length of stay: range of mean difference in days: -3.50 to -17.90, 95% CI range: -5.26 to -32.88, -1.74 to -3.54). One retrospective cohort in women with RPTL showed a nonsignificant decrease in the need for assisted ventilation among infants of the SQ terbutaline pump group.[18] No data were available on need for oxygen per nasal cannula.

Key Question 3. Maternal Harms

The data available on incidence of maternal harms are sparse. One small prospective cohort showed a significant increase in tachycardia/nervousness among women using SQ terbutaline pump (OR = 25.48, 95% CI 1.23, 526.64).[13] In one RCT of terbutaline infusion versus placebo, 45.8 percent of patients discontinued the terbutaline infusion compared to 32 percent of patients who discontinued placebo treatment.[10] The available data does not suggest the reasons for discontinuation of therapy (e.g., inconvenience versus nuisance side effects versus major complications).

Results for the outcomes of maternal mortality, pulmonary edema, maternal hyperglycemia, and therapy discontinuation were inconclusive because studies were not adequately powered to detect these rare findings. No data were available for several recognized adverse outcomes, including hypokalemia, refractory hypotension, heart failure, myocardial infarction, and study withdrawal due to adverse effects (strength of evidence is insufficient).

The Food and Drug Administration (FDA) has issued new warnings against the use of terbutaline in pregnant women for prevention or prolonged treatment (beyond 48 to 72 hours) of preterm labor. [24] Based on postmarketing reports of maternal deaths and serious cardiovascular adverse events associated with the obstetrical use of terbutaline, the FDA is requiring that a Boxed Warning and Contraindication be placed on injectable and oral terbutaline drug labels. Between 1976 and 2009, 16 maternal deaths were reported; at least three of these cases were clearly reported to be in association with the administration of SQ terbutaline pump. Between 1998 and 2009, 12 maternal cases of serious cardiovascular events were reported, including arrhythmias, myocardial infarction, pulmonary edema, hypertension and tachycardia; at least three of these cases were clearly reported to be in association with the administration of the SQ terbutaline pump.[24] Although meriting transparent disclosure in the form of a warning, evidence emerging from case reports is usually regarded as noncomparative and a hypothesis generating signal rather than a hypothesis testing confirmation.[25] Furthermore, case reports are useful in identifying rare and unexpected adverse events—the rarer the adverse event, the stronger the effect size, and the magnitude of effect size is an important criterion that increases our confidence in an estimate.[9] However, adverse events such as death, hypertension, tachycardia, arrhythmias, and pulmonary edema that were reported with the use of terbutaline are not so unexpected in any adult population—pregnant women may experience these adverse events in the absence of terbutaline therapy due to other reasons.

Key Question 4. Neonatal Harms

Neonatal harms data were also very sparse. In one small RCT, only one case of hypoglycemia was identified in an infant whose mother received placebo infusion.[11] Given such a small event rate, the utility of this information is limited by insufficient power. No data were available for the incidence of neonatal hypocalcemia or ileus.

Key Question 5. Level of Activity and Level of Care

Differences in maternal activity and level of care could potentially explain differences in outcomes. Level of activity was rated as low, normal, or high based on a composite assessment of the following variables: marital status, working status, caring for other children in the home, available social support, bed rest, and restriction of maternal activities. Level of care was rated as low, moderate, or high based on the following variables: nursing assessments, home uterine activity monitoring, home visits, education about preterm labor, telephone support, restriction of maternal activities, and other cointerventions. Unfortunately, few studies reported these as study level covariates, which precluded statistical assessment of heterogeneity by meta-regression. Furthermore, a qualitative assessment of heterogeneity revealed no apparent trends.

Key Question 6. Incidence of Pump Failure

SQ terbutaline is administered by a mechanical pump, and, therefore, it is important to consider possible technology-related issues. Although the Key Question only specified missed doses, dislodgment, and overdose, we investigated a wider range of pump-related problems, including pump malfunction and local pain or skin irritation. No study reported on outcomes of missed doses or overdose. One case series reported a 2 percent incidence of dislodgement of the SQ catheter.[22] The same series reported a 2 percent incidence of unspecified pump malfunction. One small RCT reported the side effects of local pain and skin irritation, which were present in less than 20 percent of patients and not statistically different in patients receiving terbutaline infusion compared to a placebo pump.[11] No infusion site infections were reported in another case series.[23] Although these studies do not suggest that pump-related complications are significant, adverse events related to the pump device should be documented in future studies.

Conclusions

The available evidence for the SQ terbutaline pump as maintenance tocolytic therapy in women with arrested preterm labor pertained to only two of the specific populations of interest: women primarily with singleton gestation and RPTL or those with twin gestation and RPTL. This evidence base came entirely from observational studies, and most studies (45 percent) originated from a single proprietary database. The available RCT evidence did not apply to any of the specific preterm populations described in Key Questions 1 and 2, but rather included nonspecific populations of women with preterm labor.

For neonatal death, the strength of evidence favoring SQ terbutaline pump therapy compared with oral tocolytics is low for women with twin gestation and RPTL (OR=0.09, 95% CI: 0.01, 0.70). Strength of evidence favoring the terbutaline pump compared to oral tocolytics or no treatment is also low for the surrogate outcomes of pregnancy prolongation in women with twin gestation and/or RPTL. Insufficient evidence addressed bronchopulmonary dysplasia, death within initial hospitalization, significant intraventricular hemorrhage, and maternal withdrawal

due to adverse events. The strength of evidence for nonspecific populations of women with preterm labor described in RCTs is insufficient.

Scant and underpowered evidence demonstrated inconclusive results for all other neonatal health outcomes, neonatal harms, maternal harms, and pump-related outcomes. Observational studies of medium to high risk of bias, with potential for participant double-counting, showed the benefit of the SQ terbutaline pump compared with oral tocolytics for other surrogate outcomes, such as birth weight and NICU admission.

FDA postmarketing surveillance has detected maternal deaths and maternal cardiovascular events in association with terbutaline tocolysis in general, and pump therapy in particular. However, causal association cannot be established with this evidence.

In conclusion, although evidence suggests that pump therapy is beneficial as maintenance tocolysis, our confidence in the validity and reproducibility of this evidence is low. While postmarketing surveillance has detected cases of serious harms, the safety of the therapy remains unclear.

Comparison of Results With Other Systematic Reviews

In agreement with the review by Nanda et al., we found that the available RCT evidence showed nonsignificant differences between the SQ terbutaline pump and placebo or oral terbutaline for several outcomes.[4] Nanda et al. concluded, "Terbutaline pump maintenance therapy has not been shown to decrease the risk of preterm birth by prolonging pregnancy" (p.2). The review also commented on the lack of information regarding safety and advocated for further study. We agree with these conclusions, but would also emphasize that the RCT evidence was likely prone to type II error.

The Hayes group conducted a systematic review of the SQ terbutaline pump for maintenance therapy and, in contrast to the review by Nanda et al., included both observational studies and RCTs.[5] This review found that the available RCT and observational evidence was conflicting and our review came to a similar conclusion; RCT evidence did not demonstrate benefit of the SQ terbutaline pump, although cohort studies of limited methodological validity demonstrated statistically significant effects in favor of the pump for several outcomes. Our review included some additional studies that were not part of the Hayes review because we performed a more recent search, we did not have a lower cutoff year, and we included case series to assess pump-related outcomes. Furthermore, the Hayes review did not specifically investigate different populations, the effect of confounding by level of maternal activity or level of maternal care, or pump-related outcomes. Our review examined these additional factors but found only limited data to address them. We also graded the strength of evidence from the body of observational studies as mostly insufficient or low for women with twin gestation and/or RPTL.

Applicability

Below we summarize characteristics of applicability based on the domains of population, intervention, comparator, outcome, and setting. The following factors should be considered by maternity care providers and policymakers when entertaining the option of recommending the SQ terbutaline pump to women with preterm labor.

Nine of 14 studies (64 percent) included women judged to be in labor on account of persistent contractions and cervical change. The definition of labor was unclear in other studies. Among the evidence that suggested that the pump was efficacious, 50 percent reported cervical

change and contractions as part of the definition of labor, and 50 percent did not report how labor was defined.

The majority of evidence included women with RPTL (i.e., treated with first-line tocolytic therapy for 48 hours, have cessation of symptoms, and then present with a second episode) and singleton gestation. Some evidence pertained additionally to women with twin gestation and RPTL, which is a high-risk, specialized group of patients.

Several studies included patients from a national proprietary database run by Matria (now called Alere) Healthcare, which provides an outpatient perinatal program consisting of 24-hour nursing and pharmacy support, home uterine activity monitoring, individualized education, and provision of tocolytic therapy to women with preterm labor. These women generally received a high level of care based on nursing assessments, home uterine activity monitoring, home visits, education about preterm labor, telephone support, restriction of maternal activities, and other cointerventions. The distribution of regions from which patients were recruited into the national database is unknown. Further, it is impossible to make any judgments about the standards followed by the individual practice sites that were providing obstetrical care to the women in the database.

In general, the dose and duration of SQ terbutaline pump therapy were typical of those used in clinical practice, although some studies did not provide adequate information regarding basal and bolus doses to allow assessment. Level of care and training provided to patients on pump were also typical in most studies, although this information was also somewhat limited. In several studies, patients received specialized outpatient support in the form of nursing/pharmacy support, monitoring and contact with physicians; this level of care may not be typical of practice. Investigators typically did not report information on cointerventions, such as bed rest, restriction of maternal activities, and administration of corticosteroids.

Multiple comparison groups were used, including no treatment, placebo, and oral tocolytics.

Outcomes most commonly reported in the literature were surrogates, such as gestational age at birth, prolongation of pregnancy, and birth weight. Data on clinical outcomes and neonatal/maternal harms, including pump-related outcomes, are sparse. Several important clinical outcomes have not been investigated. These include short-term outcomes of neonatal death within initial hospitalization, intraventricular hemorrhage, and bronchopulmonary dysplasia and long-term outcomes, such as developmental and neurobehavioral testing.

No long-term outcomes of the SQ terbutaline pump for maintenance tocolysis have been assessed. This absence of followup beyond delivery is a major limitation of the available evidence.

Future Research

Although cohort studies have provided a glimpse of the potential for SQ terbutaline pump to improve short-term neonatal outcomes for fetuses at risk for preterm birth, the answers to several important questions remain unanswered. Most importantly, it remains to be seen whether SQ terbutaline pump therapy alters long-term development or systemic impairment of offspring, and neonatal/maternal morbidity and mortality. The limitations of the available data must also be recognized. Most of the cohort studies were medium to high risk of bias. In addition, several of the cohort studies investigated participants from a single proprietary database (Matria), which raises concerns regarding double-counting of patients and common biases. Therefore, results showing effectiveness should be interpreted with caution, especially in light of the recent FDA warnings.

Information is lacking on the effectiveness and safety of the SQ terbutaline pump as a maintenance tocolytic treatment in specific populations, including women who deliver at specific gestational ages, women of different racial or ethnic backgrounds, and women with previous preterm birth or preeclampsia. Future studies, whether observational or experimental in design, should focus on garnering evidence for these specific populations.

Below we provide some specific recommendations for the conduct of RCTs and observational studies to further elucidate the potential benefits and harms of SQ terbutaline pump for maintenance tocolysis.

Randomized Trials

We recommend that an adequately powered randomized controlled and pragmatic clinical trial that assesses the SQ terbutaline pump as a maintenance tocolytic be conducted. A pragmatic RCT is designed to have broad applicability so that the results can guide decisions about practice.[26] Such a trial should be placebo controlled and include blinding of study participants, care providers, and study personnel. Consideration should be given to employing multiple treatment arms in order to evaluate the pump against other tocolytic agents and conservative management. Furthermore, the level of care provided to participants (i.e., nursing assessments, home uterine monitoring, education, telephone support, and restriction of activities) should be practical, feasible, and likely to be adopted in routine practice. Important cointerventions, such as administration of corticosteroids, should be reported. A full accounting of the number of women approached but not enrolled should be included to allow users to assess the impact of respondent bias. The analysis should be "intent to treat," where all participants assigned by randomization to each group are included in the primary comparisons, regardless of whether the assigned medication was received. Outcomes to be examined should go beyond those of prolongation of pregnancy and birthweight to hard clinical endpoints of neonatal morbidity, such as bronchopulmonary dysplasia, necrotizing enterocolitis, significant intraventricular hemorrhage (grade III/IV), retinopathy of prematurity, sepsis, stillbirth, and neonatal death. Lastly, there should be long-term followup to assess subsequent childhood outcomes. Pharmacodynamic and pharmacokinetic outcome measures can additionally be studied to understand inter-individual differences in effectiveness and toxicity and avoidance of β-agonist related tachyphylaxis.

Conducting such RCTs to assess the efficacy of tocolytics in general is notoriously difficult. A definitive trial in this domain must include a focus on accurate diagnosis of preterm labor (perhaps combining stringent clinical criteria with factors such as positive fetal fibronectin and shortened transvaginal cervical length). Emphasis must also be placed on securing funding and maintaining followup for an appropriate duration of time to allow assessment of long-term childhood outcomes, including neurobehavioral testing and developmental assessment.

Observational Studies

Although the RCT is the ideal study design for evaluating the efficacy of interventions, at times it may not be considered feasible for a number of reasons, such as a prohibitive sample size requirement and ethical considerations. We realize that collecting RCT evidence on clinically important outcomes may not be possible because a large number of patients will need to be recruited to detect rare events, such as maternal deaths. Therefore, we additionally propose:

- Well-designed, well-powered cohort studies examining clinical outcomes. These studies should include a representative and inception cohort of all patients with arrested preterm labor. Since observational studies are susceptible to the effects of confounding, future

observational studies should measure, report, and adjust for potential confounders such as fetal fibronectin, cervical length/dilation, cerclage, maternal characteristics (e.g. age, race), level of care and activity, and concomitant medications; propensity scores based on these variables may be considered. Other considerations about power, multiple comparison groups, level of care, reporting of cointerventions, and long-term followup are the same as for RCTs.

- Record linkage studies in which mother's prenatal, and infants NICU and childhood developmental electronic health records are linked may be a more practical research proposition for the near future with improvements in quality and accessibility of electronic patient records. NICU registries in which prenatal data of mothers are available can be very valuable source. However, such linkage based studies may also be impacted by biases not uncommon to cohort study designs, especially confounding because of unmeasured or unrecorded variables with important prognostic implications.

Implications

Given the sparse evidence favoring SQ terbutaline pump therapy over other tocolytics or no treatment, we have low confidence that the evidence reflects the true effect. Further research is likely to change the confidence in the estimate of effect and is likely to change the estimate.[9] Therefore, this systematic review calls into question the evidence base for the current practice of using terbutaline pump as a maintenance tocolytic agent. Decisionmakers and policymakers should take into consideration the limitations of the available data when formulating recommendations.

Most of the available data are surrogate outcomes of preterm labor. Although many decisions regarding the SQ terbutaline pump are currently made on the assumption that short-term outcomes (for example, a heavier neonate or an infant born beyond 32 weeks) will correlate well with improved long-term outcomes, rigorous scientific evaluation is needed to confirm whether such factors do, in fact, lead to better outcomes in this population. As with any intervention, the benefits of providing treatment at varying gestational ages should outweigh the risks associated with the intervention. Given the sparse epidemiological and trial evidence available on maternal and neonatal harms and the recent FDA warning against the use of terbutaline for tocolysis based on case reports of maternal deaths and serious cardiovascular events, further discussion among policymakers and health care providers is urgently needed to determine if the risks and costs of SQ terbutaline infusion by pump are justified in this vulnerable population.

References

1. Behrman R, Butler A. Preterm Birth: Causes, Consequences, and Prevention. Washington, DC: National Academies Press; 2007.
2. Centers for Disease Control and Prevention. Birthweight and Gestation. Available at: www.cdc.gov/nchs/fastats/birthwt.htm. Accessed May 12, 2010.
3. Mathews TJ, MacDorman MF. Infant mortality statistics from the 2006 period linked birth/infant death data set. Natl Vital Stat Rep 2010 Apr 30;58(17):1-31. PMID: 20815136.
4. Nanda K, Cook LA, Gallo MF, et al. Terbutaline pump maintenance therapy after threatened preterm labor for preventing preterm birth. Cochrane Database Syst Rev 2002 Dec;(4):
5. HAYES, Inc. Continuous subcutaneous terbutaline infusion for treatment of preterm labor (Structured abstract). Lansdale, Pa: Hayes, Inc 2006;
6. Hayes E, Moroz L, Pizzi L, et al. A cost decision analysis of 4 tocolytic drugs. Am J Obstet Gynecol 2007 Oct;197(4):383-6. PMID: 17904969.
7. Agency for Healthcare Research and Quality. Methods Guide for Effectiveness and Comparative Effectiveness Reviews[Draft]. AHRQ Publication No. 10(11)-EHC063-EF. Available at: www.effectivehealthcare.ahrq.gov/ehc/products/60/318/Methods-Guide_Prepublication-Draft_03-2011.pdf. Rockville, MD: 2011.
8. Santaguida, P and Raina, P. McMaster Quality Assessment Scale of Harms (McHarm) for primary studies: Manual for use of the McHarm. Available at: http://hiru.mcmaster.ca/epc/mcharm.pdf. Accessed December 22, 2010.
9. Owens DK, Lohr KN, Atkins D, et al. AHRQ series paper 5: grading the strength of a body of evidence when comparing medical interventions--agency for healthcare research and quality and the effective health-care program. J Clin Epidemiol 2010 May;63(5):513-23. PMID: 19595577.
10. Guinn DA, Goepfert AR, Owen J, et al. Terbutaline pump maintenance therapy for prevention of preterm delivery: a double-blind trial.[see comment]. Am J Obstet Gynecol 1998 Oct;179(4):874-8. PMID: 9790362.
11. Wenstrom KD, Weiner CP, Merrill D, et al. A placebo-controlled randomized trial of the terbutaline pump for prevention of preterm delivery. Am J Perinatol 1997 Feb;14(2):87-91. PMID: 9259905.
12. Lindenbaum C, Ludmir J, Teplick FB, et al. Maternal glucose intolerance and the subcutaneous terbutaline pump. Am J Obstet Gynecol 1992 Mar;166(3):925-8. PMID: 1550166.
13. Morrison JC, Chauhan SP, Carroll CS, Sr., et al. Continuous subcutaneous terbutaline administration prolongs pregnancy after recurrent preterm labor. Am J Obstet Gynecol 2003 Jun;188(6):1460-5. PMID: 12824979.
14. Morrison JC, Allbert JR, Floyd RC, et al. Interval to delivery in high-risk patients: Do tocolytic agents really work? Int J Gynaecol Obstet 1992;38(3):189-93. PMID: 1992210309.
15. Flick AA, de la Torre L, Roca LE, et al. An examination of the clinical benefits and cost-effectiveness of tocolytic replacement following recurrent preterm labor. Am J Perinatol 2010 Jan;27(1):53-9. PMID: 19823965.
16. de la Torre L, Istwan NB, Desch C, et al. Management of recurrent preterm labor in twin gestations with nifedipine tocolysis. Am J Perinatol 2008 Oct;25(9):555-60. PMID: 18773381.

17. Fleming A, Bonebrake R, Istwan N, et al. Pregnancy and economic outcomes in patients treated for recurrent preterm labor. J Perinatol 2004 Apr;24(4):223-7. PMID: 14999214.

18. Lam F, Istwan NB, Jacques D, et al. Managing perinatal outcomes: the clinical benefit and cost-effectiveness of pharmacologic treatment of recurrent preterm labor. Managed Care 2003 Jul;12(7):39-46. PMID: 12891954.

19. Lam F, Bergauer NK, Jacques D, et al. Clinical and cost-effectiveness of continuous subcutaneous terbutaline versus oral tocolytics for treatment of recurrent preterm labor in twin gestations. J Perinatol 2001 Oct;21(7):444-50. PMID: 11894512.

20. Allbert JR, Johnson C, Roberts WE, et al. Tocolysis for recurrent preterm labor using a continuous subcutaneous infusion pump. J Reprod Med 1994 Aug;39(8):614-8. PMID: 7996525.

21. Regenstein AC, Belluomini J, Katz M. Terbutaline tocolysis and glucose intolerance. Obstet Gynecol 1993 May;81(5 (Pt 1):739-41. PMID: 8469464.

22. Adkins RT, Van Hooydonk JE, Bressman PL, et al. Prevention of preterm birth: early detection and aggressive treatment with terbutaline.[see comment]. South Med J 1993 Feb;86(2):157-64. PMID: 8240475.

23. Lam F, Gill P, Smith M, et al. Use of the subcutaneous terbutaline pump for long-term tocolysis. Obstet Gynecol 1988 Nov;72(5):810-3. PMID: 3173932.

24. U.S. Food and Drug Administration. FDA Drug Safety Communication: New warnings against use of terbutaline to treat preterm labor. Available at: www.fda.gov/Drugs/DrugSafety/ucm243539.htm. Accessed April 7, 2011.

25. Chou R, Aronson N, Atkins D, et al. AHRQ series paper 4: assessing harms when comparing medical interventions: AHRQ and the effective health-care program. J Clin Epidemiol 2010 May;63(5):502-12. PMID: PM:18823754.

26. Zwarenstein M, Treweek S, Gagnier JJ, et al. Improving the reporting of pragmatic trials: an extension of the CONSORT statement. BMJ 2008;337:a2390. PMID: 19001484.

27. Iams J, Romero R, Creasy R. Preterm labor and birth. In: Creasy R, Resnik R, Iams J, eds. Creasy and Resnik's Maternal-fetal medicine: principles and practice. 6th ed. Philadelphia PA: Saunders Elsevier; 2010. p. 545–82.

28. Martin JA, Osterman MJ, Sutton PD. Are preterm births on the decline in the United States? Recent data from the National Vital Statistics System. NCHS Data Brief 2010 May;(39):1-8. PMID: 20604990.

29. Ananth CV, Vintzileos AM. Epidemiology of preterm birth and its clinical subtypes. J Matern Fetal Neonatal Med 2006 Dec;19(12):773–82. PMID: 17190687.

30. King JF, Grant A, Keirse MJ, et al. Beta-mimetics in preterm labour: an overview of the randomized controlled trials. Br J Obstet Gynaecol 1988 Mar;95(3):211–22. PMID: 2897207.

31. Hueston WJ. Preterm contractions in community settings: II. Predicting preterm birth in women with preterm contractions. Obstet Gynecol 1998 Jul;92(1):43–6. PMID: 9649090.

32. Macones GA, Segel SY, Stamilio DM, et al. Prediction of delivery among women with early preterm labor by means of clinical characteristics alone. Am J Obstet Gynecol 1999 Dec;181(6):1414–8. PMID: 10601922.

33. Bastek JA, Sammel MD, Pare E, et al. Adverse neonatal outcomes: examining the risks between preterm, late preterm, and term infants. Am J Obstet Gynecol 2008 Oct;199(4):367–8. PMID: 18928976.

34. Weismiller DG. Preterm labor. Am Fam Physician 1999 Feb 1;59(3):593–602. PMID: 10029786.

35. Sebire NJ, Jolly M, Harris J, et al. Is maternal underweight really a risk factor for adverse pregnancy outcome? A population-based study in London. BJOG 2001 Jan;108(1):61–6. PMID: 11213006.

36. Dole N, Savitz DA, Hertz-Picciotto I, et al. Maternal stress and preterm birth. Am J Epidemiol 2003 Jan 1;157(1):14-24. PMID: 12505886.

37. Rust OA, Bofill JA, Arriola RM, et al. The clinical efficacy of oral tocolytic therapy.[see comment]. Am J Obstet Gynecol 1996 Oct;175(4 Pt 1):838–42. PMID: 8885732.

38. Berkman ND, Thorp JM, Jr., Hartmann KE, et al. Management of preterm labor. Evid Rep Technol Assess (Summ) 2000 Oct;(18):1–6. PMID: 11127122.

39. Sampson M, McGowan J, Cogo E, et al. An evidence-based practice guideline for the peer review of electronic search strategies. J Clin Epidemiol 2009 Sep;62(9):944-52. PMID: 19230612.

40. UpToDate. Available at: http://www.uptodate.com/online/content/search.do. Accessed November 29, 2010.

41. Sanchez-Ramos L, Kaunitz AM, Gaudier FL, et al. Efficacy of maintenance therapy after acute tocolysis: a meta-analysis.[see comment]. Am J Obstet Gynecol 1999 Aug;181(2):484–90. PMID: 10454704.

42. Thornton JG. Maintenance tocolysis. BJOG 2005 Mar;112 Suppl 1:118–21. PMID: 15715609.

43. Higgins J, Green S, eds. Cochrane handbook for systematic reviews of interventions. Wiley Online Library; 2008. Chapter 10, Addressing Reporting Biases.

44. Allbert J, Johnson C, Roberts W. Subcutaneous terbutaline tocolysis for recurrent preterm labor via infusion pump. Am J Obstet Gynecol 1991;164:365.

Acronyms and Abbreviations

AHRQ	Agency for Healthcare Research and Quality
BMI	Body mass index
BPD	Bronchopulmonary dysplasia
CI	Confidence interval
CRD	Centre for Reviews and Dissemination
FDA	Food and Drug Administration
GA	Gestational age
GTT	Glucose tolerance test
IVH	Intraventricular hemorrhage
NICU	Neonatal intensive care unit
OR	Odds ratio
PICOTS	Population, intervention, comparison, outcome, timing, setting
PTL	Preterm labor
RCT	Randomized controlled trial
RPTL	Recurrent preterm labor
SQ	Subcutaneous
SQT	Subcutaneous terbutaline
SRC	Scientific Resource Center
TEP	Technical expert panel
Withdrawal-AE	Withdrawal due to adverse effects

Glossary

Applicability: The relevance of the evidence base to an external population.

Bias: A systematic error, arising from participant selection or outcome measurement that produces an erroneous effect estimate.

Preterm birth: Delivery before completion of the 37th week of gestation.

Strength of evidence: The strength of evidence grading reflects a global assessment of the evidence base. Strength of evidence may be designated as insufficient, low, moderate or high based on the domains of study risk of bias, consistency, directness, and precision.

Tocolytic: An agent that inhibits labor by slowing or halting uterine contractions.

Appendix A. Search Strategies

Database: Ovid MEDLINE(R) In-Process & Other Non-Indexed Citations and Ovid MEDLINE(R) <1950 to 2009 Dec Week 1> (updated to April 1, 2011)
Search Strategy:
----
1 exp Obstetric Labor, Premature/ (14094)
2 (PTL or PTB or RPTL).ti,ab. (2396)
3 ((premature* or pre-mature* or preterm or pre-term or early) adj5 (labor* or labour* or birth* or deliver*)).ti,ab. (32212)
4 ((premature* or pre-mature* or preterm or pre-term or early) adj5 ((uterine or uterus) adj2 contract*)).ti,ab. (306)
5 Tocolysis/ or Tocolytic Agents/ (1876)
6 (tocolysis or tocolytic*).ti,ab. (2856)
7 1 or 2 or 3 or 4 or 5 or 6 (40062)
8 exp Terbutaline/ (2921)
9 (Terbutalin* or Brethaire or Brethine or Bricanyl or "BRN 2370513" or "EINECS 245-385-8" or "UNII-N8ONU3L3PG").ti,ab. (3089)
10 (23031 25 6 terbutaline or 23031 32 5 terbutaline sulfate).rn. (2921)
11 8 or 9 or 10 (3761)
12 exp Injections, Subcutaneous/ (31708)
13 exp Infusion Pumps/ (9822)
14 exp Home Infusion Therapy/ (555)
15 exp Infusions, Parenteral/ (75058)
16 (subcutaneous* or SubQ or sub-cutaneous* or pump or pumps or infuse or infused or infuses or infusing or infusion* or infuser*).ti,ab. (354453)
17 ((home adj3 therapy) or (home adj3 therapies) or (home adj3 tocoyl*) or (home-based adj3 therapy) or (home-based adj3 therapies) or (home-based adj3 tocoyl*)).ti,ab. (2249)
18 ((maintenance adj3 therapy) or (maintenance adj3 therapies) or (maintenance adj3 therapeutic) or (maintenance adj3 treatment*) or (maintenance adj3 tocoly*) or (supportive adj3 therapy) or (supportive adj3 therapies) or (supportive adj3 treatment*) or (supportive adj3 tocoyls*) or (outpatient adj3 therapy) or (outpatient adj3 therapies) or (outpatient* adj3 treatment*) or (outpatient* adj3 tocoly*)).ti,ab. (27705)
19 ((long-term adj therapy) or (long-term adj therapies) or (long-term adj therapeutic) or (long-term adj treatment*) or (long-term adj management) or (long-term adj tocoly*) or (longterm adj therapy) or (longterm adj therapies) or (longterm adj therapeutic) or (longterm adj treatment*) or (longterm adj management) or (longterm adj tocoly*)).ti,ab. (23491)
20 12 or 13 or 14 or 15 or 16 or 17 or 18 or 19 (457526)
21 11 and 20 (694)
22 7 and 21 (158)
23 from 22 keep 1-158 (158)

Database: EMBASE <1980 to 2009 Week 49> (Updated to April 1, 2011)
Search Strategy:
----
1 exp premature labor/ (12859)
2 (PTL or PTB or RPTL).ti,ab. (1981)
3 ((Premature* or pre-mature* or preterm or pre-term or early) adj5 (labor* or labour* or birth* or deliver*)).ti,ab. (24223)
4 ((Premature* or pre-mature* or preterm or pre-term or early) adj5 ((uterine or uterus) adj2 contract*)).ti,ab. (243)
5 exp Tocolysis/ (2223)
6 (tocolysis or tocolytic*).ti,ab. (2419)
7 1 or 2 or 3 or 4 or 5 or 6 (30904)
8 exp terbutaline/ (8346)
9 exp terbutaline sulfate/ (492)
10 (23031 25 6 or 23031 32 5).rn. (8627)
11 (Terbutalin* or Brethaire or Brethine or Bricanyl or "BRN 2370513" or "EINECS 245-385-8" or "UNII-N8ONU3L3PG").ti,ab. (2721)
12 (Terbutalin* or Brethaire or Brethine or Bricanyl).tn. (1416)
13 8 or 9 or 11 or 12 (8802)
14 exp subcutaneous drug administration/ (72002)
15 exp infusion pump/ (2755)
16 exp infusion/ (26593)
17 (subcutaneous* or SubQ or sub-cutaneous* or pump or pumps or infuse or infused or infuses or infusing or infusion* or infuser*).ti,ab. (285686)
18 ((home adj3 therapy) or (home adj3 therapies) or (home adj3 tocoyl*) or (home-based adj3 therapy) or (home-based adj3 therapies) or (home-based adj3 tocoyl*)).ti,ab. (1578)
19 ((maintenance adj3 therapy) or (maintenance adj3 therapies) or (maintenance adj3 therapeutic) or (maintenance adj3 treatment*) or (maintenance adj3 tocoly*) or (supportive adj3 therapy) or (supportive adj3 therapies) or (supportive adj3 treatment*) or (supportive adj3 tocoyls*) or (outpatient adj3 therapy) or (outpatient adj3 therapies) or (outpatient* adj3 treatment*) or (outpatient* adj3 tocoly*)).ti,ab. (23804)
20 ((long-term adj therapy) or (long-term adj therapies) or (long-term adj therapeutic) or (long-term adj treatment*) or (long-term adj management) or (long-term adj tocoly*) or (longterm adj therapy) or (longterm adj therapies) or (longterm adj therapeutic) or (longterm adj treatment*) or (longterm adj management) or (longterm adj tocoly*)).ti,ab. (21021)
21 14 or 15 or 16 or 17 or 18 or 19 or 20 (392514)
22 13 and 21 (1163)
23 7 and 22 (188)
24 from 23 keep 1-188 (188)

CINAHL 2009 Dec 7

#	Query	Results
S24	S12 AND S23	32
S23	S13 OR S14 OR S16 OR S17 OR S18 OR S19 OR S22	30893
S22	(MH “Infusions, Parenteral+”)	4186
S21	S12 AND S20	32
S20	S13 OR S14 OR S15 OR S16 OR S17 OR S18 OR S19	30863
S19	TX (long-term W1 therapy) or (long-term W1 therapies) or (long-term W1 therapeutic) or (long-term W1 treatment*) or (long-term W1 management) or (long-term W1 tocoly*) or (longterm W1 therapy) or (longterm W1 therapies) or (longterm W1 therapeutic) or (longterm W1 treatment*) or (longterm W1 management) or (longterm W1 tocoly*))	4365
S18	TX (maintenance N3 therapy) or (maintenance N3 therapies) or (maintenance N3 therapeutic) or (maintenance N3 treatment*) or (maintenance N3 tocoyl*) or (supportive N3 therapy) or (supportive N3 therapies) or (supportive N3 treatment*) or (supportive N3 tocoly*) or (outpatient* N3 therapy) or (outpatient* N3 therapies) or (outpatient* N3 therapeutic) or (outpatient* N3 treatment*) or (outpatient* N3 tocoyl*)	4252
S17	TX (home N3 therapy) or (home N3 therapies) or (home N3 tocoly*) or (home-based N3 therapy) or (home-based N3 therapies) or (home-based N3 tocoly*)	2453
S16	TX subcutaneous* or SubQ or sub-cutaneous* or pump or pumps or infuse or infused or infuses or infusing or infusion* or infuser	21255
S15	(MH "Infusions, Parenteral")	276
S14	(MH "Infusion Pumps+")	1748
S13	(MH "Injections, Subcutaneous+")	1188
S12	S8 AND S11	63
S11	S9 or S10	206
S10	TX Terbutalin* or Brethaire or Brethine or Bricanyl or "BRN 2370513" or "EINECS 245-385-8" or "UNII-N8ONU3L3PG"	206
S9	(MH “Terbutaline”)	137
S8	S1 OR S2 OR S3 OR S4 OR S5 OR S6 OR S7	5924
S7	TX Tocolytic OR tocolysis	431
S6	“TX ((premature* N5 (uterine N2 contract*)) OR (pre-mature* N5 (uterine N2 contract*)) OR (preterm N5 (uterine N2 contract*)) OR (pre-term N5 (uterine N2 contract*)) OR early N5 (uterine N2 contract*))) or TX ((premature* N5 (uterus N2 contract*)) OR (pre-mature* N5 (uterus N2 contract*)) OR (preterm N5 (uterus N2 contract*)) OR (pre-term N5 (uterus N2 contract*)) OR (early N5 (uterus N2 contract*)))”	0
S5	TX (early N5 labor*) OR (early N5 labour*) OR (early N5 birth*) OR (early N5 deliver*)	1189
S4	TX ((preterm N5 labor*) or (preterm n5 labour*) or (preterm n5 birth*) or (preterm n5 deliver*)) or TX ((pre-term N5 labor*) or (pre-term n5 labour*) or (pre-term n5 birth*) or (pre-term n5 deliver*))	3453
S3	TX ((premature* N5 labor*) or (premature* n5 labour*) or (premature* n5 birth*) or (premature* n5 deliver*)) or TX ((pre-mature* N5 labor*) or (pre-mature* n5 labour*) or (pre-mature* n5 birth*) or (pre-mature* n5 deliver*))	2397
S2	TX PTL or PTB or RPTL	180
S1	(MH "Labor, Premature")	1539

Cochrane Library 2009, Issue 4 (updated to April 1, 2011)

ID	Search	Hits
#1	MeSH descriptor **Obstetric Labor, Premature** explode all trees	782
#2	(PTL or PTB or RPTL):ti,ab,kw	56
#3	(premature* NEAR/5 labor*) OR (premature* NEAR/5 labour*) OR (premature* NEAR/5 birth*) OR (premature* NEAR/5 deliver*):ti,ab,kw	1744
#4	(premature NEAR/5 (uterus NEAR/2 contract*)):ti,ab,kw or (premature NEAR/5 (uterine NEAR/2 contract*)):ti,ab,kw	15
#5	(pre NEXT mature* NEAR/5 labor*) OR (pre NEXT mature* NEAR/5 labour*) OR (pre NEXT mature* NEAR/5 birth*) OR (pre NEXT mature* NEAR/5 deliver*):ti,ab,kw	0
#6	((pre NEXT mature) NEAR/5 (uterus NEAR/2 contract*)):ti,ab,kw or ((pre NEXT mature) NEAR/5 (uterine NEAR/2 contract*)):ti,ab,kw	0
#7	(preterm NEAR/5 labor*) OR (preterm NEAR/5 labour*) OR (preterm NEAR/5 birth*) OR (preterm NEAR/5 deliver*):ti,ab,kw	1466
#8	(preterm NEAR/5 (uterus NEAR/2 contract*)):ti,ab,kw or (preterm NEAR/5 (uterine NEAR/2 contract*)):ti,ab,kw	15
#9	(pre NEXT term NEAR/5 labor*) OR (pre NEXT term NEAR/5 labour*) OR (pre NEXT term NEAR/5 birth*) OR (pre NEXT term NEAR/5 deliver*):ti,ab,kw	28
#10	((pre NEXT term) NEAR/5 (uterus NEAR/2 contract*)):ti,ab,kw or ((pre NEXT term) NEAR/5 (uterine NEAR/2 contract*)):ti,ab,kw	0
#11	(early NEAR/5 labor*) OR (early NEAR/5 labour*) OR (early NEAR/5 birth*) OR (early NEAR/5 deliver*) :ti,ab,kw	602
#12	(early NEAR/5 (uterus NEAR/2 contract*)):ti,ab,kw or (early NEAR/5 (uterine NEAR/2 contract*)):ti,ab,kw	9
#13	MeSH descriptor **Tocolysis** explode all trees	92
#14	(tocolysis or tocolytic*):ti,ab,kw	479
#15	(#**1** OR #**2** OR #**3** OR #**4** OR #**5** OR #**6** OR #**7** OR #**8** OR #**9** OR #**10** OR #**11** OR #**12** OR #**13** OR #**14**)	3147
#16	MeSH descriptor **Terbutaline** explode all trees	686
#17	(Terbutalin* or Brethaire or Brethine or Bricanyl or "BRN 2370513" or "EINECS 245-385-8" or "UNII-N8ONU3L3PG"):ti,ab,kw	1220

#18	(#16 OR #17)	1220
#19	MeSH descriptor **Injections, Subcutaneous** explode all trees	2896
#20	MeSH descriptor **Infusion Pumps** explode all trees	806
#21	MeSH descriptor **Home Infusion Therapy** explode all trees	41
#22	MeSH descriptor **Infusions, Parenteral** explode all trees	9362
#23	(subcutaneous* or SubQ or (sub NEXT cutaneous*) or pump or pumps or infuse or infused or infuses or infusing or infusion* or infuser*):ti,ab,kw	38786
#24	(home NEAR/3 therapy) or (home NEAR/3 therapies) or (home NEAR/3 tocoyl*) or ((home NEXT based) NEAR/3 therapy) or ((home NEXT based) NEAR/3 therapies) or ((home NEXT based) NEAR/3 tocoyl*):ti,ab,kw	657
#25	(maintenance NEAR/3 therapy) or (maintenance NEAR/3 therapies) or (maintenance NEAR/3 therapeutic) or (maintenance NEAR/3 treatment*) or (maintenance NEAR/3 tocoly*) or (supportive NEAR/3 therapy) or (supportive NEAR/3 therapies) or (supportive NEAR/3 treatment*) or (supportive NEAR/3 tocoyls*) or (outpatient NEAR/3 therapy) or (outpatient NEAR/3 therapies) or (outpatient* NEAR/3 treatment*) or (outpatient* NEAR/3 tocoly*):ti,ab,kw	6598
#26	(long NEXT term NEXT therapy) or (long NEXT term NEXT therapies) or (long NEXT term NEXT therapeutic) or (long NEXT term NEXT treatment*) or (long NEXT term NEXT management) or (long NEXT term NEXT tocoly*) or (longterm NEXT therapy) or (longterm NEXT therapies) or (longterm NEXT therapeutic) or (longterm NEXT treatment*) or (longterm NEXT management) or (longterm NEXT tocoly*):ti,ab,kw	3944
#27	(#**19** OR #**20** OR #**21** OR #**22** OR #**23** OR #**24** OR #**25** OR #**26**)	49538
#28	(#**15** AND #**18** AND #**27**)	51

51 records

DSR – 3
DARE – 1
CENTRAL – 41
HTA – 1
NHS EED – 5

CRD Databases – 2010 Jan 2

		Search	Matching Records
#	1	MeSH Obstetric Labor, Premature EXPLODE 1	146
#	2	PTL OR PTB OR RPT	13
#	3	(premature* NEAR labor*) OR (premature* NEAR labour*) OR (premature* NEAR birth*) OR (premature* NEAR deliver*)	153
#	4	(premature NEAR contract*)	11
#	5	(pre NEAR mature* NEAR labor*) OR (pre NEAR mature* NEAR labour*) OR (pre NEAR mature* NEAR birth*) OR (pre NEAR mature* NEAR deliver*)	1
#	6	pre NEAR mature NEAR contract*	0
#	7	(preterm NEAR labor*) OR (preterm NEAR labour*) OR (preterm NEAR birth*) OR (preterm NEAR deliver*)	342
#	8	preterm NEAR contract*	25
#	9	(pre NEAR term NEAR labor*) OR (pre NEAR term NEAR labour*) OR (pre NEAR term NEAR birth*) OR (pre NEAR term NEAR deliver*)	97
#	10	(pre NEAR term NEAR contract*)	7
#	11	(early NEAR labor*) OR (early NEAR labour*) OR (early NEAR birth*) OR (early NEAR deliver*)	281
#	12	early NEAR contract*	28
#	13	MeSH Tocolysis EXPLODE 1	14
#	14	tocolysis OR tocolytic*	67
#	15	MeSH Terbutaline EXPLODE 1 2	17
#	16	Terbutalin* OR Brethaire OR Brethine OR Bricanyl OR "BRN 2370513" OR "EINECS 245-385-8" OR "UNII-N8ONU3L3PG"	37
#	17	#1 OR #2 OR #3 OR #4 OR #5 OR #6 OR #7 OR #8 OR #9 OR #10 OR #11 OR #12 OR #13 OR #14	726
#	18	#15 OR #16	44
#	19	#17 AND #18	18
#	20	MeSH Injections, Subcutaneous EXPLODE 1	103
#	21	MeSH Infusion Pumps EXPLODE 1 2	89
#	22	MeSH Home Infusion Therapy EXPLODE 1 2	26
#	23	MeSH Infusions, Parenteral EXPLODE 1	359
#	24	subcutaneous* OR SubQ OR (sub NEAR cutaneous*) OR pump OR	1589

	pumps OR infuse OR infused OR infuses OR infusing OR infusion* OR infuser*	
# 25	(home NEAR therapy) OR (home NEAR therapies) OR (home NEAR tocoyl*)	280
# 26	(maintenance NEAR therapy) or (maintenance NEAR therapies) or (maintenance NEAR therapeutic) or (maintenance NEAR treatment*) or (maintenance NEAR tocoly*) or (supportive NEAR therapy) or (supportive NEAR therapies) or (supportive NEAR treatment*) or (supportive NEAR tocoyls*) or (outpatient NEAR therapy) or (outpatient NEAR therapies) or (outpatient* NEAR treatment*) or (outpatient* NEAR tocoly*)	0
# 27	(maintenance NEAR therapy) OR (maintenance NEAR therapies) OR (maintenance NEAR therapeutic) OR (maintenance NEAR treatment*) OR (maintenance NEAR tocoly*)	707
# 28	(supportive NEAR therapy) OR (supportive NEAR therapies) OR (supportive NEAR treatment*) OR (supportive NEAR tocoyls*)	350
# 29	(outpatient NEAR therapy) OR (outpatient NEAR therapies) OR (outpatient* NEAR treatment*) OR (outpatient* NEAR tocoly*)	991
# 30	#20 OR #21 OR #22 OR #23 OR #24 OR #25 OR #26 OR #27 OR #28 OR #29	3794
# 31	#19 AND #30	14

14 records

DARE - 7
NHS EED - 6
HTA - 1

Appendix B. Grey Literature Search

Search Dates: Nov. 27, 2009; Nov 29, 2009; Dec 31, 2009; Jan 2, 2010

Statistics
Canadian perinatal health report.
Public Health Agency of Canada, 2008
http://www.phac-aspc.gc.ca/publicat/2008/cphr-rspc/pdf/cphr-rspc08-eng.pdf
Alberta Reproductive Health: Pregnancies and Births Table Update, 2005
Alberta Health & Wellness; Alberta Perinatal Health Program, 2005
http://www.health.alberta.ca/documents/Reproductive-Health-2005.pdf

Systematic Reviews/Health Technology Assessments
Screening to prevent spontaneous preterm birth: systematic reviews of accuracy and effectiveness literature with economic modelling. Health Technol Assess 2009;13(43):1–627
Summary: http://www.hta.ac.uk/execsumm/summ1343.shtml
Full text: http://www.hta.ac.uk/fullmono/mon1343.pdf
Continuous subcutaneous terbutaline infusion for treatment of preterm labor. HAYES, Inc. Healthcare Technology Brief Publication. 2006
http://www.crd.york.ac.uk/CRDWeb/ShowRecord.asp?ID=32009100278
Subscription required
Management of preterm labor, 2000
Evidence report/Technology assessment no 18
http://www.ncbi.nlm.nih.gov/bookshelf/br.fcgi?book=hserta&part=A26682

Safety
Short-acting beta agonists and risk of myocardial ischaemia
Final SPC and PL wording agreed by PhVWP October 2009
http://www.hma.eu/fileadmin/dateien/Human_Medicines/CMD_h_/Product_Information/PhVWP_Recommendations/SABAs/CMDh-PhVWP-008-2009-Rev0a.pdf
ICU MEDICAL, INC. ORBIT 90" SUBCUTANEOUS INFUSION SET
Device leak, 2006
http://www.accessdata.fda.gov/scripts/cdrh/cfdocs/cfMAUDE/Detail.CFM?MDRFOI__ID=795454
CADD-MICRO TERBUTALINE PUMP SHOWER BAG
Injury, 2005
http://www.accessdata.fda.gov/scripts/cdrh/cfdocs/cfMAUDE/Detail.CFM?MDRFOI__ID=578910
Warning on use of terbutaline sulfate for preterm labor
JAMA 1998;279(1):9
http://jama.ama-assn.org/cgi/content/extract/279/1/9-a

Guidelines
Terbutaline pump for preterm labor
Aetna, 25 Aug 2009
http://www.aetna.com/cpb/medical/data/400_499/0468.html
Management of Labour
ICSI, May 2009
http://www.icsi.org/labor/labor__management_of__full_version__2.html
Obstetric and Medical Complications. In: Guidelines for perinatal care
ACOG, 2007
http://www.acog.org/publications/guidelinesForPerinatalCare/gpc-175.pdf
Management of preterm labor
http://www.acog.org/publications/pdfs/pb043.pdf
Tocolytic drugs for women in preterm labour
RCOG, 2002
http://www.rcog.org.uk/files/rcog-corp/GT1BTocolyticDrug2002revised.pdf

Conference Literature
Continuous Subcutaneous Terbutaline Therapy Improves Outcome in Pregnancies Complicated by Preterm Labour: Presented at ACOG. 11 May 2009
[Presentation title: Using Meta-Analysis Methodology to Evaluate Treatment of Preterm Labor. Abstract 79]
http://www.peerviewpress.com/continuous-subcutaneous-terbutaline-therapy-improves-outcome-pregnancies-complicated-preterm-labour-presented-acog

Economics
Ambrose S, Rhea DJ, Istwan NB, Collins A, Stanziano G. Clinical and economic outcomes of preterm labor management: inpatient vs outpatient. J Perinatol 2004;24(8):515–9.
Economic evaluation:
http://www.crd.york.ac.uk/CRDWeb/ShowRecord.asp?View=Full&ID=22004009091
Fleming A, Bonebrake R, Istwan N, Rhea D, Coleman S, Stanziano G. Pregnancy and economic outcomes in patients treated for recurrent preterm labor. J Perinatol 2004;24(4):223–7.
Economic evaluation:
http://www.crd.york.ac.uk/CRDWeb/ShowRecord.asp?View=Full&ID=22004006413
Morrison JC, Chauhan SP, Carroll CS, Sr., Bofill JA, Magann EF. Continuous subcutaneous terbutaline administration prolongs pregnancy after recurrent preterm labor. Am J Obstet Gynecol 2003;188(6):1460–5.
Economic evaluation :
http://www.crd.york.ac.uk/CRDWeb/ShowRecord.asp?View=Full&ID=22003009556
Lam F, Istwan NB, Jacques D, Coleman SK, Stanziano GJ. Managing perinatal outcomes: the clinical benefit and cost-effectiveness of pharmacologic treatment of recurrent preterm labor. Manag Care 2003;12(7):39–46.
Full text: http://www.managedcaremag.com/archives/0307/0307.peer_terbutaline.pdf
Economic evaluation:
http://www.crd.york.ac.uk/CRDWeb/ShowRecord.asp?View=Full&ID=22003006379

Lam F, Bergauer NK, Jacques D, Coleman SK, Stanziano GJ. Clinical and cost-effectiveness of continuous subcutaneous terbutaline versus oral tocolytics for treatment of recurrent preterm labor in twin gestations. J Perinatol 2001;21(7):444–50.
Economic evaluation:
http://www.crd.york.ac.uk/CRDWeb/ShowRecord.asp?View=Full&ID=22002006321

General/Miscellaneous
Note: Oregon Centre for Evidence-Based Policy appears to have done an evaluation on this topic, but I can’t find it on their Web site. You may wish to follow up with Mark Gibson, Deputy Director, gibsomar@ohsu.edu
Source info
http://docs.google.com/viewer?a=v&q=cache:0swFA5nHTqwJ:www.ecri.org/Documents/CERC/Gibson_Slides.pdf+Terbutaline+%2Bpreterm&hl=en&pid=bl&srcid=ADGEESiy9CSSqC5hjlZLxayoNVAQl9eIrd2xxfdEr86KQ-f_S6EVlmVX1HF3z_k9eThgxJc0N2Mr9thxd1UbF8WzukHgJszLh5oVxaKLX2Hy9tlDXcSOsNAY29X5E3yKXPmqAcVXLNvE&sig=AHIEtbSsSIQGqbdzwHPWmw2u5XB-ZwzDnA
Parenteral tocolytic therapy
Cigna Medical Coverage Policy, Sep 2009
http://www.cigna.com/customer_care/healthcare_professional/coverage_positions/medical/mm_0379_coverageposition_terbutaline_pump_and_tocolytic_therapy.pdf
After arrest of preterm labor, is continuous subcutaneous infusion of terbutaline effective treatment to prevent preterm birth?
http://www.infopoems.com/search/?query=terbutaline
Subscription required
Preterm labor
Medscape, 2009
http://emedicine.medscape.com/article/260998-overview
Determinants and prevention of low birth weight: a synopsis of the evidence
Institute for Health Economics, Dec 2008
http://www.ihe.ca/documents/IHE%20Report%20LowBirthWeight%20final.pdf
Tocoylsis with intravenous or subcutaneous terbutaline
Blue Cross, North Caroline, Dec 2008
http://www.bcbsnc.com/assets/services/public/pdfs/medicalpolicy/tocolysis_with_intravenous_or_subcutaneous_terbutaline.pdf
Born too soon: the continuing challenge of preterm labor and birth in the United States
J Midwifery Womens Health 2007;52(3):281-90.
PubMed: http://www.ncbi.nlm.nih.gov/pubmed/17467595
Preterm labour and birth: a survey of clinical practice regarding use of tocolytics, antenatal corticosteroids, and progesterone
JOGC, 2007
http://www.sogc.org/jogc/abstracts/full/200702_Obstetrics_1.pdf
Preterm labour
Merck, 2005
http://www.merck.com/mmpe/sec18/ch264/ch264f.html
Frequently asked questions on tocolytics

BJOG 2005;112 Suppl 1:94–6.
http://www.porodnice.cz/upload/predcasny-porod/literatura/Frequently_askd_Q_on_tocolytics.pdf
Sanchez-Ramos L, Huddleston J. The therapeutic value of maintenance tocolysis: an overview of the evidence. Clinics Perinatol 2003;30(4):841–54.
PubMed: http://www.ncbi.nlm.nih.gov/pubmed/14714925
Terbutaline studies, 2003
http://www.twinslist.org/tablest.html
Subcutaneous terbutaline pump in triplet gestation, In: Keith & Blickstien, editors. Triplet pregnancies and their consequences. Parthenon Publishing Group, 2002. p. 181–202.
http://books.google.com/books?hl=en&lr=&id=1aAtl4zt5SMC&oi=fnd&pg=PA181&dq=%22Terbutaline+pump%22+%2Bpreterm&ots=9rflddzapK&sig=PKMAevXgDOfTdcCcn7vj9Kasjy8#
Management of preterm labour
JAOA 2001;101(2 Suppl):S14–8
http://www.jaoa.org/cgi/reprint/101/2_suppl/14S.pdf
Discusses terbutaline pump therapy
Gyetvai K, Hannah M E, Hodnett E D, Ohlsson A. Tocolytics for preterm labor: a systematic review. Obstetrics and Gynecology 1999; 94(5 Part 2): 869–877
PubMed: http://www.ncbi.nlm.nih.gov/pubmed/10546776?dopt=Abstract
Critical Appraisal:
http://www.crd.york.ac.uk/CRDWeb/ShowRecord.asp?View=Full&ID=11999002142
Meirowitz N B, Ananth C V, Smulian J C, Vintzileos A M. Value of maintenance therapy with oral tocolytics: a systematic review. Journal of Maternal-Fetal Medicine 1999; 8(4): 177–83
PubMed: http://www.ncbi.nlm.nih.gov/pubmed/10406302?dopt=Abstract
Critical Appraisal:
http://www.crd.york.ac.uk/CRDWeb/ShowRecord.asp?View=Full&ID=11999004436
Shellhaas et al. Ambulatory management of preterm labour. Clin Obstet Gynecol 1998;41(3):491–502
http://www.ncbi.nlm.nih.gov/pubmed/9742347
FDA Advisory Cttee for Reproductive Health Drugs, 1998
http://www.fda.gov/OHRMS/DOCKETS/AC/98/transcpt/3407t1.rtf
Cowan M. Home care of the pregnant woman using terbutaline. MCN Am J Matern Child Nurs 1993;18(2):99–105.
http://journals.lww.com/mcnjournal/Citation/1993/03000/Home_Care_Of_the_Pregnant_Woman_Using_Terbutaline.8.aspx
Women's experiences using terbutaline pump therapy for the management of preterm labor (Dissertation, 1993)
http://nursinglibrary.org/Portal/main.aspx?pageid=4024&sid=9372
Comanagement of the patient on subcutaneous terbutaline pump therapy
J Nurse Midwifery;1991:36(3):204–8
http://www.sciencedirect.com/science?_ob=ArticleURL&_udi=B6T8N-4G0105T-15B&_user=10&_rdoc=1&_fmt=&_orig=search&_sort=d&_docanchor=&view=c&_searchStrId=1150447764&_rerunOrigin=scholar.google&_acct=C000050221&_version=1&_urlVersion=0&_userid=10&md5=1424a1cfe166517951e6cc1113e16f96

Regulatory
Canada

Licence No.: 7709 Type: Device Group Family

Licence Section			
**Device Class**	**First Issue Date**	**Licence Name**	
2	1999-07-12	SOF-SET INFUSION SET	
**Device Section**		**Identifier Section**	
**First Issue Date**	**Device Name**	**First Issue Date**	**Device Identifier**
1999-07-12	SOF-SET INFUSION SET	1999-07-12	MMT-112
		2002-05-06	MMT-111
		2009-03-10	MMT-111T
		2009-03-10	MMT-112T

Licence No.: 13631 Type: Single Device

Licence Section			
**Device Class**	**First Issue Date**	**Licence Name**	
2	1999-11-02	SOF-SET MICRO QR INFUSION SET	
**Device Section**		**Identifier Section**	
**First Issue Date**	**Device Name**	**First Issue Date**	**Device Identifier**
1999-11-02	SOF-SET MICRO QR	1999-11-02	MMT-320
		1999-11-02	MMT-321
		2009-03-10	MMT-320T
		2009-03-10	MMT-321T

Licence No.: 14508 Type: Device Group

Licence Section			
**Device Class**	**First Issue Date**	**Licence Name**	
2	1999-11-23	MINIMED SOF-SET ULTIMATE QR INFUSION SET	
**Device Section**		**Identifier Section**	
**First Issue Date**	**Device Name**	**First Issue Date**	**Device Identifier**
1999-11-23	MINIMED SOF-SET ULTIMATE QR	1999-11-23	MMT-315
		1999-11-23	MMT-316
		2009-03-10	MMT-315T
		2009-03-10	MMT-316T

Licence No.: 37241 Type: Single Device

Licence Section			
**Device Class**	**First Issue Date**	**Licence Name**	
2	2002-04-05	MINIMED PARADIGM SOF-SET ULTIMATE QR MODEL NO. 317, 318	
**Device Section**		**Identifier Section**	
**First Issue Date**	**Device Name**	**First Issue Date**	**Device Identifier**
2002-04-05	MINIMED PARADIGM SOF-SET ULTIMATE QR INFUSION SET	2002-04-05	MMT-317
		2002-04-05	MMT-318
		2009-03-16	MMT-317T
		2009-03-16	MMT-318T

Licence No.: 37244 Type: Single Device

Licence Section			
**Device Class**	**First Issue Date**	**Licence Name**	
2	2002-04-05	MINIMED PARADIGM SOF-SET ULTIMATE QR MODEL NO. 324, 325	
**Device Section**		**Identifier Section**	
**First Issue Date**	**Device Name**	**First Issue Date**	**Device Identifier**
2002-04-05	MINIMED PARADIGM SOF-SET ULTIMATE QR INFUSION SET	2002-04-05	MMT-324
		2002-04-05	MMT-325
		2009-03-16	MMT-325T

Licence No.: 11270 Type: Device Family

<table>
<tr><th colspan="4">Licence Section</th></tr>
<tr><th>Device Class</th><th>First Issue Date</th><th colspan="2">Licence Name</th></tr>
<tr><td>2</td><td>1999-09-02</td><td colspan="2">DISETRONIC CARTRIDGES FOR MICRODOSE INFUSION PUMPS</td></tr>
<tr><th colspan="2">Device Section</th><th colspan="2">Identifier Section</th></tr>
<tr><th>First Issue Date</th><th>Device Name</th><th>First Issue Date</th><th>Device Identifier</th></tr>
<tr><td rowspan="7">2005-07-06</td><td rowspan="7">DISETRONIC PLASTIC CARTRIDGES</td><td>2007-08-28</td><td>04567463001</td></tr>
<tr><td>2007-08-28</td><td>04567528001</td></tr>
<tr><td>2007-08-28</td><td>04923707001</td></tr>
<tr><td>2008-04-16</td><td>04854047001</td></tr>
<tr><td>2008-04-24</td><td>04949064001</td></tr>
<tr><td>2008-04-24</td><td>04949935001</td></tr>
<tr><td>2008-04-24</td><td>05206073001</td></tr>
</table>

Grey Literature Search: SRC
Search Date: April 8, 2010

Regulatory Information
FDA
Health Canada
Authorized Medicines for EU

Clinical Trial Registries
ClinicalTrials.gov
Current Controlled Trials
Clinical Study Results
WHO Clinical Trials

Abstracts and Conference Papers
Conference Papers Index
Scopus

Grants and Federally Funded Research
NIH RePORTER (a searchable database of federally funded biomedical research projects conducted at universities, hospitals, and other research institutions)
HSRPROJ (a database providing access to ongoing grants and contracts in health services research)

Other Miscellaneous Sources
Hayes, Inc. Health Technology Assessment
NY Academy of Medicine's Grey Literature Index

Appendix C. Scientific Information Packet Request

Requests for Scientific Information Packets (SIPs) were made from the following companies:

M Infusion Therapy
AAIPharma Inc.
Akorn, Inc.
Abraxis Pharmaceuticals (APP Pharmaceuticals)
AstraZeneca Pharmaceuticals, LP
Baxter Healthcare Corp
Becton, Dickinson and Company
Bedford Laboratories Inc.
BREG, Inc.
C.R. Bard, Inc.
Disetronic Medical Systems AG
Disetronic Medical Systems Inc.
Hikma Pharmaceuticals (USA) Ltd.
I-Flow Corporation
Impax Laboratories, Inc.
International Infusion, LLC (Intra Pump Infusion Systems)
Lannett Company, Inc.
MarCal Medical, Inc.
Medtronic Diabetes
Medtronic MiniMed, Inc.
Novartis Pharmaceuticals Corporation
RMS Medical Products
Roche Diagnostics
Sanofi Aventis US
Sorenson Medical Inc.
Tandem Medical Equipment Inc.
Teva Pharmaceuticals USA

Responses were received from the following companies:
AAIPharma Inc.
Abraxis Pharmaceuticals (APP Pharmaceuticals)
Baxter Healthcare Corp
BREG, Inc.
C.R. Bard, Inc.
Impax Laboratories, Inc.
International Infusion, LLC (Intra Pump Infusion Systems)
RMS Medical Products
Roche Diagnostics
Sanofi Aventis US

Appendix D. Screening, Data Extraction, Risk of Bias, and Applicability Forms

Level 1 Screening Form (Titles and Abstracts):

1. Does the record describe a study for which an abstract and/or a full-text article has been published in English?
    - No
    - Yes
    - Unsure

2. Does the record describe a review article?
    - No
    - Yes
    - Unsure

3. Does the record describe a single case study?
    - No
    - Yes
    - Unsure
    - N/A

4. Does the record describe a study that includes pregnant women >24 weeks and <37 weeks gestation?
    - No
    - Yes
    - Unsure
    - N/A

5. Does the record describe a study that includes pregnant women with arrested preterm labor?
    - No
    - Yes
    - Unsure
    - N/A

6. Does the record describe a study that includes at least one treatment group administered subcutaneous (SC) terbutaline by infusion pump as maintenance tocolytic therapy (i.e. not primary tocolytic therapy)?

- No
- Yes
- Unsure
- N/A

7. Has the study assessed at least one of the following outcomes?

Neonatal Health Outcomes: bronchopulmonary dysplasia, necrotizing enterocolitis, significant intraventricular hemorrhage (grade III/IV), periventricular leukomalacia, seizures, retinopathy of prematurity, sepsis, stillbirth, death within initial hospitalization, neonatal death.

Other Health Outcomes: gestational age at delivery, incidence of delivery at <28 weeks, <34 weeks and <37 weeks gestational age, prolongation of pregnancy, birthweight, need for assisted ventilation, need for oxygen per nasal cannula, NICU admission

Maternal Harms: pulmonary edema, heart failure, arrhythmia, myocardial infarction, refractory hypotension, hypokalemia, hyperglycemia, maternal withdrawal due to adverse effects, maternal discontinuation of therapy

Neonatal Harms: hypoglycemia, hypocalcemia, ileus

Pump Failure: missed doses, dislodgment, overdose

- No
- Yes
- Unsure
- N/A

8. Should this record be excluded for any other reason that has not yet been captured with the above questions? If yes, please describe that reason.

- No
- Yes
- N/A

Level 2 Screening Form (Full-text):

1. Does the record describe a study for which an abstract and/or a full-text article has been published in English?
    - No
    - Yes
    - Can't tell, abstract and/or full-text not available

2. Does the record describe a review article?
    - No
    - Yes

3. Does the record describe a single or multiple (individual) case reports?
    - No
    - Yes
    - N/A because record already excluded by a prior question

4. Does the record describe a study that includes pregnant women >24 weeks and <37 weeks gestation?
    - No
    - Yes
    - N/A because record already excluded by a prior question

5. Does the study include only women with ruptured membranes?
    - No
    - Yes
    - Data not reported
    - N/A because record already excluded by a prior question

6. Does the record describe a study that includes pregnant women with arrested preterm labor after primary tocolytic treatment?
    - No
    - Yes
    - N/A because record already excluded by a prior question

7. Has subcutaneous (SC) terbutaline by infusion pump been administered as a maintenance tocoloytic therapy in at least one treatment group (i.e. not primary tocolytic treatment)?

- No
- Yes
- N/A because record already excluded by a prior question

8. Has the study assessed at least one of the following outcomes?
   - Neonatal Health Outcomes: bronchopulmonary dysplasia, necrotizing enterocolitis, significant intraventricular hemorrhage (grade III/IV), periventricular leukomalacia, seizures, retinopathy of prematurity, sepsis, stillbirth, death within initial hospitalization, neonatal death
   - Other Health Outcomes: gestational age at delivery, incidence of delivery at <28 weeks, <34 weeks and <37 weeks gestational age, prolongation of pregnancy period, birthweight, need for assisted ventilation, need for oxygen per nasal cannula, NICU admission
   - Maternal Harms: pulmonary edema, heart failure, arrhythmia, myocardial infarction, refractory hypotension, hypokalemia, hyperglycemia, maternal withdrawal due to adverse effects, maternal discontinuation of therapy
   - Neonatal Harms: hypoglycemia, hypocalcemia, ileus
   - Harms or adverse events related to the pump device, but not necessarily terbutaline: for example missed doses, pump dislodgment, overdose or infection, allergic reaction or thrombosis at the infection site

- No
- Yes
- N/A because record already excluded by a prior question

9. Should this study be excluded for any other reason that has not yet been captured with above questions?

- No
- Yes. If yes, please indicate reason
- N/A because record already excluded by a prior question

Level 3 Screening Form (Further assessment of study design and outcomes for those citations that passed through Level 2 screening):

1. Which of the following categories of outcomes has the study assessed (check all that apply)?
    - Neonatal Health Outcomes: bronchopulmonary dysplasia, necrotizing enterocolitis, significant intraventricular hemorrhage (grade III/IV), periventricular leukomalacia, seizures, retinopathy of prematurity, sepsis, stillbirth, death within initial hospitalization, neonatal death
    - Other Health Outcomes: gestational age at delivery, incidence of delivery at <28 weeks, <32 weeks, <34 weeks and <37 weeks gestational age, prolongation of pregnancy period, birthweight, need for assisted ventilation, need for oxygen per nasal cannula, NICU admission
    - Maternal Harms: pulmonary edema, heart failure, arrhythmia, myocardial infarction, refractory hypotension, hypokalemia, hyperglycemia, maternal withdrawal due to adverse effects, maternal discontinuation of therapy
    - Neonatal Harms: hypoglycemia, hypocalcemia, ileus
    - Harms or adverse events related to the pump device, but not necessarily terbutaline: for example missed doses, pump dislodgment, overdose or infection, allergic reaction or thrombosis at the infection site
    - N/A – the study has not assessed any of the above outcomes
    - Long-term childhood outcomes such as childhood development, neurobehavioural testing, long-term lung function, long-term vision or other long-term childhood outcomes

Based on the answer to the above question, citations were directed to one of the subsequent Level 3 screening forms:

If option 5 was the only one chosen (i.e harm or adverse events related to the pump device):

a. Are incidence data (versus prevalence) available for any outcome related to pump failure?
    - No
    - Yes

If options 1, 2, 3, or 4 were the only ones chosen (i.e. maternal or neonatal outcomes):

a. Does the study include at least one comparison group receiving placebo, standard trea another intervention?
    - No
    - Yes

b. Please specify the study design:

- Randomized controlled trial
- Non-randomized controlled trial
- Prospective cohort
- Retrospective cohort
- Case-control
- Cross-sectional
- Other (please specify):
- N/A - because record already excluded by question 1

c. Does the study design allow for an evaluation of the effectiveness or harms of subcutaneous (SC) terbutaline by infusion pump as the sole maintenance tocoloytic therapy?
Note: study designs which are (treatment X + terbutaline pump vs. X alone) or (X + terbutaline pump vs. treatment X + treatment Y) are not to be excluded. Study designs that are (terbutaline pump + treatment X vs. terbutaline pump alone or in conjunction with treatment Y) are to be excluded (unless there is pump failure data)

- No
- Yes
- N/A - because record already excluded by question 1

If a combination of pump related outcomes and maternal/neonatal outcomes were chosen:

a. To be included in the review, either condition (1) and/or (2) below must be met:

(1) For outcomes related to pump failure incidence data (versus prevalence data) must be available

(2) For neonatal or other outcomes, maternal harms or neonatal harms, the study must:

- include at least one comparison group receiving placebo, standard treatment or another intervention **AND**
- be a controlled trial (randomized or non-randomized), a prospective or retrospective cohort study, a case-control study or a cross-sectional study **AND**
- allow for an evaluation of the effectiveness or harms of subcutaneous terbutaline by infusion pump as the sole maintenance tocolytic therapy (note: study designs which are (treatment X + terbutaline pump vs. X alone) or (X + terbutaline pump vs. treatment X + treatment Y) are to be included. Study designs that are (terbutaline pump + treatment X vs. terbutaline pump alone or in conjunction with treatment Y) are to be excluded (unless there is incident pump failure data, as above)

Is condition (1) and/or (2) above met?

- No
- Yes

b. Which of the following categories of outcomes has the study assessed AND met the above eligibility criteria (select all that apply)?

- Neonatal Health Outcomes: bronchopulmonary dysplasia, necrotizing enterocolitis, significant intraventricular hemorrhage (grade III/IV), periventricular leukomalacia, seizures, retinopathy of prematurity, sepsis, stillbirth, death within initial hospitalization, neonatal death
- Other Health Outcomes: gestational age at delivery, incidence of delivery at <28 weeks, <32 weeks, <34 weeks and <37 weeks gestational age, prolongation of pregnancy period, birthweight, need for assisted ventilation, need for oxygen per nasal cannula, NICU admission
- Maternal Harms: pulmonary edema, heart failure, arrhythmia, myocardial infarction, refractory hypotension, hypokalemia, hyperglycemia, maternal withdrawal due to adverse effects, maternal discontinuation of therapy
- Neonatal Harms: hypoglycemia, hypocalcemia, ileus
- Harms or adverse events related to the pump device, but not necessarily terbutaline: for example missed doses, pump dislodgment, overdose or infection, allergic reaction or thrombosis at the infection site

If option 7 has been chosen (long-term outcomes):

Please indicate which long-term outcomes have been assessed in the study (check all that apply)

- Childhood Development. Please provide details.
- Neurobehavioural Testing. Please provide details
- Long-term Lung Function. Please provide details
- Long-term Vision. Please provide details
- Other. Please describe

Risk of Bias Assessment

1. Are the treatment and comparison groups similar in terms of baseline characteristics and prognostic factors?
    - Yes
    - No. If no, please explain the differences
    - Unclear
    - N/A - there is no comparison group (studies of pump failure only)

2. Did participants in the treatment and comparison groups receive the same (or a similar distribution of) primary tocolytic to control their acute episode of preterm labor?
   - Yes
   - No. If no, please describe the differences.
   - Unclear (data not reported)
   - N/A - there is no comparison group (studies of pump failure only)

3. If this is an experimental study, were patients blinded to treatment allocation?
   - Yes
   - No
   - Unclear (data not reported)
   - N/A (not an experimental study)

4. If this is an experimental study, were healthcare providers blinded to treatment allocation?
   - Yes
   - No
   - Unclear (data not provided)
   - N/A (not an experimental study)

5. If this is an experimental study, were healthcare providers blinded to the frequency and intensity of maternal contractions? (Select all that apply)
   - At initiation of maintenance therapy with the subcutaneous terbutaline pump (at treatment allocation)
   - During maintenance therapy with the subcutaneous terbutaline pump
   - When assessing treatment outcomes (of interest to this review)
   - Health care providers were at no point blinded to the frequency and intensity of maternal contractions
   - Unclear (data not reported)
   - N/A (not an experimental study)

6. If this is an experimental study, was the outcome assessor blinded to treatment allocation?
   - Yes
   - No
   - Unclear (data not reported)
   - N/A (not an experimental study)

7. Was an intention-to-treat analysis conducted?
Note: An intention-to-treat (ITT) analysis aims to include all participants randomized into a trial irrespective of what happened subsequently. Indicate "yes" if participants were analyzed in the intervention groups to which they were assigned, regardless of the intervention they actually received. To receive a "yes" response, all participants must be included in the analysis (i.e. missing data has been imputed by some means).

- Yes
- No
- Unclear (data not reported)
- N/A (case series)

8. Was there either: i) a differential loss to followup between the compared groups; or ii) an overall high loss to followup?

- Yes. If yes, please provide details:
- No
- Unclear (data not reported)

9. Was the sample size adequate to determine a difference in outcomes between comparison groups or between pre and post intervention?

- Yes
- No
- Unclear
- N/A

10. Was there a differential level of care (e.g., home uterine contraction monitoring, education, nurse visits, individualized dosing schedules, other co-interventions) between the treatment and comparison groups?

- Yes
- No
- Unclear (data not reported)
- N/A - there is no comparison group (studies of pump failure only)

11. Are the study funders likely to have had any influence on study outcomes that might have biased the study results?

- Yes
- No
- Unclear (data not reported)

12. Is there any indication of selective outcome reporting?
Note: to assess selective outcome reporting, please compare the outcomes listed in the methods section of the report to the reported results. Indicate "yes" if all measured outcomes are accounted for in the results section, and are adequately reported.

- Yes. If yes, please describe:
- No
- Unclear

13. If multiple outcome assessors were used, is it likely there was high reliability in outcome assessment between all assessors? (e.g., inter-rater reliability testing was conducted and adequate)

- Yes
- No. If no, please describe
- Unclear (data not reported)
- N/A - multiple assessors were not used

14. Was compliance with the study protocol (i.e. treatment or comparator intervention) adequate in all study groups?

- Yes
- No. If no, please describe:
- Unclear (data not reported)

15. If this is a randomized controlled trial, was the allocation sequence adequately generated?
Note: Indicate "yes" if the method of randomization to treatment groups is likely to produce comparable groups, for example through use of a random number table or a computerized random number generator.

- Yes
- No
- Unclear (data not reported)
- N/A - this is not a randomized controlled trial

16. If this is a randomized controlled trial, was the process of concealing the random allocation sequence adequate?
Note: Indicate "yes" if a process was in place to adequately conceal future intervention allocations from study personnel, for example through pharmacy controlled randomization, or the use of sequentially numbered, sealed and opaque envelopes.

- Yes
- No
- Unclear (data not reported)
- N/A - this is not a randomized controlled trial

17. If this is a randomized controlled trial, at the time of study enrollment is there any indication that study personnel were able to predict future intervention assignments?
Note: Indicate "yes" if any reported baseline imbalances are likely to have resulted from study personnel selectively enrolling patients into the study based on their prediction of future intervention assignments.

- Yes
- No
- Unclear (data not reported)
- N/A - this is not a randomized controlled trial

18. If this is an observational study or a nonrandomized trial, is the sample population from which the comparison group(s) was drawn the same as the sample population from which the treatment group was drawn?

- Yes
- No. If no, please describe:
- Unclear
- N/A - this is not an observational study/nonrandomized trial or there is no comparison group (studies of pump failure only)

19. If this is an observational study or a nonrandomized trial, were appropriate methods undertaken to control for important confounders (e.g., matching)?

- Yes
- No
- Unclear
- N/A - this is not an observational study/nonrandomized trial or there is no comparison group (studies of pump failure only)

20. If this is a retrospective study that used multiple data sources, is it likely there was consistency in outcome definition across those data sources?

- Yes
- No. If no, please describe:
- Unclear (data not reported)
- N/A - this is not a retrospective study that uses multiple data sources

21. For studies assessing maternal or neonatal harms: If the harm outcomes assessed in the study are not generally known to have standard definitions, then were these harms pre-defined using standardized or precise definitions?

- Yes
- No
- Unclear (data not reported)
- N/A - this is not a study assessing maternal or neonatal harms
- N/A - this study measured harms with standardized definitions. If so, please specify these harms

22. If this is a study assessing maternal or neonatal harms, was the mode of harms collection specified as active (versus passive)?

Note: Active harms assessment is when participants are asked about the occurrence of specific harms in structured questionnaires or interviews or pre-defined laboratory or diagnostic tests, usually performed at pre-specified time intervals.

Passive assessment of harms occurs when study participants spontaneously report (on their own initiative) or are allowed to report harmful events not probed with active ascertainment.

- Yes
- No
- Unclear (data not reported)
- N/A - this is not a study assessing maternal or neonatal harms

23. If this is a study assessing maternal or neonatal harms, did the report specify who collected harms data, including their training and background?

- Yes
- No
- Unclear
- N/A - this is not a study assessing maternal or neonatal harms

24. Were the subjects who were included in the study representative of the source population? For instance, subjects would be representative if the entire source population was recruited for the study, if a sample of consecutive patients was recruited, or if a random sample was obtained.

- Yes. Please explain
- No. Please explain
- Unclear (e.g. sampling methodology is not reported). Please explain

25. Were the primary outcomes in the study defined by either prespecified or standardized clinical definitions?

- Yes. Please list what these outcomes are and any definitions provided in paper
- No. Please list what these outcomes are
- Unclear
- N/A - the study does not list any primary outcomes

Overall Risk of Bias (study quality) Assessment

For each outcome assessed within this study, please provide an overall assessment of the risk of bias associated with measurement of that outcome based on your answers to the above questions.

26. Please specify study outcome:

- *Bronchopulmonary dysplasia*
- *Necrotizing enterocolitis*
- *Intraventricular hemorrhage*
- *Periventricular luokomalacia*
- *Seizures*
- *Retinopathy of prematurity*
- *Sepsis*
- *Stillbirth*
- *Death within initial hospitalization*
- *Neonatal death*
- *Gestational age at delivery*
- *Incidence of delivery at various gestational ages*
- *Prolongation of pregnancy*
- *Birthweight*
- *Need for assisted ventilation*
- *need for oxygen per nasal cannula*
- *NICU admission*
- *maternal pulmonary edema*
- *maternal heart failure*
- *maternal arrhythmia*
- *maternal myocardial infarction*
- *maternal refractory hypotension*
- *maternal hypokalemia*
- *maternal hyperglycemia*
- *maternal mortality*
- *maternal withdrawal*
- *maternal discontinuation of therapy*
- *neonatal hypoglycemia*
- *neonatal hypocalcemia*
- *neonatal ileus*
- *missed doses (pump failure)*
- *pump dislodgment*
- *overdose (pump failure)*
- *other pump failure outcome (please specify)*
- *PPI*
- *Ratio birthweight/GA at delivery*

Overall risk of bias assessment

Please select one of either good, fair or poor and provide an explanation for your response.

- Good (low risk of bias)
- Fair
- Poor (high risk of bias)
- Please explain your response

Applicability Assessment Form:

POPULATION

Please consider each of the following criteria and indicate which, if any, might limit applicability:

1. Inclusion/exclusion criteria

A condition that might limit applicability is narrow eligibility criteria

- Yes. If yes, please explain:
- No
- Unclear

2. Exclusion rate

A condition that might limit applicability is a high exclusion rate

- Yes. If yes, please explain:
- No
- Unclear (data not reported)

3. Demographic characteristics

A condition that might limit applicability is a large difference between demographics of study population and that of patients in the community

- Yes. If yes, please explain:
- No
- Unclear

4. Run in period, considering attrition before randomization and reasons (if reported)

A condition that might limit applicability is a run in period with high-exclusion rate for non-adherence or side effects

- Yes. If yes, please explain:
- No
- Unclear
- N/A - non-randomized study

INTERVENTION

Please consider each of the following criteria and indicate which, if any, might limit applicability

5. Dose and duration

Condition that might limit applicability are doses or treatment schedules not reflected in current practice.

- Yes. If yes, please explain
- No
- Unclear

6. Co-interventions

A condition that might limit applicability is the delivery of co-interventions that are likely to modify effectiveness of therapy.

- Yes. If yes, please explain:
- No
- Unclear

7. Level of care

A condition that might limit applicability is a level of care or visit frequency not used or likely to be feasible in typical practice.

- Yes. If yes, please explain:
- No
- Unclear

8. Training provided regarding pump administration

A condition that might limit applicability is the provision of intensive education that is not likely to be feasible in typical practice.

- Yes. If yes, please explain:
- No
- Unclear

COMPARISON

Please consider each of the following criteria and indicate which, if any, might limit applicability

9. Dose and schedule of comparator

A condition that is likely to limit applicability is an inadequate dose of comparison therapy

- Yes. If yes, please explain:
- No
- Unclear
- N/A - no comparison group (study of pump failure only) or comparison group receive[illegible] treatment/placebo

10. Whether comparator is the best available alternative to terbutaline pump

A condition that might limit applicability is the use of a sub-standard alternative therapy

- Yes. If yes, please explain:
- No
- Unclear
- N/A - no comparison group (study of pump failure only) or comparison group received no treatment/placebo

OUTCOMES

Please consider each of the following criteria and indicate which, if any, might limit applicability

11. Clinical benefits on relative and absolute scale

Conditions that might limit applicability are the assessment of surrogate rather than clinical outcomes or failure to measure most important outcomes.

- Yes. If yes, please explain:
- No
- Unclear

12. Individual harms and how defined, on relative and absolute scale

A condition that might limit applicability is failure to distinguish minor from serious adverse effects.

- Yes. If yes, please explain:
- No
- Unclear
- N/A - this is not a study of individual harms

TIMING OF OUTCOMES MEASUREMENT

Please consider each of the following criteria and indicate which, if any, might limit applicability

13. Timing of followup

A condition that might limit applicabiity is if followup is too short to detect important benefits or harms.

- Yes. If yes, please explain:
- No
- Unclear

SETTING

Please consider each of the following criteria and indicate which, if any, might limit applicability

14. Geographic setting

A condition that might limit applicability is if within the study setting standards of care differ markedly from the setting of interest.

- Yes. If yes, please explain:
- No
- Unclear

15. Clinical setting

A condition that might limit applicabiity is if the study setting serves a specialty population or level of care that differs importantly from that seen in standard tertiary care settings.

- Yes. If yes, please explain:
- No
- Unclear

Appendix E. Excluded Studies

Level 1 exclusions (n=215):

Adamcova M, Kokstein Z, Palicka V, et al. Cardiac troponin T in neonates after acute and long-term tocolysis. Biology of the Neonate 2000;78(4):288-92.

Adzick NS, Sutton LN, Crombleholme TM, et al. Successful fetal surgery for spina bifida. Lancet 1998;352(9141):1675-6.

Anderson KE, Bengtsson LP, Gustafson I, et al. The relaxing effect of terbutaline on the human uterus during term labor. American Journal of Obstetrics & Gynecology 1975 Mar 1;121(5):602-9.

Anotayanonth S, Subhedar NV, Garner P, et al. Betamimetics for inhibiting preterm labor. Cochrane Database Syst Rev 2004;(4):CD004352.

Arias F. Intrauterine resuscitation with terbutaline: a method for the management of acute intrapartum fetal distress. Am J Obstet Gynecol 1978 May 1;131(1):39-43.

Athanassiou AM, Turrentine MA. Myocardial infarction and coronary artery dissection during pregnancy associated with type IV Ehlers-Danlos syndrome. American Journal of Perinatology 1996 Apr;13(3):181-3.

Ayromlooi J, Desiderio DM, Tobias M. Terbutaline-induced changes in ovine, fetal and maternal hemodynamics, and fetal electroencephalogram. Pediatric Pharmacology 1981;1(4):321-30.

Ayromlooi J, Desiderio DM, Tobias MD, et al. Isoxsuprine-induced changes in maternal and fetal hemodynamics and fetal electroencephalogram. Drug Development Research 1982;2(1):55-64.

Barnett AH, Stubbs SM, Mander AM. Management of premature labor in diabetic pregnancy. Diabetologia 1980;18(5):365-8.

Bergman B, Bokstrom H, Borga O, et al. Transfer of terbutaline across the human placenta in late pregnancy. Eur J Respir Dis Suppl 1984;134:81-6.

Berkman ND, Thorp JM, Jr., Lohr KN, et al. Tocolytic treatment for the management of preterm labor: a review of the evidence. Am J Obstet Gynecol 2003 Jun;188(6):1648-59.

Bernstein IM, Catalano PM. Ketoacidosis in pregnancy associated with the parenteral administration of terbutaline and betamethasone. A case report. Journal of Reproductive Medicine 1990 Aug;35(8):818-20.

Besinger RE, Niebyl JR. The safety and efficacy of tocolytic agents for the treatment of preterm labor. Obstet Gynecol Surv 1990 Jul;45(7):415-40.

Besinger RE, Niebyl JR, Keyes WG, et al. Randomized comparative trial of indomethacin and ritodrine for the long-term treatment of preterm labor. American Journal of Obstetrics & Gynecology 1991 Apr;164(4):981-6.

Bhat N, Seifer D, Hensleigh P. Paradoxical response to intravenous terbutaline. American Journal of Obstetrics and Gynecology 1985;153(3):310-1.

Bhide A. Oral nifepidine versus subcutaneous terbutaline tocolysis for external cephalic version: A double-blind randomised trial - Commentary. BJOG : An International Journal of Obstetrics and Gynaecology 2009;116(1):80-1.

Bivins HA, Jr., Newman RB, Fyfe DA, et al. Randomized trial of oral indomethacin and terbutaline sulfate for the long-term suppression of preterm labor. Am J Obstet Gynecol 1993 Oct;169(4):1065-70.

Blackstone J. Effects of tocolytics on the fetal heart. Journal of Maternal -Fetal Investigation 1996;6(3):158-62.

Bloss JD, Hankins GD, Gilstrap LC, III, et al. Pulmonary edema as a delayed complication of ritodrine therapy. A case report. Journal of Reproductive Medicine 1987 Jun;32(6):469-71.

Boehm FH, Lombardi SJ, Rosemond RL. Immediate cerclage following delivery of one nonviable twin: A report of three cases. Journal of Reproductive Medicine for the Obstetrician and Gynecologist 1992;37(12):986-8.

Boog G, Brahim MB, Gandar R. Beta-mimetic drugs and possible prevention of respiratory distress syndrome. Br J Obstet Gynaecol 1975 Apr;82(4):285-8.

Borberg C, Gillmer MD, Beard RW, et al. Metabolic effects of beta-sympathomimetic drugs and dexamethasone in normal and diabetic pregnancy. Br J Obstet Gynaecol 1978 Mar;85(3):184-9.

Braden GL, von Oeyen PT, Germain MJ, et al. Ritodrine- and terbutaline-induced hypokalemia in preterm labor: mechanisms and consequences. Kidney International 1997 Jun;51(6):1867-75.

Bremme K, Eneroth P, Carsjo BM, et al. Blood pressure regulation in third-trimester pregnant women receiving tocolytic terbutaline infusion. European Journal of Obstetrics, Gynecology, & Reproductive Biology 1986 Oct;23(1-2):53-60.

Bremme K, Eneroth P, Nordstrom L, et al. Effects of infusion of the beta-adrenoceptor agonist terbutaline on serum magnesium in pregnant women. Magnesium 1986;5(2):85-94.

Bricker L, Peden H, Tomlinson AJ, et al. Titrated low-dose vaginal and/or oral misoprostol to induce labor for prelabor membrane rupture: A randomised trial. BJOG : An International Journal of Obstetrics and Gynaecology 2008;115(12):1503-11.

Brittain C, Carlson JW, Gehlbach DL, et al. A case report of massive vulvar edema during tocolysis for preterm labor. American Journal of Obstetrics and Gynecology 1991;165(2):420-2.

Brown HL, Hiett AK, Britton KA, et al. Tocolytic treatment for preterm contractions with and without cervical changes. American Journal of Perinatology 1997 Aug;14(7):405-9.

Brown SM, Tejani NA. Terbutaline sulfate in the prevention of recurrence of premature labor. Obstetrics & Gynecology 1981 Jan;57(1):22-5.

Cabar FR, Bittar RE, Gomes CM, et al. [Atosiban as a tocolytic agent: a new proposal of a therapeutic approach]. [Portuguese]. Revista Brasileira de Ginecologia e Obstetricia 2008 Feb;30(2):87-92.

Caritis SN. Treatment of preterm labor. A review of the therapeutic options. Drugs 1983 Sep;26(3):243-61.

Caritis SN, Darby MJ, Chan L. Pharmacologic treatment of preterm labor. Clinical Obstetrics and Gynecology 1988;31(3):635-51.

Caritis SN, Edelstone DI, Mueller-Heubach E. Pharmacologic inhibition of preterm labor. Am J Obstet Gynecol 1979 Mar 1;133(5):557-78.

Caritis SN, Morishima HO, Stark RI, et al. Effects of terbutaline on the pregnant baboon and fetus. Obstetrics & Gynecology 1977 Jul;50(1):56-60.

Caritis SN, Mueller-Heubach E, Morishima HO, et al. Effect of terbutaline on cardiovascular state and uterine blood flow in pregnant ewes. Obstetrics & Gynecology 1977 Nov;50(5):603-6.

Caritis SN, Toig G, Heddinger LA, et al. A double-blind study comparing ritodrine and terbutaline in the treatment of preterm labor. American Journal of Obstetrics & Gynecology 1984 Sep 1;150(1):7-14.

Carlan SJ, Dent JM, Huckaby T, et al. The effect of epidural anesthesia on safety and success of external cephalic version at term. Anesthesia and Analgesia 1994;79(3):525-8.

Carstensen MH, Bahnsen J, Sterzing E. [Tocolysis with beta sympathomimetics alone or combined with the calcium antagonist verapamil? Influence of verapamil on the fetus]. Geburtshilfe Frauenheilkd 1983 Aug;43(8):481-5.

Chandiramani M, Shennan A. Preterm labor: update on prediction and prevention strategies. Curr Opin Obstet Gynecol 2006 Dec;18(6):618-24.

Chauhan SP, Rutherford SE, Hess LW, et al. Prophylactic intrapartum amnioinfusion for patients with oligohydramnios. A prospective randomized study. Journal of Reproductive Medicine 1992 Sep;37(9):817-20.

Chimura T, Mitsui T, Inoue K. [Beta 1-, beta 2-effects of ritrodine and terbutaline in the treatment of preterm labor (author's transl)]. [Japanese]. Nippon Sanka Fujinka Gakkai Zasshi - Acta Obstetrica et Gynaecologica Japonica 1981 Oct;33(10):1629-34.

Chung M-Y, Huang C-B. Hyperkalemia in premature infants weighing less than 1,500 grams. Clinical Neonatology 1999;6(1):1-5.

Collaris R, Tan PC. Oral nifepidine versus subcutaneous terbutaline tocolysis for external cephalic version: a double-blind randomised trial. BJOG : An International Journal of Obstetrics & Gynaecology 2009 Jan;116(1):74-80.

Conde-Agudelo A, Romero R, Kusanovic JP. Nifedipine in the management of preterm labor: A systematic review and metaanalysis. American Journal of Obstetrics and Gynecology 2011;(2):134.

Cotton DB, Strassner HT, Hill LM, et al. Comparison of magnesium sulfate, terbutaline and a placebo for inhibition of preterm labor. A randomized study. J Reprod Med 1984 Feb;29(2):92-7.

Cowan M. Home care of the pregnant woman using terbutaline. [Review] [41 refs]. MCN, American Journal of Maternal Child Nursing 1993 Mar;18(2):99-105.

Creasy RK. Preterm birth prevention: where are we? Am J Obstet Gynecol 1993 Apr;168(4):1223-30.

Dagbjartsson A, Herbertsson G, Stefansson TS, et al. Beta-adrenoceptor agonists and hypoxia in sheep fetuses. Acta Physiologica Scandinavica 1989 Oct;137(2):291-9.

Davies AE, Robertson MJS. Pulmonary oedema after the administration of intravenous salbutamol and ergometrine. Case report. British Journal of Obstetrics and Gynaecology 1980;87(6):539-41.

De Albuquerque PB, Spallicci MDB, Zugaib M. The control of premature labor. University Hospital of the Sao Paulo University. [Portuguese]. Revista Brasileira de Medicina 2002;59(4):277-82.

De VM, Porto M, Major CA, et al. Tocolysis in advanced preterm labor: Impact on neonatal outcome. American Journal of Perinatology 1995;12(4):294-8.

Dodd JM, Crowther CA, Dare MR, et al. Oral betamimetics for maintenance therapy after threatened preterm labor. [Review] [45 refs]. Cochrane Database of Systematic Reviews 2006;(1):CD003927, 2006.

Domenichini Y, Thoulon JM. The cardio-vascular complications of the use of beta-mimetic drugs in obstetrics. [French]. Journal de Gynecologie Obstetrique et Biologie de la Reproduction 1982;11(7):861-7.

Egarter CH, Husslein PW, Rayburn WF. Uterine hyperstimulation after low-dose prostaglandin E2 therapy: tocolytic treatment in 181 cases. American Journal of Obstetrics & Gynecology 1990 Sep;163(3):794-6.

Ekelund L, Enhorning G. Glucocorticoids and beta-adrenergic-receptor agonists: their combined effect on fetal rabbit lung surfactant. American Journal of Obstetrics & Gynecology 1985 Aug 15;152(8):1063-7.

El-Sayed Y, Pullen K, Riley ET, et al. Randomized comparison of intravenous nitroglycerin and subcutaneous terbutaline for external cephalic version under tocolysis. Am J Obstet Gynecol 2004 Dec;191(6):2051-5.

Elliott JP. Management of high-order multiple gestation. [Review] [46 refs]. Clinics in Perinatology 2005;32(2):387-402.

Elliott JP, Radin TG. The effect of corticosteroid administration on uterine activity and preterm labor in high order multiple gestations. Obstetrics and Gynecology 1995;85(2):250-4.

Epstein MF, Nicholls E, Stubblefield PG. Neonatal hypoglycemia after beta-sympathomimetic tocolytic therapy. J Pediatr 1979 Mar;94(3):449-53.

Eronen M. The hemodynamic effects of antenatal indomethacin and a beta-sympathomimetic agent on the fetus and the newborn: a randomized study. Pediatr Res 1993 Jun;33(6):615-9.

Eronen M, Pesonen E, Kurki T, et al. The effects of indomethacin and a beta-sympathomimetic agent on the fetal ductus arteriosus during treatment of premature labor: a randomized double-blind study. Am J Obstet Gynecol 1991 Jan;164(1 Pt 1):141-6.

Faidley CK, Dix PM, Morgan MA, et al. Electrocardiographic abnormalities during ritodrine administration. Southern Medical Journal 1990 May;83(5):503-6.

Ferguson II JE, Hensleigh PA, Kredenster D. Adjunctive use of magnesium sulfate with ritodrine for preterm labor tocolysis. American Journal of Obstetrics and Gynecology 1984;148(2):166-71.

Fernandez CO, Bloom SL, Smulian JC, et al. A randomized placebo-controlled evaluation of terbutaline for external cephalic version. Obstetrics & Gynecology 1997 Nov;90(5):775-9.

Fiascone JM, Rhodes TT, Grandgeorge SR, et al. Bronchopulmonary dysplasia: A review for the pediatrician. Current Problems in Pediatrics 1989;19(4):169-227.

Fletcher SE, Fyfe DA, Case CL, et al. Myocardial necrosis in a newborn after long-term maternal subcutaneous terbutaline infusion for suppression of preterm labor.[see comment]. American Journal of Obstetrics & Gynecology 1991 Nov;165(5 Pt 1):1401-4.

Fox MD, Allbert JR, McCaul JF, et al. Neonatal morbidity between 34 and 37 weeks' gestation. J Perinatol 1993 Sep;13(5):349-53.

Fox NS, Gelber SE, Kalish RB, et al. Contemporary practice patterns and beliefs regarding tocolysis among U.S. maternal-fetal medicine specialists. Obstetrics and Gynecology 2008;112(1):42-7.

Freda MC. Commentary on Home care of the pregnant woman using terbutaline [original article by Cowan M appears in MCN 1993;18(2):99-105]. AWHONN's Women's Health Nursing Scan 1993 Nov;7(6):20-1.

Freysz H, Willard D, Lehr A, et al. A long term evaluation of infants who received a beta-mimetic drug while in utero. J Perinat Med 1977;5(2):94-9.

Fuchs F. Prevention of premature birth. Clin Perinatol 1980 Mar;7(1):3-15.

Gemelli M, De LF, Manganaro R, et al. Transient electrocardiographic changes suggesting myocardial ischaemia in newborn infants following tocolysis with beta-sympathomimetics. Eur J Pediatr 1990 Jul;149(10):730-3.

Gjerdingen DK. Premature labor, Part II: Management. J Am Board Fam Pract 1992 Nov;5(6):601-15.

Glock JL, Morales WJ. Efficacy and safety of nifedipine versus magnesium sulfate in the management of preterm labor: a randomized study. American Journal of Obstetrics & Gynecology 1993 Oct;169(4):960-4.

Graber EA. Dilemmas in the pharmacological management of preterm labor. Obstet Gynecol Surv 1989 Jul;44(7):512-7.

Graham G, Baxi L, Tharakan T. Laparoscopic cholecystectomy during pregnancy: A case series and review of the literature. Obstetrical and Gynecological Survey 1998;53(9):566-74.

Guernsey BG, Villarreal Y, Snyder MD, et al. Pulmonary edema associated with the use of betamimetic agents in preterm labor. American Journal of Hospital Pharmacy 1981 Dec;38(12):1942-8.

Guilliams S, Held B. Contemporary management and conduct of preterm labor and delivery: a review. Obstet Gynecol Surv 1979 Mar;34(3):248-55.

Guinn DA, Goepfert AR, Owen J, et al. Management options in women with preterm uterine contractions: a randomized clinical trial. American Journal of Obstetrics & Gynecology 1997 Oct;177(4):814-8.

Gyetvai K, Hannah ME, Hodnett ED, et al. Tocolytics for preterm labor: a systematic review. Obstet Gynecol 1999 Nov;94(5 Pt 2):869-77.

Haas DM, Imperiale TF, Kirkpatrick PR, et al. Tocolytic therapy: a meta-analysis and decision analysis. Obstet Gynecol 2009 Mar;113(3):585-94.

Hallak M, Moise K, Jr., Lira N, et al. The effect of tocolytic agents (indomethacin and terbutaline) on fetal breathing and body movements: a prospective, randomized, double-blind, placebo-controlled clinical trial. Am J Obstet Gynecol 1992 Oct;167(4 Pt 1):1059-63.

Han S, Crowther CA, Moore V. Magnesium maintenance therapy for preventing preterm birth after threatened preterm labour. [Review] [27 refs][Update of Cochrane Database Syst Rev. 2000;(2):CD000940; PMID: 10796222]. Cochrane Database of Systematic Reviews 2010;(7):CD000940 . [PMID: 20614423].

Hawkins JL. Epidural analgesia for labor and delivery. New England Journal of Medicine 2010;(16):1503-10.

Herbst A, Nilsson C. Diagnosis of early preterm labor. BJOG : An International Journal of Obstetrics and Gynaecology 2006;113(SUPPL. 3):60-7.

Herbst A, Wide-Swensson D, Ingemarsson I. Significant delay of birth in advanced preterm labor at 23 gestational weeks with an oxytocin antagonist. European Journal of Obstetrics, Gynecology, & Reproductive Biology 2003 May 1;108(1):109-10.

Herskowitz A, Herskowitz B. Acute paraparesis due to terbutaline sulfate. South Med J 2002 Feb;95(2):275-6.

Higby K, Xenakis EM, Pauerstein CJ. Do tocolytic agents stop preterm labor? A critical and comprehensive review of efficacy and safety. Am J Obstet Gynecol 1993 Apr;168(4):1247-56.

Hill WC. Risks and complications of tocolysis. Clin Obstet Gynecol 1995 Dec;38(4):725-45.

Hudgens DR, Conradi SE. Sudden death associated with terbutaline sulfate administration.[see comment]. American Journal of Obstetrics & Gynecology 1993 Jul;169(1):120-1.

Hueston WJ. Prevention and treatment of preterm labor. American Family Physician 1989;40(5):139-46.

Hueston WJ. A critical assessment of preterm labor prevention strategies. J Fam Pract 1992 Jul;35(1):81-9.

Iams JD, Johnson FF, Creasy RK. Prevention of preterm birth. Clin Obstet Gynecol 1988 Sep;31(3):599-615.

Ibrahim H, Kahn E, Harper RG, et al. Erythropoietin (EPO) levels in fetal rats after ritodrine and terbutaline administration. Biochemical Medicine and Metabolic Biology 1994;52(2):128-31.

Ingemarsson I. Pharmacology of tocolytic agents. Clin Obstet Gynaecol 1984 Aug;11(2):337-51.

Ingemarsson I, Arulkumaran S, Kottegoda SR. Complications of beta-mimetic therapy in preterm labor. Aust N Z J Obstet Gynaecol 1985 Aug;25(3):182-9.

Jacquemyn Y. Use of tocolytics: What is the benefit of gaining even more time? BJOG : An International Journal of Obstetrics and Gynaecology 2006;113(SUPPL. 3):78-80.

Jiang J, Hu J, Bai C. Role of aquaporin and sodium channel in pleural water movement. Respiratory Physiology & Neurobiology 2003 Dec 16;139(1):83-8.

Johnson P. Suppression of preterm labor: Current concepts. Drugs 1993;45(5):684-92.

Johnson WL, Harbert GM, Martin CB. Pharmacologic control of uterine contractility. In vitro human and in vivo monkey studies. Am J Obstet Gynecol 1975 Oct 15;123(4):364-75.

Jovanovic R. Serial serum potassium and glucose levels during treatment of premature labor with oral terbutaline. International Journal of Gynaecology & Obstetrics 1985 Oct;23(5):399-404.

Kanai M, Davies P, von AD, et al. Lung growth induced by prenatal tracheal occlusion and its modifying factors: A study in the rat model of congenital diaphragmatic hernia. Journal of Pediatric Surgery 2001;36(2):251-9.

Kast A, Hermer M. Beta-adrenoceptor tocolysis and effects on the heart of fetus and neonate: A review. Journal of Perinatal Medicine 1993;21(2):97-106.

Keirse MJ. A survey of tocolytic drug treatment in preterm labor. British Journal of Obstetrics & Gynaecology 1984 May;91(5):424-30.

Keirse MJ. New perspectives for the effective treatment of preterm labor. Am J Obstet Gynecol 1995 Aug;173(2):618-28.

King JF, Grant A, Keirse MJ, et al. Beta-mimetics in preterm labor: an overview of the randomized controlled trials. Br J Obstet Gynaecol 1988 Mar;95(3):211-22.

Kitano Y, Kanai M, Davies P, et al. BAPS prize-1999: Lung growth induced by prenatal tracheal occlusion and its modifying factors: a study in the rat model of congenital diaphragmatic hernia. Journal of Pediatric Surgery 2001 Feb;36(2):251-9.

Kobayashi H, Mizutani S, Wright JW. Placental leucine aminopeptidase- and aminopeptidase A-deficient mice offer insight concerning the mechanisms underlying preterm labor and preeclampsia. Journal of Biomedicine and Biotechnology 2011 Article Number: 286947

Kosasa TS, Busse R, Wahl N, et al. Long-term tocolysis with combined intravenous terbutaline and magnesium sulfate: a 10-year study of 1000 patients. Obstetrics & Gynecology 1994 Sep;84(3):369-73.

Kramer MS. Preventing preterm birth: are we making any progress? Yale J Biol Med 1997 May;70(3):227-32.

Kramer WB, Saade GR, Belfort M, et al. A randomized double-blind study comparing the fetal effects of sulindac to terbutaline during the management of preterm labor. Am J Obstet Gynecol 1999 Feb;180(2 Pt 1):396-401.

Kuczkowski KM, Benumof JL. Rebound hyperkalemia after cessation of intravenous tocolytic therapy with terbutaline in the treatment of preterm labor: anesthetic implications.[see comment]. Journal of Clinical Anesthesia 2003 Aug;15(5):357-8.

Kurzel RB, Ott J. Cerclage, tocolysis and antibiotics for premature rupture of the membranes in previable twins: A case report. Journal of Reproductive Medicine for the Obstetrician and Gynecologist 1992;37(3):289-90.

Lam F, Elliott J, Jones JS, et al. Clinical issues surrounding the use of terbutaline sulfate for preterm labor. [Review] [99 refs]. Obstetrical & Gynecological Survey 1998 Nov;53(11 Suppl):S85-S95.

Lam F, Gill P. beta-Agonist tocolytic therapy. [Review] [114 refs]. Obstetrics & Gynecology Clinics of North America 2005 Sep;32(3):457-84.

Lamont RF. The management of preterm labor. Clin Obstet Gynaecol 1986 Jun;13(2):231-46.

Leonardi MR, Hankins GDV. What's new in tocolytics. Clinics in Perinatology 1992;19(2):367-84.

Levav AL, Chan L, Wapner RJ. Long-term magnesium sulfate tocolysis and maternal osteoporosis in a triplet pregnancy: a case report. American Journal of Perinatology 1998 Jan;15(1):43-6.

Lewis R, Mercer BM, Salama M, et al. Oral terbutaline after parenteral tocolysis: a randomized, double-blind, placebo-controlled trial. American Journal of Obstetrics & Gynecology 1996 Oct;175(4 Pt 1):834-7.

Lipshitz J. Use of a beta 2-sympathomimetic drug as a temporizing measure in the treatment of acute fetal distress. Am J Obstet Gynecol 1977 Sep 1;129(1):31-6.

Ljungstrom B, Mamsen A, Secher NJ. Indications for tocolytic therapy: incidence of true preterm labor with uterine contractions as the sole deciding factor. Am J Perinatol 1989 Apr;6(2):218-21.

Lyrenas S, Grahnen A, Lindberg B, et al. Pharmacokinetics of terbutaline during pregnancy. European Journal of Clinical Pharmacology 1986;29(5):619-23.

Macones GA, Sehdev HM, Berlin M, et al. Evidence for magnesium sulfate as a tocolytic agent. Obstetrical and Gynecological Survey 1997;52(10):652-8.

Magann EF, Cleveland RS, Dockery JR, et al. Acute tocolysis for fetal distress: terbutaline versus magnesium sulphate. Australian & New Zealand Journal of Obstetrics & Gynaecology 1993 Nov;33(4):362-4.

Magann EF, Norman PF, Bass JD, et al. Acute tocolysis for suspected intrapartum fetal distress: maternal effects of terbutaline versus magnesium sulfate. International Journal of Obstetric Anesthesia 1995 Jul;4(3):140-4.

Mancuso KM, Yancey MK, Murphy JA, et al. Epidural analgesia for cephalic version: A randomized trial. Obstetrics and Gynecology 2000;95(5):648-51.

Marsal K, Fisk NM, Calder AA, et al. The oxytocin antagonist atosiban versus the beta-agonist terbutaline in the treatment of preterm labor. A randomized, double-blind, controlled study. Acta Obstetricia et Gynecologica Scandinavica 2001;80(5):413-22.

McCombs J. Update on tocolytic therapy. [Review] [68 refs]. Annals of Pharmacotherapy 1995 May;29(5):515-22.

Meirowitz NB, Ananth CV, Smulian JC, et al. Value of maintenance therapy with oral tocolytics: a systematic review. [Review] [11 refs]. Journal of Maternal -Fetal Medicine 1999 Jul;8(4):177-83.

Menticoglou SM, Morrison I, Harman CR, et al. Maximum possible impact of tocolytics in preventing preterm birth: a retrospective assessment. Am J Perinatol 1992 Sep;9(5-6):394-7.

Mercer BM, Merlino AA. Magnesium sulfate for preterm labor and preterm birth. Obstetrics and Gynecology 2009;114(3):650-68.

Moravec MA, Hurlbert BJ. Hypokalemia associated with terbutaline administration in obstetrical patients. Anesthesia & Analgesia 1980 Dec;59(12):917-20.

Motazedian S, Ghaffarpasand F, Mojtahedi K, et al. Terbutaline versus salbutamol for suppression of preterm labor: a randomized clinical trial. Ann Saudi Med 2010 Sep;30(5):370-5. [PMID: 20697169].

Neiger R, Star J. Hyperglycemia in non-diabetic gravidas receiving steroids for induction of fetal lung maturation. Journal of Maternal -Fetal Investigation 1997;7(2):89-91.

Niebyl JR. Drugs for inhibition of premature labor. Clin Perinatol 1979 Mar;6(1):53-63.

O'Grady JP, Veille J-C, Holland RL, et al. External cephalic version: A clinical experience. Journal of Perinatal Medicine 1986;14(3):189-96.

O'Leary JA. Prophylactic tocolysis of twins. Am J Obstet Gynecol 1986 Apr;154(4):904-5.

Olofsson P, Rydhstrom H, Sjoberg N-O. How Swedish obstetricians manage premature rupture of the membranes in preterm gestations. American Journal of Obstetrics and Gynecology 1988;159(5):1028-34.

Ostermann M, Porreco RP. Urgent cerclage at advanced cervical dilatation. Journal of Maternal -Fetal Investigation 1993;2(6):279-82.

Pacheco LD, Rosen MP, Gei AF, et al. Management of uterine hyperstimulation with concomitant use of oxytocin and terbutaline. American Journal of Perinatology 2006 Aug;23(6):377-80.

Palmer BS. The pharmacist's role in developing a critical pathway for preterm labor management. Journal of Pharmacy Practice 1997;10(6):379-82.

Papke KR. Management of preterm labor and prevention of premature delivery. Nurs Clin North Am 1993 Jun;28(2):279-88.

Parilla BV, Dooley SL, Minogue JP, et al. The efficacy of oral terbutaline after intravenous tocolysis. American Journal of Obstetrics & Gynecology 1993 Oct;169(4):965-9.

Pearson GD, Veille JC, Rahimtoola S, et al. Peripartum cardiomyopathy: National Heart, Lung, and Blood Institute and Office of Rare Diseases (National Institutes of Health) workshop recommendations and review. JAMA 2000 Mar 1;283(9):1183-8.

Penning S, Garite TJ. Management of fetal distress. Obstetrics and Gynecology Clinics of North America 1999;26(2):259-74.

Petersen R, Carter LS, Chescheir NC, et al. Effects of terbutaline sulfate on fetal cardiac function. Am J Obstet Gynecol 1989 Sep;161(3):509-12.

Pisani RJ, Rosenow EC, III. Pulmonary edema associated with tocolytic therapy. Ann Intern Med 1989 May 1;110(9):714-8.

Price AH, Clissold SP. Salbutamol in the 1980s. A reappraisal of its clinical efficacy. Drugs 1989;38(1):77-122.

Quinn PG, Sherman BW, Tavill AS, et al. Terbutaline hepatitis in pregnancy: report of two cases and literature review. Am J Gastroenterol 1994 May;89(5):781-4.

Quirk J, Bowes J. Intrapartum monitoring and management of the low birth weight fetus. Clinics in Perinatology 1982;9(2):363-80.

Rasmussen OB, Larsen B. [Pulmonary edema during terbutaline infusion against threatened premature labor]. [Danish]. Ugeskrift for Laeger 1993 Apr 26;155(17):1303-4.

Rath W. Maintenance tocolysis between clinical practice and evidence-based medicine. [German]. Geburtshilfe und Frauenheilkunde 2007;67(1):28-32.

Rath W, Bartz C. Tocolysis in preterm labor - Current status. [German]. Geburtshilfe und Frauenheilkunde 2005;65(6):570-9.

Reichmann JP. Home uterine activity monitoring: the role of medical evidence. Obstet Gynecol 2008 Aug;112(2 Pt 1):325-7.

Reichmann JP. Home uterine activity monitoring: an evidence review of its utility in multiple gestations. J Reprod Med 2009 Sep;54(9):559-62.

Reid DL, Davidson SR, Phernetton TM, et al. Influence of terbutaline on ovine uterine response to prostaglandin E₂ challenge. American Journal of Obstetrics and Gynecology 1992;166(1 I):231-5.

Rezapour M, Roomans GM, Backstrom T, et al. X-ray microanalysis of myometrium in parturient women at term. Journal of Submicroscopic Cytology & Pathology 1996 Jan;28(1):75-80.

Ricci JM, Hariharan S, Helfgott A, et al. Oral tocolysis with magnesium chloride: a randomized controlled prospective clinical trial. Am J Obstet Gynecol 1991 Sep;165(3):603-10.

Richards SR, Chang FE, Stempel LE. Hyperlactacidemia associated with acute ritodrine infusion. American Journal of Obstetrics and Gynecology 1983;146(1):1-5.

Ridgway III LE, Muise K, Wright JW, et al. A prospective randomized comparison of oral terbutaline and magnesium oxide for the maintenance of tocolysis. American Journal of Obstetrics and Gynecology 1990;163(3):879-82.

Rosene KA, Featherstone HJ, Benedetti TJ. Cerebral ischemia associated with parenteral terbutaline use in pregnant migraine patients. American Journal of Obstetrics & Gynecology 1982 Jun 15;143(4):405-7.

Ross MG, Nicolls E, Stubblefield PG, et al. Intravenous terbutaline and simultaneous beta 1-blockade for advanced premature labor. American Journal of Obstetrics & Gynecology 1983 Dec 15;147(8):897-902.

Rouse DJ, Widness JA, Weiner CP. Effect of intravenous beta-sympathomimetic tocolysis on human fetal serum erythropoietin levels. American Journal of Obstetrics and Gynecology 1993;168(4):1278-82.

Russo LR, Besinger RE, Tomich PG, et al. Effect of chronic tocolytic therapy on maternal ventricular function in pregnant rabbits. American Journal of Obstetrics & Gynecology 1996 Oct;175(4 Pt 1):847-52.

Rust OA, Bofill JA, Arriola RM, et al. The clinical efficacy of oral tocolytic therapy.[see comment]. American Journal of Obstetrics & Gynecology 1996 Oct;175(4 Pt 1):838-42.

Ryden G. The effect of salbutamol and terbutaline in the management of premature labor. Acta Obstetricia et Gynecologica Scandinavica 1977;56(4):293-6.

Ryden G, Andersson RG, Berg G. Is the relaxing effect of beta-adrenergic agonists on the human myometrium only transitory? Acta Obstet Gynecol Scand Suppl 1982;108:47-51.

Sanchez-Ramos L, Huddleston JF. The therapeutic value of maintenance tocolysis: An overview of the evidence. Clinics in Perinatology 2003;30(4):841-54.

Sanchez-Ramos L, Kaunitz AM, Gaudier FL, et al. Efficacy of maintenance therapy after acute tocolysis: a meta-analysis.[see comment]. American Journal of Obstetrics & Gynecology 1999 Aug;181(2):484-90.

Santi MD, Henry GW, Douglas GL. Magnesium sulfate treatment of preterm labor as a cause of abnormal neonatal bone mineralization. Journal of Pediatric Orthopaedics 1994;14(2):249-53.

Savvidou M, Edwards G, Davies N. Use of steroids, tocolytics and antibiotics in the management of preterm labor in Wales. J Obstet Gynaecol 1998 Jul;18(4):315-7.

Sawaya GF, Robertson PA. Hepatotoxicity with the administration of nifedipine for treatment of preterm labor. American Journal of Obstetrics & Gynecology 1992 Aug;167(2):512-3.

Scheerer LJ, Newman RB, Katz M. Tocodynamometry: Past and present. Current Problems in Obstetrics, Gynecology and Fertility 1988;11(3):79-103.

Schwarz R, Retzke U. Cardiovascular effects of terbutalin in pregnant women. Acta Obstetricia et Gynecologica Scandinavica 1983;62(5):419-23.

Sciscione AC, Ivester T, Largoza M, et al. Acute pulmonary edema in pregnancy. Obstetrics & Gynecology 2003 Mar;101(3):511-5.

Shinkai N, Takasuna K, Takayama S. Inhibitory effects of formoterol on lipopolysaccharide-induced premature delivery through modulation of proinflammatory cytokine production in mice. Reproduction 2003;125(2):199-203.

Shumway JB, Al-Malt A, Amon E, et al. Impact of oligohydramnios on maternal and perinatal outcomes of spontaneous premature rupture of the membranes at 18-28 weeks. Journal of Maternal -Fetal Medicine 1999;8(1):20-3.

Sisson MC. Preventing preterm labor. Is terbutaline our best option?. [Review] [35 refs]. AWHONN Lifelines 1997 Apr;1(2):42-6.

Skjaerris J, Aberg A. Prevention of prematurity in twin pregnancy by orally administered terbutaline. Acta Obstetricia et Gynecologica Scandinavica - Supplement 1982;108:39-40, 1982.:39-40.

Slotkin TA, Baker FE, Dobbins SS, et al. Prenatal terbutaline exposure in the rat: Selective effects on development of noradrenergic projections to cerebellum. Brain Research Bulletin 1989;23(4-5):263-5.

Slotkin TA, Kudlacz EM, Lappi SE, et al. Fetal terbutaline exposure causes selective postnatal increases in cerebellar alpha-adrenergic receptor binding. Life Sciences 1990;47(22):2051-7.

Slotkin TA, Seidler FJ. Transient postnatal elevation of norepinephrine content and turnover in brain regions of rats exposed to terbutaline prenatally: evidence for autoregulation of noradrenergic development? Research Communications in Chemical Pathology & Pharmacology 1992 Jun;76(3):269-78.

Smigaj D, Roman-Drago NM, Amini SB, et al. The effect of oral terbutaline on maternal glucose metabolism and energy expenditure in pregnancy. American Journal of Obstetrics & Gynecology 1998 May;178(5):1041-7.

Smith CS, Woodland MB. Clinical comparison of oral nifedipine and subcutaneous terbutaline for initial tocolysis. American Journal of Perinatology 1993 Jul;10(4):280-4.

Smith GN, Brien JF. Use of nitroglycerin for uterine relaxation. Obstetrical and Gynecological Survey 1998;53(9):559-65.

Souney PF, Kaul AF, Osathanondh R. Pharmacotherapy of preterm labor. [Review] [201 refs]. Clinical Pharmacy 1983 Jan;2(1):29-44.

Spinillo A, Capuzzo E, Stronati M, et al. Obstetric risk factors for periventricular leukomalacia among preterm infants. Br J Obstet Gynaecol 1998 Aug;105(8):865-71.

Stanton RJ. Comanagement of the patient on subcutaneous terbutaline pump therapy. Journal of Nurse-Midwifery 1991 May;36(3):204-8.

Sullivan CA, Morrison JC. Emergent management of the patient in preterm labor. Obstetrics and Gynecology Clinics of North America 1995;22(2):197-214.

Svenningsen NW. Follow-up studies on preterm infants after maternal beta-receptor agonist treatment. Acta Obstet Gynecol Scand Suppl 1982;108:67-70.

TambyRaja RL. Current concepts in the management of preterm labor. Singapore Med J 1989 Dec;30(6):578-83.

Tan PC, Daud SA, Omar SZ. Concurrent dinoprostone and oxytocin for labor induction in term premature rupture of membranes: A randomized controlled trial. Obstetrics and Gynecology 2009;113(5):1059-65.

Teoh TG. Outcome of 80 cases of external cephalic version. Medical Journal of Malaysia 1996 Dec;51(4):469-74.

Thornton JG. Maintenance tocolysis. BJOG 2005 Mar;112 Suppl 1:118-21.

Thorp J, Hartmann KE. Evidence-based management of preterm labor: The role of tocolytics and antibiotics. Current Problems in Obstetrics, Gynecology and Fertility 2002;25(6):212-29.

Thoulon JM, Berland M, Beaune J. Beta-sympathomimetics and premature delivery. [French]. Revue Francaise de Gynecologie et d'Obstetrique 1985;80(12):901-15.

Thurlow JA, Kinsella SM. Intrauterine resuscitation: Active management of fetal distress. International Journal of Obstetric Anesthesia 2002;11(2):105-16.

Tibaldi JM, Lorber DL, Nerenberg A. Diabetic ketoacidosis and insulin resistance with subcutaneous terbutaline infusion: a case report. American Journal of Obstetrics & Gynecology 1990 Aug;163(2):509-10.

Travis BE, McCullough JM. Pharmacotherapy of preterm labor. Pharmacotherapy 1993 Jan;13(1):28-36.

Tsatsaris V, Papatsonis D, Goffinet F, et al. Tocolysis with nifedipine or beta-adrenergic agonists: a meta-analysis. Obstet Gynecol 2001 May;97(5 Pt 2):840-7.

US National Heart,Lung,and Blood Institute.National Asthma Education Program. Executive summary: management of asthma during pregnancy. Report of the Working Group on Asthma and Pregnancy. United States Department of Health and Human Services National Institutes of Health; 1993.

Van de BM, Van BF, Lineman R, et al. Perinatal factors and periventricular-intraventricular hemorrhage in preterm infants. Am J Dis Child 1986 Nov;140(11):1125-30.

Varnagy A, Tamas P, Toth T, et al. Comparison of atosiban-magnesium sulfate combination and terbutalin for tocolysis (Preliminary study). [Hungarian]. Magyar Noorvosok Lapja 2003;66(6):331-4.

Veille JC. Management of preterm premature rupture of membranes. [Review] [61 refs]. Clinics in Perinatology 1988 Dec;15(4):851-62.

Viamontes CM. Pharmacologic intervention in the management of preterm labor: an update. J Perinat Neonatal Nurs 1996 Mar;9(4):13-30.

Von Der Pool BA. Preterm labor: Diagnosis and treatment. American Family Physician 1998;57(10):2457-64.

Wager J, Fredholm BB, Lunell NO, et al. Development of tolerance to oral salbutamol in the third trimester of pregnancy: A study of circulatory and metabolic effects. British Journal of Clinical Pharmacology 1981;12(4):489-95.

Weale NK, Kinsella SM. Intrauterine fetal resuscitation. Anaesthesia and Intensive Care Medicine 2007;8(7):282-5.

Weidinger H, Wiest W, Schleich A, et al. The effects of betamimetic drugs used for tocolysis on the fetal myocardium. J Perinat Med 1976;4(4):280-5.

Weintraub Z, Solovechick M, Reichman B, et al. Effect of maternal tocolysis on the incidence of severe periventricular/intraventricular haemorrhage in very low birthweight infants. Arch Dis Child Fetal Neonatal Ed 2001 Jul;85(1):F13-F17

Wheeler DG. Preterm birth prevention. J Nurse Midwifery 1994 Mar;39(2 Suppl):66S-80S.

Multicenter randomized, controlled trial of a preterm birth prevention program. Collaborative Group on Preterm Birth Prevention. Am J Obstet Gynecol 1993 Aug;169(2 Pt 1):352-66.

Preterm labor. International Journal of Gynecology and Obstetrics 1995;50(3):303-13.

Effectiveness and safety of the oxytocin antagonist atosiban versus beta-adrenergic agonists in the treatment of preterm labor. The Worldwide Atosiban versus Beta-agonists Study Group. BJOG 2001 Feb;108(2):133-42.

Abstracts of papers and posters to be presented at the ACOG 53rd Annual Clinical Meeting, May 7-11, 2005, San Francisco, California. Obstetrics & Gynecology 2005 Apr;105(4):1s-124s.

Full-Text: SQ terbutaline pump was not given as maintenance tocoytic therapy in at least one group (n=61):

Adam K, Ou C-N, Cotton DB. Combined effect of terbutaline and betamethasone on glucose homeostasis in preterm labor. Fetal Diagnosis and Therapy 1993;8(3):187-94.

Barden TP, Stander RW. Myometrial and cardiovascular effects of an adrenergic blocking drug in human pregnancy. Am J Obstet Gynecol 1968 May 1;101(1):91-9.

Beall MH, Edgar BW, Paul RH, et al. A comparison of ritodrine, terbutaline, and magnesium sulfate for the suppression of preterm labor. Am J Obstet Gynecol 1985 Dec 15;153(8):854-9.

Berg G, Lindberg C, Ryden G. Terbutaline in the treatment of preterm labor. European Journal of Respiratory Diseases - Supplement 1984;134:219-30, 1984.:219-30.

Bergman B, Hedner T. Antepartum administration of terbutaline and the incidence of hyaline membrane disease in preterm infants. Acta Obstet Gynecol Scand 1978;57(3):217-21.

Bishop EH, Woutersz TB. Arrest of premature labor. JAMA 1961 Nov 25;178:812-4.

Bivins HA, Newman RB, Fyfe DA, et al. Randomized comparative trial of indomethacin and terbutaline for the long term treatment of preterm labor. American Journal of Obstetrics and Gynecology 1993;168:375

Bohm N, Adler CP. Focal necroses, fatty degeneration and subendocardial nuclear polyploidization of the myocardium in newborns after beta-sympathicomimetic suppression of premature labor. Eur J Pediatr 1981 May;136(2):149-57.

Bremme K, Eneroth P. Serum estriol levels during beta-receptor agonist infusion in the third trimester of pregnancy. Gynecologic & Obstetric Investigation 1981;12(2):93-8.

Bremme K, Eneroth P, Hagenfeld L, et al. Changes in maternal serum aldosterone, potassium and prolactin levels during beta-receptor agonist treatment in third trimester pregnancies. Hormone & Metabolic Research 1982 Apr;14(4):198-204.

Caritis SN, Carson D, Greebon D, et al. A comparison of terbutaline and ethanol in the treatment of preterm labor. American Journal of Obstetrics and Gynecology 1982;142(2):183-90.

Chan J, Cabrol D, Ingemarsson I, et al. Pragmatic comparison of beta2-agonist side effects within the Worldwide Atosiban versus Beta Agonists study. Eur J Obstet Gynecol Reprod Biol 2006 Sep;128(1-2):135-41.

Chau AC, Gabert HA, Miller JM, Jr. A prospective comparison of terbutaline and magnesium for tocolysis. Obstetrics & Gynecology 1992 Nov;80(5):847-51.

Coleman B, Grant T, Mueller B. Hospitalization and infant outcomes among women exposed and unexposed to tocolysis. J Perinatol 2005 Apr;25(4):258-64.

Cotton DB, Strassner HT, Lipson LG, et al. The effects of terbutaline on acid base, serum electrolytes, and glucose homeostasis during the management of preterm labor. American Journal of Obstetrics & Gynecology 1981 Nov 15;141(6):617-24.

Dolfin T, Skidmore MB, Fong KW, et al. Perinatal factors that influence the incidence of subependymal and intraventricular hemorrhage in low birthweight infants. Am J Perinatol 1984 Jan;1(2):107-13.

Dudley D, Gagnon D, Varner M. Long-term tocolysis with intravenous magnesium sulfate. Obstetrics and Gynecology 1989;73(3 I):373-8.

Fisher JE, Smith RS, Lagrandeur R, et al. Gestational diabetes mellitus in women receiving beta-adrenergics and corticosteroids for threatened preterm delivery. Obstet Gynecol 1997 Dec;90(6):880-3.

Fredholm BB, Lunell NO, Persson B, et al. Development of tolerance to the metabolic actions of beta 2-adrenoceptor stimulating drugs. Acta Obstet Gynecol Scand Suppl 1982;108:53-9.

Haspedis L. Terbutaline in the treatment of preterm labor: a comparison of intravenous and subcutaneous administration. Journal of the American Osteopathic Association 1988 Apr;88(4):489-93.

Herron MA, Katz M, Creasy RK. Evaluation of a preterm birth prevention program: preliminary report. Obstetrics & Gynecology 1982 Apr;59(4):452-6.

Howard J, Killam AP, Penney LL, et al. A double blind randomized study of terbutaline in premature labor. Military Medicine 1982;147(4):305-7.

Ingemarsson I. Effect of terbutaline on premature labor. A double-blind placebo-controlled study. American Journal of Obstetrics & Gynecology 1976 Jun 15;125(4):520-4.

Ingemarsson I, Bengtsson B. A five-year experience with terbutaline for preterm labor: low rate of severe side effects. Obstetrics & Gynecology 1985 Aug;66(2):176-80.

Jimenez Aramayo JF, Jimenez MF, Lopez RC. [Tocolytic therapy with magnesium sulfate and terbutaline for inhibition of premature labor]. Ginecol Obstet Mex 1990 Sep;58:265-9.

Karlsson K, Krantz M, Hamberger L. Comparison of various betamimetics on preterm labor, survival and development of the child. Journal of Perinatal Medicine 1980;8(1):19-26.

Katz M, Robertson PA, Creasy RK. Cardiovascular complications associated with terbutaline treatment for preterm labor. Am J Obstet Gynecol 1981 Mar 1;139(5):605-8.

Kilburn, KH, Thrasher, J, and Immers, NB. Autism, terbutaline and neurological impairment. CR Online Journal. http://www.collegiumramazzini.org/gest/up/Kilburn.pdfLast Accessed: 29-4-2010

Korenbrot CC, Aalto LH, Laros RK, Jr. The cost effectiveness of stopping preterm labor with beta-adrenergic treatment. N Engl J Med 1984 Mar 15;310(11):691-6.

Kosasa TS, Nakayama RT, Hale RW, et al. Ritodrine and terbutaline compared for the treatment of preterm labor. Acta Obstet Gynecol Scand 1985;64(5):421-6.

Kovacs L, Zalanyi S Jr, Szasz K, et al. [Use of terbutaline sulfate (Bricalyn Astra-Egyt) for betamimetic tocolysis]. Zentralbl Gynakol 1982;104(22):1445-50.

Kuno N, Ishikawa K. Serum creatine phosphokinase elevation in patients treated with intravenous magnesium sulfate. International Journal of Gynecology and Obstetrics 2002;76(3):257-66.

Lampert MB, Hibbard J, Weinert L, et al. Peripartum heart failure associated with prolonged tocolytic therapy. Am J Obstet Gynecol 1993 Feb;168(2):493-5.

Laohapojanart N, Soorapan S, Wacharaprechanont T, et al. Safety and efficacy of oral nifedipine versus terbutaline injection in preterm labor. Journal of the Medical Association of Thailand 2007 Nov;90(11):2461-9.

Laros RK, Jr., Kitterman JA, Heilbron DC, et al. Outcome of very-low-birth-weight infants exposed to beta-sympathomimetics in utero. Am J Obstet Gynecol 1991 Jun;164(6 Pt 1):1657-64.

Mawaldi L, Duminy P, Tamim H. Terbutaline versus nifedipine for prolongation of pregnancy in patients with preterm labor. International Journal of Gynaecology & Obstetrics 2008 Jan;100(1):65-8.

Miller JM, Jr., Keane MW, Horger EO, III. A comparison of magnesium sulfate and terbutaline for the arrest of premature labor. A preliminary report. J Reprod Med 1982 Jun;27(6):348-51.

Mosler KH, Dornhöfer W. The inhibition of premature labor by -adrenergic sympathomimetics and prostaglandin antagonists in managing premature onset of labor. Naunyn Schmiedebergs Arch Pharmacol 1973;277:

Phuapradit W, Pongthai S, Chaturachinda K. Treatment of premature labor with terbutaline. J Med Assoc Thai 1983 Aug;66(8):453-7.

Pitzer M, Schmidt MH, Esser G, et al. Child development after maternal tocolysis with beta-sympathomimetic drugs. Child Psychiatry Hum Dev 2001;31(3):165-82.

Pranikoff J, Helmchen R, Evertson L. Tocolytic therapy and intraventricular hemorrhage in the neonate. Am J Obstet Gynecol 1991;164:387

Robertson PA, Herron M, Katz M, et al. Maternal morbidity associated with isoxsuprine and terbutaline tocolysis. Eur J Obstet Gynecol Reprod Biol 1981 May;11(6):371-8.

Ron-El R, Mor Z, Weinraub Z, et al. Triplet, quadruplet and quintuplet pregnancies. Management and outcome. Acta Obstet Gynecol Scand 1992 Jul;71(5):347-50.

Sciscione A, Gorman R, Schlossman P, et al. A randomized prospective study of intravenous magnesium sulfate, ritrodine, and subcutaneous terbutaline as treatments for preterm labor. American Journal of Obstetrics and Gynecology 1993;168:376

Sciscione AC, Stamilio DM, Manley JS, et al. Tocolysis of preterm contractions does not improve preterm delivery rate or perinatal outcomes. Am J Perinatol 1998 Mar;15(3):177-81.

Sharif DS, Huhta JC, Moise KJ, Jr., et al. Changes in fetal hemodynamics with terbutaline treatment and premature labor. J Clin Ultrasound 1990 Feb;18(2):85-9.

Smythe AR, Sakakini J, Jr. Maternal metabolic alterations secondary to terbutaline therapy for premature labor. Obstetrics & Gynecology 1981 May;57(5):566-70.

Solum T. Clinical experience of terbutaline in preterm labor. Acta Obstetricia et Gynecologica Scandinavica 1982;61(Suppl. 108):37-8.

Sorensen KE, Borlum KG. Fetal cardiac function in response to long-term maternal terbutalin treatment. Acta Obstetricia et Gynecologica Scandinavica 1988;67(2):105-7.

Spatling L, Fallenstein F, Schneider H, et al. Bolus tocolysis: treatment of preterm labor with pulsatile administration of a beta-adrenergic agonist. Am J Obstet Gynecol 1989 Mar;160(3):713-7.

Stubblefield PG, Heyl PS. Treatment of premature labor with subcutaneous terbutaline. Obstetrics & Gynecology 1982 Apr;59(4):457-62.

Surichamorn P. The efficacy of terbutaline and magnesium sulfate in the management of preterm labor. J Med Assoc Thai 2001 Jan;84(1):98-104.

Svenningsen NW, Holmquist P, Ingemarsson I. Antenatal management of terbutaline--effects on infants born preterm. J Singapore Paediatr Soc 1985;27(3-4):208-14.

Takagi K, Satoh K. Is long-term tocolysis effective for threatened premature labor? Journal of International Medical Research 2009;37(1):227-39.

Thorp JA, Jones PG, Clark RH, et al. Perinatal factors associated with severe intracranial hemorrhage. Am J Obstet Gynecol 2001 Oct;185(4):859-62.

Wagner JM, Morton MJ, Johnson KA, et al. Terbutaline and maternal cardiac function. JAMA 1981 Dec 11;246(23):2697-701.

Wallace RL, Caldwell DL, Ansbacher R, et al. Inhibition of premature labor by terbutaline. Obstetrics & Gynecology 1978 Apr;51(4):387-92.

Weerakul W, Chittacharoen A, Suthutvoravut S. Nifedipine versus terbutaline in management of preterm labor. Int J Gynaecol Obstet 2002 Mar;76(3):311-3.

Weiss MA. Retrospective analysis of the effects of ritodrine and terbutaline in the management of preterm labor. Clin Pharm 1982 Sep;1(5):453-6.

Wright JW, Patterson RM, Ridgway LE, III, et al. Effect of tocolytic agents on fetal umbilical velocimetry. American Journal of Obstetrics & Gynecology 1990 Sep;163(3):748-50.

Zalut T, Reed KL, Shenker L. Incidence of premature labor in diabetic patients. American Journal of Perinatology 1985;2(4):276-8.

Full-Text: Review article, letter to the editor commentary, or newspaper article (n=51):

Benedetti TJ. Maternal complications of parenteral beta-sympathomimetic therapy for premature labor. Am J Obstet Gynecol 1983 Jan 1;145(1):1-6.

Bey M, Blanchard AC, Darnell J, et al. Myocardial necrosis in a newborn after long-term maternal subcutaneous terbutaline for suppression of premature labor.[comment]. American Journal of Obstetrics & Gynecology 1992 Jul;167(1):292-3.

Boyle JG. Beta-adrenergic agonists. Clinical Obstetrics and Gynecology 1995;38(4):688-96.

Brinsmead M. Fetal and neonatal effects of drugs administered in labor. Med J Aust 1987 May 4;146(9):481-6.

Catanzarite V. Subcutaneous tocolytic infusion therapy for patients at very high risk for preterm birth.[comment]. Journal of Perinatology 1992 Dec;12(4):387-8.

Challis J R G, Lye F A P S. Control of parturition. In Labor and Delivery: The Proceedings of the 2nd World Congress on Labor and Delivery, May 1997, Rome, Italy Taylor & Francis; 1998. p. 9.

Clayton J, Senior J, Marshall K. Drugs for preterm labor. Practitioner 1993 Jul;237(1528):582-4.

Cole RM, Lamont RF. Current perspectives on drug treatment for preterm labor. J Obstet Gynaecol 1998 Jul;18(4):309-14.

Creasy R K. Side effects of beta-mimetic administration: Tocolytic agents. In Proceedings of a Symposium on Tocolytic Therapy.New Orleans: The Society of Perinatal Obstetricians 1980. p. 87.

Crowther CA, Moore V. Magnesium maintenance therapy for preventing preterm birth after threatened preterm labor. Cochrane Database of Systematic Reviews 1998 Mar;(1):

Cseh I. Place and role of Bricanyl in obstetrical practice. Therapia Hungarica 1993;41(1):23-7.

Devoe LD. Terbutaline pump treatment of premature labor.[comment]. Southern Medical Journal 1993 Sep;86(9):1076, 1993.

Drife J, Kuenzel W. Editorial. European Journal of Obstetrics Gynecology and Reproductive Biology 2006;128(1-2):1-4.

Gill P, Smith M, McGregor C. Terbutaline by pump to prevent recurrent preterm labor. MCN, American Journal of Maternal Child Nursing 1989 May;14(3):163-7.

Gookin KS, Nagey DA, Bailey-Jones C, et al. Randomized comparison of home uterine activity monitoring and routine care in patients discharged after treatment for preterm labor [2]. Obstetrics and Gynecology 1994;83(1):159-60.

Hannah ME. Search for best tocolytic for preterm labor. Lancet 2000 Aug 26;356(9231):699-700.

Hawkins D. Management of premature labor. Journal of Obstetrics & Gynaecology 1989;10(Suppl. 1):S10-S12

HAYES, Inc. Continuous subcutaneous terbutaline infusion for treatment of preterm labor (Structured abstract). Lansdale, Pa: Hayes, Inc 2006;

Hearne AE, Nagey DA. Therapeutic agents in preterm labor: tocolytic agents. Clin Obstet Gynecol 2000 Dec;43(4):787-801.

Huddleston JF. Preterm labor. Clin Obstet Gynecol 1982 Mar;25(1):123-36.

Janke J. Use of terbutaline sulfate in a subcutaneous infusion. Pump to treat preterm labor. Nurse Practitioner 1993;18(9):33-4.

Jelsema RD. Subcutaneous terbutaline pump.[comment]. Journal of Reproductive Medicine 1998 Apr;43(4):401-2.

Katz VL, Seeds JW. Fetal and neonatal cardiovascular complications from beta-sympathomimetic therapy for tocolysis. Am J Obstet Gynecol 1989 Jul;161(1):1-4.

Lam F, Gill P. Subcutaneous terbutaline therapy in triplet gestation. In: Keith L, Blickstein I, editors. Triplet pregnancies and their consequences, New York: Parthenon; 2002. p. 181-202.

Lam F. Miniature pump infusion of terbutaline: An option in preterm labor. Contemporary OB/GYN 1988;33:53-70.

Lam F. The dosing of subcutaneous terbutaline pump tocolytic therapy is critical.[comment]. American Journal of Obstetrics & Gynecology 1992 Oct;167(4 Pt 1):1156-7.

Landesman R, Fuchs F. Control of uterine activity and prevention of premature birth. Basic Life Sci 1974;4(PT. B):219-41.

Lashgari S, Kueck AS, Oyelese Y. Atrial fibrillation in pregnancy associated with oral terbutaline therapy.[comment]. Obstetrics & Gynecology 2003 Apr;101(4):814, 2003.

Leveno KJ, Cunningham FG. Beta-adrenergic agonists for preterm labor. N Engl J Med 1992 Jul 30;327(5):349-51.

Lipshitz J. Preterm parturition. Beta-adrenergic agonists. Semin Perinatol 1981 Jul;5(3):252-65.

Martin AJ. Severe unwanted effects associated with betasympathomimetics when used in the treatment of premature labor: causes, incidence and preventative measures. Br J Clin Pract 1981 Sep;35(9):325-9.

McPherson ML. Management of preterm labor with continuous subcutaneous home terbutaline therapy. Journal of Home Health Care Practice 1992 Nov;5(1):22-5.

Miller DD. Tocolytic therapy for the prevention of preterm labor. Journal of Intravenous Nursing 1995;18(5):233-8.

Moise KJ, Jr. Terbutaline pump treatment of premature labor.[comment]. Southern Medical Journal 1993 Sep;86(9):1076-7.

Monga M, Creasy RK. Pharmacologic management of preterm labor. Semin Perinatol 1995 Feb;19(1):84-96.

Moore B, Briggs G, Freeman R. Terbutaline for tocolysis: Do advantages outweigh risks? Contemp OB/GYN 1988;32:53

Nanda K, Cook LA, Gallo MF, et al. Terbutaline pump maintenance therapy after threatened preterm labor for preventing preterm birth. Cochrane Database of Systematic Reviews 2002 Dec;(4):

Niebyl JR, Johnson JW. Inhibition of preterm labor. Clin Obstet Gynecol 1980 Mar;23(1):115-26.

Nightingle SL. From the Food and Drug Administration. Journal of the American Medical Association 1998;279(1):9

Norwitz ER, Robinson JN, Challis JRG. The control of labor. New England Journal of Medicine 1999;341(9):660-6.

Prevost RR. Treatment of pregnancy-related illnesses. American Pharmacy 1995;35(10):25-32.

Roan S. T-pump therapy undergoing a close second look. Los Angeles Times -- Southern California Edition (Front Page) 1998 Sep 15;S3

Rogge P, Young S, Goodlin R. Post-partum pulmonary oedema associated with preventive therapy for premature labor. Lancet 1979 May 12;313(8124):1026-7.

Rose VL. Warning on terbutaline sulfate in preterm labor. American Family Physician 1998;57(8):2014-5.

Shellhaas CS, Iams JD. Ambulatory management of preterm labor. Clinical Obstetrics and Gynecology 1998;41(3):491-502.

Simhan HN, Caritis SN. Prevention of preterm delivery. N Engl J Med 2007 Aug 2;357(5):477-87.

Unbehaun V. Effects of sympathomimetic tocolytic agents on the fetus. J Perinat Med 1974;2(1):17-29.

Voelker R. Infusion helps prolong pregnancy. Journal of the American Medical Association 1998;279(12):902

Witter FR, Zimmerman AW, Reichmann JP, et al. In utero beta 2 adrenergic agonist exposure and adverse neurophysiologic and behavioral outcomes. Am J Obstet Gynecol 2009 Dec;201(6):553-9.

New help for preterm birth prevention: pump therapy lets patients remain at home. Naacog Newsletter 1989 Oct;16(4):4, 10, 1989.

Worth noting: terbutaline pump warning. OB-GYN Malpract Prev 1997 Mar;4(3):23

Full-Text: A study of maternal/neonatal outcomes or harms without at least one comparison group (n=25):

Allbert JR, Wise CA, Lou CH, et al. Subcutaneous tocolytic infusion therapy for patients at very high risk for preterm birth. J Perinatol 1992 Mar;12(1):28-31.

Ambrose S, Jacques D, Stanziano G. Clinical and economic outcomes associated with home vs hospital use of continuous subcutaneous tocolytic therapy. In Proceedings of the annual meeting of the American College of Obstetricians and Gynecologists 2001.

Ambrose S, Rhea DJ, Istwan NB, et al. Clinical and economic outcomes of preterm labor management: inpatient vs outpatient. Journal of Perinatology 2004 Aug;24(8):515-9.

Elliott JP, Bergauer NK, Jacques DL, et al. Pregnancy prolongation in triplet pregnancies. Oral vs. continuous subcutaneous terbutaline.

Journal of Reproductive Medicine 2001 Nov;46(11):975-82.

Elliott JP, Flynn MJ, Kaemmerer EL, et al. Terbutaline pump tocolysis in high-order multiple gestation.[see comment]. Journal of Reproductive Medicine 1997 Nov;42(11):687-94.

Elliott JP, Istwan NB, Rhea D, et al. The occurrence of adverse events in women receiving continuous subcutaneous terbutaline therapy. American Journal of Obstetrics & Gynecology 2004 Oct;191(4):1277-82.

Elliott JP, Radin TG. Quadruplet pregnancy: contemporary management and outcome.[see comment]. Obstetrics & Gynecology 1992 Sep;80(3 Pt 1):421-4.

Fischer JR, Kaatz BL. Continuous subcutaneous infusion of terbutaline for suppression of preterm labor. Clinical Pharmacy 1991 Apr;10(4):292-6.

Gaziano E, Wagner W, Rhea D. Inpatient vs. outpatient surveillance for singleton pregnancies following inpatient treatment with magnesium sulfate for preterm labor. In Society for Gynecologic Investigation Annual Meeting 2004.

Gianopoulos J, Carlson N, Schumachen B. SQ terbutaline pump for premature labor. Am J Obstet Gynecol 1991;164:426

Hamersley SL, Coleman SK, Bergauer NK, et al. Delayed-interval delivery in twin pregnancies. J Reprod Med 2002 Feb;47(2):125-30.

Hammersley S, Pinckert T, Gorman K. The use of continuous subcutaneous terbutaline in a private maternal–fetal medicine practice. Obstet Gynecol 1999;93:67-8.

Jones J, Morrison J, Istwan N, et al. The interval to spontaneous delivery following discontinuation of maintenance tocolysis. Journal of Maternal -Fetal and Neonatal Medicine 2006;19(6):331-5.

Jones P, Greenspan J, Bochner C. Continuous subcutaneous terbutaline infusion for the prevention of recurrent preterm labor. Am J Obstet Gynecol 1991;164:426

Lam F, Bergauer N, Stanziano G, et al. Pregnancy prolongation and route of tocolytic administration in patients with singleton gestation. Am J Obstet Gynecol 1998;178(Suppl. 1):180

Lam F, Bergauer NK, Coleman SK, et al. A comparison of gestational days gained with oral terbutaline versus continuous subcutaneous terbutaline in women with twin gestations. Journal of Perinatology 2000 Oct;20(7):408-13.

Moise KJ, Jr., Sala DJ, Zurawin RK, et al. Continuous subcutaneous terbutaline pump therapy for premature labor: safety and efficacy. Southern Medical Journal 1992 Mar;85(3):255-60.

Perry KG, Jr., Morrison JC, Rust OA, et al. Incidence of adverse cardiopulmonary effects with low-dose continuous terbutaline infusion. American Journal of Obstetrics & Gynecology 1995 Oct;173(4):1273-7.

Pomini F, De CS, Ferrazzani S, et al. [Continuous subcutaneous administration of terbutaline in the therapy of threatening premature labor]. [Italian]. Minerva Ginecologica 1993 Jan;45(1-2):57-63.

Rebarber A, Roman A, Istwan N. Factors associated with spontaneous preterm delivery in singleton gestations. Obstet Gynecol 2004;103(4):

Rebarber A, Cleary-Goldman J, Istwan N, et al. The association of elective cessation of tocolysis and preterm birth in singleton gestations. American Journal of Perinatology 2009 May;26(5):351-5.

Roman A, Rebarber A, Istwan N. Clinical and demographic factors associated with spontaneous preterm delivery in twin pregnancies diagnosed with preterm labor at 34 weeks. Am J Obstet Gynecol 2003;189:S123

Viscarello R, Griffith S, Jacques D. The effect of proactive dose acceleration in triplets receiving continuous subcutaneous tocolysis. Obstet Gynecol 2002;99:12

Weinbaum P, Olson M. The effect of subcutaneous infusion on uterine activity in patients at risk for preterm delivery. Am J Obstet Gynecol 1993;166:362

Wolfsen R, Winn S. Prolongation of twin pregnancy with magnesium sulphate / subcutaneous terbutaline pump therapy in the face of advanced cervical dilation and effacement. Am J Obstet Gynecol 1992;166:366

Full-Text: Case reports (n=18):

Benedetti TJ, Hargrove JC, Rosene KA. Maternal pulmonary edema during premature labor inhibition. Obstet Gynecol 1982 Jun;59(6 Suppl):33S-7S.

Brandstetter RD, Gotz V. Inadvertent overdose of parenteral terbutaline. Lancet 1980;1(8166):485

Clesham GJ. Beta adrenergic agonists and pulmonary oedema in preterm labor. BMJ 1994 Jan 22;308(6923):260-2.

Gimovsky ML. Short-term tocolysis adjunctive to intrapartum term breech management. American Journal of Obstetrics and Gynecology 1985;153(2):233

Gross TL, Sokol RJ. Severe hypokalemia and acidosis: a potential complication of beta-adrenergic treatment. Am J Obstet Gynecol 1980 Dec 15;138(8):1225-6.

Hawker F. Five cases of pulmonary oedema associated with beta 2-sympathomimetic treatment of premature labor. Anaesth Intensive Care 1984 May;12(2):159-62.

Hill WC. Overdosage of subcutaneous terbutaline in the treatment of preterm labor. Nebraska Medical Journal 1986 Jul;71(7):242-3.

Ingemarsson I. Tocolytic therapy and clinical experience. Combination therapy. BJOG : An International Journal of Obstetrics & Gynaecology 2005 Mar;112 Suppl 1:89-93, 2005 Mar.:89-93.

Jacobs MM, Knight AB, Arias F. Maternal pulmonary edema resulting from betamimetic and glucocorticoid therapy. Obstet Gynecol 1980 Jul;56(1):56-9.

Kalb,K.A. Women's experiences using terbutaline pump therapy for the management of preterm labor. University of Minnesota. 1993.

Levy DL. Morbidity caused by terbutaline infusion pump therapy.[comment]. American Journal of Obstetrics & Gynecology 1994 Jun;170(6):1835, 1994.

Mabie WC, Pernoll ML, Witty JB, et al. Pulmonary edema induced by betamimetic drugs. South Med J 1983 Nov;76(11):1354-60.

Mahon WA, Reid DW, Day RA. The in vivo effects of beta adrenergic stimulation and blockade on the human uterus at term. J Pharmacol Exp Ther 1967 Apr;156(1):178-85.

Milos M, Aberle DR, Parkinson BT, et al. Maternal pulmonary edema complicating beta-adrenergic therapy of preterm labor. AJR Am J Roentgenol 1988 Nov;151(5):917-8.

Sala DJ, Moise KJ, Jr. The treatment of preterm labor using a portable subcutaneous terbutaline pump. JOGNN - Journal of Obstetric, Gynecologic, & Neonatal Nursing 1990 Mar;19(2):108-15.

Semchyshyn S, Zuspan FP, O'Shaughnessy R. Pulmonary edema associated with the use of hydrocortisone and a tocolytic agent for the management of premature labor. J Reprod Med 1983 Jan;28(1):47-52.

Thorkelsson T, Loughead JL. Long-term subcutaneous terbutaline tocolysis: report of possible neonatal toxicity. Journal of Perinatology 1991 Sep;11(3):235-8.

Tye KH, Desser KB, Benchimol A. Angina pectoris associated with use of terbutaline for premature labor. JAMA 1980 Aug 15;244(7):692-3.

Full-Text: Excluded for another reason (no patient data, clearly not relevant to SQ terbutaline pump, betamimetic treatment not specified) (n=13):

Caritis SN. Ritodrine infusion and cardiomyopathy (I). American Journal of Obstetrics and Gynecology 1990;163(1 I):254-5.

Elliott J, Istwan N, Jacques. Expectant management of the quadruplet pregnancy: inpatient or outpatient. Am J Obstet Gynecol 2001;185(6 Suppl. 1):110

Eskes TK, de HJ. The influence of beta-mimetic catecholamines upon the fetal circulation. Z Geburtshilfe Perinatol 1972 Apr;176(2):97-107.

Goldenberg RL, Rouse DJ. Prevention of premature birth. N Engl J Med 1998 Jul 30;339(5):313-20.

Griffin G. Are oral betamimetics effective maintenance therapies after threatened preterm labor? American Family Physician 2007;75(5):648-50.

Groome LJ, Goldenberg RL, Cliver SP, et al. Neonatal periventricular-intraventricular hemorrhage after maternal beta-sympathomimetic tocolysis. The March of Dimes Multicenter Study Group. Am J Obstet Gynecol 1992 Oct;167(4 Pt 1):873-9.

Hatjis CG, Swain M. Systemic tocolysis for premature labor is associated with an increased incidence of pulmonary edema in the presence of maternal infection. Am J Obstet Gynecol 1988 Sep;159(3):723-8.

Kuhlmann RS, Cruikshank DP. Maternal trauma during pregnancy. Clinical Obstetrics and Gynecology 1994;37(2):274-93.

Norton ME. Teratogen update: Fetal effects of indomethacin administration during pregnancy. Teratology 1997;56(4):282-92.

Obradovich JH, Woods DD. Users as designers: how people cope with poor HCI design in computer-based medical devices. Human Factors 1996 Dec;38(4):574-92.

Palta M, Sadek M, Lim TS, et al. Association of tocolytic therapy with antenatal steroid administration and infant outcomes. Newborn Lung Project. Am J Perinatol 1998 Feb;15(2):87-92.

Richards NA, Yentis SM. Anaesthesia, analgesia and peripartum management in women with pre-existing cardiac and respiratory disease. Fetal and Maternal Medicine Review 2006;17(4):327-47.

Multiple gestations. Clin Perinatol 2005 Jun;32(2):xi

Full-Text: Abstract or full-text not in English (either no or cannot tell)/ insufficient information provided in abstract (full-text not in English) (n=12):

Chimura T. [Clinical studies on imipenem/cilastatin sodium in the early therapy of preterm premature rupture of the membrane or threatened abortion and premature delivery]. [Japanese]. Japanese Journal of Antibiotics 1992 Aug;45(8):1023-8.

Criee C-P, Laier-Groeneveld G. Beta₂ sympathicomimetic agents. [German]. Internist 1995;36(9):918-23.

Grospietsch G. [Medical tocolysis in threatening premature labor: what is safe in therapy?]. Gynakologe 1991 Aug;24(4):188-97.

Kishikawa T, Kawarabayashi T, Sugimori H. [Combined therapy with magnesium sulfate and terbutaline for preterm labor tocolysis]. [Japanese]. Nippon Sanka Fujinka Gakkai Zasshi - Acta Obstetrica et Gynaecologica Japonica 1986 Sep;38(9):1670-3.

Lam F, Gill P J, Smith M. Comparison of portable subcutaneous terbutaline pump and oral terbutaline treatment for long-term tocolysis: A randomized clinical trial. Abstract 400. In Eighth annual meeting—Society of Perinatal Obstetricians 1988.

Lam F, Klutz R. The pharmacokinetic impact of the pharmacodynamic effect of subcutaneous terbutaline in the treatment of preterm labor. In Continental Gynecologic Society Annual Meeting 1989. p.1-22.

Lowenberg E, Escobedo F, Garcia P, et al. [The treatment of threatened preterm labor. A comparative study between 2 beta-mimetics]. [Spanish]. Ginecologia y Obstetricia de Mexico 1988 May;56:111-5, 1988 May.:111-5.

Morales WJ, Marko BH. Adjusted oral terbutaline vs subcutaneous terbutaline pump therapy in the management of preterm labor. Proceedings of 10th Annual Meeting of Society of Perinatal Obstetricians; 1990 Jan 23 27; Houston, Texas, USA 1990;149

Vargas G, Lowenberg E. Terburalina. Un nuevo utero-inhibidor. Ginec Obstet Mex 1974;36:75

Woodland MB, Smith C, Byers J, et al. Clinical comparison of oral nifedipine and subcutaneous terbutaline use for initial tocolysis. Proceedings of 10th Annual Meeting of Society of Perinatal Obstetricians; 1990 Jan 23 27; Houston, Texas, USA 1990;523

Institute for Clinical Systems Improvement (ICSI). Tocolytic therapy for preterm labor. 49. Bloomington, MN: ICSI; 2000.

Prevention program boosts preterm labor outcomes. Home Care Case Management 1997 Feb;4(2):21-3, insert.

Full-Text: Unavailable (n=7):

Ingemarsson I. Terbutaline treatment of premature labor - Low incidence of severe side effects. In 10th World Congress of Gynecology and Obstetrics. 1982. p. Abstract 1213.

Jones J, Istwan. Pregnancy prolongation after discontinuation of subcutaneous tocolysis in singleton gestations. Obstet Gynecol 2003;101:4

Lam F, Graves W K, Peacock W G. Side effects of beta-mimetic administration: Tocolytic agents. In ACOG Districts VIII and IX Meeting, Las Vegas, Nevada 1987.

McGettigan M, Bhutani V, Rathbone A. Prenatal b-mimetic exposure of the newborn: maternal oral vs. continuous infusion therapy. Pediatr Res 1991;30:62A

Moise K J, Dorman K, Giebel R. A randomized study of intravenous versus subcutaneous/oral terbutaline in the treatment of preterm labor. In Seventh Annual Meeting of the Society of Perinatal Obstetricians. 1987. p. Abstract #276.

Sharif D, Huhta J, Moise K J. Changes in fetal hemodynamics with preterm labor and terbutaline. In Seventh Annual Meeting of the Society of Perinatal Obstetricians. 1987. p. Abstract 140.

Watson P, Shapiro K, Fure J. Betamimetic-associated pulmonary edema: Time and dose relationships. In Fifth annual meeting, Society of Perinatal Obstetricians. 1985. p. Abstract #68.

Full-Text: Does not include women with arrested preterm labor (n=4):

Besinger RE, Moniak CW, Paskiewicz LS, et al. The effect of tocolytic use in the management of symptomatic placenta previa. American Journal of Obstetrics & Gynecology 1995 Jun;172(6):1770-5.

Estaban-Altirriba J. Treatment of fetal distress, in Tocolytic Agents. In Society of Perinatal Obstetricians. Proceedings of a Symposium on Tocolytic Therapy 1980. p.69-75.

Parsons MT, Owens CA, Spellacy WN. Thermic effects of tocolytic agents: decreased temperature with magnesium sulfate. Obstetrics & Gynecology 1987 Jan;69(1):88-90.

Towers CV, Pircon RA, Heppard M, et al. Is tocolysis safe in the management of third-trimester bleeding? American Journal of Obstetrics and Gynecology 1999;180(6 I):1572-8.

Full-Text: Does not assess one of the specified outcomes (n=3):

Berg G, Andersson RG, Ryden G. Beta-adrenoceptor function of leukocytes in pregnant women treated with terbutaline for preterm labor. Acta Obstetricia et Gynecologica Scandinavica 1984;63(6):555-61.

Berkus M, Langer O, Kigby K. Effect of terbutaline pump on maternal glucose metabolism. Am J Obstet Gynecol 1999;180(Suppl. 1):S41

Towers CV, Kaminskas CM, Garite TJ, et al. Pulmonary injury associated with antepartum pyelonephritis: can patients at risk be identified? Am J Obstet Gynecol 1991 Apr;164(4):974-8.

Full-Text: Companion article with no additional data (n=2):

Ambrose S, Jacques D, Stanziano. Clinical and economic outcomes of continuous subcutaneous tocolysis. Obstet Gynecol 2001;97:47

Wenstrom K, Weiner C, Merrill D, et al. A placebo-controlled trial of the terbutaline (T) pump for prevention of preterm delivery [abstract]. American Journal of Obstetrics and Gynecology 1995;172:416

Full-Text: Study design does not allow for an evaluation of SQ terbutaline pump as the sole maintenance tocolytic therapy (n=1):

Viscarello R, Griffith S, Charney. Outpatient management of higher order multiples with a comprehensive care plan improves outcome. Obstet Gynecol 2002;99:71

Appendix F. Evidence Tables

Table F1. Detailed study-level characteristics

Study Identification	Study Population	Definition of Preterm Labor	Comparison Group (N): Mean Daily Dose ± SD (mg)<br>Intervention Group (N): Mean Daily Dose ± SD (mg)	Overall Risk of Bias With Explanation<br>***(rating applies to all outcomes unless specified otherwise)***
**First Author (year):** Guinn (1998)[9]<br>**Design:** RCT<br>**Setting:** Birmingham Hospital, Alabama (Nov 1994–Apr 1997)<br>**Funding Source:** MiniMed Technologies (supported in part)	**n =** 52<br>**Mean Maternal Age ± SD (years):** 21.6 ± 5.7<br>**Mean Gestational Age ± SD (weeks):** 30.6 ± 2.8 (T)<br>**Gestation:** singletons<br>**Primary Tocolytic Treatment:** Magnesium sulfate (IV) (with or without indomethacin)<br>**Previous Maintenance Tocolytics:** NR<br>**Inclusion Criteria:** Singleton gestation; intact membranes; between 22 and $33^{6/7}$ weeks gestation; received parenteral magnesium sulfate therapy (with or without indomethacin); arrested preterm labor (<4 contractions/h for ≥ 24 hours)<br>**Exclusion Criteria:** Contraindication to tocolysis; persistent maternal tachycardia (>120 beats/min); history of cardiac arrhythmia; history of pulmonary edema; uncontrolled diabetes; suspected chorioamnionitis	Uterine contractions<br>> 4 per hour and greater than or equal to one of the following: ≥ 1 cm cervical dilation, ≥ 80% cervical effacement, and documented cervical change.	**C:** Placebo (saline pump) (28): NA<br>**I:** SQ terbutaline (24): NR	Low<br>The comparability of groups cannot be assessed for certain because information on all relevant factors has not been presented (e.g., prognostic factors, such as cervical length and fetal fibronectin). However, randomization was carried out properly and patients/health care providers were blinded to treatment allocation, which will limit selection and detection biases.

Table F1. Detailed study-level characteristics (continued)

Study Identification	Study Population	Definition of Preterm Labor	Comparison Group (N): Mean Daily Dose ± SD (mg) Intervention Group (N): Mean Daily Dose ± SD (mg)	Overall Risk of Bias With Explanation *(rating applies to all outcomes unless specified otherwise)*
**First Author (year):** Wenstrom (1997)[10]<br>**Design:** RCT<br>**Setting:** University of Iowa Hospital<br>(Jan 1990–Apr 1994)<br>**Funding Source:** NR<br>**Companion Article:** [43]	**n =** 42<br>**Mean Maternal Age ± SD (years):** 26.2 ± 5.3<br>**Mean Gestational Age ± SD (weeks):** 30.4 ± 2.3 (T)<br>**Gestation:** singletons or twins<br>**Primary Tocolytic Treatment:** Magnesium sulfate (IV)<br>(if magnesium was insufficient, indomethacin (PO) was administered)<br>**Previous Maintenance Tocolytics:** NR<br>**Inclusion Criteria:** Diagnosis of preterm labor<br>**Exclusion Criteria:** Contraindication to beta-mimetic therapy (i.e. heart disease, insulin-dependent diabetes mellitus, intolerance to terbutaline) or to continued tocolysis in general; cervical dilation > 4 cm	Regular, persistent uterine contractions that produce cervical change in gravidas ≥ 20 weeks and < 35 weeks.	**C_1:** Placebo (saline pump) (12): NA<br>**C_2:** Oral terbutaline (15): NR<br>**I:** SQ terbutaline (15): NR	High (oral terbutaline arm)<br>High (placebo arm)<br>Placebo arm:<br>The sample likely represents a very select group, since >90% of eligible subjects declined to participate. The study is likely to be underpowered. There is evidence that randomization was carried out properly, but blinding was not that effective. Missing information makes it difficult to judge comparability of groups in baseline characteristics and prognostic factors, primary tocolytic therapy, and level of care.<br>Oral terbutaline arm:<br>Same as above, except for complete absence of blinding.

Table F1. Detailed study-level characteristics (continued)

Study Identification	Study Population	Definition of Preterm Labor	Comparison Group (N): Mean Daily Dose ± SD (mg) Intervention Group (N): Mean Daily Dose ± SD (mg)	Overall Risk of Bias With Explanation *(rating applies to all outcomes unless specified otherwise)*
**First Author (year):** Lindenbaum (1992)[11]‡<br>**Design:** Nonrandomized trial<br>**Setting:** Hospital of the University of Pennsylvania (NR)<br>**Funding Source:** NR	**n =** 91<br>**Mean Maternal Age ± SD (years):** 32.4 ± 2.7<br>**Mean Gestational Age ± SD (weeks):** 29.1 ± 1.7 (T)<br>**Gestation:** singletons<br>**Primary Tocolytic Treatment:** Magnesium sulfate (IV) or Ritodrine (IV)<br>(other agents may have been administered as well)<br>**Previous Maintenance Tocolytics:** NR<br>**Inclusion Criteria:** Women 26–36 weeks' gestation; diagnosis of preterm labor; admitted to labor floor of hospital; normal 1-hour oral glucose tolerance test between 24-28 weeks' gestation<br>**Exclusion Criteria:** History of pregestational or gestational diabetes; macrosomia; current steroid therapy; multiple gestation	Documented cervical change or uterine contractions ≥ 6 per hour that was unresponsive to bed rest and intravenous hydration.	**C:** Oral Terbutaline (54): 30 ± NR<br>**I:** SQ terbutaline (37): NR	High (birth weight and gestational age at delivery)<br>Medium (maternal hyperglycemia)<br>Primary flaw in this study is the difference in groups with respect to severity/prognosis (i.e., groups were divided based on length of primary tocolytic treatment). Also, comparability of groups cannot be assessed due to missing information.<br>The potential difference in severity/prognosis among treatment and comparison groups should not impact the outcome of maternal hyperglycemia. However, issues pertaining to missing information still remain.

Table F1. Detailed study-level characteristics (continued)

Study Identification	Study Population	Definition of Preterm Labor	Comparison Group (N): Mean Daily Dose ± SD (mg) Intervention Group (N): Mean Daily Dose ± SD (mg)	Overall Risk of Bias With Explanation *(rating applies to all outcomes unless specified otherwise)*
**First Author (year):** Morrison (2003)[12]<br>**Design:** Prospective Cohort<br>**Setting:** NR (Jan 2001–Dec 2001)<br>**Funding Source:** NR	**n =** 60<br>**Mean Maternal Age ± SD (years):** 25.6 ± 5.2<br>**Mean Gestational Age ± SD (weeks):** 29.5 ± 2.3 (P)<br>**Gestation:** singletons<br>**Primary Tocolytic Treatment:** Magnesium sulfate (IV)<br>(If magnesium was insufficient, indomethacin (PR) or nifedipine (PO) was administered.)<br>**Previous Maintenance Tocolytics:** NR<br>**Inclusion Criteria:** Two or more episodes of preterm labor; stabilized in hospital with IV tocolytics<br>**Exclusion Criteria:**<br>Further continuation of pregnancy contraindicated (hypertension, fetal distress, intrauterine growth restriction, severe vaginal bleeding); insulin-dependent diabetes; preterm premature rupture of membranes; allergy to beta-sympathomimetic drugs; fetal anomalies; fetal death	Persistent uterine contractions (>12 per hour), cervical change in dilation, and effacement since first episode of PTL.	**C:** No Treatment (45): NA<br>**I:** SQ terbutaline (15): NR	High<br>Primary flaw with this study is that there is evidence that groups were not comparable (with respect to risk factors for preterm birth, primary tocolytic therapy, level of care).

Table F1. Detailed study-level characteristics (continued)

Study Identification	Study Population	Definition of Preterm Labor	Comparison Group (N): Mean Daily Dose ± SD (mg) Intervention Group (N): Mean Daily Dose ± SD (mg)	Overall Risk of Bias With Explanation *(rating applies to all outcomes unless specified otherwise)*
**First Author (year):** Morrison (1992)[13]<br>**Design:** Prospective Cohort<br>**Setting:** NR<br>**Funding Source:** Vicksburg Hospital Medical Foundation (supported in part)	**n =** 69<br>**Mean Maternal Age ± SD (years):** 28.6 ± 4.7<br>**Mean Gestational Age ± SD (weeks):** NR<br>**Gestation:** Not specified (likely included a mixture of women with single and multiple gestation)<br>**Primary Tocolytic Treatment:** NR<br>**Previous Maintenance Tocolytics:** Oral tocolytics (NR)<br>(only received by terbutaline pump group)<br>**Inclusion Criteria:** Treated with IV tocolysis for documented preterm labor; subcutaneous terbutaline group had failed maintenance oral tocolytic therapy (had RPTL)<br>**Exclusion Criteria:** Preterm rupture of membranes; agent discontinued due to failure of tocolysis or advanced cervical dilatation at < 37 weeks; scheduled cesarean deliveries; early delivery for obstetric/medical indications	Regular, persistent uterine contractions (usually > 12/hr) with associated cervical change from the previous exam or a change in cervical status with regular contractions, or contractions plus an initial cervical examination ≥ 2 cm	**C:** Oral tocolytics - ritodrine or terbutaline (41): NR<br>**I:** SQ terbutaline (28): NR	High<br>Major flaw is that the subcutaneous pump group had RPTL and comparison group did not. Therefore, the intervention group may have had a more serious condition. Also, there is missing information, which makes it difficult to assess other potential limitations.

Table F1. Detailed study-level characteristics (continued)

Study Identification	Study Population	Definition of Preterm Labor	Comparison Group (N): Mean Daily Dose ± SD (mg) Intervention Group (N): Mean Daily Dose ± SD (mg)	Overall Risk of Bias With Explanation *(rating applies to all outcomes unless specified otherwise)*
**First Author (year):** Flick (2010)[14]<br>**Design:** Retrospective cohort<br>**Setting:** Throughout United States (Matria database)<br>**Funding Source:** NR	**n =** 1366<br>**Mean Maternal Age ± SD (years):** 28.7 ± 6.1<br>**Mean Gestational Age ± SD (weeks):** 30.6 ± 2.9 (P)<br>**Gestation:** singletons<br>**Primary Tocolytic Treatment:** NR<br>**Previous Maintenance Tocolytics:** Oral nifedipine<br>mean daily dose ± SD (mg): 58.5 ± 26.5<br>**Inclusion Criteria:** Singleton gestation; < than 35 weeks gestation; referred for hospitalization due to RPTL; prescribed oral nifedipine for maintenance tocolysis; hospitalized for a minimum of 24 hours; received preterm labor treatment; intact membranes; subsequently discharged to resume outpatient services with oral nifedipine or continuous subcutaneous terbutaline infusion<br>**Exclusion Criteria:** Delivered upon hospitalization; ruptured membranes; > 35 weeks gestation when hospitalized; did not resume outpatient services	NR	**C:** Oral Nifedipine (830): NR<br>**I:** SQ terbutaline (536): NR	High<br>Primary flaw is that groups were not similar in baseline characteristics and prognostic factors (i.e., differed in smoking status). Also, missing information makes it difficult to assess similarity of groups with respect to other factors.

Table F1. Detailed study-level characteristics (continued)

Study Identification	Study Population	Definition of Preterm Labor	Comparison Group (N): Mean Daily Dose ± SD (mg) Intervention Group (N): Mean Daily Dose ± SD (mg)	Overall Risk of Bias With Explanation *(rating applies to all outcomes unless specified otherwise)*
**First Author (year):** de la Torre (2008)[15]<br>**Design:** Retrospective Cohort<br>**Setting:** Throughout United States (Matria database)<br>**Funding Source:** NR	**n =** 656<br>**Mean Maternal Age ± SD (years):** 30.3 ± 5.8<br>**Mean Gestational Age ± SD (weeks):** 30.1 ± 2.9 (P)<br>**Gestation:** twins<br>**Primary Tocolytic Treatment:** NR<br>**Previous Maintenance Tocolytics:** Oral Nifedipine<br>mean daily dose ± SD (mg): 62.3 ± 26.9<br>**Inclusion Criteria:** Twin gestation; prescribed oral nifedipine as maintenance tocolysis after an initial episode of preterm labor; hospitalized at <35 weeks gestation for RPTL; at least a 24 hour hospital stay<br>**Exclusion Criteria:** Delivered within 48 hours of hospitalization; did not resume maintenance tocolysis; ruptured membranes; referred for hospital evaluation but not admitted	Uterine activity above 4–6 contractions per hour or maternal reports of persistent pelvic pressure, cramping, backache, or increased vaginal discharge.	**C:** Oral Nifedipine (418): 73.7 ± 23.4<br>**I:** SQ terbutaline (238): NR	Medium<br>There is a lot of missing information, which makes it difficult to assess comparability of groups (in terms of baseline characteristics and prognostic factors, primary tocolytic therapy, and compliance). But difficult to say that there is any limitation that would invalidate the results for sure.
**First Author (year):** Fleming (2004)[16]<br>**Design:** Retrospective cohort<br>**Setting:** Throughout United States (Matria database)<br>(Jun 1992–Jun 2000)<br>**Funding Source:** NR	**n =** 284<br>**Mean Maternal Age ± SD (years):** NR<br>**Mean Gestational Age ± SD (weeks):** 30.4 ± 2.6 (P)<br>**Gestation:** singletons<br>**Primary Tocolytic Treatment:** NR<br>**Previous Maintenance Tocolytics:** Oral Nifedipine<br>**Inclusion Criteria:** singleton gestation; prescribed nifedipine following an initial episode of preterm labor; subsequent hospitalization for RPTL at <34 weeks; stabilized by tocolysis per attending physician's plan of treatment; outpatient tocolysis resumed with nifedipine or continuous subcutaneous terbutaline<br>**Exclusion Criteria:** subjects who could not be matched by gestational age	NR	**C:** Oral nifedipine (142): 66.7 ± 37.1<br>**I:** SQ terbutaline (142): 3.2 ± 1.6	Medium<br>There is considerable missing information, which makes it difficult to assess the comparability of groups. There is some indication that there are baseline differences (i.e., in age and marital status) and data on many other important factors have not been reported (e.g., cervical length, race, SES). However, there are no major flaws that can be singled out as invalidating the results.

Table F1. Detailed study-level characteristics (continued)

Study Identification	Study Population	Definition of Preterm Labor	Comparison Group (N): Mean Daily Dose ± SD (mg) Intervention Group (N): Mean Daily Dose ± SD (mg)	Overall Risk of Bias With Explanation *(rating applies to all outcomes unless specified otherwise)*
**First Author (year):** Lam (2003)[17] **Design:** Retrospective Cohort **Setting:** Throughout United States (Matria database) (Apr 1995 – Jan 1999) **Funding Source:** NR	**n =** 558 **Mean Maternal Age ± SD (years):** 27.4 ± 5.9 **Mean Gestational Age ± SD (weeks):** 31.6 ± 2.2 (P) **Gestation:** singletons **Primary Tocolytic Treatment:** NR **Previous Maintenance Tocolytics:** NR **Inclusion Criteria:** Singleton gestation; initial episode of preterm labor at > 20 weeks; subsequent hospitalization for RPTL < 35 weeks; stabilized and discharged home following RPTL **Exclusion Criteria:** Not prescribed tocolytics; lost to followup; medically indicated delivery	NR	**C:** Oral tocolytics (95.3% received oral terbutaline) (279): mean oral terbutaline dose 24.0 ± 9.3 **I:** SQ terbutaline (279): 3.5 ± 1.1	High Primary flaw is that groups were not similar at baseline (differed in smoking status and previous PTD). Also, missing data makes it difficult to assess several other potential limitations.
**First Author (year):** Lam (2001)[18] **Design:** Retrospective Cohort **Setting:** Throughout United States (Matria database) (Jan 1992 – Jul 1998) **Funding Source:** NR	**n =** 706 **Mean Maternal Age ± SD (years):** 28.8 ± 5.5 **Mean Gestational Age ± SD (weeks):** 31.3 ± 2.3 (P) **Gestation:** twins **Primary Tocolytic Treatment:** NR **Previous Maintenance Tocolytics:** NR **Inclusion Criteria:** Twin gestation; initial episode of preterm labor which was treated with oral tocolysis; hospitalized for RPTL at < 35 weeks gestation; stabilized on an inpatient basis for RPTL and then discharged to outpatient services **Exclusion Criteria:** Delivered after RPTL; remained hospitalized; discharged from outpatient services	NR	**C:** Oral tocolytics (92.3% received oral terbutaline) (353): mean oral terbutaline dose 25.6 ± 10.4 **I:** SQ terbutaline (353): 3.9 ± 1.4	Medium There is a large amount of missing information, which makes it difficult to assess the comparability of groups and other potential limitations. But there are no major flaws that can be identified that would invalidate the results.

Table F1. Detailed study-level characteristics (continued)

Study Identification	Study Population	Definition of Preterm Labor	Comparison Group (N): Mean Daily Dose ± SD (mg) Intervention Group (N): Mean Daily Dose ± SD (mg)	Overall Risk of Bias With Explanation *(rating applies to all outcomes unless specified otherwise)*
**First Author (year):** Allbert (1994)[19]<br>**Design:** Retrospective Cohort<br>**Setting:** NR<br>**Funding Source:** Vicksburg Hospital Medical Foundation (supported in part)<br>**Companion Article:**[42]	**n =** 64<br>**Mean Maternal Age ± SD (years):** 27.5 ± 4.3<br>**Mean Gestational Age ± SD (weeks):** 32.2 ± 2.7 (T)<br>**Gestation:** Not specified (likely included a mixture of women with single and multiple gestation)<br>**Primary Tocolytic Treatment:** NR<br>**Previous Maintenance Tocolytics:** NR<br>**Inclusion Criteria:** Documented RPTL; at 20–34 weeks' gestation; between the ages of 15 and 45 years<br>**Exclusion Criteria:** Continuation of pregnancy contraindicated (fetal distress, chorioamnionitis, intrauterine growth retardation, abruption, preeclampsia, etc.); insulin-dependent diabetes mellitus; allergy to beta-sympathomimetic drugs; premature rupture of membranes; cardiac arrhythmia; significant hemorrhage; fetal anomalies; fetal demise	Persistent uterine contractions and progressive cervical change.	**C:** Oral terbutaline (32): NR<br>**I:** SQ terbutaline (32): NR	Medium<br>There is a lot of missing information, which makes it difficult to assess comparability among groups and whether groups were derived from the same population. There is a possibility that groups received a different level of care, since only the subcutaneous terbutaline group has been specified as receiving home nursing care. However, it is unclear if this factor alone would be sufficient to impact the results to a large extent.

Table F1. Detailed study-level characteristics (continued)

Study Identification	Study Population	Definition of Preterm Labor	Comparison Group (N): Mean Daily Dose ± SD (mg) Intervention Group (N): Mean Daily Dose ± SD (mg)	Overall Risk of Bias With Explanation *(rating applies to all outcomes unless specified otherwise)*
**First Author (year):** Regenstein (1993)[20]‡<br>**Design:** Retrospective cohort<br>**Setting:** NR (Dec 1986–Jan 1992)<br>**Funding Source:** National Institutes of Health Training	**n =** 69<br>**Mean Maternal Age ± SD (years):** 31.4 ± 5.9<br>**Mean Gestational Age ± SD (weeks):** NR<br>**Gestation:** Not specified (included a mixture of women with single and multiple gestation)<br>**Primary Tocolytic Treatment:** NR<br>**Previous Maintenance Tocolytics:** NR<br>**Inclusion Criteria:** Neceiving home nursing care or care by perinatology service; gestational diabetes screening performed after initiation of chronic terbutaline tocolysis<br>**Exclusion Criteria:** NR	NR	**C:** Oral terbutaline (38): 25.9 ± 11.2<br>**I:** SQ terbutaline (31): 2.5 ± 1.0	High<br>Although the harm outcome of maternal hyperglycemia was defined and collected actively, the primary flaw with this study is that groups were not similar in baseline characteristics (i.e., in race and family history of gestational diabetes). Also, since no methods were used to control for confounders, there is a high likelihood that groups may differ in other baseline characteristics and prognostic factors, which have not been reported. There is also a lot of missing information which makes it difficult to assess the comparability of groups (e.g., primary tocolytic, loss to followup, differential level of care, compliance).

Table F1. Detailed study-level characteristics (continued)

Study Identification	Study Population	Definition of Preterm Labor	Comparison Group (N): Mean Daily Dose ± SD (mg) Intervention Group (N): Mean Daily Dose ± SD (mg)	Overall Risk of Bias With Explanation *(rating applies to all outcomes unless specified otherwise)*
**First Author (year):** Adkins (1993)[21]<br>**Design:**<br>Case Series<br>**Setting:** An urban obstetrics and gynecology group practice, Tennessee<br>(Nov 1989–Feb 1991)<br>**Funding Source:** PharmaThera Inc.	**n =** 51<br>**Mean Maternal Age ± SD (years):** 31.0 ± 4.0<br>**Mean Gestational Age ± SD (weeks):** 29.1 ± 3.6 (T)<br>**Gestation:** singletons or twins<br>**Primary Tocolytic Treatment:** Magnesium sulfate (IV) or terbutaline (SC)<br>**Previous Maintenance Tocolytics:** Oral tocolytics<br>*(only received by some patients)*<br>**Inclusion Criteria:** 20 to 35 weeks gestation; established diagnosis of preterm labor; intact membranes; cervical dilation ≤ 4 cm<br>**Exclusion Criteria:** Contraindication to terbutaline therapy (abnormal fetal heart rate pattern, complete abruption placentae, chorioamnionitis, and progressive preeclampsia).	Uterine contractions > four per hour and progressive cervical change.	**I:** SQ terbutaline (51): NR	Medium<br>There is missing information, which makes it difficult to assess some quality items. However, there was no high loss to followup and subjects were representative of source population. Adequacy of sample size is unclear (n=51), although it is larger than the previous case series of nine subjects.

Table F1. Detailed study-level characteristics (continued)

Study Identification	Study Population	Definition of Preterm Labor	Comparison Group (N): Mean Daily Dose ± SD (mg) Intervention Group (N): Mean Daily Dose ± SD (mg)	Overall Risk of Bias With Explanation *(rating applies to all outcomes unless specified otherwise)*
**First Author (year):** Lam (1988)[22]<br>**Design:**<br>Case Series<br>**Setting:** NR<br>**Funding Source:** NR	**n =** 9<br>**Mean Maternal Age ± SD (years):** NR<br>**Mean Gestational Age ± SD (weeks):** 29.6 ± 3.7 (T)<br>**Gestation:** not specified<br>**Primary Tocolytic Treatment:** Magnesium Sulfate (IV)<br>**Previous Maintenance Tocolytics:** Oral Terbutaline<br>**Inclusion Criteria:** had RPTL during oral terbutaline treatment; intact membranes; cervical dilation < 4 cm; absence of fetal distress or anomalies; absence of maternal disease with which magnesium sulfate or beta-mimetic tocolysis might interfere<br>**Exclusion Criteria:** NR	Regular uterine contractions > four per hour leading to progressive cervical change.	**I:** SQ terbutaline (9): NR	Medium<br>There is a lot of missing information, which makes it difficult to assess potential for selection bias (e.g., were the nine subjects in the study the entire sample, or were these the number left over after losses to followup?). Also, harm outcomes have not been defined. However, the study does not have any obvious major flaws, which would invalidate the results.

SC = subcutaneous; IV = intravenous; NR = not reported; PTL = preterm labor; SD = standard deviation; RPTL = recurrent preterm labor; RCT = randomized controlled trial; SQ = subcutaneous

* Either at preterm labor (indicated by P) or at start of subcutaneous terbutaline therapy (indicated by T). If study population stated RPTL as an inclusion criterion, then this is the gestational age at the episode of RPTL.

† Received by entire study population, unless specified otherwise.

‡ Data from a third treatment arm, which consisted of a control group without preterm labor, has not been presented.

Table F2. Full-text question posed for criteria listed in risk of bias charts

Risk of Bias Chart	Full Question
Baseline characteristics/ prognostic factors	Are the treatment and comparison groups similar in terms of baseline characteristics and prognostic factors?
Primary tocolytic agent(s)	Did participants in the treatment and comparison groups receive the same (or a similar distribution of) primary tocolytic to control their acute episode of preterm labor?
Level of care	Was there a differential level of care (e.g., home uterine contraction monitoring, education, nurse visits, individualized dosing schedules, other co-interventions) between the treatment and comparison groups?
Population used to sample comparison and treatment groups	If this is an observational study or a nonrandomized trial, is the sample population from which the comparison group(s) was drawn the same as the sample population from which the treatment group was drawn?
Loss to followup	Was there either: (i) a differential loss to followup between the compared groups; or (ii) an overall high loss to followup?
Compliance with study protocol	Was compliance with the study protocol (i.e., treatment or comparator intervention) adequate in all study groups?
Methods to control for confounders	If this is an observational study or a nonrandomized trial, were appropriate methods undertaken to control for important confounders (e.g., matching)?
Representativeness of subjects to source	Were the subjects who were included in the study representative of the source population? For instance, subjects would be representative if the entire source population was recruited for the study, if a sample of consecutive patients was recruited, or if a random sample was obtained.
Blinding of patients to treatment allocation	If this is an experimental study, were patients blinded to treatment allocation?
Blinding of health care providers to treatment allocation	If this is an experimental study, were healthcare providers blinded to treatment allocation?
Blinding of outcome assessors to treatment allocation	If this is an experimental study, was the outcome assessor blinded to treatment allocation?
Blinding of health care providers to maternal contractions	If this is an experimental study, were healthcare providers blinded to the frequency and intensity of maternal contractions?
Generation of allocation sequence	If this is a randomized controlled trial, was the allocation sequence adequately generated?
Concealment of allocation sequence	If this is a randomized controlled trial, was the process of concealing the random allocation sequence adequate?
Prediction of future intervention assignments by study personnel	If this is a randomized controlled trial, at the time of study enrollment is there any indication that study personnel were able to predict future intervention assignments?
Intention-to-treat analysis	Was an intention-to-treat analysis conducted?
Sample size	Was the sample size adequate to determine a difference in outcomes between comparison groups or between pre and post intervention?
Selective outcome reporting	Is there any indication of selective outcome reporting?
Reliability in outcome assessment (if multiple outcome assessors used)	If multiple outcome assessors were used, is it likely there was high reliability in outcome assessment between all assessors (e.g., inter-rater reliability testing was conducted and adequate)?
Consistency in outcome definition	If this is a retrospective study that used multiple data sources, is it likely there was consistency in outcome definition across those data sources?
Definition of primary outcome(s)	Were the primary outcomes in the study defined by either prespecified or standardized clinical definitions?
Prespecification of harm outcomes	For studies assessing maternal or neonatal harms: If the harm outcomes assessed in the study are not generally known to have standard definitions, then were these harms predefined using standardized or precise definitions?
Reporting of harm outcomes as active	If this is a study assessing maternal or neonatal harms, was the mode of harms collection specified as active (versus passive)?
Reporting of training/background of personnel collecting harms data	If this is a study assessing maternal or neonatal harms, did the report specify who collected harms data, including their training and background?

Table F3. Detailed risk of bias assessments for individual studies

Study Design: Author (year)	Risk of Bias Criteria Rated Negatively	Risk of Bias Criteria Rated Positively	Risk of Bias Criteria Rated as Unclear	Final Rating by Outcome<br>*(rating applies to all outcomes unless indicated otherwise)*
RCT: Guinn (1998)[9]		Groups similar in primary tocolytic therapy.<br>Patients blinded to treatment allocation.<br>Health care providers blinded to treatment allocation/Outcome assessor blinded to treatment allocation (assumed to be same as health care providers).<br>Intention-to-treat analysis conducted.<br>No differential or high loss to followup.<br>Sample size adequate.<br>No differential level of care among groups.<br>No indication of selective outcome reporting.<br>Allocation sequence was generated adequately.<br>Allocation sequence concealed adequately.<br>No indication that study personnel could predict future intervention assignments.<br>Measured harms with standardized definition (maternal discontinuation of therapy).<br>Mode of harms collection not explicitly specified as active. However, not relevant for harm of discontinuation of therapy.<br>Report does not explicitly specify who collected harms data. However, not relevant for harm of discontinuation of therapy.<br>Primary outcome (prolongation of pregnancy) has been defined.	If groups were similar in baseline characteristics and prognostic factors.<br>If health care providers were blinded to maternal contractions.<br>If there was reliability among multiple outcome assessors (unclear if there were multiple outcome assessors).<br>If compliance with study protocol was adequate.<br>Representativeness of subjects to source population.	**Outcomes:**<br>(1) Maternal discontinuation of therapy<br>(2) NICU admission<br>(3) Intraventricular hemorrhage<br>(4) Prolongation of pregnancy<br>(5) Gestational age at delivery<br>(6) Birth weight<br>**LOW:** The comparability of groups cannot be assessed for certain because information on all relevant factors has not been presented (e.g., prognostic factors, like cervical length and fetal fibronectin). However, randomization was carried out properly and patients/health care providers were blinded to treatment allocation, which will limit selection and detection biases.

Table F3. Detailed risk of bias assessments for individual studies (continued)

Study Design: Author (year)	Risk of Bias Criteria Rated Negatively	Risk of Bias Criteria Rated Positively	Risk of Bias Criteria Rated as Unclear	Final Rating by Outcome<br>*(rating applies to all outcomes unless indicated otherwise)*
RCT: Wenstrom (1997)[10]<br>*Saline pump arm*	Patients were not adequately blinded (the intention was to blind, but 60% in terbutaline pump group and 67% in saline pump group had to be unblinded).<br>Healthcare providers were not adequately blinded (the intention was to blind, but 60% in terbutaline pump group and 67% in saline pump group had to be unblinded)/Same consideration applies to outcome assessors, since they are assumed to be the same as healthcare providers.<br>Sample size too small.<br>Harms outcomes do not have standard clinical definitions and were not predefined (local skin irritation, local pain, neonatal hypoglycemia).<br>Mode of harms collection not specified as active.<br>Report does not specify who collected harms, including training and background.<br>Subjects were not representative of source population because >90% of eligible subjects declined to participate.	Intention-to-treat analysis conducted.<br>No differential or high loss to followup.<br>Allocation sequence generated adequately.<br>Allocation sequence concealed adequately.<br>No indication that study personnel could predict future intervention assignments.	If groups were similar in baseline characteristics and prognostic factors.<br>If groups were similar in primary tocolytic therapy.<br>If health care providers were blinded to maternal contractions.<br>If there was differential level of care among groups.<br>If there was selective outcome reporting.<br>If there was reliability among multiple outcome assessors (unclear if there were multiple assessors).<br>If compliance with study protocol was adequate.	**Outcomes:**<br>(1) Gestational age at delivery<br>(2) Birth weight<br>(3) Prolongation of pregnancy<br>(4) Local skin irritation<br>(5) Local pain<br>(6) Neonatal hypoglycemia<br>(7) Sepsis<br>(8) Retinopathy of prematurity<br>(9) NICU<br>(10) Perinatal deaths<br>**HIGH:** The sample likely represents a very select group, since >90% of eligible subjects declined to participate. The study is likely to be underpowered. There is evidence that randomization was carried out properly, but blinding was not that effective. Missing information makes it difficult to judge comparability of groups in baseline characteristics and prognostic factors, primary tocolytic therapy, and level of care.

Table F3. Detailed risk of bias assessments for individual studies (continued)

Study Design: Author (year)	Risk of Bias Criteria Rated Negatively	Risk of Bias Criteria Rated Positively	Risk of Bias Criteria Rated as Unclear	Final Rating by Outcome *(rating applies to all outcomes unless indicated otherwise)*
RCT: Wenstrom (1997)[10]<br><br>*Oral terbutaline arm*	Patients were not blinded to treatment allocation.<br><br>Health care providers were not blinded to treatment allocation/Same applies to outcome assessors since they are assumed to be the same as healthcare providers.<br><br>Sample size too small.<br><br>Harm outcomes do not have standard clinical definitions and were not predefined (local skin irritation, local pain, neonatal hypoglycemia).<br><br>Mode of harms collection not specified as active.<br><br>Report does not specify who collected harms, including training and background.<br><br>Subjects were not representative of source population because >90% of eligible subjects declined to participate.	Intention-to-treat analysis conducted.<br><br>No differential or high loss to followup.<br><br>Allocation sequence generated adequately.<br><br>Allocation sequence concealed adequately.<br><br>No indication that study personnel could predict future intervention assignments.	If groups were similar in baseline characteristics and prognostic factors.<br><br>If groups were similar in primary tocolytic therapy.<br><br>If healthcare providers were blinded to maternal contractions.<br><br>If there was differential level of care among groups.<br><br>If there was selective outcome reporting.<br><br>If there was reliability among multiple outcome assessors (unclear if there were multiple assessors).<br><br>If compliance with study protocol was adequate.	**Outcomes:**<br><br>(1) Gestational age at delivery<br><br>(2) Birth weight<br><br>(3) Prolongation of pregnancy<br><br>(4) Local skin irritation<br><br>(5) Local pain<br><br>(6) Neonatal hypoglycemia<br><br>(7) Sepsis<br><br>(8) Retinopathy of prematurity<br><br>(9) NICU<br><br>(10) Perinatal deaths<br><br>**HIGH:** The sample likely represents a very select group, since >90% of eligible subjects declined to participate. The study is likely to be underpowered. There is evidence that randomization was carried out properly, but patient and healthcare providers were not blinded to treatment allocation. Missing information makes it difficult to judge comparability of groups in baseline characteristics and prognostic factors, primary tocolytic therapy, and level of care.

Table F3. Detailed risk of bias assessments for individual studies (continued)

Study Design: Author (year)	Risk of Bias Criteria Rated Negatively	Risk of Bias Criteria Rated Positively	Risk of Bias Criteria Rated as Unclear	Final Rating by Outcome *(rating applies to all outcomes unless indicated otherwise)*
Nonrandomized Trial: Lindenbaum (1992)[11]	Patients were not blinded to treatment allocation.<br><br>Health care providers not blinded to treatment allocation/Same applies to outcome assessors, since they are assumed to be the same as health care providers.<br><br>Health care providers not blinded to maternal contractions.<br><br>Comparison group not drawn from same population as treatment group.<br><br>Appropriate methods not taken to control for confounders.	No differential or high loss to followup.<br><br>No indication of selective outcome reporting.<br><br>Harm outcome (maternal hyperglycemia) was pre-defined.<br><br>Mode of harms collection was specified as active.<br><br>Report does not explicitly specify who collected harms data. However, GTT results will likely be obtained by trained laboratory personnel and interpreted by qualified healthcare professionals.<br><br>Subjects were representative of source population.<br><br>Primary outcome (maternal hyperglycemia) has been defined.	If groups were similar in baseline characteristics and prognostic factors.<br><br>If groups were similar in primary tocolytic treatment.<br><br>If an intention-to-treat analysis was conducted.<br><br>If sample size was adequate.<br><br>If there was differential level of care among groups.<br><br>If there was reliability among multiple outcome assessors (unclear if there were multiple assessors).<br><br>If compliance with study protocol was adequate.	**Outcomes:**<br><br>(1) Gestational age at delivery<br><br>(2) Birth weight<br><br>(3) Maternal hyperglycemia<br><br>**HIGH** (birthweight and gestational age at delivery): Primary flaw in this study is the difference in groups with respect to severity/prognosis (i.e., groups were divided based on length of primary tocolytic treatment). Also, comparability of groups cannot be assessed due to missing information.<br><br>**MEDIUM** (maternal hyperglycemia): The potential difference in severity/prognosis among treatment and comparison groups should not impact the outcome of maternal hyperglycemia. However, issues pertaining to missing information still remain.

Table F3. Detailed risk of bias assessments for individual studies (continued)

Study Design: Author (year)	Risk of Bias Criteria Rated Negatively	Risk of Bias Criteria Rated Positively	Risk of Bias Criteria Rated as Unclear	Final Rating by Outcome *(rating applies to all outcomes unless indicated otherwise)*
Prospective Cohort: Morrison (2003)[12]	Groups differ in baseline characteristics and prognostic factors (in particular, differ in risk factors for preterm birth).<br>Groups differ in primary tocolytic.<br>Differential level of care received by groups (only terbutaline group received home uterine contraction monitoring.<br>Indication of selective outcome reporting (amount terbutaline infused and neonatal morbidity not reported).<br>Methods to control for confounders insufficient (matched for several factors, but there are still differences in the risk factors for preterm birth).<br>Mode of harms collection not specified as active.	No differential or high loss to followup.<br>Measured harms with standard definitions (maternal arrhythmia and maternal discontinuation of therapy).<br>Report does not explicitly specify who collected harms data. However, it can be assumed that arrhythmia would be detected by qualified healthcare professionals.<br>Subjects were representative of source population.	If an intention-to-treat analysis was conducted.<br>If sample size adequate.<br>If there was reliability among multiple outcome assessors (unclear if there were multiple outcome assessors).<br>If compliance with study protocol was adequate.<br>If comparison group was drawn from same population as treatment group.	**Outcomes:**<br>(1) Maternal arrhythmia<br>(2) Maternal discontinuation of therapy<br>(3) Gestational age at delivery<br>(4) Prolongation of pregnancy<br>(5) PPI<br>(6) Birth weight<br>(7) Intraventricular hemorrhage<br>(8) Necrotizing enterocolitis<br>(9) NICU admission<br>**HIGH:** Primary flaw with this study is that there is evidence that groups were not comparable (with respect to risk factors for preterm birth, primary tocolytic therapy, level of care).

Table F3. Detailed risk of bias assessments for individual studies (continued)

Study Design: Author (year)	Risk of Bias Criteria Rated Negatively	Risk of Bias Criteria Rated Positively	Risk of Bias Criteria Rated as Unclear	Final Rating by Outcome<br>*(rating applies to all outcomes unless indicated otherwise)*
Prospective Cohort: Morrison (1992)[13]	Groups were not similar in baseline characteristics and prognostic factors (subcutaneous terbutaline group had RPTL but other group did not).<br>Comparison group not drawn from the same population as treatment group.<br>Appropriate methods not taken to control for confounders.	No indication of selective outcome reporting.<br>Primary outcome has been defined (interval from discontinuance of tocolytic to spontaneous labor).	If groups were similar in primary tocolytic treatment.<br>If an intention-to-treat analysis was conducted.<br>If there was differential or high loss to followup.<br>If sample size was adequate.<br>If there was differential level of care among groups.<br>If there was reliability among multiple outcome assessors (unclear if there were multiple assessors).<br>If compliance with study protocol was adequate.<br>If subjects were representative of source population.	**Outcomes:**<br>(1) Gestational age at delivery<br>**HIGH:** Major flaw is that the subcutaneous pump group had RPTL and comparison group did not. Therefore, the intervention group may have had a more serious condition. Also, there is missing information, which makes it difficult to assess other potential limitations.
Retrospective Cohort: Flick (2010)[14]	Groups were not similar in baseline characteristics and prognostic factors (in particular, differed in smoking status).<br>Appropriate methods not undertaken to control for confounders.	No differential or high loss to followup.<br>No differential level of care.<br>No indication of selective outcome reporting.<br>Comparison group drawn from the same population as treatment group.<br>Subjects were representative of source population.<br>Primary outcome (prolongation of pregnancy) has been defined.	If groups were similar in primary tocolytic therapy.<br>If an intention-to-treat analysis was conducted.<br>If sample size was adequate.<br>If there was reliability among multiple outcome assessors (data from Matria database, so likely there were multiple outcome assessors, but cannot determine reliability among them).<br>If compliance with study protocol was adequate.	**Outcomes:**<br>(1) Prolongation of pregnancy<br>(2) Gestational age at delivery<br>(3) Birth weight<br>(4) NICU admission<br>**HIGH:** Primary flaw is that groups were not similar in baseline characteristics and prognostic factors (i.e. differed in smoking status). Also, missing information makes it difficult to assess similarity of groups with respect to other factors.

Table F3. Detailed risk of bias assessments for individual studies (continued)

Study Design: Author (year)	Risk of Bias Criteria Rated Negatively	Risk of Bias Criteria Rated Positively	Risk of Bias Criteria Rated as Unclear	Final Rating by Outcome<br>*(rating applies to all outcomes unless indicated otherwise)*
Retrospective Cohort:de la Torre (2008)[15]	No methods to control for confounders.	No differential or high loss to followup.<br>No differential level of care between groups.<br>No indication of selective outcome reporting.<br>Comparison and treatment groups drawn from same sample population.<br>Subjects were representative of source population.<br>Primary outcome (prolongation of pregnancy) was defined.	If groups were similar in baseline characteristics and prognostic factors.<br>If groups were similar in primary tocolytic therapy.<br>If intention-to-treat analysis conducted.<br>If sample size adequate.<br>If there was reliability among multiple outcome assessors (likely that there were multiple outcome assessors, since women were from the Matria database. But reliability among assessors cannot be assessed).<br>If compliance with study protocol was adequate.	**Outcomes:**<br>(1) Prolongation of pregnancy<br>(2) Gestational age at delivery<br>(3) Birth weight<br>(4) NICU admission<br>**MEDIUM:** There is a lot of missing information, which makes it difficult to assess comparability of groups (in terms of baseline characteristics and prognostic factors, primary tocolytic therapy, and compliance). But difficult to say that there is any limitation that would invalidate the results for sure.

Table F3. Detailed risk of bias assessments for individual studies (continued)

Study Design: Author (year)	Risk of Bias Criteria Rated Negatively	Risk of Bias Criteria Rated Positively	Risk of Bias Criteria Rated as Unclear	Final Rating by Outcome<br>*(rating applies to all outcomes unless indicated otherwise)*
Retrospective Cohort: Fleming (2004)[16]	Primary outcome of gestational age < 35 weeks has not been adequately specified (i.e., method for determining gestational age not described).	No differential level of care.<br>No indication of selective outcome reporting.<br>Comparison group drawn from same population as treatment group.	If groups were similar in baseline characteristics and prognostic factors.<br>If groups were similar in primary tocolytic therapy.<br>If intention-to-treat analysis conducted.<br>If there was differential or high loss to followup.<br>If sample size was adequate.<br>If there was reliability in outcome assessors (likely that there were multiple outcome assessors, since the Matria database was used. But reliability among assessors cannot be determined).<br>If compliance with study protocol was adequate.<br>If appropriate methods were used to control for important confounders.<br>If subjects were representative of source population.	**Outcomes:**<br>(1) Pregnancy prolongation<br>(2) Gestational age at delivery<br>(3) Stillbirths/neonatal deaths<br>(4) NICU admission<br>(5) Birth weight<br>**MEDIUM:** There is considerable missing information, which makes it difficult to assess the comparability of groups. There is some indication that there are baseline differences (i.e., in age and marital status) and data on many other important factors have not been reported (e.g., cervical length, race, SES). However, there are no major flaws that can be singled out as invalidating the results.

Table F3. Detailed risk of bias assessments for individual studies (continued)

Study Design: Author (year)	Risk of Bias Criteria Rated Negatively	Risk of Bias Criteria Rated Positively	Risk of Bias Criteria Rated as Unclear	Final Rating by Outcome *(rating applies to all outcomes unless indicated otherwise)*
Retrospective Cohort: Lam (2003)[17]	Groups differ in baseline characteristics and prognostic factors (in particular: smoking status and previous preterm delivery).<br><br>Intention-to-treat analysis not done (losses to followup excluded).<br><br>Methods were not sufficient to control for confounders (only matched by gestational age at delivery).	No differential level of care between groups.<br><br>Comparison group drawn from the same sample population as treatment group.<br><br>Measured harms with standardized definitions (maternal pulmonary edema and maternal death).<br><br>Mode of harms collection not explicitly specified as active. However, this is not very relevant for outcomes of pulmonary edema and maternal death.<br><br>Report does not explicitly specify who collected harms data. However, it is reasonable to assume that pulmonary edema would be assessed by qualified healthcare professionals.	If groups were similar in primary tocolytic therapy.<br><br>If there was differential or high loss to followup (losses to followup were excluded).<br><br>If sample size was adequate.<br><br>If there was selective outcome reporting.<br><br>If there was reliability in outcome assessors (data was from Matria database, so likely that there were multiple outcome assessors. But reliability among assessors cannot be determined).<br><br>If compliance with study protocol was adequate.<br><br>If subjects were representative of source population.	**Outcomes:**<br><br>(1) Pregnancy prolongation<br><br>(2) Gestational age at delivery<br><br>(3) Birth weight<br><br>(4) NICU admission<br><br>(5) Stillbirth<br><br>(6) Ventilator required<br><br>(7) Maternal pulmonary edema<br><br>(8) Maternal deaths<br><br>**HIGH:** Primary flaw is that groups were not similar at baseline (differed in smoking status and previous PTD). Also, missing data makes it difficult to assess several other potential limitations.

Table F3. Detailed risk of bias assessments for individual studies (continued)

Study Design: Author (year)	Risk of Bias Criteria Rated Negatively	Risk of Bias Criteria Rated Positively	Risk of Bias Criteria Rated as Unclear	Final Rating by Outcome *(rating applies to all outcomes unless indicated otherwise)*
Retrospective Cohort: Lam (2001)[18]		No high or differential loss to followup.<br>No differential level of care among groups.<br>Comparison group drawn from same population as treatment group.<br>Measured harms with standard definitions (maternal pulmonary edema and maternal deaths).<br>Mode of harms collection not explicitly specified as active. However, this is not very relevant for harms of maternal pulmonary edema and maternal death.<br>Report does not explicitly specify who collected harms data. However, it can be assumed that pulmonary edema and death would be assessed by qualified personnel.<br>Subjects were representative of source population.	If groups were similar in baseline characteristics and prognostic factors.<br>If groups were similar in primary tocolytic therapy.<br>If an intention-to-treat analysis was conducted.<br>If sample size was adequate.<br>If there was selective outcome reporting.<br>If there was reliability among multiple outcome assessors (data from Matria database, so likely there were multiple outcome assessors, but reliability among assessors cannot be determined).<br>If compliance with study protocol was adequate.<br>If appropriate methods used to control for confounders (matched by gestational age at hospitalization for recurrent preterm labor).	**Outcomes:**<br>(1) Prolongation of pregnancy<br>(2) Gestational age at delivery<br>(3) Birth weight<br>(4) NICU admission<br>(5) Stillbirth/neonatal deaths<br>(6) Maternal pulmonary edema<br>(7) Maternal deaths<br>**MEDIUM:** There is a large amount of missing information, which makes it difficult to assess the comparability of groups and other potential limitations. But there are no major flaws that can be identified that would invalidate the results.

Table F3. Detailed risk of bias assessments for individual studies (continued)

Study Design: Author (year)	Risk of Bias Criteria Rated Negatively	Risk of Bias Criteria Rated Positively	Risk of Bias Criteria Rated as Unclear	Final Rating by Outcome<br>*(rating applies to all outcomes unless indicated otherwise)*
Retrospective Cohort: Allbert (1994)[19]	Differential level of care among groups (it appears that only the subcutaneous terbutaline group received home nursing care).	No indication of selective outcome reporting.<br>Consistency in outcome definition among multiple data sources (Not clear if multiple data sources were used. However, use of multiple data sources should not make much of a difference because all outcomes have been defined or are self-explanatory).<br>Primary outcome defined (gestational age ≥ 37 weeks and method for determining gestational age specified).	If groups were similar in baseline characteristics and prognostic factors.<br>If groups were similar in primary tocolytic therapy.<br>If an intention-to-treat analysis was conducted.<br>If there was high or differential loss to followup.<br>If sample size was adequate.<br>Reliability among multiple outcome assessors (unclear of there were multiple assessors).<br>If compliance with study protocol was adequate.<br>If comparison group came from same sample population as treatment group.<br>If appropriate methods were undertaken to control for confounders (matched for age, race, parity, gestational age and cervical dilation at the diagnosis of recurrent labor).<br>If subjects were representative of source population.	**Outcomes:**<br>(1) Gestational age at delivery<br>(2) PPI<br>(3) Birth weight<br>**MEDIUM:** There is a lot of missing information, which makes it difficult to assess comparability among groups and whether groups were derived from the same population. There is a possibility that groups received a different level of care, since only the subcutaneous terbutaline group has been specified as receiving home nursing care. However, it is unclear if this factor alone would be sufficient to impact the results to a large extent.

Table F3. Detailed risk of bias assessments for individual studies (continued)

Study Design: Author (year)	Risk of Bias Criteria Rated Negatively	Risk of Bias Criteria Rated Positively	Risk of Bias Criteria Rated as Unclear	Final Rating by Outcome *(rating applies to all outcomes unless indicated otherwise)*
Retrospective Cohort:<br><br>Regenstein (1993)[20]	Groups are not similar in baseline characteristics and prognostic factors.<br><br>No methods to control for confounders.	No bias due to study funding.<br><br>No indication of selective outcome reporting.<br><br>Harm outcome (maternal hyperglycemia) was predefined using precise definition based on 3-hour GTT.<br><br>Harms data collection was specified as active versus passive.<br><br>Report does not explicitly specify who collected harms data, including their training and background. However, GTT results will likely be obtained by trained laboratory personnel and interpreted by qualified health care professionals.<br><br>Primary outcome (glucose intolerance i.e. maternal hyperglycemia) is defined based on 1-hour and 3-hour GTT.	If groups were similar in primary tocolytic therapy.<br><br>If intention-to-treat analysis conducted.<br><br>If there was differential or high loss to followup.<br><br>If sample size adequate.<br><br>If there was differential level of care between groups.<br><br>If there was reliability among multiple outcome assessors (unclear if there were multiple outcome assessors).<br><br>If compliance with study protocol was adequate.<br><br>If comparison group was drawn from the same population as treatment group.<br><br>If subjects were representative of source population.	**Outcomes:**<br><br>(1) Maternal hyperglycemia (gestational diabetes)<br><br>(2) Gestational age at delivery<br><br>(3) Birth weight<br><br>HIGH (Maternal hyperglycemia - harm outcome):<br>Although this harm outcome was defined and collected actively, the primary flaw with this study is that groups were not similar in baseline characteristics (i.e., in race and family history of gestational diabetes). Also, since no methods were used to control for confounders, there is a high likelihood that groups may differ in other baseline characteristics and prognostic factors, which have not been reported. There is also a lot of missing information which makes it difficult to assess the comparability of groups (e.g. primary tocolytic, loss to followup, differential level of care, compliance).<br><br>HIGH (all other outcomes): same reasons as above.

Table F3. Detailed risk of bias assessments for individual studies (continued)

Study Design: Author (year)	Risk of Bias Criteria Rated Negatively	Risk of Bias Criteria Rated Positively	Risk of Bias Criteria Rated as Unclear	Final Rating by Outcome<br>*(rating applies to all outcomes unless indicated otherwise)*
Case series: Adkins (1993)[21]	Bias due to study funding (from PharmaThera Inc).<br>Harms were not predefined (pump malfunction and dislodgment).<br>Mode of harms collection not specified as active.<br>Report does not specify who collected harms data, including background and training.	No high loss to followup.<br>Subjects were representative of source population.	If sample size was adequate.<br>If there was selective outcome reporting.<br>If there was reliability among multiple outcome assessors (unclear if there were multiple assessors).<br>If compliance with study protocol was adequate.	**Outcomes:**<br>(1) Pump malfunction<br>(2) Dislodgment<br>**MEDIUM:** There is missing information, which makes it difficult to assess some quality items. However, there was no high loss to followup and subjects were representative of source population. Adequacy of sample size is unclear (n=51), although it is larger than the previous case series of nine subjects.
Case series: Lam (1988)[22]	Harm outcomes of mechanical failures/complications and infusion site infections have not been defined.<br>Harms data collection not specified as active.<br>Report does not specify who collected harms data, including their training and background.		If there was high loss to followup.<br>If sample size was adequate.<br>If there was selective outcome reporting.<br>If there was reliability among multiple outcome assessors (unclear if there were multiple assessors).<br>If compliance with study protocol was adequate.<br>If subjects were representative of source population.	**Outcomes:**<br>(1) Mechanical failures and complications<br>(2) Infusion site infection<br>**MEDIUM:** There is a lot of missing information, which makes it difficult to assess potential for selection bias (e.g., were the nine subjects in the study the entire sample, or were these the number left over after losses to followup?). Also, harm outcomes have not been defined. However, the study does not have any obvious major flaws, which would invalidate the results.

GTT = glucose tolerance test; NICU = neonatal intensive care unit; PPI = pregnancy prolongation index; PTD = preterm delivery; RCT = randomized controlled trial; SES = socioeconomic status

Table F4. Studies that reported neonatal health outcomes (Key Question 1)

Outcome	First Author (Year)	Study Design (n=Sample Size)	Mean Maternal Age (Years)	Mean GA (Weeks)*	Comparator(s)	Results—SQ Terbutaline Pump: % (n/N)	Results—Comparison: % (n/N)	Results—OR (95% CI)
BPD	N/A	N/A	N/A	N/A	N/A	N/A	N/A	N/A
Death, neonatal	Fleming[†,] (2004)[16]	Retrospective Cohort (n=284)	NR	30.4 ± 2.6 (P)	Oral nifedipine	0% (0/142)	0% (0/142)	1.00 (0.02, 50.75)
	Lam[†,‡] (2001)[18]	Retrospective Cohort (n=706)	28.8 ± 5.5	31.3 ± 2.3 (P)	Oral tocolytics (92.3% received oral terbutaline)	0.1% (1/706)	1.6% (11/706)	0.09 (0.01, 0.70)
	Wenstrom[§] (1997)[10]	RCT (n=42)	26.2 ± 5.3	30.4 ± 2.3 (T)	Placebo (C_1)<br>Oral terbutaline (C_2)	0% (0/19)	C_1: 0% (0/15)<br>C_2: 0% (0/16)	0.79 (0.01, 42.38)<br>0.85 (0.02, 45.00)
Death within initial hospitalization	N/A	N/A	N/A	N/A	N/A	N/A	N/A	N/A
IVH (Grade III/IV)	Morrison[†] (2003)[12]	Prospective Cohort (n=60)	25.6 ± 5.2	29.5 ± 2.3 (P)	No treatment	0% (0/15)	8.9% (4/45)	0.30 (0.02, 5.85)
	Guinn (1998)[9]	RCT (n=52)	21.6 ± 5.7	30.6 ± 2.8 (T)	Placebo	0% (0/23)[**]	0% (0/28)	1.21 (0.02, 63.48)
NEC	Morrison[†] (2003)[12]	Prospective Cohort (n=60)	25.6 ± 5.2	29.5 ± 2.3 (P)	No treatment	0% (0/15)	2.2% (1/45)	0.96 (0.04, 24.74)
PVL	N/A	N/A	N/A	N/A	N/A	N/A	N/A	N/A
Retinopathy of prematurity	Wenstrom[§] (1997)[10]	RCT (n=42)	26.2 ± 5.3	30.4 ± 2.3 (T)	Placebo (C_1) Oral terbutaline (C_2)	5.3% (1/19)	C_1: 0% (0/15)<br>C_2: 0% (0/16)	2.51 (0.10, 66.20)<br>2.68 (0.10, 70.31)
Seizures	N/A	N/A	N/A	N/A	N/A	N/A	N/A	N/A
Sepsis	Wenstrom[§] (1997)[10]	RCT (n=42)	26.2 ± 5.3	30.4 ± 2.3 (T)	Placebo (C_1) Oral terbutaline (C_2)	0% (0/19)	C_1: 0% (0/15)<br>C_2: 6.2% (1/16)	0.79 (0.01, 42.38)<br>0.26 (0.01, 6.97)

Table F4. Studies that reported neonatal health outcomes (Key Question 1) (continued)

Outcome	First Author (Year)	Study Design (n=Sample Size)	Mean Maternal Age (Years)	Mean GA (Weeks)*	Comparator(s)	Results—SQ Terbutaline Pump: % (n/N)	Results—Comparison: % (n/N)	Results—OR (95% CI)
Stillbirth	Fleming[†] (2004)[16]	Retrospective Cohort (n=284)	NR	30.4 ± 2.6 (P)	Oral nifedipine	1.4% (2/142)	0.7% (1/142)	2.01 (0.18, 22.47)
	Lam[†] (2003)[17]	Retrospective Cohort (n=558)	27.4 ± 5.9	31.6 ± 2.2 (P)	Oral tocolytics (95.3% received oral terbutaline)	0.4% (1/279)	0% (0/279)	3.01 (0.12, 74.23)
	Lam[†,‡] (2001)[18]	Retrospective Cohort (n=706)	28.8 ± 5.5	31.3 ± 2.3 (P)	Oral tocolytics (92.3% received oral terbutaline)	0.4% (3/706)	0.6% (4/706)	0.75 (0.17, 3.36)

BPD = bronchopulmonary dysplasia; CI = confidence interval; GA = gestational age; IVH = intraventricular hemorrhage; N/A = not applicable; NEC = necrotizing enterocolitis; NR = not reported; OR = odds ratio; PVL = periventricular leukomalacia; RCT = randomized controlled trial; SQ = subcutaneous

Note: Subjects were women with singleton gestation only, unless indicated otherwise.

[*] Either at preterm labor (indicated by P) or at start of subcutaneous terbutaline therapy (indicated by T). If study population stated RPTL as an inclusion criterion, then this is the gestational age at the episode of RPTL.

[†] Study population consisted exclusively of women with RPTL.

[‡] Study population consisted exclusively of women with twin gestation. Denominator is number of infants.

[§] Study population consisted of women with single and twin gestation. Denominator is number of infants.

[**] One infant born at 33 weeks' gestation was unavailable for followup.

Table F5. Studies that reported mean gestational age at delivery (Key Question 2)

Outcome	First Author (Year)	Study Design (n=Sample Size)	Mean Maternal Age (Years)	Mean GA (Weeks)*	Comparator(s)	Results—SQ Terbutaline Pump: Mean ± SD	Results—Comparison: Mean ± SD	Results—Difference in Means (95% CI)
Mean GA at delivery<br><br>*Results are reported as mean GA at delivery in weeks*	Flick† (2010)[14]	Retrospective cohort (n=1366)	28.7 ± 6.1	30.6 ± 2.9 (P)	Oral nifedipine	36.7 ± 1.9	36.0 ± 2.9	0.70 (0.42, 0.98)
	de la Torre†,** (2008) [15]	Retrospective cohort (n=656)	30.3 ± 5.8	30.1 ± 2.9 (P)	Oral nifedipine	34.8 ± 2.2	34.1 ± 2.5	0.70 (0.43, 0.97)
	Fleming† (2004)[16]	Retrospective cohort (n=284)	NR	30.4 ± 2.6 (P)	Oral nifedipine	36.6 ± 2.1	35.7 ± 3.1	0.90 (0.28, 1.52)
	Lam† (2003)[17]	Retrospective cohort (n=558)	27.4 ± 5.9	31.6 ± 2.2 (P)	Oral tocolytics (95.3% received oral terbutaline)	36.5 ± 2.1	35.7 ± 2.8	0.80 (0.39, 1.21)
	Morrison† (2003)[12]	Prospective cohort (n=60)	25.6 ± 5.2	29.5 ± 2.3 (P)	No treatment	36.7 ± 1.7	33.3 ± 3.0	3.40 (1.80, 5.00)
	Lam†,** (2001)[18]	Retrospective cohort (n=706)	28.8 ± 5.5	31.3 ± 2.3 (P)	Oral tocolytics (92.3% received oral terbutaline)	35.2 ± 2.0	34.5 ± 2.3	0.70 (0.48, 0.92)
	Guinn (1998)[9]	RCT (n=52)	21.6 ± 5.7	30.6 ± 2.8 (T)	Placebo	34.4 ± 3.4	34.9 ± 4.1	-0.50 (-2.57, 1.57)
	Wenstrom†† (1997)[10]	RCT (n=42)	26.2 ± 5.3	30.4 ± 2.3 (T)	Placebo (C_1) Oral terbutaline (C_2)	35.7 ± 3.0	C_1: 35.4 ± 3.0<br>C_2: 34.3 ± 4.0	0.30 (-1.73, 2.33)<br>1.40 (-0.92, 3.72)
	Regenstein‡‡ (1993)[20]	Retrospective cohort (n=69)	31.4 ± 5.9	NR	Oral terbutaline§	35.2 ± 3.3	36.6 ± 2.7	-1.40 (-2.82, 0.02)
	Lindenbaum (1992)[11]	Nonrandomized comparative trial (n=91)	32.4 ± 2.7	29.1 ± 1.7 (T)	Oral terbutaline§	36.6 ± 1.2	37.9 ± 1.3	-1.30 (-1.83, -0.77)‡‡‡
	Morrison***,††† (1992)[13]	Prospective cohort (n=69)	28.6 ± 4.7	NR	Oral tocolytics	37.5 ± 1.2	37.1 ± 0.96	0.40 (-0.11, 0.91)

CI = confidence interval; GA = gestational age; NR = not reported; RCT = randomized controlled trial; SD = standard deviation; SQ = subcutaneous

Note: Subjects were women with singleton gestation only, unless indicated otherwise.

* Either at preterm labor (indicated by P) or at start of subcutaneous terbutaline therapy (indicated by T). If study population stated RPTL as an inclusion criterion, then this is the gestational age at the episode of RPTL.

† Study population consisted exclusively of women with RPTL.

§ A third comparison arm (control group) was not extracted because this group did not have preterm labor.

** Study population consisted exclusively of women with twin gestation.

†† Study population consisted of women with single and twin gestation.

‡‡ Gestation not specified, although study population likely consisted of women with single and multiple gestation.

*** Gestational age at delivery was calculated by adding the variables gestational age at tocolytic cessation and interval to delivery. The associated standard deviations were calculated based on the reported standard deviations for interval to delivery (standard deviation of gestational age at tocolytic cessation was assumed to be 0 for both groups).

††† Gestation not specified, although study population likely included a mixture of women with single and multiple gestation.

‡‡‡ There were discrepancies in the information presented in the text and table of this paper. Mean gestational age at delivery for SQ terbutaline pump was reported as 36.6 weeks in table (as reported above) and 37.2 weeks in text.

Table F6. Studies that reported incidence of delivery at various gestational ages (Key Question 2)

Outcome	First Author (Year)	Study Design (n=Sample Size)	Mean Maternal Age (Years)	Mean GA (Weeks)*	Comparator(s)	Results—SQ Terbutaline Pump: % (n/N)	Results—Comparison: % (n/N)	Results—OR (95% CI)
Incidence of delivery < 28 weeks	N/A	N/A	N/A	N/A	N/A	N/A	N/A	N/A
Incidence of delivery < 32 weeks	Flick† (2010)[14]	Retrospective cohort (n=1366)	28.7 ± 6.1	30.6 ± 2.9 (P)	Oral nifedipine	2.6% (14/536)	8.4% (70/830)	0.29 (0.16, 0.52)
	de la Torre†,‡ (2008)[15]	Retrospective cohort (n=656)	30.3 ± 5.8	30.1 ± 2.9 (P)	Oral nifedipine	9.2% (44/476)	17.7% (148/836)	0.47 (0.33, 0.68)
	Fleming† (2004)[16]	Retrospective cohort (n=284)	NR	30.4 ± 2.6 (P)	Oral nifedipine	2.8% (4/142)	12.7% (18/142)	0.20 (0.07, 0.61)
	Lam† (2003)[17]	Retrospective cohort (n=558)	27.4 ± 5.9	31.6 ± 2.2 (P)	Oral tocolytics (95.3% received oral terbutaline)	2.5% (7/279)	10.8%(30/279)	0.21 (0.09, 0.50)
	Morrison† (2003)[12]	Prospective cohort (n=60)	25.6 ± 5.2	29.5 ± 2.3 (P)	No treatment	0% (0/15)	46.7% (21/45)	0.04 (0.00, 0.65)
	Lam†,‡ (2001)[18]	Retrospective cohort (n=706)	28.8 ± 5.5	31.3 ± 2.3 (P)	Oral tocolytics (92.3% received oral terbutaline)	6.2% (44/706)	11.3% (80/706)	0.52 (0.35, 0.76)
Incidence of delivery < 34 weeks	Guinn (1998)[9]	RCT (n=52)	21.6 ± 5.7	30.6 ± 2.8 (T)	Placebo	41.7% (10/24)	42.8% (12/28)	0.95 (0.32, 2.87)
Incidence of delivery < 37 weeks	Flick† (2010)[14]	Retrospective cohort (n=1366)	28.7 ± 6.1	30.6 ± 2.9 (P)	Oral nifedipine	51.3% (275/536)	59.3% (492/830)	0.72 (0.58, 0.90)
	Fleming† (2004)[16]	Retrospective cohort (n=284)	NR	30.4 ± 2.6 (P)	Oral nifedipine	52.1% (74/142)	59.2% (84/142)	0.75 (0.47, 1.20)
	Lam† (2003)[17]	Retrospective cohort (n=558)	27.4 ± 5.9	31.6 ± 2.2 (P)	Oral tocolytics (95.3% received oral terbutaline)	52.7% (147/279)	61.3% (171/279)	0.70 (0.50, 0.98)
	Morrison† (2003)[12]	Prospective cohort (n=60)	25.6 ± 5.2	29.5 ± 2.3 (P)	No treatment	46.7% (7/15)	95.6% (43/45)	0.04 (0.01, 0.23)
	Guinn (1998)[9]	RCT (n=52)	21.6 ± 5.7	30.6 ± 2.8 (T)	Placebo	70.8% (17/24)	60.7% (17/28)	1.57 (0.49, 5.02)
	Allbert†,§ (1994)[19]	Retrospective cohort (n=64)	27.5 ± 4.3	32.2 ± 2.7 (T)	Oral terbutaline	34.4% (11/32)	84.4% (27/32)	0.10 (0.03, 0.32)

CI = confidence interval; GA = gestational age; N/A = not applicable; NR = not reported; OR = odds ratio; RCT = randomized controlled trial; SQ = subcutaneous

Note: Subjects were women with singleton gestation only, unless indicated otherwise.

* Either at preterm labor (indicated by P) or at start of subcutaneous terbutaline therapy (indicated by T). If study population stated RPTL as an inclusion criterion, then this is the gestational age at the episode of RPTL.

† Study population consisted exclusively of women with RPTL.

‡ Study population consisted exclusively of women with twin gestation. Denominator is number of infants.

§ Gestation not specified, although population most likely included women with single and multiple gestation. Denominator is number of women.

Table F7. Studies that reported prolongation of pregnancy (Key Question 2)

Outcome	First Author (Year)	Study Design (n=Sample Size)	Mean Maternal Age (Years)	Mean GA (weeks)*	Comparator(s)	Results—SQ Terbutaline Pump: Mean ± SD or % (n/N)	Results—Comparison: Mean ± SD or % (n/N)	Results—Either Difference in Means (95% CI) or OR (95% CI)
Mean prolongation of pregnancy<br>*Results are reported as mean prolongation in days*	Flick† (2010)[14]	Retrospective cohort (n=1366)	28.7 ± 6.1	30.6 ± 2.9 (P)	Oral nifedipine	44.0 ± 23.0<br>*Measured from hospital admission for RPTL*	36.5 ± 24.0	7.50 (4.94, 10.06)
	de la Torre†,‡ (2008)[15]	Retrospective cohort (n=656)	30.3 ± 5.8	30.1 ± 2.9 (P)	Oral nifedipine	34.7 ± 18.8<br>*Measured from episode of RPTL*	27.5 ± 19.9	7.20 (4.10, 10.30)
	Fleming† (2004)[16]	Retrospective cohort (n=284)	NR	30.4 ± 2.6 (P)	Oral nifedipine	43.3 ± 21.6<br>*Measured from episode of RPTL*	37.1 ± 24.8	6.20 (0.79, 11.61)
	Lam† (2003)[17]	Retrospective cohort (n=558)	27.4 ± 5.9	31.6 ± 2.2 (P)	Oral tocolytics (95.3% received oral terbutaline)	33.9 ± 19.0<br>*Measurement interval not specified*	28.4 ± 19.8	5.50 (2.28, 8.72)
	Morrison† (2003)[12]	Prospective cohort (n=60)	25.6 ± 5.2	29.5 ± 2.3 (P)	No treatment	49.8 ± 19.2<br>*Measured from episode of RPTL*	24.5 ± 12.8	25.30 (16.77, 33.83)
	Guinn (1998)[9]	RCT (n=52)	21.6 ± 5.7	30.6 ± 2.8 (T)	Placebo	28.8 ± 22.0<br>*Measured from random assignment*	27.9 ± 22.9	0.90 (-11.36, 13.16)
	Wenstrom§ (1997)[10]	RCT (n=42)	26.2 ± 5.3	30.4 ± 2.3 (T)	Placebo (C_1)<br>Oral terbutaline (C_2)	35.0 ± 28.7<br>*Measurement interval not specified*	C_1: 35.0 ± 17.5<br>C_2: 29.4 ± 27.3	0.00 (-18.53, 18.53)<br>5.60 (-14.45, 25.65)
Pregnancy prolongation > 7 days	Flick† (2010)[14]	Retrospective cohort (n=1366)	28.7 ± 6.1	30.6 ± 2.9 (P)	Oral nifedipine	98.7% (529/536)<br>*Measured from hospital admission for RPTL*	90.6% (752/830)	7.84 (3.59, 17.12)
	Fleming† (2004)[16]	Retrospective cohort (n=284)	NR	30.4 ± 2.6 (P)	Oral nifedipine	96.5% (137/142)<br>*Measured from episode of RPTL*	91.5% (130/142)	2.53 (0.87, 7.38)

Table F7. Studies that reported prolongation of pregnancy (Key Question 2) (continued)

Outcome	First Author (Year)	Study Design (n=Sample Size)	Mean Maternal Age (Years)	Mean GA (Weeks)*	Comparator(s)	Results—SQ Terbutaline Pump: Mean ± SD or % (n/N)	Results—Comparison: Mean ± SD or % (n/N)	Results—Either Difference in Means (95% CI) or OR (95% CI)
Pregnancy prolongation > 14 days	Flick[†] (2010)[14]	Retrospective cohort (n=1366)	28.7 ± 6.1	30.6 ± 2.9 (P)	Oral nifedipine	93.8% (503/536) *Measured from hospital admission for RPTL*	81.4% (676/830)	3.47 (2.34, 5.15)
	de la Torre[†,‡] (2008)[15]	Retrospective cohort (n=656)	30.3 ± 5.8	30.1 ± 2.9 (P)	Oral nifedipine	84.4% (201/238) *Measured from episode of RPTL*	68.7% (287/418)	2.48 (1.65, 3.73)
	Fleming[†] (2004)[16]	Retrospective cohort (284)	NR	30.4 ± 2.6 (P)	Oral nifedipine	93.0% (132/142) *Measured from episode of RPTL*	82.4% (117/142)	2.82 (1.30, 6.12)
	Lam[†] (2003)[17]	Retrospective cohort (n=558)	27.4 ± 5.9	31.6 ± 2.2 (P)	Oral tocolytics (95.3% received oral terbutaline)	85.7% (239/279) *Measurement interval not specified*	71.3% (199/279)	2.40 (1.57, 3.67)
	Lam[†,‡] (2001)[18]	Retrospective cohort (n=706)	28.8 ± 5.5	31.3 ± 2.3 (P)	Oral tocolytics (92.3% received oral terbutaline)	73.6% (260/353) *Measured from episode of RPTL*	59.2% (209/353)**	1.93 (1.40, 2.65)

CI = confidence interval; GA = gestational age; N/A = not applicable; NR = not reported; OR = odds ratio; RCT = randomized controlled trial; RPTL = recurrent preterm labor; SD = standard deviation; SQ = subcutaneous

Note: Subjects were women with singleton gestation only, unless indicated otherwise.

* Either at preterm labor (indicated by P) or at start of subcutaneous terbutaline therapy (indicated by T). If study population stated RPTL as an inclusion criterion, then this is the gestational age at the episode of RPTL.

† Study population consisted exclusively of women with RPTL.

‡ Study population consisted exclusively of women with twin gestation.

§ Study population consisted of women with single and twin gestation.

** Additional reported data: SQ terbutaline pump group gained an average of 4.5 gestational days (95% CI: 2.3–6.8) compared with oral tocolytic group.[18]

Table F8. Studies that reported birth weight (Key Question 2)

Outcome	First Author (Year)	Study Design (n=Sample Size)	Mean Maternal Age (Years)	Mean GA (Weeks)[*]	Comparator(s)	Results—SQ Terbutaline Pump: Mean ± SD or % (n/N)	Results—Comparison: Mean ± SD or % (n/N)	Results—Either Difference in Means (95% CI) or OR (95% CI)
Mean birth weight<br><br>*Results are reported as mean birth weight in grams*	de la Torre[†,‡] (2008)[15]	Retrospective cohort (n=656)	30.3 ± 5.8	30.1 ± 2.9 (P)	Oral nifedipine	2252 ± 501	2089 ± 564	163 (102, 224)
	Fleming[†] (2004)[16]	Retrospective cohort (n=284)	NR	30.4 ± 2.6 (P)	Oral nifedipine	2900 ± 568	2638 ± 784	262 (103, 421)
	Lam[†] (2003)[17]	Retrospective cohort (n=558)	27.4 ± 5.9	31.6 ± 2.2 (P)	Oral tocolytics (95.3% received oral terbutaline)	2941 ± 556	2676 ± 667	265 (163, 367)
	Morrison[†] (2003)[12]	Prospective cohort (n=60)	25.6 ± 5.2	29.5 ± 2.3 (P)	No treatment	2700 ± 464	1979 ± 670	721 (355, 1087)
	Lam[†,‡] (2001)[18]	Retrospective cohort (n=706)	28.8 ± 5.5	31.3 ± 2.3 (P)	Oral tocolytics (92.3% received oral terbutaline)	2343 ± 493	2207 ± 523	136 (83, 189)
	Guinn (1998)[9]	RCT (n=52)	21.6 ± 5.7	30.6 ± 2.8 (T)	Placebo	2349 ± 770	2324 ± 768	25 (-394, 444)
	Wenstrom[§] (1997)[10]	RCT (n=42)	26.2 ± 5.3	30.4 ± 2.3 (T)	Placebo (C_1) Oral terbutaline (C_2)	2688 ± 599	C_1: 2457 ± 727<br>C_2: 2204 ± 808	231 (-214, 676)<br>484 (17, 951)
	Allbert[†,**] (1994)[19]	Retrospective cohort (n=64)	27.5 ± 4.3	32.2 ± 2.7 (T)	Oral terbutaline	2853 ± 702	2682 ± 528	171 (-133, 475)
	Regenstein[††] (1993)[20]	Retrospective cohort (n=69)	31.4 ± 5.9	NR	Oral terbutaline[‡‡]	2558 ± 838	3262 ± 567	-704 (-1037, -371)
	Lindenbaum (1992)[11]	Nonrandomized comparative trial (n=91)	32.4 ± 2.7	29.1 ± 1.7 (T)	Oral terbutaline[‡‡]	3017 ± 303	3229 ± 584	-212 (-417, -7)[§§]
Incidence of low birth weight (< 2,500 g)	Flick[†] (2010)[14]	Retrospective cohort (n=1366)	28.7 ± 6.1	30.6 ± 2.9 (P)	Oral nifedipine	20.3% (109/536)	32.9% (273/830)	0.52 (0.40, 0.67)
	de la Torre[†,‡] (2008)[15]	Retrospective cohort (n=656)	30.3 ± 5.8	30.1 ± 2.9 (P)	Oral nifedipine	67.2% (320/476)	78.3% (655/836)	0.57 (0.44, 0.73)
	Fleming[†] (2004)[16]	Retrospective cohort (n=284)	NR	30.4 ± 2.6 (P)	Oral nifedipine	23.2% (33/142)	43.0% (61/142)	0.40 (0.24, 0.67)
	Lam[†] (2003)[17]	Retrospective cohort (n=558)	27.4 ± 5.9	31.6 ± 2.2 (P)	Oral tocolytics (95.3% received oral terbutaline)	20.8% (58/279)	38.0% (106/279)	0.43 (0.29, 0.62)
	Morrison[†] (2003)[12]	Prospective cohort (n=60)	25.6 ± 5.2	29.5 ± 2.3 (P)	No treatment	20.0% (3/15)	51.1% (23/45)	0.24 (0.06, 0.96)
	Lam[†,‡] (2001)[18]	Retrospective cohort (n=706)	28.8 ± 5.5	31.3 ± 2.3 (P)	Oral tocolytics (92.3% received oral terbutaline)	61.5% (432/702)	71.5% (494/691)	0.64 (0.51, 0.80)

Table F8. Studies that reported birth weight (Key Question 2) (continued)

Outcome	First Author (Year)	Study Design (n=Sample Size)	Mean Maternal Age (Years)	Mean GA (Weeks)*	Comparator(s)	Results—SQ Terbutaline Pump: Mean ± SD or % (n/N)	Results—Comparison: Mean ± SD or % (n/N)	Results—Either Difference in Means (95% CI) or OR (95% CI)
Incidence of very low birth weight (< 1,500 g)	de la Torre†,‡ (2008)[15]	Retrospective cohort (n=656)	30.3 ± 5.8	30.1 ± 2.9 (P)	Oral nifedipine	6.5% (31/476)	15.0% (125/836)	0.40 (0.26, 0.60)
	Fleming† (2004)[16]	Retrospective cohort (n=284)	NR	30.4 ± 2.6 (P)	Oral nifedipine	2.1% (3/142)	7.0% (10/142)	0.28 (0.08, 1.06)
	Lam† (2003)[17]	Retrospective cohort (n=558)	27.4 ± 5.9	31.6 ± 2.2 (P)	Oral tocolytics (95.3% received oral terbutaline)	1.4% (4/279)	6.1% (17/279)	0.22 (0.07, 0.67)
	Lam†,‡ (2001)[18]	Retrospective cohort (706)	28.8 ± 5.5	31.3 ± 2.3 (P)	Oral tocolytics (92.3% received oral terbutaline)	4.1% (29/702)	8.5% (59/691)	0.46 (0.29, 0.73)
Ratio of birth weight/GA at delivery	N/A	N/A	N/A	N/A	N/A	N/A	N/A	N/A

CI = confidence interval; GA = gestational age; NR = not reported; OR = odds ratio; RCT = randomized controlled trial; SD = standard deviation; SQ = subcutaneous

Note: Subjects were women with singleton gestation only, unless indicated otherwise.

* Either at preterm labor (indicated by P) or at start of subcutaneous terbutaline therapy (indicated by T). If study population stated RPTL as an inclusion criterion, then this is the gestational age at the episode of RPTL.

† Study population consisted exclusively of women with RPTL.

‡ Study population consisted exclusively of women with twin gestation. Denominator is number of infants.

§ Study population consisted of women with single and twin gestation.

** Gestation not specified, although population most likely included women with single and multiple gestation.

†† Gestation not specified, although study population likely consisted of women with single and multiple gestation. Reported mean birthweight is for singletons only.

‡‡ A second comparison group, consisting of women without preterm labor, was not extracted.

§§ There were discrepancies in the information presented in the text and table of this paper. The table reported the numbers as indicated above. However, the text reported groups with the reverse numbers (i.e. SQ terbutaline pump: 3229 ± 584 and oral terbutaline: 3017 ± 303).

Table F9. Studies that reported other outcomes (Key Question 2)

Outcome	First Author (Year)	Study Design (n=Sample Size)	Mean Maternal Age (Years)	Mean GA (Weeks)[*]	Comparator(s)	Results—SQ Terbutaline Pump: Mean ± SD or % (n/N)	Results—Comparison: Mean ± SD or % (n/N)	Results—Either Difference in Means (95% CI) or OR (95% CI)
Mean PPI	Morrison[†] (2003)[12]	Prospective cohort (n=60)	25.6 ± 5.2	29.5 ± 2.3 (P)	No treatment	0.92 ± 0.19	0.51 ± 0.28	0.41 (0.26, 0.56)
	Allbert[†,**] (1994)[19]	Retrospective cohort (64)	27.5 ± 4.3	32.2 ± 2.7 (T)	Oral terbutaline	0.86 ± 0.25	0.72 ± 0.25	0.14 (0.02, 0.26)
Need for assisted ventilation	Lam[†] (2003)[17]	Retrospective cohort (n=558)	27.4 ± 5.9	31.6 ± 2.2 (P)	Oral tocolytics (95.3% received oral terbutaline)	24.4% (68/279)<br>*Only assessed for those with NICU admission*	26.2% (73/279)	0.91 (0.62, 1.33)
Need for oxygen per nasal cannula	N/A	N/A	N/A	N/A	N/A	N/A	N/A	N/A
NICU admission incidence	Flick[†] (2010)[14]	Retrospective cohort (n=1366)	28.7 ± 6.1	30.6 ± 2.9 (P)	Oral nifedipine	23.1% (124/536)	34.0% (282/830)	0.58 (0.46, 0.75)
	de la Torre[†,‡] (2008)[15]	Retrospective cohort (n=656)	30.3 ± 5.8	30.1 ± 2.9 (P)	Oral nifedipine	44.7% (213/476)	52.9% (442/836)	0.72 (0.58, 0.91)
	Fleming[†] (2004)[16]	Retrospective cohort (n=284)	NR	30.4 ± 2.6 (P)	Oral nifedipine	23.2% (33/142)	43.7% (62/142)	0.39 (0.23, 0.65)
	Lam[†] (2003)[17]	Retrospective cohort (n=558)	27.4 ± 5.9	31.6 ± 2.2 (P)	Oral tocolytics (95.3% received oral terbutaline)	18.6% (52/279)<br>*NICU (Level III) admission*	26.2% (73/279)	0.65 (0.43, 0.97)
	Morrison[†] (2003)[12]	Prospective cohort (n=60)	25.6 ± 5.2	29.5 ± 2.3 (P)	No treatment	33.3% (5/15)	64.4% (29/45)	0.28 (0.08, 0.95)
	Lam[†,‡] (2001)[18]	Retrospective cohort (n=706)	28.8 ± 5.5	31.3 ± 2.3 (P)	Oral tocolytics (92.3% received oral terbutaline)	38.5% (270/702)	55.0% (380/691)	0.51 (0.41, 0.63)
	Guinn (1998)[9]	RCT (n=52)	21.6 ± 5.7	30.6 ± 2.8 (T)	Placebo	43.5% (10/23)<br>*Neonates remaining in NICU > 24 hours*	46.4% (13/28)	0.89 (0.29, 2.69)

Table F9. Studies that reported other outcomes (Key Question 2) (continued)

Outcome	First Author (Year)	Study Design (n=Sample Size)	Mean Maternal Age (Years)	Mean GA (Weeks)*	Comparator(s)	Results—SQ Terbutaline Pump: Mean ± SD or % (n/N)	Results—Comparison: Mean ± SD or % (n/N)	Results—Either Difference in Means (95% CI) or OR (95% CI)
NICU mean length of stay<br>*Results are reported as mean length of stay in days*	Flick† (2010)[14]	Retrospective cohort (n=1366)	28.7 ± 6.1	30.6 ± 2.9 (P)	Oral nifedipine	2.8 ± 9.2	6.5 ± 17.2	-3.70 (-5.29, -2.11)
	Lam† (2003)[17]	Retrospective cohort (n=558)	27.4 ± 5.9	31.6 ± 2.2 (P)	Oral tocolytics(95.3% received oral terbutaline)	14.1 ± 17.7<br>*NICU (Level III) admission*	21.0 ± 22.5	-6.90 (-10.26, -3.54)
	Morrison† (2003)[12]	Prospective cohort (n=60)	25.6 ± 5.2	29.5 ± 2.3 (P)	No treatment	1.9 ± 4.9	19.8 ± 29.3	-17.90 (-32.88, -2.92)
	Lam†,‡ (2001)[18]	Retrospective cohort (n=706)	28.8 ± 5.5	31.3 ± 2.3 (P)	Oral tocolytics (92.3% received oral terbutaline)	17.3 ± 16.1	20.8 ± 17.4	-3.50 (-5.26, -1.74)
	Wenstrom§ (1997)[10]	RCT (n=42)	26.2 ± 5.3	30.4 ± 2.3 (T)	Placebo (C_1) Oral terbutaline (C_2)	10.9 ± 19.4	C_1: 15.0 ± 18.8<br>C_2: 15.4 ± 17.0	-4.10 (-17.06, 8.86)<br>-4.50 (-16.70, 7.70)

CI = confidence interval; GA = gestational age; N/A = not applicable; NICU = neonatal intensive care unit; NR = not reported; OR = odds ratio; PPI = pregnancy prolongation index; RCT = randomized controlled trial; SD = standard deviation; SQ = subcutaneous

Note: Subjects were women with singleton gestation only, unless indicated otherwise.

* Either at preterm labor (indicated by P) or at start of subcutaneous terbutaline therapy (indicated by T). If study population stated RPTL as an inclusion criterion, then this is the gestational age at the episode of RPTL.

† Study population consisted exclusively of women with RPTL.

‡ Study population consisted exclusively of women with twin gestation. Denominator is number of infants.

§ Study population consisted of women with single and twin gestation.

** Gestation not specified, although population most likely included women with single and multiple gestation.

Table F10. Studies that reported maternal harms (Key Question 3)

Outcome	First Author (Year)	Study Design (n=Sample Size)	Mean Maternal Age (Years)	Mean GA (Weeks)[*]	Comparator(s)	Results—SQ Terbutaline Pump: % (n/N)	Results—Comparison: % (n/N)	Results—OR (95% CI)
Arrhythmia	Morrison[†] (2003)[12]	Prospective cohort (n=60)	25.6 ± 5.2	29.5 ± 2.3 (P)	No treatment	20.0% (3/15)<br>*Defined as tachycardia, nervousness*	0% (0/45)	25.48 (1.23, 526.64)
Heart failure	N/A	N/A	N/A	N/A	N/A	N/A	N/A	N/A
Hyperglycemia<br>*Reported results indicate women with gestational diabetes, based on 3-hour GTT.*	Regenstein[‡] (1993)[20]	Retrospective cohort (n=69)	31.4 ± 5.9	NR	Oral terbutaline§	20.0% (6/30)	11.4% (4/35)	1.94 (0.49, 7.65)
	Lindenbaum (1992)[11]	Nonrandomized comparative trial (n=91)	32.4 ± 2.7	29.1 ± 1.7 (T)	Oral terbutaline[§]	5.4% (2/37)	11.1% (6/54)	0.46 (0.09, 2.40)
Hypokalemia	N/A	N/A	N/A	N/A	N/A	N/A	N/A	N/A
Mortality	Lam[†] (2003)[17]	Retrospective cohort (n=558)	27.4 ± 5.9	31.6 ± 2.2 (P)	Oral tocolytics (95.3% received oral terbutaline)	0% (0/279)	0% (0/279)	1 (0.02, 50.58)
	Lam[†,**] (2001)[18]	Retrospective cohort (n=706)	28.8 ± 5.5	31.3 ± 2.3 (P)	Oral tocolytics (92.3% received oral terbutaline)	0% (0/353)	0% (0/353)	1 (0.02, 50.54)
Myocardial infarction	N/A	N/A	N/A	N/A	N/A	N/A	N/A	N/A
Pulmonary edema	Lam[†] (2003)[17]	Retrospective cohort (n=558)	27.4 ± 5.9	31.6 ± 2.2 (P)	Oral tocolytics (95.3% received oral terbutaline)	0% (0/279)	0.4% (1/279)	0.33 (0.01, 8.19)
	Lam[†,**] (2001)[18]	Retrospective cohort (n=706)	28.8 ± 5.5	31.3 ± 2.3 (P)	Oral tocolytics (92.3% received oral terbutaline)	0.3% (1/353)	0% (0/353)	3.01 (0.12, 74.11)
Refractory hypotension	N/A	N/A	N/A	N/A	N/A	N/A	N/A	N/A

Table F10. Studies that reported maternal harms (Key Question 3) (continued)

Outcome	First Author (Year)	Study Design (n=Sample Size)	Mean Maternal Age (Years)	Mean GA (Weeks)*	Comparator(s)	Results—SQ Terbutaline Pump: % (n/N)	Results—Comparison: % (n/N)	Results—OR (95% CI)
Therapy discontinuation	Morrison† (2003)[12]	Prospective cohort (n=60)	25.6 ± 5.2	29.5 ± 2.3 (P)	No treatment	0% (0/15)	N/A	N/A
	Guinn (1998)[9]	RCT (n=52)	21.6 ± 5.7	30.6 ± 2.8 (T)	Placebo	45.8% (11/24)	32.1% (9/28)	1.79 (0.58, 5.52)
Withdrawal-AE	N/A	N/A			N/A	N/A	N/A	N/A

CI = confidence interval; GA = gestational age; GTT = glucose tolerance test; N/A = not applicable; NR = not reported; OR = odds ratio; RCT = randomized controlled trial; SQ = subcutaneous; Withdrawal-AE = withdrawal due to adverse effects

Note: Subjects were women with singleton gestation only, unless indicated otherwise.

* Either at preterm labor (indicated by P) or at start of subcutaneous terbutaline therapy (indicated by T). If study population stated RPTL as an inclusion criterion, then this is the gestational age at the episode of RPTL.

† Study population consisted exclusively of women with RPTL.

‡ Gestation not specified, although study population likely consisted of women with single and multiple gestation.

§ Data for a second comparison group, which consisted of women without preterm labor, was not extracted.

** Study population consisted exclusively of women with twin gestation.

Table F11. Studies that reported neonatal harms (Key Question 4)

Outcome	First Author (Year)	Study Design (n=Sample Size)	Mean Maternal Age (Years)	Mean GA (Weeks)[*]	Comparator(s)	Results—SQ Terbutaline Pump: % (n/N)	Results—Comparison: % (n/N)	Results—OR (95% CI)
Hypoglycemia	Wenstrom[†] (1997)[10]	RCT (n=42)	26.2 ± 5.3	30.4 ± 2.3 (T)	Placebo (C_1)<br>Oral terbutaline (C_2)	0% (0/19)	C_1: 6.7% (1/15)<br>C_2: 0% (0/16)	0.25 (0.01, 6.53)<br>0.85 (0.02, 45.03)
Hypocalcemia	N/A	N/A	N/A	N/A	N/A	N/A	N/A	N/A
Ileus	N/A	N/A	N/A	N/A	N/A	N/A	N/A	N/A

CI = confidence interval; GA = gestational age; N/A = not applicable; OR = odds ratio; RCT = randomized controlled trial; SQ = subcutaneous

[*] Either at preterm labor (indicated by P) or at start of subcutaneous terbutaline therapy (indicated by T). If study population stated RPTL as an inclusion criterion, then this is the gestational age at the episode of RPTL.

[†] Study population consisted of women with single and twin gestation. Denominator is number of infants.

Table F12. Criteria for rating level of maternal activity variables

Criteria	Low Activity Level	Normal Activity Level	High Activity Level
**Marital Status**	Married or living common-law with partner not working outside the home	Married or living common-law with partner working outside the home	Single, divorced, widowed, or separated
**Working Status**	Not working	Occasional or part-time work	Full-time work
**Caring for Other Children in the Home[1]**	No other children in the home	One other child in the home	More than one other child in the home
**Available Social Support**	Women report substantial support from friends and family	Women report limited support to be available from friends and family	No support available to women from friends and family
**Bed Rest**	Complete bed rest with bathroom privileges only	Bed rest suggested when an increase in uterine contractions only	Bed rest not recommended
**Restriction of Maternal Activities**	Maternal activities, such as household chores and intercourse, recommended to be completely restricted	Restriction of activities suggested when an increase in uterine contractions only	No restriction of maternal activities recommended

Table F13. Risk of bias ratings for level of maternal activity

First Author (year)	Marital Status	Working Status	Caring for Other Children	Social Support	Bed Rest	Restriction of Maternal Activities	Overall Rating
RCTs							
Guinn (1998)[9]	Not reported<br>INSUFFICIENT DATA TO MAKE RATING	Not reported<br>INSUFFICIENT DATA TO MAKE RATING	Multiparity: 57% of placebo group and 63% of terbutaline group<br>INSUFFICIENT DATA TO MAKE RATING	Not reported<br>INSUFFICIENT DATA TO MAKE RATING	Not reported<br>INSUFFICIENT DATA TO MAKE RATING	Not reported<br>INSUFFICIENT DATA TO MAKE RATING	UNCLEAR
Wenstrom (1997)[10]	Not reported<br>INSUFFICIENT DATA TO MAKE RATING	Not reported<br>INSUFFICIENT DATA TO MAKE RATING	Median parity provided for all groups<br>INSUFFICIENT DATA TO MAKE RATING	Not reported<br>INSUFFICIENT DATA TO MAKE RATING	Patients were instructed to remain at bed rest<br>LOW	Not reported<br>INSUFFICIENT DATA TO MAKE RATING	LOW<br>*Based on bed rest*
NONRANDOMIZED TRIALS							
Lindenbaum (1992)[11]	Not reported<br>INSUFFICIENT DATA TO MAKE RATING	Not reported<br>INSUFFICIENT DATA TO MAKE RATING	Not reported<br>INSUFFICIENT DATA TO MAKE RATING	Not reported<br>INSUFFICIENT DATA TO MAKE RATING	Not reported<br>INSUFFICIENT DATA TO MAKE RATING	Not reported<br>INSUFFICIENT DATA TO MAKE RATING	UNCLEAR
PROSPECTIVE COHORTS							
Morrison (2003)[12]	Not reported<br>INSUFFICIENT DATA TO MAKE RATING	Not reported<br>INSUFFICIENT DATA TO MAKE RATING	Not reported<br>INSUFFICIENT DATA TO MAKE RATING	Not reported<br>INSUFFICIENT DATA TO MAKE RATING	Bed rest advised<br>LOW	Interdiction of intercourse advised<br>LOW	LOW
Morrison (1992)[13]	Not reported<br>INSUFFICIENT DATA TO MAKE RATING	Not reported<br>INSUFFICIENT DATA TO MAKE RATING	Parity reported<br>INSUFFICIENT DATA TO MAKE RATING	Not reported<br>INSUFFICIENT DATA TO MAKE RATING	Not reported<br>INSUFFICIENT DATA TO MAKE RATING	Not reported<br>INSUFFICIENT DATA TO MAKE RATING	UNCLEAR

Table F13. Risk of bias ratings for level of maternal activity (continued)

First Author (year)	Marital Status	Working Status	Caring for Other Children	Social Support	Bed Rest	Restriction of Maternal Activities	Overall Rating
RETROSPECTIVE COHORTS							
Flick (2010)[14]	Married: 71.8% in nifedipine group and 85.3% in terbutaline group<br>**INSUFFICIENT DATA TO MAKE RATING**	Not reported<br>**INSUFFICIENT DATA TO MAKE RATING**	Not reported<br>**INSUFFICIENT DATA TO MAKE RATING**	Not reported<br>**INSUFFICIENT DATA TO MAKE RATING**	Not reported<br>**INSUFFICIENT DATA TO MAKE RATING**	Not reported<br>**INSUFFICIENT DATA TO MAKE RATING**	**UNCLEAR**
de la Torre (2008)[15]	Married: 80.9% in nifedipine group and 87.8% in SQ terbutaline group<br>**INSUFFICIENT DATA TO MAKE RATING**	Not reported<br>**INSUFFICIENT DATA TO MAKE RATING**	Nulliparous: 56% in nifedipine group and 58.8% in SQ terbutaline group<br>**INSUFFICIENT DATA TO MAKE RATING**	Not reported<br>**INSUFFICIENT DATA TO MAKE RATING**	Not reported<br>**INSUFFICIENT DATA TO MAKE RATING**	Not reported<br>**INSUFFICIENT DATA TO MAKE RATING**	**UNCLEAR**
Fleming (2004)[16]	Married: 71.8% in nifedipine group and 85.2% in SQ terbutaline group<br>**INSUFFICIENT DATA TO MAKE RATING**	Not reported<br>**INSUFFICIENT DATA TO MAKE RATING**	Nulliparous: 43% in nifedipine group and 40.8% in SQ terbutaline group<br>**INSUFFICIENT DATA TO MAKE RATING**	Not reported<br>**INSUFFICIENT DATA TO MAKE RATING**	Not reported<br>**INSUFFICIENT DATA TO MAKE RATING**	Not reported<br>**INSUFFICIENT DATA TO MAKE RATING**	**UNCLEAR**
Lam (2003)[17]	Married: 69.2% in oral tocolytic group and 84.2% in SQ terbutaline group<br>**INSUFFICIENT DATA TO MAKE RATING**	Not reported<br>**INSUFFICIENT DATA TO MAKE RATING**	Not reported<br>**INSUFFICIENT DATA TO MAKE RATING**	Not reported<br>**INSUFFICIENT DATA TO MAKE RATING**	Not reported<br>**INSUFFICIENT DATA TO MAKE RATING**	Not reported<br>**INSUFFICIENT DATA TO MAKE RATING**	**UNCLEAR**

Table F13. Risk of bias ratings for level of maternal activity (continued)

First Author (year)	Marital Status	Working Status	Caring for Other Children	Social Support	Bed Rest	Restriction of Maternal Activities	Overall Rating
Lam (2001)[18]	Married: 77.3% in oral tocolytic group and 87.8% in SQ terbutaline group<br>INSUFFICIENT DATA TO MAKE RATING	Not reported<br>INSUFFICIENT DATA TO MAKE RATING	Not reported<br>INSUFFICIENT DATA TO MAKE RATING	Not reported<br>INSUFFICIENT DATA TO MAKE RATING	Not reported<br>INSUFFICIENT DATA TO MAKE RATING	Not reported<br>INSUFFICIENT DATA TO MAKE RATING	UNCLEAR
Allbert (1994)[19]	Not reported<br>INSUFFICIENT DATA TO MAKE RATING	Not reported<br>INSUFFICIENT DATA TO MAKE RATING	Parity provided for oral terbutaline and SQ terbutaline groups<br>INSUFFICIENT DATA TO MAKE RATING	Not reported<br>INSUFFICIENT DATA TO MAKE RATING	Bed rest advised<br>LOW	Prohibition of intercourse advised<br>LOW	LOW
Regenstein (1993)[20]	Not reported<br>INSUFFICIENT DATA TO MAKE RATING	Not reported<br>INSUFFICIENT DATA TO MAKE RATING	Parity reported<br>INSUFFICIENT DATA TO MAKE RATING	Not reported<br>INSUFFICIENT DATA TO MAKE RATING	Not reported<br>INSUFFICIENT DATA TO MAKE RATING	Not reported<br>INSUFFICIENT DATA TO MAKE RATING	UNCLEAR

Table F13. Risk of bias ratings for level of maternal activity (continued)

First Author (year)	Marital Status	Working Status	Caring for Other Children	Social Support	Bed Rest	Restriction of Maternal Activities	Overall Rating
CASE SERIES							
Adkins (1993)[21]	Not reported<br>**INSUFFICIENT DATA TO MAKE RATING**	Not reported<br>**INSUFFICIENT DATA TO MAKE RATING**	Not reported<br>**INSUFFICIENT DATA TO MAKE RATING**	Not reported<br>**INSUFFICIENT DATA TO MAKE RATING**	If contractions were detected, patients were instructed to void, hydrate, remain at bed rest.<br>**LOW**	Not reported<br>**INSUFFICIENT DATA TO MAKE RATING**	**LOW**<br>***Based on bed rest***
Lam (1988)[22]	Not reported<br>**INSUFFICIENT DATA TO MAKE RATING**	Not reported<br>**INSUFFICIENT DATA TO MAKE RATING**	Not reported<br>**INSUFFICIENT DATA TO MAKE RATING**	Not reported<br>**INSUFFICIENT DATA TO MAKE RATING**	Patients were instructed to remain in bed, but were permitted bathroom privileges<br>**LOW**	Not reported<br>**INSUFFICIENT DATA TO MAKE RATING**	**LOW**<br>***Based on bed rest***

RCT = randomized controlled trial; SQ = subcutaneous

Table F14. Criteria for rating level of maternal care variables

Criteria	Low Level of Care	Moderate Level of Care	High Level of Care
**Nursing Assessments**	No nursing assessment made	Patient questioned regarding any barriers to compliance with prescribed therapy	An in-person assessment at the patient's home to identify barriers to successful compliance of prescribed therapy
**Home Uterine Activity Monitoring**	No home uterine activity monitoring recommended	Home uterine activity monitoring recommended, with or without a monitor	Home uterine activity monitored and data sent via telephone or computer to a central care centre to be assessed by a trained health professional
**Home Visits**	No home visits provided	At least one home visit provided	Regular (e.g., weekly) home visits provided
**Education About Preterm Labor**	No education was provided Written or oral education on the signs and symptoms of preterm labor, possible adverse reactions to treatment, etc.	Written and oral education provided on signs and symptoms of preterm labor, possible adverse reactions to treatment, etc.	Written and oral education provided on signs and symptoms of preterm labor, possible adverse reactions to treatment, etc. and education was individualized.
**Telephone Support**	No telephone support available	Telephone support available during select hours of the day only	Telephone support available 24 hours a day, 7 days a week by trained health professionals
**Restriction of Maternal Activities**	No suggestions made by a health professional regarding restriction of maternal activities	Global recommendations for restriction of maternal activities made for all women	Individualized suggestions for restriction of maternal activities made based on each patient's condition

Table F15. Ratings for level of maternal care

First Author (Year)	Nursing Assessments	Home Uterine Activity Monitoring	Home Visits	Education About Preterm Labor	Telephone Support	Restriction of Maternal Activities	Other Cointerventions	Overall Rating
RCTs								
Guinn (1998)[9]	Not reported<br>**INSUFFICIENT DATA TO MAKE RATING**	Not reported<br>**INSUFFICIENT DATA TO MAKE RATING**	Not reported<br>**INSUFFICIENT DATA TO MAKE RATING**	Educated about signs and symptoms of preterm labor "The women were also educated about early signs and symptoms of preterm labor"<br>**LOW**	Nursing support available 24 hours/day to answer questions and monitor therapy.<br>**HIGH**	Not reported<br>**INSUFFICIENT DATA TO MAKE RATING**	Outpatients were followed up on a weekly basis until 36 weeks' gestation.<br>**MODERATE**	**MODERATE**
Wenstrom (1997)[10]	Not reported<br>**INSUFFICIENT DATA TO MAKE RATING**	Not reported<br>**INSUFFICIENT DATA TO MAKE RATING**	Not reported<br>**INSUFFICIENT DATA TO MAKE RATING**	Not reported<br>**INSUFFICIENT DATA TO MAKE RATING**	Not reported<br>**INSUFFICIENT DATA TO MAKE RATING**	Patients were instructed to remain at bed rest<br>**MODERATE**	Patients seen in outpatient clinic weekly or biweekly<br>**MODERATE**	**UNCLEAR**
NONRANDOMIZED TRIALS								
Lindenbaum (1992)[11]	Not reported<br>**INSUFFICIENT DATA TO MAKE RATING**	Not reported<br>**INSUFFICIENT DATA TO MAKE RATING**	Not reported<br>**INSUFFICIENT DATA TO MAKE RATING**	Not reported<br>**INSUFFICIENT DATA TO MAKE RATING**	Not reported<br>**INSUFFICIENT DATA TO MAKE RATING**	Not reported<br>**INSUFFICIENT DATA TO MAKE RATING**	Not reported<br>**INSUFFICIENT DATA TO MAKE RATING**	**UNCLEAR**

Table F15. Ratings for level of maternal care (continued)

First Author (Year)	Nursing Assessments	Home Uterine Activity Monitoring	Home Visits	Education About Preterm Labor	Telephone Support	Restriction of Maternal Activities	Other Cointerventions	Overall Rating
PROSPECTIVE COHORTS								
Morrison (2003)[12]	**Verbal information received from John Morrison (March 24/11)** A daily telephone call was made by a perinatal nurse. Subjects were questioned about the signs and symptoms of preterm labor (e.g., contraction, tightening, cervical changes) and were also asked open-ended questions.<br>**MODERATE**	Patients in terbutaline group received a uterine contraction monitor and were instructed to monitor twice daily. A daily telephone call by a perinatal nurse was done to gather this information.<br>**RATING CANNOT BE MADE DUE TO CONFOUNDING**	**Verbal information received from John Morrison (March 24/11)** At least one home visit was made by a nurse to set up the SQ terbutaline pump.<br>**MODERATE**	Educated about the signs and symptoms of preterm labor "Women in the study and control groups were taught the signs and symptoms associated with preterm labor."<br>**Verbal information received from John Morrison (March 24/11)** Education also provided during daily calls by nurse.<br>**LOW**	Patients were given a 24-hour hotline number to call if they had any questions.<br>**HIGH**	Bed rest and interdiction of intercourse advised.<br>**MODERATE**	Patients were followed up in a preterm birth prevention clinic.<br>**Verbal information received from John Morrison (March 24/11)** Assessed for signs and symptoms of preterm labour and provided education: this was repeated every 1–2 weeks. During this time patients were also questioned further, education was reconfirmed, and more tests may have been performed.<br>**MODERATE**	**PUMP GROUP: HIGH CONTROL: MODERATE**

Table F15. Ratings for level of maternal care (continued)

First Author (Year)	Nursing Assessments	Home Uterine Activity Monitoring	Home Visits	Education About Preterm Labor	Telephone Support	Restriction of Maternal Activities	Other Cointerventions	Overall Rating
Morrison (1992)[13]	Intensive perinatal nurse assessments were available<br><br>**Verbal information received from John Morrison (March 24/11)**<br>A daily telephone call was made by a perinatal nurse. Subjects were questioned about the signs and symptoms of preterm labor (e.g., contraction, tightening, cervical changes) and were also asked open-ended questions. (same as Morrison 2003)<br><br>MODERATE	Monitored uterine activity twice a day.<br><br>MODERATE	**Verbal information received from John Morrison (March 24/11)**<br>At least one home visit was made by a nurse to set up the SQ terbutaline pump.<br><br>MODERATE	**Verbal information received from John Morrison (March 24/11)**<br>Educated about the signs and symptoms of preterm labor "Women in the study and control groups were taught the signs and symptoms associated with preterm labor." (same as Morrison 2003)<br><br>Education also provided during daily calls by nurse.<br><br>LOW	**Verbal information received from John Morrison (March 24/11)**<br>Patients were given a 24-hour hotline number to call if they had any questions. (same as Morrison 2003)<br><br>HIGH	**Verbal information received from John Morrison (March 24/11)**<br>Bed rest and interdiction of intercourse advised. (same as Morrison 2003)<br><br>MODERATE	**Verbal information received from John Morrison (March 24/11)**<br>Assessed for signs and symptoms of preterm labour and provided education: this was repeated every 1–2 weeks. During this time patients were also questioned further, education was reconfirmed, and more tests may have been performed. (same as Morrison 2003)<br><br>MODERATE	MODERATE

Table F15. Ratings for level of maternal care (continued)

First Author (Year)	Nursing Assessments	Home Uterine Activity Monitoring	Home Visits	Education About Preterm Labor	Telephone Support	Restriction of Maternal Activities	Other Cointerventions	Overall Rating
RETROSPECTIVE COHORTS								
Flick (2010)[14]	To identify barriers to care or issues that may make it difficult for the patients to comply with plan of care.<br>**MODERATE**	An electronic device used to monitor minimum of twice per day and as needed for PTL symptoms. Data transmitted by telephone to a care center and interpreted by perinatal nurses.<br>**HIGH**	Initial home visit by an experienced perinatal nurse to provide written and verbal education about condition.<br>**MODERATE**	Initial home visit by an experienced perinatal nurse to provide written and verbal education about condition.<br>**HIGH**	Available 24 hours a day, 7 days a week, by perinatal nurses and pharmacists.<br>**HIGH**	Not reported<br>**INSUFFICIENT DATA TO MAKE RATING**	Not reported<br>**INSUFFICIENT DATA TO MAKE RATING**	**HIGH**
de la Torre (2008)[15]	Nursing assessment to identify barriers to care.<br>**HIGH**	Patients monitored uterine contractions minimum of twice/day and as needed for PTL symptoms. This data was transmitted by telephone to a care center and interpreted by a perinatal nurse.<br>**HIGH**	A perinatal nurse conducted an initial visit to each patient's home<br>**MODERATE**	A perinatal nurse conducted an initial visit to each patient's home to provide written and verbal education about her condition (review of signs and symptoms of preterm labor, medication compliance, adverse effects, electronic uterine contraction monitor, clinical protocols).<br>**HIGH**	Telephone support by nurses and pharmacists available 24 hours/day 7 days/week.<br>**HIGH**	Not reported<br>**INSUFFICIENT DATA TO MAKE RATING**	Not reported<br>**INSUFFICIENT DATA TO MAKE RATING**	**HIGH**

Table F15. Ratings for level of maternal care (continued)

First Author (Year)	Nursing Assessments	Home Uterine Activity Monitoring	Home Visits	Education About Preterm Labor	Telephone Support	Restriction of Maternal Activities	Other Cointerventions	Overall Rating
Fleming (2004)[16]	Adherence to the prescribed regimen was encouraged, assessed, and documented daily.<br>**MODERATE**	Uterine contraction data collected at least twice daily and were transmitted to a perinatal center staffed with nurses who evaluated the data and completed a telephone assessment of signs and symptoms.<br>**HIGH**	Initial home visit and followup visits conducted as needed.<br>**MODERATE**	Individual patient teaching sessions with a nurse about the signs and symptoms of preterm labor.<br>**HIGH**	Perinatal nurses were available 24 hours/day for 7 days/week for data evaluation, patient calls, and nursing support.<br>**HIGH**	Not reported<br>**INSUFFICIENT DATA TO MAKE RATING**	Not reported<br>**INSUFFICIENT DATA TO MAKE RATING**	HIGH
Lam (2003)[17]	Daily nursing assessments of electronically transmitted uterine activity data and assessment of patients' clinical condition. The extent of adherence to the prescribed regimen was also assessed and adherence encouraged during each nurse-patient contact.<br>**MODERATE**	Use of a monitoring device for uterine contractions and data electronically transmitted.<br>**Information received from Fung Lam April 4/11**<br>Uterine activity monitored twice daily and when needed.<br>**HIGH**	**Information received from Fung Lam April 4/11**<br>Initial home visit by an experienced perinatal nurse to provide written and verbal education about condition.<br>**MODERATE**	Individual patient teaching sessions with a nurse about the signs and symptoms of preterm labor.<br>**HIGH**	Nursing staff available at all times for patient phone calls.<br>**Information received from Fung Lam April 4/11**<br>24/7 telephone support available.<br>**HIGH**	**Information received from Fung Lam April 4/11**<br>Activity level was prescribed by each patient health care provider and not by the provider of outpatient services.<br>Uterine contractions used to determine tolerated activity level.<br>**HIGH**	**Information received from Fung Lam April 4/11**<br>Tocolysis adjusted for an increase in monitored uterine contractions.<br>Frequency of physician office visits determined by each patient's health care provider.<br>**INSUFFICIENT DATA TO MAKE RATING**	HIGH

Table F15. Ratings for level of maternal care (continued)

First Author (Year)	Nursing Assessments	Home Uterine Activity Monitoring	Home Visits	Education About Preterm Labor	Telephone Support	Restriction of Maternal Activities	Other Cointerventions	Overall Rating
Lam (2001)[18]	Daily telephone nursing assessment of objective patient data and subjective symptoms.<br><br>MODERATE	Home uterine activity monitoring (no further details provided).<br><br>**Information received from Fung Lam April 4/11**<br>Use of a monitoring device for uterine contractions and data electronically transmitted.<br><br>Uterine activity monitored twice daily and when needed.<br><br>HIGH	**Information received from Fung Lam April 4/11**<br>Initial home visit by an experienced perinatal nurse to provide written and verbal education about condition.<br><br>MODERATE	Educated about the signs and symptoms of preterm labor "This program included patient education regarding the signs and symptoms of preterm labor."<br><br>**Information received from Fung Lam April 4/11**<br>Initial home visit by an experienced perinatal nurse to provide written and verbal education about condition.<br><br>HIGH	Daily telephone nursing assessment.<br><br>**Information received from Fung Lam April 4/11**<br>24/7 telephone support available.<br><br>HIGH	**Information received from Fung Lam April 4/11**<br>Activity level was prescribed by each patient healthcare provider and not by the provider of outpatient services.<br><br>Uterine contractions used to determine tolerated activity level.<br><br>HIGH	**Information received from Fung Lam April 4/11**<br>Tocolysis adjusted for an increase in monitored uterine contractions.<br><br>Frequency of physician office visits determined by each patient's health care provider.<br><br>INSUFFICIENT DATA TO MAKE RATING	HIGH

Table F15. Ratings for level of maternal care (continued)

First Author (Year)	Nursing Assessments	Home Uterine Activity Monitoring	Home Visits	Education About Preterm Labor	Telephone Support	Restriction of Maternal Activities	Other Cointerventions	Overall Rating
Allbert (1994)[19]	**Verbal information received from John Morrison (March 24/11)** A daily telephone call was made by a perinatal nurse. Subjects were questioned about the signs and symptoms of preterm labor (e.g. contraction, tightening, cervical changes) and were also asked open-ended questions. (same as Morrison 2003)	Patients conducted home uterine contraction monitoring twice daily.	Home nursing care received by SQ terbutaline group, appears only in this group.<br><br>**Verbal information received from John Morrison (March 24/11)** At least one home visit was made by a nurse to set up the SQ terbutaline pump.	**Verbal information received from John Morrison (March 24/11)** Educated about the signs and symptoms of preterm labor "Women in the study and control groups were taught the signs and symptoms associated with preterm labor." (same as Morrison 2003)<br><br>Education also provided during daily calls by nurse.	Daily phone contact by a perinatal nurse<br><br>**Verbal information received from John Morrison (March 24/11)** Patients were given a 24 hour hotline number to call if they had any questions. (same as Morrison 2003)	Bed rest and prohibition of intercourse advised.	**Verbal information received from John Morrison (March 24/11)** Assessed for signs and symptoms of preterm labour and provided education: this was repeated every 1-2 weeks. During this time patients were also questioned further, education was reconfirmed, and more tests may have been performed. (same as Morrison 2003)	**PUMP GROUP:** **HIGH** **CONTROL:** **MODERATE**
	**MODERATE**	**MODERATE**	**RATING CANNOT BE MADE DUE TO POTENTIAL CONFOUNDING**	**LOW**	**HIGH**	**MODERATE**	**MODERATE**	

Table F15. Ratings for level of maternal care (continued)

First Author (Year)	Nursing Assessments	Home Uterine Activity Monitoring	Home Visits	Education About Preterm Labor	Telephone Support	Restriction of Maternal Activities	Other Cointerventions	Overall Rating
Regenstein (1993)[20]	Not reported	Not reported	Study included women receiving home nursing care or care by perinatology service, so cannot be sure whether equal number of patients in oral and SQ terbutaline groups received home care.	Not reported	Not reported	Not reported	Not reported	UNCLEAR
	INSUFFICIENT DATA TO MAKE RATING	INSUFFICIENT DATA TO MAKE RATING	RATING CANNOT BE MADE DUE TO POTENTIAL CONFOUNDING	INSUFFICIENT DATA TO MAKE RATING	INSUFFICIENT DATA TO MAKE RATING	INSUFFICIENT DATA TO MAKE RATING	INSUFFICIENT DATA TO MAKE RATING	
CASE SERIES								
Adkins (1993)[21]	Not reported	Uterine self-palpitation was taught as a method for detecting contractions twice daily.	Home infusion therapy nurse-clinician made an initial home visit. F/U care included: weekly appointments with physicians, frequent telephone calls from home infusion therapy nurse-clinician and physician's offices, and home visits as needed.	Patients educated about the signs and symptoms of preterm labor. "Patients received individual instruction from both physicians and nurses regarding the signs and symptoms of preterm labor."	F/U care included: weekly appointments with physicians, frequent telephone calls from home infusion therapy nurse-clinician and physician's offices.	Bed rest recommended when there was an increase in uterine contractions.	Standard nonpharmacologic al interventions, such as bed rest and oral hydration, were a part of the therapeutic regimen.	MODERATE
	INSUFFICIENT DATA TO MAKE RATING	MODERATE	MODERATE	HIGH	MODERATE	MODERATE	MODERATE	

Table F15. Ratings for level of maternal care (continued)

First Author (Year)	Nursing Assessments	Home Uterine Activity Monitoring	Home Visits	Education About Preterm Labor	Telephone Support	Restriction of Maternal Activities	Other Cointerventions	Overall Rating
Lam (1988)[22]	**Information received from Fung Lam April 4/11** Daily telephone nursing assessment of objective patient data and subjective symptoms.<br><br>MODERATE	Uterine activity was monitored at least twice daily and data was transmitted to study center.<br><br>**Information received from Fung Lam April 4/11** Home uterine activity monitoring .<br><br>Use of a monitoring device for uterine contractions and data electronically transmitted.<br><br>Uterine activity monitored twice daily and when needed.<br><br>HIGH	Weekly followup home visits were carried out by perinatal nurses.<br><br>**Information received from Fung Lam April 4/11** Initial in-hospital and home visit by study nurse (an experienced perinatal nurse) to provide written and verbal education about condition When patient was at home, there were weekly nursing home visits.<br><br>HIGH	**Information received from Fung Lam April 4/11** Educated about the signs and symptoms of preterm labor "This pilot program included patient education regarding the signs and symptoms of preterm labor."<br><br>Initial in-hospital and home visit by study nurse (an experienced perinatal nurse) to provide written and verbal education about condition When patient was at home, there were weekly nursing home visits.<br><br>HIGH	**Information received from Fung Lam April 4/11** Daily telephone nursing assessment by study nurse.<br><br>24/7 telephone support available.<br><br>HIGH	Patients were instructed to remain in bed, but were permitted bathroom privileges.<br><br>**Information received from Fung Lam April 4/11** Activity level was prescribed by study physiciand There were no providers of terbutaline pump outpatient services at this time.<br><br>Uterine contractions used to determine tolerated activity level.<br><br>HIGH	Patients noted their perceived uterine activity on daily preterm labor logs.<br><br>**Information received from Fung Lam April 4/11** Activity level was prescribed by study physiciand There were no providers of terbutaline pump outpatient services at this time.<br><br>Uterine contractions used to determine tolerated activity level.<br><br>HIGH	HIGH

RCT = randomized controlled trial; SQ = subcutaneous

Table F16. Studies that reported pump-related outcomes (Key Question 6)

Outcome	First Author (Year)	Study Design (n=Sample Size)	Mean Maternal Age (Years)	Mean GA (Weeks)*	Comparator(s)	Results—SQ Terbutaline Pump: % (n/N)	Results—Comparison: % (n/N)	Results—OR (95% CI)
Dislodgment	Adkins (1993)[21]	Case series (n=51)	31.0 ± 4.0	29.1 ± 3.6 (T)	No comparator	2.0% (1/51)	N/A	(exact central CI, 0.5%, 10%)
Missed doses	N/A	N/A	N/A	N/A	N/A	N/A	N/A	N/A
Overdose	N/A	N/A	N/A	N/A	N/A	N/A	N/A	N/A
Other:								
Infusion site infection	Lam (1988)[22]	Case series (n=9)	NR	29.6 ± 3.7 (T)	No comparator	0% (0/9)	N/A	N/A
Local pain	Wenstrom (1997)[10]	RCT (n=42)	26.2 ± 5.3	30.4 ± 2.3 (T)	Placebo (C_1)<br>Oral terbutaline (C_2)	13.3% (2/15)	C_1:17% (2/12)<br>C_2: 0% (0/15)	0.77 (0.09, 6.45)<br>5.74 (0.25, 130.38)
Local skin irritation	Wenstrom (1997)[10]	RCT (n=42)	26.2 ± 5.3	30.4 ± 2.3 (T)	Placebo (C_1)<br>Oral terbutaline (C_2)	6.7% (1/15)	C_1: 0% (0/12)<br>C_2: 0% (0/15)	2.59 (0.10, 69.34)<br>3.21 (0.12, 85.21)
Pump malfunction/ mechanical failures and complications	Lam (1988)[22]	Case series (n=9)	NR	29.6 ± 3.7 (T)	No comparator	0% (0/9)	N/A	N/A
	Adkins (1993)[21]	Case series (n=51)	31.0 ± 4.0	29.1 ± 3.6 (T)	No comparator	2.0% (1/51)	N/A	(exact central CI, 0.5%, 10%)

CI = confidence interval; GA = gestational age; N/A = not applicable; NR = not reported; OR = odds ratio; RCT = randomized controlled trial; SQ = subcutaneous* Either at preterm labor (indicated by P) or at start of subcutaneous terbutaline therapy (indicated by T). If study population stated RPTL as an inclusion criterion, then this is the gestational age at the episode of RPTL.

Appendix G. Modifications to Key Questions

Based on comments received during the peer-review process, we made modifications to the format of the Key Questions, as described below:

- The ordering of outcomes within each Key Question
- The format of definitions for extremely preterm, very preterm, preterm, and later preterm (e.g., modification from women between 28 +0 and 31 +6 weeks of gestation to women between 28 weeks, 0 days and 31 weeks, 6 days of gestation)
- Addition of the word “ethnic” to subgroup f
- Addition of the word “surrogate” to Key Question 2
- WDAE changed to Withdrawal-AE in Key Question 3
- Addition of the term “terbutaline-related” harms to Key Question 4
